# Photocatalytic carbanion generation from C–H bonds – reductant free Barbier/Grignard-type reactions†

**DOI:** 10.1039/c9sc04987h

**Published:** 2019-11-12

**Authors:** Anna Lucia Berger, Karsten Donabauer, Burkhard König

**Affiliations:** Institute of Organic Chemistry, Faculty of Chemistry and Pharmacy, University of Regensburg Universitätsstraße 31 93053 Regensburg Germany burkhard.koenig@ur.de

## Abstract

We report a redox-neutral method for the generation of carbanions from benzylic C–H bonds in a photocatalytic Grignard-type reaction. The combination of photo- and hydrogen atom transfer (HAT) catalysis enables the abstraction of a benzylic hydrogen atom, generating a radical intermediate. This radical is reduced *in situ* by the organic photocatalyst to a carbanion, which is able to react with electrophiles such as aldehydes or ketones, yielding homobenzylic secondary and tertiary alcohols.

## Abstract 2

We report a redox-neutral method for the generation of carbanions from benzylic C–H bonds in a photocatalytic Grignard-type reaction.

## Graphical abstract

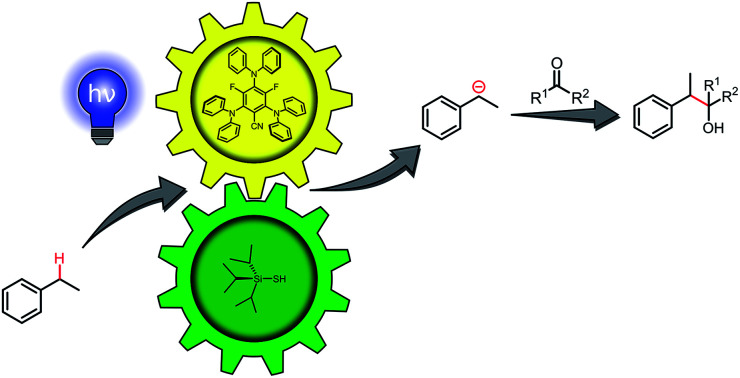

## Introduction

Novel catalytic methods generally aim to produce a desired chemical compound from ever-simpler starting materials, maximizing the atom and step economy.^1^ Hence, the functionalization of C–H bonds has received great attention, as it illustrates the most straightforward retrosynthetic path for the synthesis of a targeted product.^2^ There are several methods for C–H functionalizations summarized in comprehensive reviews.^3^ A prominent example is the C–H activation by metal insertion,^3*c*–*f*^ comprising cases of very high and catalyst controlled regioselectivity.^4^ Another prevalent method is hydrogen atom transfer,^3*g*^ which is used to generate carbon centred radicals for subsequent functionalization from unreactive C–H bonds by the abstraction of a hydrogen atom.

Recently, the combination of hydrogen atom transfer (HAT) and photocatalysis has evolved into a powerful method yielding carbon radicals under mild conditions often without the need of a sacrificial oxidant or reductant.^5^ With this approach, several impressive examples for C–C and C–X bond formations were reported, utilizing C–H bonds in order to arrive at the desired product in high or even full atom economy.^6^

**Scheme 1:**
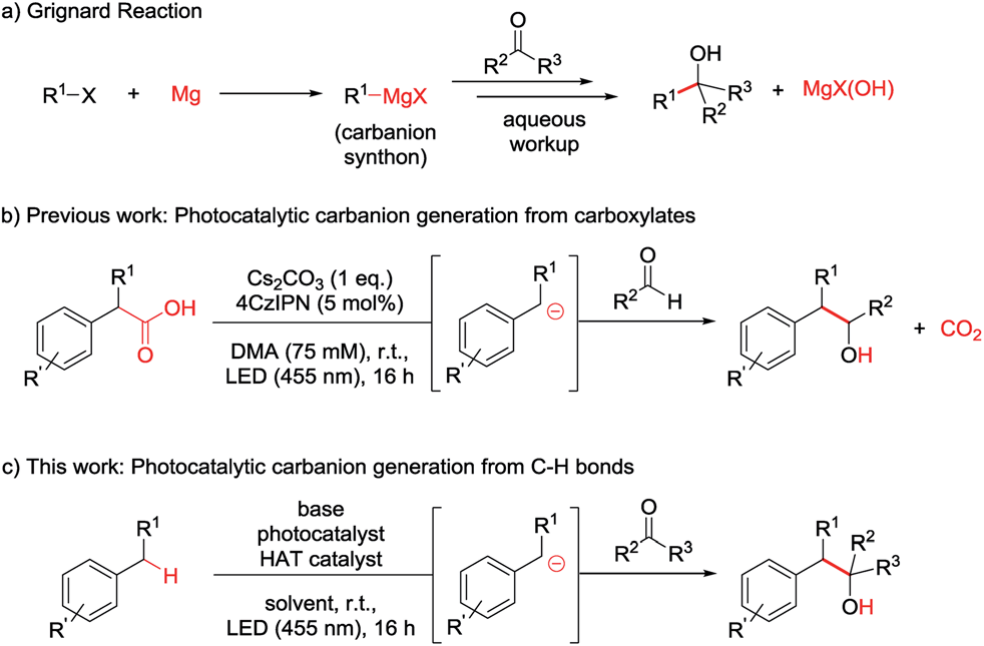
(a) Grignard reaction. (b) Photocatalytic carbanion generation from carboxylates and addition to aldehydes. (c) Envisioned photocatalytic carbanion generation from C–H bonds for Grignard-type reactions in full atom economy.

While photocatalysis, especially in combination with HAT catalysis, mainly revolves around the generation and subsequent reaction of radical species,^7^ some groups have recently proposed the generation of carbanions as crucial intermediates in photocatalytic transformations.^7*a*,8^ The formation of carbanionic intermediates is of particular interest as they are the reactive intermediates in the widely used Grignard and Barbier reactions (Scheme 1a).^9^ However, these reactions produce stoichiometric amounts of metal salt waste^9*c*^ and require organohalide starting materials which often have to be prepared.^10^

In our previous report we aimed to overcome those drawbacks by using carboxylates to generate carbanionic intermediates in a photocatalytic reaction (Scheme 1b).^8*g*^ However, only aldehydes were efficient electrophiles and CO_2_ was released as a stoichiometric by-product. Developing this method further, we wondered if C–H bonds could directly be activated to form the desired Grignard analogous products, maximizing the atom economy.

The most straightforward C–H activation giving potential access to carbanion intermediates from unfunctionalized starting materials is the deprotonation of the respective C–H bond. However, with a p*K*_a_ value of approximately 43 (in DMSO),^11^ even benzylic C–H bonds would require the use of highly active bases like *n*-BuLi (p*K*_a_ approx. 50) exceeding *e.g.* LDA (p*K*_a_ = 36 in THF)^12^ in reactivity, which limits the functional group tolerance and gives rises to potential side reactions. Additionally, many of these strong bases can directly add to carbonyl compounds or be quenched by the deprotonation of the more acidic proton in alpha position of the carbonyl (p*K*_a_ of acetone = 26 in DMSO),^13^ which may also be the case for the desired benzyl anion. Additionally, waste products resulting from the use of metal bases again diminish the atom economy. The generation of carbanions by the combination of HAT- and photocatalysis could overcome these issues and illustrates a valuable method for a redox-neutral, waste-free synthesis of Grignard-type products without the use of metals or strong bases (Scheme 1c).

In a recent report, our group could show the applicability of this concept for the photocarboxylation of benzylic C–H bonds *via* carbanionic intermediates.^14^ In this work, we aim to extend this method to the synthesis of secondary and tertiary homobenzylic alcohols from unfunctionalized starting materials and aldehydes or ketones in a photocatalytic two-step deprotonation reaction.

## Results and discussion

We chose ethylbenzene (**1a**) as model substrate, because its benzylic C–H bonds have a low bond dissociation energy (BDE = 85.4 kcal mol^−1^)^15^ and benzylic radicals can be converted into the corresponding carbanion by single electron transfer (SET) using a reduced photocatalyst.^8*g*^ Acetone (**2a**) was chosen as electrophile, as ketones do not bear a carbonyl hydrogen, which has shown to be prone to C–H abstraction by electrophilic radicals.^16^

Product formation was observed using a combination of 4CzIPN (**A**) as photocatalyst and (^*i*^Pr)_3_SiSH as HAT catalyst. Together with K_2_CO_3_ as base and dry MeCN as solvent, the coupling product (**3a**) between **1a** and **2a** was detected in traces (Table 1, entry 1). A higher yield of 21% was obtained by adding grinded 4 Å molecular sieves to the reaction (Table 2, entry 2). Increasing the amount of **2a** by using it as a co-solvent in a 1 : 1 mixture with dry acetonitrile gave a yield of 49% (Table 1, entry 3). Reducing the amount of (^*i*^Pr)_3_SiSH and molecular sieves gave a slightly enhanced yield (Table 1, entry 4). Using 3DPA2FBN (**B**) as a photocatalyst increased the yield to 50% when 10 eq. **2a** were used and 86% when acetone was used as a co-solvent (Table 1, entries 5 and 6). The reaction improved slightly by reducing the loading of photocatalyst **B** to 3 mol% and the amount of K_2_CO_3_ to 10 mol% (Table 1, entry 7). Control experiments showed, that the yield is significantly lower when the reaction is performed without base (Table 1, entry 8) and no product was detected in absence of light, photocatalyst or HAT catalyst (Table 1, entries 9–11).

**Table 1:** Optimization of the reaction conditions for the photocatalytic HAT-reaction of ethylbenzene with acetone as an electrophile^a^

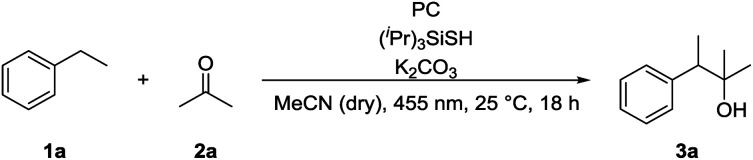						
Entry	Amount of **2a**	Photocatalyst (mol%)	Amount of (^*i*^Pr)_3_SiSH	Amount of base	Additive	Yield^b^ [%]
1	10 eq.	4CzIPN (5)	20 mol%	20 mol%	—	3
2	10 eq.	4CzIPN (5)	20 mol%	20 mol%	4 Å MS (100 mg)	21
3	Co-solvent (1 : 1)	4CzIPN (5)	20 mol%	20 mol%	4 Å MS (100 mg)	49
4	10 eq.	4CzIPN (5)	10 mol%	20 mol%	4 Å MS (50 mg)	30
5	10 eq.	3DPA2FBN (5)	10 mol%	20 mol%	4 Å MS (50 mg)	50
6	Co-solvent (1 : 1)	3DPA2FBN (5)	10 mol%	20 mol%	4 Å MS (50 mg)	86
7	10 eq.	3DPA2FBN (3)	10 mol%	10 mol%	4 Å MS (50 mg)	59
8	10 eq.	3DPA2FBN (5)	10 mol%	—	4 Å MS (50 mg)	27
9	10	—	10 mol%	20 mol%	4 Å MS (100 mg)	0
10^c^	10	4CzIPN (5)	10 mol%	20 mol%	4 Å MS (100 mg)	0
11	10	4CzIPN (5)	—	20 mol%	4 Å MS (100 mg)	0
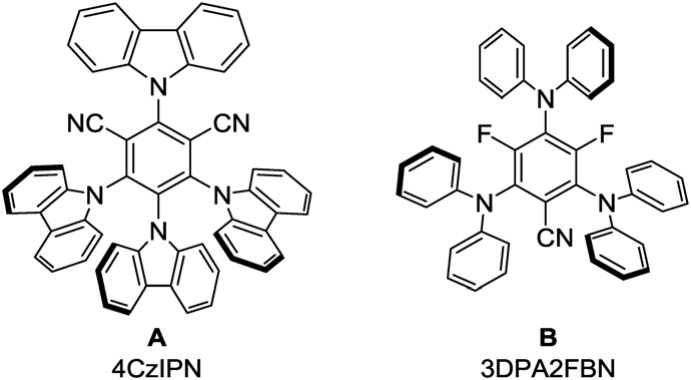						

a The reaction was performed using 1 eq. (0.2 mmol) **1a** in 2 mL degassed solvent.

b Yields were determined with GC-FID analysis using *n*-decane as an internal standard.

c Reaction was performed in the dark.

**Table 2:** Investigations of product inhibition of the reaction^a^

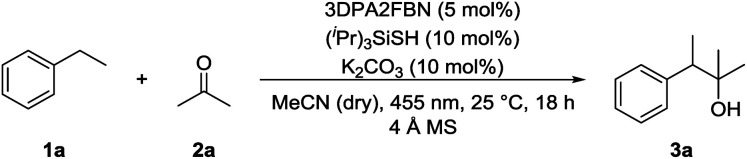		
Entry	Additive	Yield^b^ [%]
1^c^	3DPA2FBN (5 mol%)	41
2^c^	(^*i*^Pr)_3_SiSH (10 mol%)	50
3^c^	3DPA2FBN (3 mol%)	60
	(^*i*^Pr)_3_SiSH (10 mol%)	
4	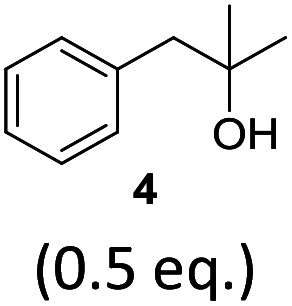	39
5	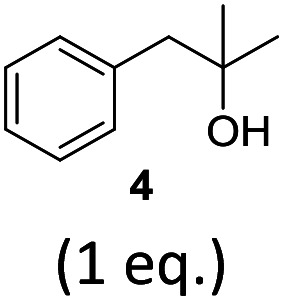	11
6	1-Heptanol (1 eq.)	21

a The reaction was performed using 1 eq. (0.2 mmol) **1a** and 10 eq. **2a** in 2 mL degassed solvent.

b Yields were determined with GC-FID analysis using *n*-decane as an internal standard.

c Additional catalyst was added after 14 h.

**Fig. 1:**
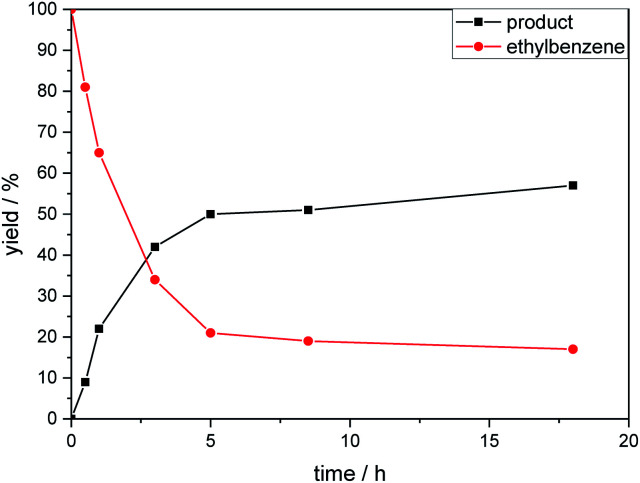
Product formation and consumption of starting material during the reaction.

The kinetic profile of the reaction shows a quite fast linear increase of product formation in the first hours. However, after 5 hours, the conversion of starting material stops at a product yield of 50 to 55%, which increased only slightly by prolonging the reaction time (Fig. 1).

To exclude the possibility, that the termination of the reaction is caused by the decomposition of either the photocatalyst or the hydrogen atom transfer catalyst, both compounds were added to the reaction separately or in combination after several hours (Table 2, entries 1–3). However, the yield of the desired product **3a** could not be increased for any of the combinations. To test if the reaction was inhibited by the formation of the product, 2-methyl-1-phenyl-2-propanol **4** was added due to its structural similarity to product **3a**. Indeed, the yield decreased to 39% when 0.5 eq. **4** was added and to 11% with 1 eq. **4** (Table 2, entries 4 and 5). The addition of 1 eq. 1-heptanol also decreased the yield to 21% (Table 2, entry 6), indicating that the presence of alcohols causes the reaction to stop, presumably due to the protic hydroxy groups quenching the carbanion.

The scope of the reaction was investigated for various ethylbenzene derivatives, ketones and aldehydes (Table 3). In most cases, good yields were obtained when the electrophile acetone was used as a co-solvent in a 1 : 1 mixture with acetonitrile, while using 10 eq. of electrophile led to moderate yields. Besides ethylbenzene **1a** (41%/72%, **3a**), 4- or 2-ethyltoluene were also viable substrates for the reaction (**3b** and **3c**). Notably, 4-ethyltoluene **1b** was the only substrate where using less electrophile seemed to be beneficial for the reaction, as a yield of 62% was obtained for 10 eq. **2a**, while using acetone as a co-solvent only lead to 55% of the desired product **3b**. Using cumene **1d** decreased the yield to 29% (11% with 10 eq. **2a**), presumably due to enhanced steric hindrance in the benzylic position (**3d**). The reaction proceeded well with isopentylbenzene **1e**, yielding the corresponding product **3e** in 47% and 79%, respectively. Ethylbenzene derivatives containing electron donating substituents, such as methoxy- (**3f–3i**) or amide-groups (**3j**) led to significantly increased yields of up to 87% (**3f** and **3j**). In contrast, no product was obtained with electron deficient substrates such as 4-ethylbenzonitrile or 1-ethyl-4-(trifluoromethyl)benzene, presumably due to a kinetically more hindered hydrogen atom abstraction^17^ or the lower reactivity of the corresponding carbanion intermediate. While unsubstituted toluene did not lead to any product formation due to the bond dissociation energy of the benzylic C–H bond exceeding the capability of the hydrogen atom transfer catalyst (toluene: BDE = 89 kcal mol^−1^, (^*i*^Pr)_3_SiSH: BDE = 87 kcal mol^−1^),^18^ 4-methoxytoluene **1i** gave the corresponding product **3i** in 19% and 53%, respectively. Chlorine and fluorine substituents at the aromatic ring were also well tolerated in the reaction (**3k** and **3l**) and using triethylbenzene **1m** led to 87% of the triple substituted product **3m** when acetone was used as a co-solvent. For this substrate, no product could be isolated when only 10 eq. **2a** was used, as an inseparable mixture of single, double and triple substituted product was obtained. *p*-Phenyl substituted ethylbenzene could also be used in the reaction, yielding 62% of product **3n** (31% with 10 eq. **2a**). In contrast, 2-ethylnaphthalene **1o** gave only low yields of 7% and 22%, respectively (**3o**). Heteroaromatic substrates were also viable substrates for the reaction as moderate to good yields were obtained when 2-ethylthiophene **1p** or -benzofurane **1q** were used (**3p** and **3q**). Moving to ketones, the effect of steric hindrance was investigated first. A good yield can still be obtained when the carbon chain is extended at on side (**3r**), whereas the yield is notably affected when both sides bear longer chains (**3s** and **3t**) or an additional group is present in α-position (**3u** and **3v**). No ring opening products were observed when a cyclopropane ring was present in α-position, indicating that no radical processes are involved in the addition to the electrophile. The reaction proceeds well with cyclic ketones (**3w** and **3x**), especially with cyclobutanone (**3x**), altogether displaying the significant influence of steric hindrance. In terms of functional group tolerance, alkenes (**3y**), alkyl chlorides (**3z**), ethers (**3aa**), esters (**3ab**) and protected amines (**3ac**) are viable substrates. However, the amount of electrophile has to be reduced in these cases, causing a decrease in yield. Notably, if an α,β-unsaturated system is used, the 1,4-addition product (**3ad**) is obtained, while the 1,2-addition product was not observed. As noted above, aldehydes are prone to C–H abstraction from the carbonyl position,^16^ seemingly leading to deleterious side reactions. Hence, the reaction conditions were adapted, mainly by using an excess of the ethyl benzene instead of the electrophile (see ESI for all optimization parameters†). Under the modified reaction conditions, aldehydes are feasible substrates, but yields are generally only low to moderate (up to 43% for **6a**). As with ketones, steric hindrance has a significant effect (**6a–6e**). Thioethers are tolerated (**6f**) despite the presence of C–H bonds in α-position to the heteroatom. Further, employing aromatic aldehydes gave the desired products as well (**6g** and **6h**), and the yield increased with an additional electron withdrawing ester group (**6h**).

**Table 3:** Scope of the reaction

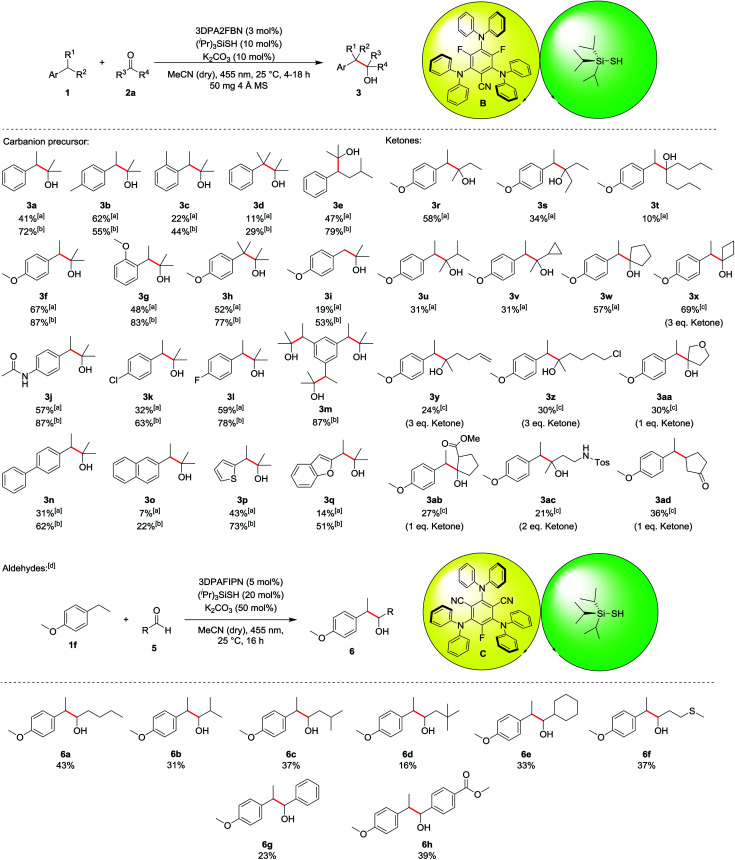

a The reaction was performed using 1 eq. (0.2 mmol) **1** and 10 eq. of the respective ketone in 2 mL dry, degassed MeCN.

b The reaction was performed using 1 eq. (0.2 mmol) **1** and **2a** as co-solvent in a 1 : 1 mixture with dry MeCN in 2 mL degassed solvent mixture.

c The reaction was performed using 1 eq. (0.2 mmol) **1** and the respective ketone in the amount given in the table in 2 mL dry, degassed MeCN.

d The reaction was performed using 1 eq. (0.15 mmol) **5** and 3 eq. 1f in 2 mL dry, degassed MeCN.

**Scheme 2:**
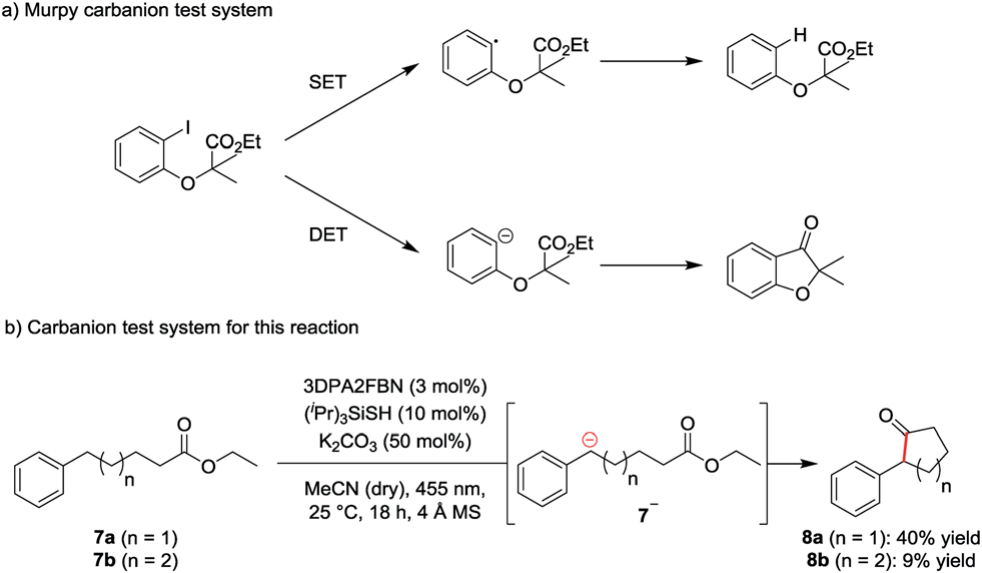
Carbanion test system (a) developed by Murphy *et al.* for the detection of aryl anions and (b) test system used for this reaction.

To investigate the mechanism of the reaction, a carbanion test system based on a molecule used by Murphy *et al.* to confirm the generation of aryl anions (Scheme 2a) was used.^19^ According to Murphy, radicals are not capable of adding to esters. Therefore, ethyl-5-phenylpentanoate **7a** was subjected to the standard reaction conditions. The formation of the cyclic ketone **8a** indicates the presence of the anionic intermediate **7a−** (Scheme 2b).

In addition to this, fluorescence quenching studies were performed to confirm the interaction of the excited state of the photocatalyst with the deprotonated HAT catalyst (^*i*^Pr)_3_SiS^−^. Efficient fluorescence quenching was observed for the photocatalysts **B** and **C** upon addition of (^*i*^Pr)_3_SiS^−^, indicating the oxidation of the deprotonated hydrogen atom transfer catalyst by the excited state of the photocatalyst (ESI, Fig. S3 and S4†). To further confirm this, cyclic voltammetry measurements were performed (ESI, Fig. S5†). Indeed, a potential of 0.67 V *vs.* SCE in MeCN was obtained for a 1 : 2 mixture of (^*i*^Pr)_3_SiSH and K_2_CO_3_ which is well in the range of photocatalyst **B** and **C** (*E*_1/2_(3DPA2FBN*/3DPA2FBN˙^−^) = 0.92 V *vs.* SCE, *E*_1/2_(3DPAFIPN*/3DPAFIPN˙^−^) = 1.09 V *vs.* SCE).^20^ Lastly, the formation of benzylic radicals (**1•**) during the reaction is indicated by the presence of small amounts of the homocoupling product **9** in the reaction mixture (ESI, Fig. S6†).

**Scheme 3:**
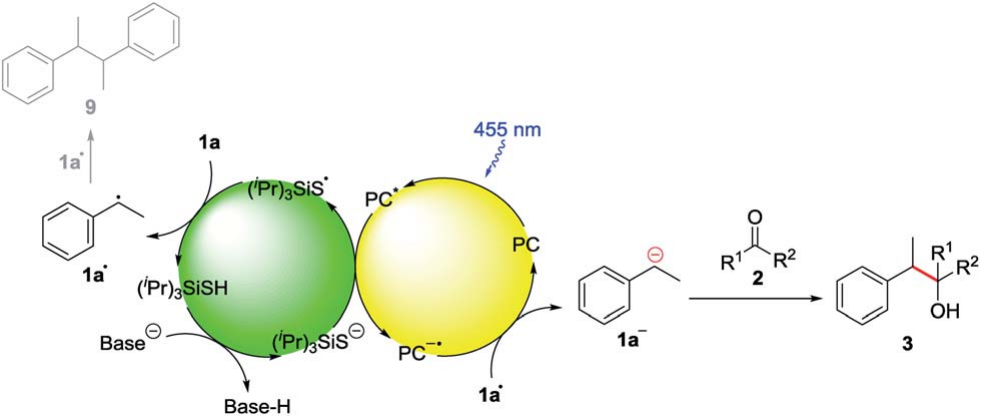
Proposed reaction mechanism.

Based on these mechanistic investigations, the reaction mechanism depicted in Scheme 3 is proposed. The photocatalyst is excited upon irradiation with blue light and after deprotonation with K_2_CO_3_, (^*i*^Pr)_3_SiS^−^ can be oxidized to (^*i*^Pr)_3_SiS˙ by a SET to the excited photocatalyst PC*. The generated sulfur radical is capable of abstracting a hydrogen atom from ethylbenzene **1a**, generating the benzylic radical **1a•** (**1a**: BDE = 85.4 kcal mol^−1^,^15^ (^*i*^Pr)_3_SiSH: BDE = 87 kcal mol^−1^).^18^ Compound **1a•** (*E*_1/2_(**1a•**/**1a−**) = 1.60 V *vs.* SCE)^21^ can be reduced by the radical anion of the photocatalyst PC˙^−^ (*E*_1/2_(3DPA2FBN/3DPA2FBN^−^) = −1.92 V *vs.* SCE, *E*_1/2_(3DPAFIPN/3DPAFIPN^−^) = −1.59 V *vs.* SCE),^20^ thus closing the photocatalytic cycle. The resulting benzylic anion **1a−** reacts with electrophiles like aldehydes or ketones, leading to the desired product **3**.

## Conclusions

In summary, we have developed a method for the photocatalytic generation of carbanions from benzylic C–H bonds, which react with electrophiles, such as aldehydes or ketones, to generate homobenzylic alcohols as products. The reaction represents a formal two-step deprotonation of the non-acidic benzylic C–H bond and could be a mechanistic alternative to classic C–C bond forming reactions such as the Grignard or Barbier reaction, giving the same products. However, instead of using stoichiometric amounts of a zero-valent metal and halogenated precursor, an organic photocatalyst, catalytic amounts of a hydrogen atom transfer reagent and visible light are used to generate carbanionic intermediates directly from C–H bonds, yielding the desired product in a redox neutral reaction with full atom economy.

## Conflicts of interest

There are no conflicts to declare.

## Supplementary Material

SC-010-C9SC04987H-s001

## References

[cit1] Hendrickson J. B. (1975). J. Am. Chem. Soc..

[cit2] Yamaguchi J., Yamaguchi A. D., Itami K. (2012). Angew. Chem., Int. Ed..

[cit3] Poulsen T. B., Jorgensen K. A. (2008). Chem. Rev..

[cit4] Liu W., Ren Z., Bosse A. T., Liao K., Goldstein E. L., Bacsa J., Musaev D. G., Stoltz B. M., Davies H. M. L. (2018). J. Am. Chem. Soc..

[cit5] Protti S., Fagnoni M., Ravelli D. (2015). ChemCatChem.

[cit6] Hager D., MacMillan D. W. (2014). J. Am. Chem. Soc..

[cit7] Zhang Y., Qian R., Zheng X., Zeng Y., Sun J., Chen Y., Ding A., Guo H. (2015). Chem. Commun..

[cit8] Liao L. L., Cao G. M., Ye J. H., Sun G. Q., Zhou W. J., Gui Y. Y., Yan S. S., Shen G., Yu D. G. (2018). J. Am. Chem. Soc..

[cit9] (c) Silverman G. S. and Rakita P. E., Handbook of Grignard Reagents, Marcel Dekker, Inc., New York, 1996

[cit10] Ni S., Padial N. M., Kingston C., Vantourout J. C., Schmitt D. C., Edwards J. T., Kruszyk M. M., Merchant R. R., Mykhailiuk P. K., Sanchez B. B., Yang S., Perry M. A., Gallego G. M., Mousseau J. J., Collins M. R., Cherney R. J., Lebed P. S., Chen J. S., Qin T., Baran P. S. (2019). J. Am. Chem. Soc..

[cit11] Bordwell F. G., Algrim D., Vanier N. R. (1977). J. Org. Chem..

[cit12] Chatterjee K., Miyake M., Stock L. M. (1990). Energy Fuels.

[cit13] Bordwell F. G. (2002). Acc. Chem. Res..

[cit14] Meng Q. Y., Schirmer T. E., Berger A. L., Donabauer K., König B. (2019). J. Am. Chem. Soc..

[cit15] Cuthbertson J. D., MacMillan D. W. (2015). Nature.

[cit16] Banerjee A., Lei Z., Ngai M. Y. (2019). Synthesis.

[cit17] Le C., Liang Y., Evans R. W., Li X., MacMillan D. W. C. (2017). Nature.

[cit18] Tanaka H., Sakai K., Kawamura A., Oisaki K., Kanai M. (2018). Chem. Commun..

[cit19] Murphy J. A., Zhou S. Z., Thomson D. W., Schoenebeck F., Mahesh M., Park S. R., Tuttle T., Berlouis L. E. (2007). Angew. Chem., Int. Ed..

[cit20] Speckmeier E., Fischer T. G., Zeitler K. (2018). J. Am. Chem. Soc..

[cit21] Wayner D. D. M., McPhee D. J., Griller D. (1988). J. Am. Chem. Soc..

