# Supplementary material for: Photocatalytic carbanion generation from C–H bonds – reductant free Barbier/Grignard-type reactions
Source: Chem Sci. 2019 Nov 12;10(48):10991–6. doi: 10.1039/c9sc04987h (PMC8133029; doi:10.1039/c9sc04987h)
Supplement: SC-010-C9SC04987H-s001 [file SC-010-C9SC04987H-s001.pdf]

Electronic Supplementary Material (ESI) for Chemical Science.
This journal is © The Royal Society of Chemistry 2019

Table of Contents

Supporting information 2

1. General information 2
2. General procedures 4
   - 2.1. Synthesis of photocatalysts 4
   - 2.2. Synthesis of starting materials 9
   - 2.3. General procedure for the photocatalytic generation of carbanions from benzylic C–H bonds 11
3. Detailed optimization of the reaction conditions 33
   - 3.1. Optimization process with ketones as electrophiles 33
   - 3.2. Optimization process with aldehydes as electrophiles 37
4. Unsuccessful transformations 44
5. Mechanistic investigations 46
   - 5.1. Reaction kinetics 46
   - 5.2. Emission quenching studies (support for SET oxidation of HAT- by excited photocatalyst) 47
   - 5.3. Cyclic voltammetry measurements (further support for the feasibility of SET oxidation of the HAT- by the photocatalyst) 48
   - 5.4. Radical-radical homocoupling (support for C–H abstraction by activated HAT catalyst). 49
   - 5.5. Intramolecular ring closure using an ester as an electrophile (support for carbanion formation) 50
6. NMR-spectra 51
7. References 104

Supporting information

1. General information

Starting materials and reagents were purchased from commercial suppliers (Sigma Aldrich, Alfa Aesar, Acros, Fluka, TCI or VWR) and used without further purification. Solvents were used as p.a. grade or dried and distilled according to literature known procedures.[1] For automated flash column chromatography distilled solvents was used. All reactions with oxygen- or moisture-sensitive reagents were carried out in glassware, which was dried before use by heating under vacuum. Dry nitrogen was used as inert gas atmosphere. Liquids were added via syringe, needle and septum techniques unless otherwise stated.

All NMR spectra were measured at room temperature using a Bruker Avance 300 (300 MHz for ^{1}H, 75 MHz for ^{13}C, 282 MHz for ^{19}F) or a Bruker Avance 400 (400 MHz for ^{1}H, 101 MHz for ^{13}C, 376 MHz for ^{19}F)[2] NMR spectrometer. All chemical shifts are reported in δ-scale as parts per million [ppm] (multiplicity, coupling constant *J*, number of protons) relative to the solvent residual peaks as the internal standard.[3] Coupling constants *J* are given in Hertz [Hz]. Abbreviations used for signal multiplicity: ^{1}H-NMR: b = broad, s = singlet, d = doublet, t = triplet, q = quartet, hept = heptet dd = doublet of doublets, dt = doublet of triplets, dq = doublet of quartets, and m = multiplet;
^{13}C-NMR: (+) = primary/tertiary, (–) = secondary, (C_q) = quaternary carbon).

The mass spectrometrical measurements were performed at the Central Analytical Laboratory of the University of Regensburg. All mass spectra were recorded on a Finnigan MAT 95, ThermoQuest Finnigan TSQ 7000, Finnigan MAT SSQ 710 A or an Agilent Q-TOF 6540 UHD instrument.

For the optimization using aldehydes following GC method was used: GC measurements were performed on a GC 6890 from Agilent Technologies. Data acquisition and evaluation was done with Agilent ChemStation Rev.C.01.04. A capillary column DB–WAX UI/30 m x 0.25 mm/0.25 µM film and helium as carrier gas (flow rate of 1 mL/min) were used. The injector temperature (split injection: 30:1 split) was 280 °C, detection temperature 310 °C (FID). GC measurements were made and investigated *via* integration of the signal obtained. The GC oven temperature program was adjusted as follows: initial temperature 40 °C was kept for 3 minutes, the temperature was increased at a rate of 15 °C/min over a period of 12 minutes until 220 °C was reached and kept for 5 minutes, the temperature was again increased at a rate of 25 °C/min over a period of 48 seconds until the final temperature (240 °C) was reached and kept for 5 minutes. 1-Heptanol was used as an internal standard.

For every other use following GC method was used: GC measurements were performed on a GC 7890 from Agilent Technologies. Data acquisition and evaluation was done with Agilent ChemStation

Rev.C.01.04. GC/MS measurements were performed on a 7890A GC system from Agilent Technologies with an Agilent 5975 MSD Detector. Data acquisition and evaluation was done with MSD ChemStation E.02.02.1431.A capillary column HP-5MS/30 m x 0.25 mm/0.25 μM film and helium as carrier gas (flow rate of 1 mL/min) were used. The injector temperature (split injection: 40:1 split) was 280 °C, detection temperature 300 °C (FID). GC measurements were made and investigated via integration of the signal obtained. The GC oven temperature program was adjusted as follows: initial temperature 40 °C was kept for 3 minutes, the temperature was increased at a rate of 15 °C/min over a period of 16 minutes until 280 °C was reached and kept for 5 minutes, the temperature was again increased at a rate of 25 °C/min over a period of 48 seconds until the final temperature (300 °C) was reached and kept for 5 minutes. If noted, *n*-decane was used as an internal standard.

Analytical TLC was performed on silica gel coated alumina plates (MN TLC sheets ALUGRAM® Xtra SIL G/UV_{254}). Visualization was done by UV light (254 or 366 nm). If necessary, potassium permanganate, vanillin or ceric ammonium molybdate was used for chemical staining.

Purification by column chromatography was performed with silica gel 60 M (40-63 μm, 230-440 mesh, Merck) on a Biotage® Isolera™ Spektra One device. For irradiation with blue light OSRAM Oslon SSL 80 LDCQ7P-1U3U (blue, λ_{max} = 455 nm, I_{max} = 1000 mA, 1.12 W) was used.

For irradiation with green light Cree XPEGRN L1 G4 Q4 (green, λ_{max} = 535 nm, I_{max} = 1000 mA, 1.12 W) was used.

CV measurements were performed with the three-electrode potentiostat galvanostat PGSTAT302N from Metrohm Autolab using a glassy carbon working electrode, a platinum wire counter electrode, a silver wire as a reference electrode and TBATFB 0.1 M as supporting electrolyte. The potentials were achieved relative to the Fc/Fc+ redox couple with ferrocene as internal standard.[4] The control of the measurement instrument, the acquisition and processing of the cyclic voltammetric data were performed with the software Metrohm Autolab NOVA 1.10.4. The measurements were carried out as follows: a 0.1 M solution of TBATFB in acetonitrile was added to the measuring cell and the solution was degassed by argon purge for 5 min. After recording the baseline the electroactive compound was added (0.01 M) and the solution was again degassed a stream of argon for 5 min. The cyclic voltammogram was recorded with one to three scans. Afterwards ferrocene (2.20 mg, 12.0 μmol) was added to the solution which was again degassed by argon purge for 5 min and the final measurement was performed with three scans.

Fluorescence spectra were measured on a HORIBA FluoroMax®-4 Spectrofluorometer at room temperature. Gas tight 10 mm Hellma® quartz fluorescence cuvettes with a screw cap with PTFE -coated

silicon septum were used. FluorEssence Version 3.5.1.20 was used as a software for measurement and analysis.

2. General procedures

2.1. Synthesis of photocatalysts

2,4,5,6-Tetrakis(carbazole-9-yl)-4,6-dicyanobenzene (4CzIPN)

The photocatalyst was synthesized according to a literature procedure.[5]

NaH (60% in paraffin oil, 800 mg, 20 mmol, 10 eq.) was slowly added to a stirred solution of carbazole (1.67 g, 10 mmol, 5 eq.) in dry THF (40 mL). The reaction mixture was heated to 35 °C and stirred for 1 h before adding tetrafluoroisophthalonitrile (400 mg, 2 mmol, 1 eq.). The reaction mixture was stirred at 35 °C for 16 h, afterwards quenched by H_2O (2 mL) and concentrated *in vacuo*. The solid residue was washed with H_2O and EtOH to yield the crude product, which was purified by recrystallization from hexane/DCM to give 2,4,5,6-tetrakis(carbazol-9-yl)-4,6-dicyano-benzene (4CzIPN) as bright yellow powder (840 mg, 1.06 mmol, 53%).

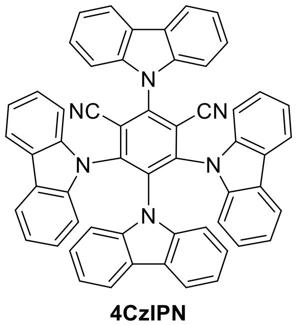

4CzIPN

^{1}H-NMR (400 MHz, $CDCl_3$, δ_H): 8.22 (d, *J* = 7.7 Hz, 2H), 7.75 – 7.67 (m, 8H), 7.52 – 7.47 (m, 2H), 7.33 (d, *J* = 7.8 Hz, 2H), 7.25 – 7.19 (m, 4H), 7.12 – 7.05 (m, 8H), 6.82 (t, *J* = 8.2 Hz, 4H), 6.63 (td, *J* = 7.6, 1.2 Hz, 2H).

^{13}C-NMR (101 MHz, $CDCl_3$, δ_C): 145.3, 144.7, 140.1, 138.3, 137.1, 134.9, 127.1, 125.9, 125.1, 124.9, 124.7, 124.0, 122.5, 122.1, 121.5, 121.1, 120.6, 119.8, 116.5, 111.8, 110.1, 109.6, 109.6.

2,4,6-Tris(diphenylamino)-3,5-difluorobenzonitrile (3DPA2FBN)

The photocatalyst was synthesized according to a literature procedure.[6]

Under nitrogen atmosphere, diphenylamine (1.27 g, 7.5 mmol, 1.25 eq.) was dissolved in dry THF (40 ml) in a flame dried Schlenk flask. Sodium hydride (60% in paraffin oil, 0.45 g, 11.3 mmol, 5.6 eq.) was slowly added and the reaction mixture was stirred at 50 °C for 30 minutes. Pentafluorobenzonitrile (255 µl, 0.39 g, 2 mmol, 1 eq.) was added and the reaction was stirred at room temperature for 24 h. The reaction mixture was quenched with water (2 ml) and concentrated under vacuum. The residue was dissolved in DCM and washed with brine. The organic phase was dried over Na_2SO_4 and the solvent was removed under reduced pressure. Purification of the crude product was performed by flash column chromatography on silica gel (PE/DCM, DCM 20 – 80%) yielding the desired product.

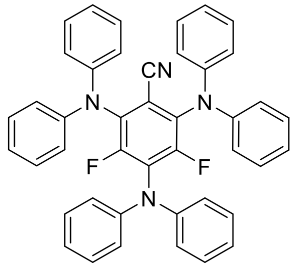

3DPA2FBN

^{1}H NMR (300 MHz, $CDCl_3$, δ_H) 7.26 – 7.20 (m, 12H), 7.07 – 7.00 (m, 6H), 6.99 – 6.95 (m, 12H).

^{19}F NMR (282 MHz, $CDCl_3$, δ_F) -120.72 (s).

HRMS (EI+) (m/z): [$M^{+\bullet}$] ($C_{43}H_{30}F_2N_4^{+\bullet}$) calc.: 640.2433, found: 640.2430.

2,4,6-Tris(diphenylamino)-5-fluoroisophthalonitrile (3DPAFIPN)[6]

The photocatalyst was synthesized analogous to 4CzIPN with 2,3,5,6-tetrafluorobenzonitirl (350 mg, 2 mmol, 1 eq.) instead of carbazole. The crude product was purified by automated flash column chromatography (PE/DCM 20-80%). 2,4,6-Tris(diphenylamino)-5-fluoroisophthalonitrile (3DPAFIPN) (910 mg, 1.40 mmol, 70%) was obtained as bright yellow powder.

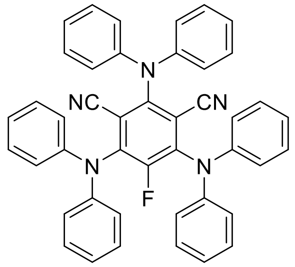

3DPAFIPN

^{1}H-NMR (400 MHz, $CDCl_3$, δ_H): 7.30 – 7.23 (m, 12H), 7.11-7.02 (m, 6H), 7.02 – 6.96 (m, 12H).

^{19}F-NMR (377 MHz, $CDCl_3$, δ_F): -121.83 (s).

Field desorption mass spectra (**FD-MS**) (m/z): [M^+] ($C_{44}H_{30}FN_5^+$) calc. 647.2485; observed 647.1977. FD-MS revealed, that 4DPAFIPN[5] is present in small amount as well (m/z): [M^+] ($C_{56}H_{40}N_6^+$) calc. 796.3314; observed 796.2684. It may be the impurity visible in the NMR.

2,3,5,6-Tetrakis(carbazol-9-yl)benzonitrile (4Cz(*p*H)BN)

The photocatalyst was synthesized analogous to 4CzIPN with diphenylamine (1.69 g, 10 mmol, 5 eq.) instead of tetrafluoroisophthalonitrile. The crude product was purified by automated flash column chromatography (PE/DCM 20-80%). 2,3,5,6-Tetrakis(carbazol-9-yl)benzonitrile (4Cz(*p*H)BN) (1.00 g, 1.31 mmol, 66%) was obtained as pale yellow powder.

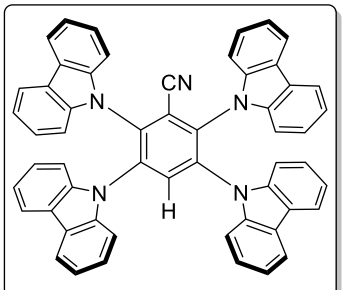

4Cz(*p*H)BN

^{1}H-NMR (300 MHz, $CDCl_3$, δ_H): 8.44 (s, 1H), 7.82-7.74 (m, 8H), 7.39-7.280 (m, 8H), 7.23-7.08 (m, 16H).

^{13}C-NMR (75 MHz, $CDCl_3$, δ_C): 139.3, 139.0, 137.9, 136.7, 125.9, 124.4, 124.0, 121.4, 121.1, 120.5, 120.4, 110.0, 109.4.

HRMS (FD-MS) (m/z): [M^+] ($C_{55}H_{33}N_5^+$) calc. 763.2731; observed 763.2712.

3,7-Di(4-biphenyl) 1-naphthalene-10-phenoxazine

The photocatalysts was synthesized according to a literature procedure.[7-9]

1-Naphthalene-10-phenoxazine

A flame dried schlenk flask was equipped with phenoxazine (2.0 g, 10.9 mmol, 1 eq.), NaO^{t}Bu (2.1 g, 21,8 mmol, 2 eq.), RuPhos (131.2 mg, 0.32 mmol. 3 mol%), RuPhos precat (229.5 mg, 0.32 mmol, 3 mol%), 1-bromonaphthalene (3.1 ml, 21.8 mmol, 2 eq.) and 12 ml dry dioxane. The reaction mixture was stirred at 130 °C for 48 h. After cooling to room temperature DCM (20 ml) was added and the solution was washed with water (3 x 20 ml), brine (1 x 20 ml) and dried over $MgSO_4$. After removing the solvents under reduced pressure, the crude product was obtained. It was purified by recrystallization from DCM. After recrystallization, the solution was layered with hexane at –25 °C and the product was obtained as a light yellow powder.

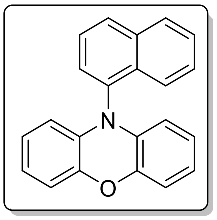

^{1}H NMR (400 MHz, $CDCl_3$, δ_H) 8.08 (d, J = 8.4 Hz, 1H), 8.02 – 7.94 (m, 2H), 7.68 – 7.60 (m, 1H), 7.59 – 7.52 (m, 2H), 7.51 – 7.44 (m, 1H), 6.78 – 6.69 (m, 2H), 6.63 (t, J = 7.6 Hz, 2H), 6.49 (dt, J = 7.7, 1.5 Hz, 2H), 5.70 (dd, J = 8.0, 1.5 Hz, 2H).

^{13}C NMR (101 MHz, $CDCl_3$, δ_C) 144.1 (C_q), 135.7 (C_q), 135.2 (C_q), 134.4 (C_q), 131.5 (C_q), 129.3 (+), 129.1 (+), 128.9 (+), 127.4 (+), 127.0 (+), 126.9 (+), 123.5 (+), 123.5 (+), 121.4 (+), 115.5 (+), 113.5 (+).

Yield: 78%

3,7-Dibromo 1-naphthalene-10-phenoxazine

In a flask which was covered in aluminum foil to block out light, 1-naphthalene-10-phenoxazine (1.6 g, 5.2 mmol, 1 eq.) was dissolved in 160 ml chloroform. 160 ml of glacial acetic acid was added to the solution. *N*-Bromosuccinimide (1.9 mg, 10.6 mmol, 2.1 eq.) was added to the stirred reaction mixture in small portions in the dark. After stirring at room temperature for 2 h, the solvents were removed under reduced pressure. The solid residue was dissolved in chloroform, washed with water (3 x 20 ml), brine (1 x 20 ml) and dried with $MgSO_4$ and the product was collected as a brown powder.

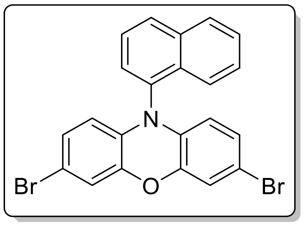

^{1}H NMR (400 MHz, Benzene-d_6, δ_H) δ 7.82 (d, *J* = 8.3 Hz, 1H), 7.57 (dd, *J* = 18.9, 8.1 Hz, 2H), 7.21 – 7.18 (m, 1H), 7.15 – 7.10 (m, 2H), 6.90 (dd, *J* = 7.3, 1.2 Hz, 1H), 6.84 (d, *J* = 2.2 Hz, 2H), 6.36 (dd, *J* = 8.5, 2.2 Hz, 2H), 5.31 (d, *J* = 8.5 Hz, 2H).

^{13}C NMR (101 MHz, Benzene-d_6, δ_C) 144.6 (C_q), 135.9 (C_q), 134.6 (C_q), 133.4 (C_q), 131.2 (C_q), 129.6 (+), 129.1 (+), 128.8 (+), 127.3 (+), 127.0 (+), 126.9 (+), 123.2 (+), 119.1 (+), 114.(+), 113.4 (+), 110.4 (C_q).

Yield: 77%

3,7-Di(4-biphenyl) 1-naphthalene-10-phenoxazine (Miyake catalyst)

In a flame dried schlenk flask 3,7-dibromo 1-naphthalene-10-phenoxazine (1.1 g, 2.2 mmol, 1 eq.) and 4-biphenylboronic acid (1.9 g, 9.7 mmol, 4 eq.) were dissolved in 90 ml THF. 27 ml of a 2 M solution of K_2CO_3 in water was added to the solution and the reaction mixture was stirred at 80 °C for 20 minutes. After that, a solution of palladium tetrakis(triphenylphosphine) (420 mg, 0.4 mmol, 15 mol%) in 90 ml THF was added and the mixture was refluxed at 100 °C for 24 h. After cooling to room temperature, the solvents were removed under reduced pressure. The solid residue was dissolved in DCM, washed with water (2 x 20 ml), brine (1 x 20 ml) and dried with $MgSO_4$. The crude product was purified by recrystallization in DCM/Methanol and the product was obtained as a light tan powder.

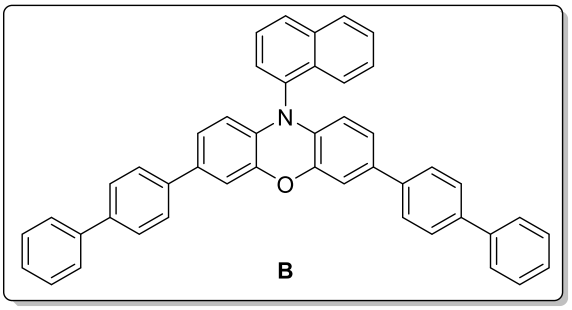

B

^{1}H NMR (400 MHz, DMSO-d_6, δ_H) 8.18 (dd, *J* = 14.5, 8.0 Hz, 2H), 8.02 (d, *J* = 8.2 Hz, 1H), 7.81 – 7.76 (m, 2H), 7.72 – 7.7 (m, 14H), 7.46 (t, *J* = 7.8 Hz, 4H), 7.38 – 7.33 (m, 2H), 7.21 (d, *J* = 2.1 Hz, 2H), 6.98 (dd, *J* = 8.4, 2.1 Hz, 2H), 5.73 (d, *J* = 8.3 Hz, 2H).

Yield: 80%

2.2. Synthesis of starting materials

N-(3-Oxobutyl)-*p*-toluenesulfonamide[10]

The substrate was synthesized according to a literature procedure.[10]

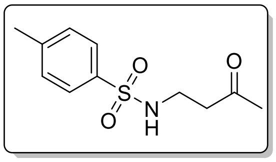

To a solution of *p*-toluenesulfonamide (1.71 g, 10.0 mmol, 1 eq.) dissolved in $CHCl_3$ (40 mL) were added Al_2O_3 (2 g, neutral) and methyl vinyl ketone (1.01 mL, 12.0 mmol, 1.2 eq.). The mixture was stirred at 45 °C in a stoppered flask for 6 days. Afterwards, the solution was filtered and the Al_2CO_3 was washed with EtOAc (30 mL). The solvent was evaporated and the crude product was purified by Automated flash column chromatography (DCM/MeOH, 2% MeOH) to afford *N*-(3-Oxobutyl)-*p*-toluenesulfonamide as white solid (600 mg, 25% yield).

^{1}H NMR (400 MHz, $CDCl_3$, δ_H) 7.63 (d, *J* = 8.3 Hz, 2H), 7.25 (d, *J* = 8.0 Hz, 2H), 5.37 (t, *J* = 6.4 Hz, 1H), 3.07 (q, *J* = 6.1 Hz, 2H), 2.62 (t, *J* = 6.0 Hz, 2H), 2.36 (s, 3H), 2.04 (s, 3H).

N-(4-Ethylphenyl)acetamide

The substrate was synthesized according to a literature procedure.[11]

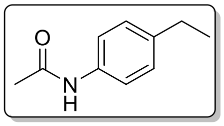

4-ethylaniline (1.21 g, 10 mmol, 1 eq) was added to a round-bottom flask. Then the flask was purged with argon and dry DCM (40 mL) was added. Acetic anhydride (1.14 mL, 12 mmol, 1.2 eq) was added and the reaction was stirred at room temperature and monitored by TLC. Upon completion, the reaction mixture was washed with a saturated solution of sodium carbonate, the organic layers dried with $MgSO_4$ and the solvent removed under reduced pressure. Purification by column chromatography (ethyl acetate/petroleum ether) afforded the product as a white solid (1.52 g, 93% yield).

^{1}H NMR (300 MHz, $CDCl_3$, δ_H) 7.53 (s, 1H), 7.43 – 7.35 (m, 2H), 7.17 – 7.08 (m, 2H), 2.60 (q, *J* = 7.7 Hz, 2H), 2.15 (s, 3H), 1.20 (t, *J* = 7.6 Hz, 3H).

^{13}C NMR (75 MHz, $CDCl_3$, δ_C) 168.5, 140.5, 135.6, 128.4, 120.3, 28.4, 24.6, 15.8.

HRMS (EI) (m/z): [M^+] $C_{10}H_{13}NO^+$: calc.: 163.0992, found: 163.0993.

Ethyl-5-phenylpentanoate (7a)[12]

The substrate was synthesized according to modified literature procedure.[13]

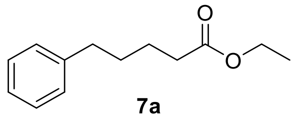

7a

Sulfuric acid (533 µL, 10.0 mmol, 1 eq.) was added to a solution of 5-phenylpentanoic acid (1.78 g, 10.0 mmol, 1 eq.) in ethanol (30 mL) cooled to 0 °C. The reaction mixture was warmed to room temperature and subsequently refluxed for 16 h. After cooling to room temperature, the solvent was evaporated. The residue was diluted with EtOAc (50 mL) and washed with NH_4Cl (aq., sat.) (3x20 mL). The organic phase was washed with brine (20 mL) and dried over $MgSO_4$. The solvent was evaporated and the crude product was purified by automated flash column chromatography (Petroleum ether/Ethyl acetate 0-20%). Ethyl-5-phenylpentanoate (1.79 g, 8.68 mmol, 87%) was obtained as colorless liquid.

^{1}H NMR (300 MHz, $CDCl_3$, δ_H) 7.31-7.24 (m, 2H), 7.21-7.14 (m, 3H), 4.12 (q, *J* = 7.1 Hz, 2H), 2.67-2.59 (m, 2H), 2.36-2.29 (m, 2H), 1.74-1.59 (m, 4H), 1.25 (t, *J* = 7.1 Hz, 3H).

^{13}C NMR (75 MHz, $CDCl_3$, δ_C) 173.8, 142.3, 128.5, 128.5, 125.9, 60.4, 35.7, 34.4, 31.1, 24.8, 14.4.

Ethyl-6-phenylhexanoate (7b)[14]

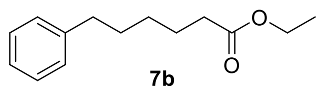

7b

The substrate was synthesized analogous to ethyl-5-phenylpentanoate (**7a**) with 6-phenylhexanoic acid (470 mg, 2.5 mmol, 1 eq.) instead of 5-phenylpentanoic acid (1.78 g, 10.0 mmol, 1 eq.) using the solvents in half of the amount noted above.

Yield: 70% (387 mg, 1.75 mmol), colorless liquid.

^{1}H NMR (300 MHz, $CDCl_3$, δ_H) 7.31-7.14 (m, 5H), 4.12 (q, *J* = 7.1 Hz, 2H), 2.65-2.57 (m, 2H), 2.29 (t, *J* = 7.5 Hz, 2H), 1.72-1.55 (m, 4H), 1.42-1.30 (m, 2H), 1.25 (t, *J* = 7.1 Hz, 3H).

^{13}C NMR (75 MHz, $CDCl_3$, δ_C) 173.9, 142.7, 128.5, 128.4, 125.8, 60.3, 35.9, 34.4, 31.3, 28.9, 25.0, 14.4.

2.3. General procedure for the photocatalytic generation of carbanions from benzylic C–H bonds

General procedure for the photocatalytic benzylation of ketones (general procedure A)

A 5 mL crimp cap vial was equipped with the photocatalyst 3DPA2FBN (3.8 mg, 6.0 µmol, 3 mol%), K_2CO_3 (2.8 mg, 20.0 µmol, 10 mol%), grinded molecular sieves (50 mg) and a stirring bar. The vessel was capped and dry acetonitrile (2 ml), the ethylbenzene derivative **1** (0.2 mmol, 1 eq.), triisopropylsilanethiol (4.3 µl, 20.0 µmol, 10 mol%) and the corresponding ketone **2** (2.0 mmol, 10 eq., unless noted otherwise) were added via syringe under a nitrogen atmosphere. The reaction mixture was degassed by three cycles of freeze pump thaw and stirred and irradiated using a blue LED (455 nm ± 15 nm) for 16 h at 25 °C. The progress could be monitored by TLC, GC analysis and GC-MS analysis. For isolation, the reaction mixture was diluted with water (10 ml), extracted with ethyl acetate (3 x 20 ml), washed with brine (1 x 20 ml) and dried over Na_2SO_4. The crude product was obtained by removing the solvents under reduced pressure. Purification was performed by automated flash column chromatography (DCM/MeOH 0-10% MeOH if not noted otherwise) yielding the corresponding product **3**.

2-Methyl-3-phenylbutan-2-ol (3a)[15]

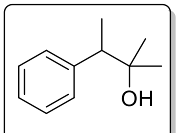

3a

^{1}H NMR (300 MHz, $CDCl_3$, δ_H) 7.37 – 7.18 (m, 5H), 2.81 (q, *J* = 7.2 Hz, 1H), 1.37 (s, 1H), 1.35 (d, *J* = 7.2 Hz, 3H), 1.19 (s, 6H).

^{13}C NMR (75 MHz, $CDCl_3$, δ_C) 143.4 (C_q), 129.1 (+), 128.2 (+), 126.6 (+), 72.8 (C_q), 50.5 (+), 28.2 (+), 27.0 (+), 15.9 (+).

HRMS (APCI) (m/z): [MNH_4^+] ($C_{11}H_{20}NO^+$) calc.: 182.1539, found: 182.1538.

Yield: with 10 eq. acetone: 41%
with acetone as co-solvent: 72%

slightly yellow liquid

2-Methyl-3-(p-tolyl)butan-2-ol (3b)

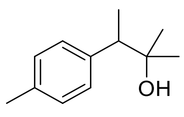

3b

^{1}H NMR (400 MHz, $CDCl_3$, δ_H) 7.19 – 7.08 (m, 4H), 2.78 (q, *J* = 7.2 Hz, 1H), 2.34 (s, 3H), 1.33 (d, *J* = 7.2 Hz, 3H), 1.19 (s, 3H), 1.18 (s, 3H).

^{13}C NMR (101 MHz, $CDCl_3$, δ_C) 140.3(C_q), 136.2 (C_q), 129.0 (+), 128.9 (+), 72.8 (C_q), 50.1 (+), 28.2 (+), 26.9 (+), 21.1 (+), 16.0 (+).

HRMS (APCI) (m/z): [MNH_4^+] ($C_{12}H_{22}NO^+$) calc.: 196.1696, found: 196.1697.

Yield: with 10 eq. acetone: 62%
with acetone as co-solvent: 55%

slightly yellow liquid

2-Methyl-3-(o-tolyl)butan-2-ol (3c)

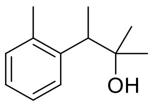

3c

^{1}H NMR (300 MHz, $CDCl_3$, δ_H) 7.38 – 7.31 (m, 1H), 7.22 – 7.09 (m, 3H), 3.17 (q, *J* = 7.1 Hz, 1H), 2.38 (s, 3H), 1.41 (s, 1H), 1.30 (d, *J* = 7.2 Hz, 3H), 1.26 (s, 3H), 1.19 (s, 3H).

^{13}C NMR (75 MHz, $CDCl_3$, δ_C) 142.3 (C_q), 136.9 (C_q), 130.5 (+), 127.5 (+), 126.2 (+), 126.0 (+), 73.6 (C_q), 43.9 (+), 28.5 (+), 27.3 (+), 20.8 (+), 16.6 (+).

HRMS (APCI) (m/z): [MNH_4^+] ($C_{12}H_{22}NO^+$) calc.: 196.1696, found: 196.1699.

Yield: with 10 eq. acetone: 22%
with acetone as co-solvent: 44%

slightly yellow liquid

2,3-Dimethyl-3-phenylbutan-2-ol (3d)[16]

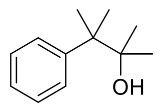

3d

^{1}H NMR (400 MHz, $CDCl_3$, δ_H) 7.48 – 7.44 (m, 2H), 7.35 – 7.29 (m, 2H), 7.25 – 7.19 (m, 1H), 1.43 (s, 6H), 1.34 (s, 1H), 1.15 (s, 6H).

^{13}C NMR (101 MHz, $CDCl_3$, δ_C) 146.4 (C_q), 128.2 (+), 127.8 (+), 126.1 (+), 74.6 (C_q), 45.2 (C_q), 25.8 (+), 24.5 (+).

HRMS (APCI) (m/z): [MNH_4^+] ($C_{12}H_{22}NO^+$) calc.: 196.1696, found: 196.1696.

Yield: with 10 eq. acetone: 11%
with acetone as co-solvent: 29%

slightly yellow liquid

2,5-Dimethyl-3-phenylhexan-2-ol (3e)[17]

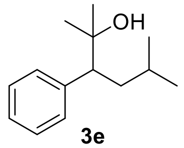

3e

^{1}H NMR (400 MHz, $CDCl_3$, δ_H) 7.35 – 7.26 (m, 2H), 7.26 – 7.20 (m, 3H), 2.69 (dd, J = 12.3, 3.2 Hz, 1H), 1.85 (ddd, J = 13.4, 12.4, 3.4 Hz, 1H), 1.49 (ddd, J = 13.6, 10.5, 3.2 Hz, 1H), 1.35 (s, 1H), 1.27 – 1.20 (m, 1H), 1.18 (d, J = 1.9 Hz, 6H), 0.85 (d, J = 6.7 Hz, 3H), 0.81 (d, J = 6.5 Hz, 3H).

^{13}C NMR (101 MHz, $CDCl_3$, δ_C) 141.4 (C_q), 129.7 (+), 128.2 (+), 126.6 (+), 72.8 (C_q), 54.9 (+), 38.7 (–), 28.3 (+), 27.8 (+), 25.8 (+), 24.4 (+), 21.1 (+).

HRMS (APCI) (m/z): [MNH_4^+] ($C_{14}H_{26}NO^+$) calc.: 224.2009, found: 224.2009.

Yield: with 10 eq. acetone: 47%
with acetone as co-solvent: 79%

slightly yellow liquid

3-(4-Methoxyphenyl)-2-methylbutan-2-ol (3f)

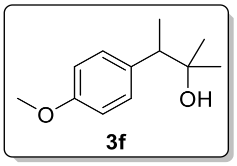

^{1}H NMR (300 MHz, $CDCl_3$, δ_H) 7.20 – 7.13 (m, 2H), 6.88 – 6.81 (m, 2H), 3.79 (s, 3H), 2.76 (q, *J* = 7.2 Hz, 1H), 1.69 (s, 1H), 1.31 (d, *J* = 7.2 Hz, 3H), 1.17 (s, 3H), 1.16 (s, 3H).

^{13}C NMR (75 MHz, $CDCl_3$, δ_C) 158.3 (C_q), 135.4 (C_q), 129.9 (+), 113.5 (+), 72.8 (C_q), 55.3 (+), 49.6 (+), 28.0 (+), 26.9 (+), 16.0 (+).

HRMS (APCI) (m/z): [MNH_4^+] ($C_{12}H_{22}NO_2^+$) calc.: 212.1645, found: 212.1648.

Yield: with 10 eq. acetone: 67%
with acetone as co-solvent: 87%

slightly yellow liquid

3-(2-Methoxyphenyl)-2-methylbutan-2-ol (3g)

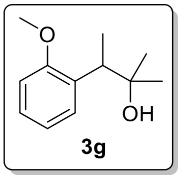

^{1}H NMR (300 MHz, $CDCl_3$, δ_H) 7.26 – 7.18 (m, 2H), 6.99 – 6.86 (m, 2H), 3.83 (s, 3H), 3.37 (q, *J* = 7.3 Hz, 1H), 2.46 (s, 1H), 1.30 (d, *J* = 7.3 Hz, 3H), 1.19 (s, 3H) 1.17 (s, 3H).

^{13}C NMR (75 MHz, $CDCl_3$, δ_C) 157.1 (C_q), 132.3 (C_q), 129.3 (+), 127.3 (+), 120.8 (+), 110.7 (+), 73.4 (C_q), 55.5 (+), 29.2 (+), 26.4 (+), 15.6 (+).

HRMS (APCI) (m/z): [MNH_4^+] ($C_{12}H_{22}NO_2^+$) calc.: 212.1645, found: 212.1641.

Yield: with 10 eq. acetone: 48%
with acetone as co-solvent: 83%

yellow liquid

3-(4-Methoxyphenyl)-2,3-dimethylbutan-2-ol (3h)[18]

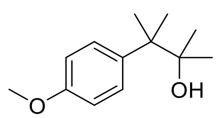

3h

^{1}H NMR (400 MHz, $CDCl_3$, δ_H) 7.39 – 7.34 (m, 2H), 6.88 – 6.83 (m, 2H), 3.80 (s, 3H), 1.40 (s, 6H), 1.27 (s, 1H), 1.14 (s, 6H).

^{13}C NMR (101 MHz, $CDCl_3$, δ_C) 157.8 (C_q), 138.4 (C_q), 129.2 (+), 113.1 (+), 74.7 (C_q), 55.3 (+), 44.6 (C_q), 25.8 (+), 24.6 (+).

HRMS (APCI) (m/z): [MNH_4^+] ($C_{13}H_{24}NO_2^+$) calc.: 226.1802, found: 226.1804.

Yield: with 10 eq. acetone: 52%
with acetone as co-solvent: 77%

slightly yellow liquid

1-(4-Methoxyphenyl)-2-methylpropan-2-ol (3i)[19]

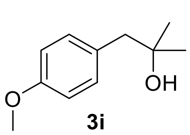

3i

^{1}H NMR (300 MHz, $CDCl_3$, δ_H) 7.16 – 7.10 (m, 2H), 6.89 – 6.82 (m, 2H), 3.80 (s, 3H), 2.71 (s, 2H), 1.44 (s, 1H), 1.21 (s, 6H).

^{13}C NMR (75 MHz, $CDCl_3$, δ_C)158.5 (C_q), 131.5 (+), 129.9 (C_q), 113.8 (+), 70.9 (C_q), 55.4 (+), 48.9 (–), 29.2 (+).

HRMS (EI+) (m/z): [$M^{\bullet+}$] ($C_{11}H_{16}O_2^{\bullet+}$) calc.: 180.1145, found: 180.1154.

Yield: with 10 eq. acetone: 19%
with acetone as co-solvent: 53%

slightly yellow liquid

N-(4-(3-Hydroxy-3-methylbutan-2-yl)phenyl)acetamide (3j)

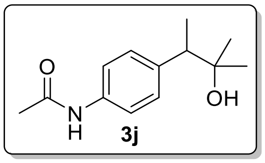

^{1}H NMR (300 MHz, $CDCl_3$, δ_H) 8.24 (s, 1H), 7.44 – 7.38 (m, 2H), 7.17 – 7.09 (m, 2H), 2.73 (q, *J* = 7.2 Hz, 1H), 2.11 (s, 3H), 2.00 (s, 1H), 1.27 (d, *J* = 7.2 Hz, 3H), 1.13 (s, 6H).

^{13}C NMR (75 MHz, $CDCl_3$, δ_C) 169.1 (C_q), 139.4 (C_q), 136.5 (C_q), 129.4 (+), 119.9 (+), 72.8 (C_q), 49.9 (+), 28.1 (+), 26.9 (+), 24.4 (+), 15.9 (+).

HRMS (APCI) (m/z): [MNH_4^+] ($C_{13}H_{23}N_2O_2^+$) calc.: 239.1754, found: 239.1756.

Yield: with 10 eq. acetone: 57%
with acetone as co-solvent: 87%

white solid

3-(4-Chlorophenyl)-2-methylbutan-2-ol (3k)

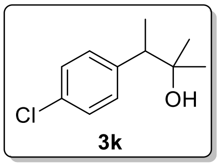

^{1}H NMR (300 MHz, $CDCl_3$, δ_H) 7.31 – 7.15 (m, 4H), 2.77 (q, *J* = 7.2 Hz, 1H), 1.35 (s, 1H), 1.31 (d, *J* = 7.2 Hz, 3H), 1.18 (s, 3H), 1.16 (s, 3H).

^{13}C NMR (75 MHz, $CDCl_3$, δ_C) 142.1 (C_q), 132.3 (C_q), 130.4 (+), 128.2 (+), 72.7 (C_q), 49.9 (+), 28.2 (+), 27.2 (+), 15.9 (+).

HRMS (APCI) (m/z): [MNH_4^+] ($C_{11}H_{19}ClNO^+$) calc.: 216.1150, found: 216.1150.

Yield: with 10 eq. acetone: 32%
with acetone as co-solvent: 53%

slightly yellow liquid

3-(4-Fluorophenyl)-2-methylbutan-2-ol (3l)

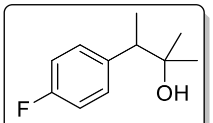

3l

^{1}H NMR (300 MHz, $CDCl_3$, δ_H) 7.25 – 7.17 (m, 2H), 7.04 – 6.93 (m, 2H), 2.78 (q, J = 7.2 Hz, 1H), 1.31 (d, J = 7.3 Hz, 3H), 1.17 (s, 3H), 1.16 (s, 3H).

^{13}C NMR (75 MHz, $CDCl_3$, δ_C) 161.7 (d, $^{1}J_{CF}$ = 244.3 Hz, C_q), 139.2 (d, $^{4}J_{CF}$ = 3.3 Hz, C_q), 130.4 (d, $^{3}J_{CF}$ = 7.7 Hz, +), 114.9 (d, $^{2}J_{CF}$ = 20.9 Hz, +), 72.7 (C_q), 49.7 (+) , 28.2 (+) , 27.1 (+) , 16.1 (+) .

^{19}F NMR (282 MHz, $CDCl_3$, δ_F) -117.4.

HRMS (APCI) (m/z): [MNH_4^+] ($C_{11}H_{19}FNO^+$) calc.: 200.1445, found: 200.1444.

Yield: with 10 eq. acetone: 59%
with acetone as co-solvent: 78%
slightly yellow liquid

3,3',3''-(Benzene-1,3,5-triyl)tris(2-methylbutan-2-ol) (3m)

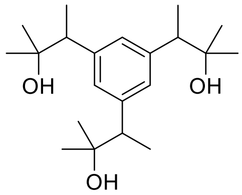

3m

^{1}H NMR (300 MHz, $CDCl_3$, δ_H) 6.97 (s, 3H), 2.80 – 2.71 (m, 3H), 1.93 (s, 3H), 1.31 (d, J = 7.2 Hz, 9H), 1.16 – 1.10 (m, 18H).

^{13}C NMR (75 MHz, $CDCl_3$, δ_C) 142.4 (C_q), 142.4 (C_q), 128.2 (+), 128.1 (+), 127.8 (+), 72.9 (C_q), 50.4 (+), 50.4 (+), 28.4 (+), 26.7 (+), 15.9 (+).

HRMS (APCI) (m/z): [MNH_4^+] ($C_{21}H_{40}NO_3^+$) calc.: 354.3003, found: 354.3003.

Yield: with 10 eq. acetone:
with acetone as co-solvent: 87%
yellow liquid

3-([1,1'-Biphenyl]-4-yl)-2-methylbutan-2-ol (3n)

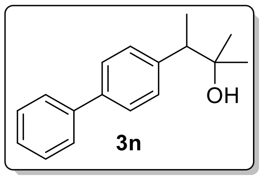

3n

^{1}H NMR (400 MHz, $CDCl_3$, δ_H) 7.65 – 7.59 (m, 2H), 7.59 – 7.54 (m, 2H), 7.49 – 7.42 (m, 2H), 7.38 – 7.32 (m, 3H), 2.88 (q, *J* = 7.2 Hz, 1H), 1.50 (s, 1H), 1.40 (d, *J* = 7.2 Hz, 3H), 1.24 (s, 6H).

^{13}C NMR (101 MHz, $CDCl_3$, δ_C) 142.6 (C_q), 141.0 (C_q), 139.5 (C_q), 129.5 (+), 128.9 (+), 127.2 (+), 127.1 (+), 126.8 (+), 72.8 (C_q), 50.2 (+), 28.3 (+), 27.1 (+), 15.9 (+).

HRMS (EI+) (m/z): [$M^{\bullet+}$] ($C_{17}H_{20}O^{\bullet+}$) calc.: 240.1509, found: 240.1517.

Yield: with 10 eq. acetone: 31%
with acetone as co-solvent: 62%

yellowish solid

2-Methyl-3-(naphthalen-2-yl)butan-2-ol (3o)[20]

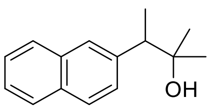

3o

^{1}H NMR (400 MHz, $CDCl_3$, δ_H) 7.85 – 7.77 (m, 3H), 7.70 (s, 1H), 7.50 – 7.40 (m, 3H), 2.99 (q, *J* = 7.2 Hz, 1H), 1.45 (d, *J* = 7.3 Hz, 3H), 1.27 (s, 1H), 1.24 (s, 3H), 1.23 (s, 3H).

^{13}C NMR (101 MHz, $CDCl_3$, δ_C) 141.1 (C_q), 133.4 (C_q), 132.5 (C_q), 127.9 (+), 127.7 (+), 127.7 (+), 127.6 (+), 127.5 (+), 126.1 (+), 125.6 (+), 73.0 (C_q), 50.6 (+), 28.4 (+), 27.2 (+), 16.1 (+).

HRMS (APCI) (m/z): [MNH_4^+] ($C_{15}H_{22}NO^+$) calc.: 232.1696, found: 232.1697.

Yield: with 10 eq. acetone: 7%
with acetone as co-solvent: 34%

slightly yellow liquid

2-Methyl-3-(thiophen-2-yl)butan-2-ol (3p)

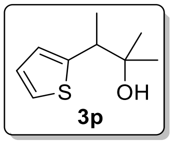

^{1}H NMR (400 MHz, $CDCl_3$, δ_H) 7.20 – 7.15 (m, 1H), 6.99 – 6.94 (m, 1H), 6.89 – 6.87 (m, 1H), 3.12 (q, J = 7.2 Hz, 1H), 1.59 (s, 1H), 1.38 (d, J = 7.2 Hz, 3H), 1.24 (s, 3H), 1.21 (s, 3H).

^{13}C NMR (101 MHz, $CDCl_3$, δ_C) 146.6 (C_q), 126.5 (+), 125.4 (+), 123.7 (+), 72.3 (C_q), 46.6 (+), 28.1 (+), 26.5 (+), 17.8/ (+).

HRMS (APCI) (m/z): [M+H$^+$] ($C_9H_{15}OS^+$) calc.: 171.0838, found: 171.0836.

Yield: with 10 eq. acetone: 43%
with acetone as co-solvent: 73%

slightly yellow liquid

3-(Benzofuran-2-yl)-2-methylbutan-2-ol (3q)

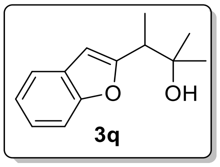

^{1}H NMR (400 MHz, $CDCl_3$, δ_H) 7.55 – 7.43 (m, 2H), 7.27 – 7.19 (m, 2H), 6.50 (s, 1H), 3.04 (q, J = 7.2 Hz, 1H), 2.01 (s, 1H), 1.40 (d, J = 7.2 Hz, 3H), 1.28 (s, 3H), 1.24 (s, 3H).

^{13}C NMR (101 MHz, $CDCl_3$, δ_C) 160.9 (C_q), 154.6 (C_q), 128.5 (C_q), 123.6 (+), 122.8 (+), 120.6 (+), 111.1 (+), 103.6 (+), 72.8 (C_q), 45.0 (+), 28.0 (+), 26.9 (+), 14.3 (+).

HRMS (EI+) (m/z): [M$^{\bullet+}$] ($C_{13}H_{16}O_2^{\bullet+}$) calc.: 204.1145, found: 204.1147.

Yield: with 10 eq. acetone: 14%
with acetone as co-solvent: 51%

colorless liquid

2-(4-Methoxyphenyl)-3-methylpentan-3-ol (3r)

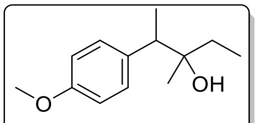

3r

Column chromatography: First column: DCM/Methanol 0-5%. Second column: *n*-pentane/EtOAc 0-35%.

^{1}H-NMR (diastereomeric mixture) (300 MHz, $CDCl_3$, δ_H): 7.21-7.14 (m, 2H), 6.88-6.82 (m, 2H), 3.79 (s, 3H), 2.86-2.74 (m, 1H), 1.61-1.38 (m, 2H), 1.30 [1.28] (d, *J* = 3.3 Hz, 3H), 1.20 (s, 1H), 1.10 [1.04] (s, 3H), 0.94 [0.92] (t, *J* = 7.4 Hz, 3H).

^{13}C-NMR (diastereomeric mixture) (75 MHz, $CDCl_3$, δ_C): 158.3 (C_q), 158.2 (C_q), 135.6 (C_q), 135.2 (C_q), 130.1 (+), 130.0 (+), 113.5 (+), 113.5 (+), 74.6 (C_q), 74.4 (C_q), 55.3 (+), 47.8 (+), 47.1 (+), 33.0 (–), 31.8 (–), 24.5 (+), 23.3 (+), 16.0 (+), 15.6 (+), 8.3 (+), 8.1 (+).

HRMS (APCI) (m/z): [MNH_4^+] ($C_{13}H_{24}NO_2^+$) calc.: 226.1802, found: 226.1805.

Yield: 58% (slightly yellow liquid).

3-Ethyl-2-(4-methoxyphenyl)pentan-3-ol (3s)

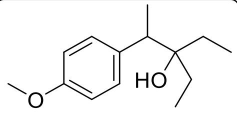

3s

Column chromatography: *n*-Pentane/EtOAc 0-20%.

^{1}H-NMR (300 MHz, $CDCl_3$, δ_H): 7.20-7.15 (m, 2H), 6.87-6.82 (m, 2H), 3.80 (s, 3H), 2.83 (q, *J* = 7.2 Hz, 1H), 1.56 (q, *J* = 7.5 MHz, 2H), 1.42-1.19 (m, 5H), 1.01 (bs, 1H), 0.91-0.80 (m, 6H).

^{13}C-NMR (75 MHz, $CDCl_3$, δ_C): 158.2 (C_q), 135.7 (C_q), 130.1 (+), 113.6 (+), 76.1 (C_q), 55.4 (+), 44.7 (+), 29.2 (–), 27.4 (–), 15.6 (+), 8.1 (+), 7.8 (+).

HRMS (APCI) (m/z): [MNH_4^+] ($C_{14}H_{26}NO_2^+$) calc.: 240.1958, found: 240.1961.

Yield: 34% (slightly yellow liquid).

5-(1-(4-Methoxyphenyl)ethyl)nonan-5-ol (3t)

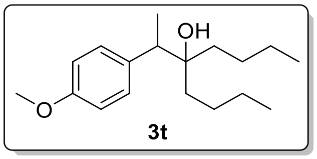

Column chromatography: *n*-Pentane/EtOAc 0-20%.

^{1}H-NMR (300 MHz, $CDCl_3$, δ_H): 7.19-7.13 (m, 2H), 6.87-6.82 (m, 2H), 3.80 (s, 3H), 2.81 (q, *J* = 7.2 Hz, 1H), 1.59-1.01 (m, 16H), 0.96-0.82 (m, 6H).

^{13}C-NMR (75 MHz, $CDCl_3$, δ_C): 158.2 (C_q), 135.7 (C_q), 130.2 (+), 113.6 (+), 75.8 (C_q), 55.4 (+), 45.4 (+), 37.3 (–), 35.4 (–), 26.0 (–), 25.8 (–), 23.6 (–), 23.4 (–), 15.6 (+), 14.3 (+), 14.3 (+).

HRMS (APCI) (m/z): [MNH_4^+] ($C_{18}H_{34}NO_2^+$) calc.: 296.2584, found: 296.2586.

Yield: 10% (colorless liquid).

2-(4-Methoxyphenyl)-3,4-dimethylpentan-3-ol (3u)

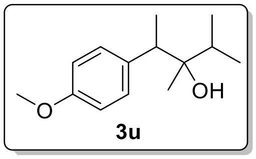

Column chromatography: *n*-Pentane/EtOAC 0-20%.

^{1}H-NMR (diastereomeric mixture) (300 MHz, $CDCl_3$, δ_H): 7.23-7.14 (m, 2H), 6.87-6.82 (m, 2H), 3.80 (s, 3H), 2.97 [2.85] (q, *J* = 7.2 Hz, 1H), 2.02-1.92 [16.2-1.53] (m, 1H), 1.27 [1.26] (d, *J* = 7.2 Hz, 3H), 1.12 (s, 1H), 1.06 [0.83] (s, 3H), 1.01-0.90 (m, 6H).

^{13}C-NMR (diastereomeric mixture) (75 MHz, $CDCl_3$, δ_C): 158.2 (C_q), 158.2 (C_q), 136.5 (C_q), 135.7 (C_q), 130.1 (+), 113.6 (+),113.5 (+), 76.2 (C_q), 76.1 (C_q), 55.3 (+), 45.3 (+), 44.5 (+), 34.6 (+), 33.7 (+), 20.4 (+), 19.4 (+), 18.2 (+), 18.0 (+), 17.3 (+), 16.8 (+), 16.3 (+), 15.0 (+).

HRMS (APCI) (m/z): [MNH_4^+] ($C_{14}H_{26}NO_2^+$) calc.: 240.1958, found: 240.1960.

Yield: 31% (colorless liquid).

2-Cyclopropyl-3-(4-methoxyphenyl)butan-2-ol (3v)

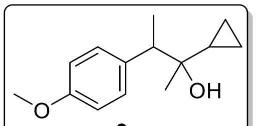

3v

Column chromatography: DCM (for 1.5 CV) followed by *n*-hexane/EtOAc 0-20% gradient.

^{1}H-NMR (diastereomeric mixture) (300 MHz, $CDCl_3$, δ_H): 7.25 – 7.15 (m, 2H), 6.88 – 6.78 (m, 2H), 3.80 (s, 3H), 2.93 – 2.77 (m, 1H), 1.38 [1,37] (d, *J* = 7.3 Hz, 2H), 1.06 [1.01] (s, 3H), 0.94 – 0.79 (m, 1H), 0.41 – 0.17 (m, 4H).

^{13}C-NMR (diastereomeric mixture) (75 MHz, $CDCl_3$ δ_C): 158.3 (C_q), 135.5 (C_q), 130.2 (+), 113.4 (+), 72.9 (C_q), 72.7 (C_q), 55.3 (+), 50.1 (+), 49.8 (+), 24.9 (+), 23.1 (+), 19.9 (+), 19.0 (+), 16.0 (+), 1.4 (–), 1.3 (–), 0.9 (–), 0.6 (–).

HRMS (APCI) (m/z): [MNH_4^+] ($C_{14}H_{24}NO_2^+$) calc.: 238.1802, found: 238.1798.

Yield: 31% (slightly yellow liquid).

1-(1-(4-Methoxyphenyl)ethyl)cyclopentan-1-ol (3w)

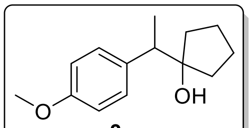

3w

Column chromatography: DCM (for 1.5 CV) followed by *n*-hexane/EtOAc 0-25% gradient.

^{1}H-NMR (400 MHz, $CDCl_3$, δ_H): 7.22-7.18 (m, 2H), 6.87-6.83 (m, 2H), 3.80 (s, 3H), 2.78 (q, *J* = 7.1 Hz, 1H), 1.89-1.55 (m, 7H), 1.34 (d, *J* = 7.1 Hz, 3H), 1.27-1.21 (m, 1H), 1.05 (bs, 1H).

^{13}C-NMR (101 MHz, $CDCl_3$, δ_C): 158.3 (C_q), 136.3 (C_q), 129.5 (+), 113.7 (+), 84.8 (C_q), 55.4 (+), 48.0 (+), 39.7 (–), 37.9 (–), 23.9 (–), 23.7 (–), 16.4 (+).

HRMS (EI) (m/z): [M^+] ($C_{14}H_{20}O_2^+$) calc.: 220.1458, found: 220.1448.

Yield: 57% (slightly yellow liquid).

1-(1-(4-Methoxyphenyl)ethyl)cyclobutan-1-ol (3x)

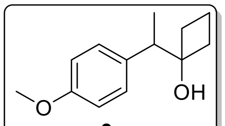

3x

Ketone equivalents: 3 eq. cyclobutanone were used for the reaction.

Column chromatography: DCM (for 1.5 CV) followed by *n*-pentane/EtOAc 0-20% gradient.

^{1}**H-NMR** (400 MHz, $CDCl_3$, δ_H): 7.24-7.19 (m, 2H), 6.88-6.83 (m, 2H), 3.80 (s, 3H), 2.88 (q, *J* = 7.1 Hz, 1H), 2.27-2.09 (m, 2H), 2.05-1.96 (m, 1H), 1.91-1.71 (m, 2H), 1.63-1.53 (m, 2H), 1.29 (d, *J* = 7.1 Hz, 3H).

^{13}**C-NMR** (101 MHz, $CDCl_3$, δ_C): 158.4 (C_q), 134.7 (C_q), 129.6 (+), 113.8 (+), 78.2 (C_q), 55.4 (+), 46.4 (+), 34.6 (–), 34.2 (–), 14.6 (+), 12.6 (–).

HRMS (EI) (m/z): [M^+] ($C_{13}H_{18}O_2^+$) calc.: 206.1301, found: 206.1299.

Yield: 69% (colorless liquid).

2-(4-Methoxyphenyl)-3-methylhept-6-en-3-ol (3y)

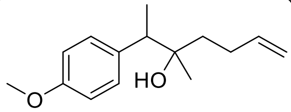

3y

Ketone equivalents: 3 eq. 5-hexen-2-one were used for the reaction.

Column chromatography: DCM (for 1.5 CV) followed by *n*-pentane/EtOAc 0-20% gradient.

^{1}**H-NMR** (diastereomeric mixture) (400 MHz, $CDCl_3$, δ_H): 7.20-7.14 (m, 2H), 6.88-6.82 (m, 2H), 5.88-5.77 (m, 1H), 5.06-4.91 (m, 2H), 3.80 (s, 3H), 2.86-2.74 (m, 1H), 2.23-2.04 (m, 2H), 1.62-1.46 (m, 2H), 1.31 [1.29] (d, *J* = 7.2 Hz, 3H), 1.27-1.21 (m, 1H), 1.12 [1.10] (s, 3H).

^{13}**C-NMR** (diastereomeric mixture) (101 MHz, $CDCl_3$, δ_C): 158.4 (C_q), 158.4 (C_q), 139.3 (+), 139.2 (+), 135.3 (C_q), 134.9 (C_q), 130.2 (+), 130.1 (+), 114.5 (–), 114.4 (–), 113.6 (+), 113.6 (+), 74.4 (C_q), 74.3 (C_q), 55.4 (+), 48.7 (+), 47.8 (+), 39.6 (–), 38.4 (–), 28.4 (–), 28.3 (–), 24.9 (+), 23.7 (+), 16.0 (+), 15.7 (+).

HRMS (APCI) (m/z): [MNH_4^+] ($C_{15}H_{26}NO_2^+$) calc.: 252.1958, found: 252.1958.

Yield: 24% (colorless liquid).

7-Chloro-2-(4-methoxyphenyl)-3-methylheptan-3-ol (3z)

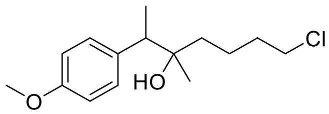

3z

Ketone equivalents: 3 eq. 6-chloro-2-hexanone were used for the reaction.

Column chromatography: DCM (for 1.5 CV) followed by *n*-pentane/EtOAc 0-20% gradient.

^{1}H-NMR (diastereomeric mixture) (300 MHz, $CDCl_3$, δ_H): 7.20-7.13 (m, 2H), 6.88-6.82 (m, 2H), 3.80 [3.80] (s, 3H), 3.54 [3.53] (t, J = 6.6 Hz, 2H), 2.86-2.74 (m, 1H), 1.82-1.37 (m, 6H), 1.29 [1.28] (d, J = 7.2 Hz, 3H), 1.20 (s, 1H), 1.11 [1.08] (s, 3H).

^{13}C-NMR (diastereomeric mixture) (75 MHz, $CDCl_3$, δ_C): 158.4 (C_q), 158.3 (C_q), 135.3 (C_q), 134.8 (C_q), 130.2 (+), 130.0 (+), 113.6 (+), 113.6 (+), 74.3 (C_q), 74.2 (C_q), 55.4 (+), 48.3 (+), 47.5 (+), 45.2 (–), 45.2 (–), 39.8 (–), 38.5 (–), 33.3 (–), 33.2 (–), 25.1 (+), 23.7 (+), 21.4 (–), 21.2 (–), 16.0 (+), 15.6 (+).

HRMS (APCI) (m/z): [MNH_4^+] ($C_{15}H_{27}ClNO_2^+$) calc.: 288.1725, found: 288.1728.

Yield: 30% (colorless liquid).

3-(1-(4-Methoxyphenyl)ethyl)tetrahydrofuran-3-ol (3aa)

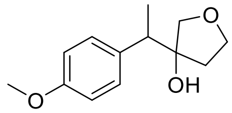

3aa

Ketone equivalents: 1 eq. tetrahydrofuran-3-one was used for the reaction.

Column chromatography: First column: DCM/MeOH (98:2). Second column: *n*-hexane/EtOAc 35-50%.

^{1}H-NMR (diastereomeric mixture) (300 MHz, $CDCl_3$, δ_H): 7.24-7.17 (m, 2H), 6.89-6.82 (m, 2H), 4.08-3.85 (m, 2H), 3.79 [3.79] (s, 3H), 3.77-3.65 + 3.30-3.25 (m, 2H), 2.87 [2.87] (q, J = 7.1 Hz, 1H), 2.05-1.91 + 1.53-1.44 (m, 2H), 1.73 [1.68] (bs, 1H), 1.38 [1.33] (d, J = 7.2 Hz, 3H).

^{13}C-NMR (diastereomeric mixture) (101 MHz, $CDCl_3$, δ_C): 158.6 (C_q), 158.6 (C_q), 135.1 (C_q), 135.1 (C_q), 129.4 (+), 129.1 (+), 113.9 (+), 113.9 (+), 83.6 (C_q), 79.0 (–), 78.0 (–), 68.1 (–), 67.8 (–), 55.4 (+), 55.4 (+), 45.8 (+), 45.6 (+), 40.0 (–), 38.7 (–), 16.5 (+), 16.4 (+).

HRMS (APCI) (m/z): [MHN_4^+] ($C_{13}H_{22}NO_3$) calc.: 240.1594, found: 240.1596.

Yield: 30% (slightly yellow solid).

Methyl 2-hydoxy-2-(1-(4-methoxyphenyl)ethyl)cyclopentane-1-carboxylate (3ab)

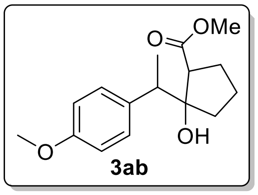

Ketone equivalents: 1 eq. methyl 2-oxocyclopentanecarboxylate was used for the reaction.

Column chromatography: First column: DCM (for 1.5 CV) followed by *n*-pentane/EtOAc 0-20% gradient. Second column: DCM/EtOAc 10%.

^{1}**H-NMR** (diastereomeric mixture) (300 MHz, $CDCl_3$, δ_H): 7.19-7.12 (m, 2H), 6.84-6.77 (m, 2H), 4.17 [3.88] (s, 1H), 3.78 [3.78] (s, 3H), 3.72 [3.27] (s, 3H), 2.95 [2.71] (q, J = 7.2 Hz, 1H), 2.61-2.43 (m, 1H), 2.06-1.56 (m, 6H), 1.34 [1.33] (q, J = 7.1 Hz, 3H).

^{13}**C-NMR** (diastereomeric mixture) (75 MHz, $CDCl_3$, δ_C): 177.2 (C_q), 177.0 (C_q), 158.4 (C_q), 158.3 (C_q), 135.8 (C_q), 135.5 (C_q), 129.8 (+), 129.6 (+), 113.5 (+), 113.4 (+), 85.3 (C_q), 85.1 (C_q), 55.4 (+), 55.3 (+), 51.9 (+), 51.5(+), 50.6 (+), 49.6 (+), 48.5 (+), 47.1 (+), 38.7 (–), 35.4 (–), 30.2 (–), 29.1 (–), 22.0 (–), 21.6 (–), 16.8 (+), 16.4 (+).

HRMS (APCI) (m/z): [MNH_4^+] ($C_{16}H_{26}NO_4$) calc.: 296.1856, found: 296.1857.

Yield: 27% (colorless liquid).

***N*-(3-hydroxy-4-(4-methoxyphenyl)-3-methylpentyl)-4-methylbenzenesulfonamide (3ac)**

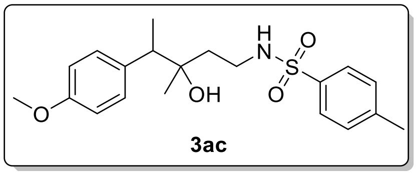

Ketone equivalents: 2 eq. *N*-(3-oxobutyl)-*p*-toluenesulfonamide were used for the reaction.

Column chromatography: DCM/MeOH (98:2).

^{1}**H-NMR** (diastereomeric mixture) (300 MHz, $CDCl_3$, δ_H): 7.76-7.70 (m, 2H), 7.32-7.26 (m, 2H), 7.09-7.02 (m, 2H), 6.86-6.79 (m, 2H), 5.63-5.40 (m, 1H), 3.79 [3.78] (s, 3H), 3.20-2.97 (m, 2H), 2.74-2.62 (m, 1H), 2.41 (s, 3H), 1.74-1.44 (m, 3H), 1.23 [1.19] (d, J = 7.2 Hz, 3H), 1.00 [0.97] (s, 3H).

^{13}**C-NMR** (diastereomeric mixture) (75 MHz, $CDCl_3$, δ_C): 158.7 (C_q), 158.6 (C_q), 143.3 (C_q), 143.3 (C_q), 137.0 (C_q), 137.0 (C_q), 134.2 (C_q), 133.4 (C_q), 130.2 (+), 129.9 (+), 129.7 (+), 129.7 (+), 127.2 (+), 127.2 (+), 113.8 (+), 113.8 (+), 75.3 (C_q), 75.1 (C_q), 55.4 (+), 49.6 (+), 48.5 (+), 39.6 (–), 39.6 (–), 37.5 (–), 36.5 (–), 23.9 (+), 23.0 (+), 21.6 (+), 15.8 (+), 15.4 (+).

HRMS (ESI) (m/z): [MH^+] ($C_{20}H_{28}NO_4S^+$) calc.: 378.1734, found: 378.1735.

Yield: 21% (yellow solid).

3-(1-(4-Methoxyphenyl)ethyl)cyclopentan-1-one (3ad)

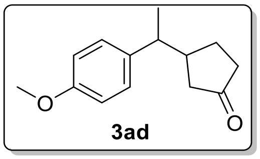

Ketone equivalents: 1 eq. 2-cyclopentene-1-one was used for the reaction.

Column chromatography: First column: DCM/MeOH 0-5%. Second column: *n*-pentane/EtOAc 0-40%.

^{1}H-NMR (diastereomeric mixture) (400 MHz, $CDCl_3$, δ_H): 7.12-7.05 (m, 2H), 6.88-6.81 (m, 2H), 3.80 [3.78] (s, 3H), 2.59-1.88 (m, 6H), 1.81-1.70 (m, 1H), 1.64-1.33 (m, 1H), 1.30 [1.27] (d, J = 6.9 Hz, 3H).

^{13}C-NMR (diastereomeric mixture) (101 MHz, $CDCl_3$, δ_C): 219.5 (C_q), 219.2 (C_q), 158.2 (C_q), 158.2 (C_q), 138.0 (C_q), 137.6 (C_q), 128.2 (+), 128.1 (+), 114.0 (+), 113.9 (+), 55.4 (+), 45.1 (+), 44.9 (+), 44.7 (+), 44.5 (+), 44.4 (–), 44.4 (–), 39.1 (–), 39.0 (–), 28.5 (–), 28.4 (–), 21.2 (+), 20.2 (+).

HRMS (EI) (m/z): [M^+] ($C_{14}H_{18}O_2^+$) calc.: 218.1301, found: 218.1305.

Yield: 36% (colorless liquid).

General procedure for the photocatalytic benzylation of aldehydes (general procedure B)

A 5 mL crimp cap vial equipped with a magnetic stirring bar was loaded with 3DPAFIPN (4.9 mg, 7.50 µmol, 3 mol%), K_2CO_3 (10.4 mg, 75.0 µmol, 50 mol%), $(^{i}Pr)_3SiSH$ (4.3 µL, 20 µmol, 10 mol%), the corresponding ethyl benzene derivative (450 µmol, 3 eq.), the corresponding aldehyde (150 µmol, 1 eq.) and dry MeCN. In doing so, all solid compounds were added before capping the vial, whereas all liquid compounds were added *via* syringe after setting the capped vial under inert conditions. The reaction mixture was degassed by three cycles of freeze-pump-thaw and subsequently stirred under light irradiation using a 455 nm (±15 nm) LED for 16 h at 25 °C. Two reaction batches were combined and diluted with brine (10 mL), water (10 mL) and ethyl acetate (15 mL). The phases were separated, and the water phase was extracted with ethyl acetate (3 x 7 mL). The combined organic phases were washed with brine (10 mL) and dried over Na_2SO_4. The solvent was removed under reduced pressure and the crude product was purified by automated flash column chromatography (n-Pentane/Ethyl acetate, 0-20% if not noticed otherwise).

2-(4-Methoxyphenyl)heptan-3-ol (6a)

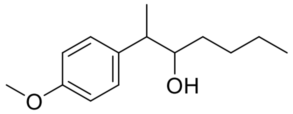

6a

^{1}H-NMR (diastereomeric mixture) (400 MHz, $CDCl_3$, δ_H): 7.18-7.11 (m, 2H), 6.89-6.84 (m, 2H), 3.80 (s, 3H), 3.64-3.58 (m, 1H), 2.78-2.67 (m, 1H), 1.60-1.23 (m, 10H), 0.91 [0.87] (t, *J* = 7.1 Hz, 3H).

^{13}C-NMR (diastereomeric mixture) (101 MHz, $CDCl_3$, δ_C): 158.4 (C_q), 158.2 (C_q), 136.8 (C_q), 135.5 (C_q), 129.2 (+), 128.8 (+), 114.1 (+), 114.0 (+), 76.5 (+), 76.3 (+), 55.4 (+), 45.3 (+), 44.8 (+), 34.4 (–) 34.4 (–), 28.4 (–), 28.1 (–), 22.9 (–), 22.8 (–), 18.3 (+), 15.6 (+), 14.2 (+), 14.2 (+).

HRMS (APCI) (m/z): [MNH_4^+] ($C_{14}H_{26}NO_2^+$) calc.: 240.1958, found: 240.1961.

Yield: 43% (slightly yellow liquid).

2-(4-Methoxyphenyl)-4-methylpentan-3-ol (6b)

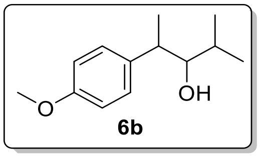

Column chromatography: *n*-Pentane/EtOAc 0-25%.

^{1}H-NMR (diastereomeric mixture) (400 MHz, $CDCl_3$, δ_H): 7.21-7.11 (m, 2H), 6.89-6.83 (m, 2H), 3.80 [3.79] (s, 3H), 3.40-3.34 (m, 1H), 2.89-2.77 (m, 1H), 1.81-1.55 (m, 1H), 1.43 (bs, 1H), 1.28 [1.23] (d, *J* = 7.0 Hz, 3H), 1.04-0.88 (m, 6H).

^{13}C-NMR (diastereomeric mixture) (101 MHz, $CDCl_3$, δ_C): 158.4 (C_q), 158.2 (C_q), 137.3 (C_q), 136.1 (C_q), 129.2 (+), 128.6 (+), 114.1 (+), 114.0 (+), 81.5 (+), 80.7 (+), 55.4 (+), 42.6 (+), 42.0 (+), 30.3 (+), 30.1 (+), 20.6 (+), 20.1 (+), 18.9 (+), 16.9 (+), 15.9 (+), 15.6 (+).

HRMS (APCI) (m/z): [MNH_4^+] ($C_{13}H_{24}NO_2^+$) calc.: 226.1802, found: 226.1802.

Yield: 31% (slightly yellow liquid).

2-(4-Methoxyphenyl)-5-methylhexan-3-ol (6c)

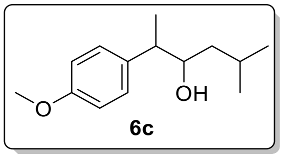

^{1}H-NMR (diastereomeric mixture) (400 MHz, $CDCl_3$, δ_H): 7.18-7.11 (m, 2H), 6.89-6.84 (m, 2H), 3.80 (s, 3H), 3.74-3.64 (m, 1H), 2.76-2.62 (m, 1H), 1.87-1.71 (m, 1H), 1.43 (bs, 1H), 1.34-1.13 (m, 5H), 0.94-0.84 (m, 6H).

^{13}C-NMR (diastereomeric mixture) (101 MHz, $CDCl_3$, δ_C): 158.4 (C_q), 158.2 (C_q), 136.8 (C_q), 135.5 (C_q), 129.3 (+), 128.8 (+), 114.1 (+), 114.0 (+), 74.3 (+), 74.2 (+), 55.4 (+), 45.9 (+), 45.2 (+), 44.1 (–), 43.9 (–), 24.9 (+), 24.8 (+), 24.0 (+), 23.8 (+), 21.9 (+), 21.8 (+), 18.3 (+), 15.6 (+).

HRMS (APCI) (m/z): [MNH_4^+] ($C_{14}H_{26}NO_2^+$) calc.: 240.1958, found: 240.1962.

Yield: 37% (slightly yellow liquid).

2-(4-Methoxyphenyl)-5,5-dimethylhexan-3-ol (6d)

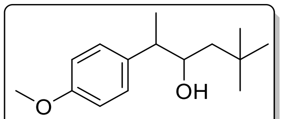

6d

^{1}H-NMR (diastereomeric mixture) (300 MHz, $CDCl_3$, δ_H): 7.19-7.11 (m, 2H), 6.89-6.83 (m, 2H), 3.82-3.70 (m, 4H), 2.74-2.61 (m, 1H), 1.50 [1.37] (dd, *J* = 14.6, 1.5 Hz, 2H), 1.27 [1.25] (d, *J* = 7.1 Hz, 3H), 0.94 [0.90] (s, 9H).

^{13}C-NMR (diastereomeric mixture) (75 MHz, $CDCl_3$, δ_C): 158.4 (C_q), 158.2 (C_q), 136.8 (C_q), 135.4 (C_q), 129.3 (+), 128.9 (+), 114.0 (+), 113.9 (+), 74.0 (+), 73.8 (+), 55.4 (+), 48.5 (–), 48.5 (–), 46.6 (+), 46.3 (+), 30.4 (C_q), 30.4 (C_q), 30.3 (+), 30.2 (+), 18.1 (+), 15.3 (+).

HRMS (APCI) (m/z): [MNH_4^+] ($C_{15}H_{28}NO_2^+$) calc.: 254.2115, found: 254.2114.

Yield: 16% (slightly yellow liquid).

1-Cyclohexyl-2-(4-methoxyphenyl)propan-1-ol (6e)

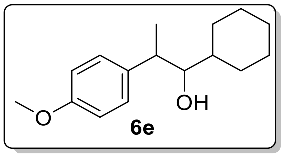

6e

Column chromatography: *n*-Pentane/EtOAc 0-25%.

^{1}H-NMR (diastereomeric mixture) (400 MHz, $CDCl_3$, δ_H): 7.20-7.11 (m, 2H), 6.89-6.84 (m, 2H), 3.80 (s, 3H), 3.39-3.33 (m, 1H), 2.95-2.82 (m, 1H), 1.95-1.59 (m, 5H), 1.45-1.19 (m, 10H).

^{13}C-NMR (diastereomeric mixture) (101 MHz, $CDCl_3$, δ_C): 158.4 (C_q), 158.1 (C_q), 137.4 (C_q), 136.0 (C_q), 129.2 (+), 128.7 (+), 114.1 (+), 114.0 (+), 80.7 (+), 80.3 (+), 55.4 (+), 41.8 (+), 41.0 (+), 40.2 (+), 40.2 (+), 30.8 (–), 30.1 (–), 27.9 (–), 26.7 (–), 26.7 (–), 26.6 (–), 26.4 (–), 26.4 (–), 26.1 (–), 19.0 (+), 15.0 (+).

HRMS (EI) (m/z): [M^+] ($C_{16}H_{24}O_2^+$) calc.: 248.1771, found: 248.1767.

Yield: 33% (slightly yellow liquid).

4-(4-Methoxyphenyl)-1-(methylthio)pentan-3-ol (6f)

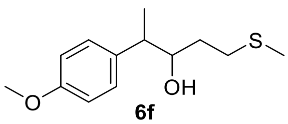

6f

Column chromatography: *n*-Pentane/EtOAc 0-25%.

^{1}H-NMR (diastereomeric mixture) (400 MHz, $CDCl_3$, δ_H): 7.18-7.10 (m, 2H), 6.89-6.83 (m, 2H), 3.81-3.71 (m, 4H), 2.77-2.52 (m, 3H), 2.09 [2.03] (s, 3H), 1.90-1.53 (m, 3H), 1.30 [1.27] (t, *J* = 7.0 Hz, 3H).

^{13}C-NMR (diastereomeric mixture) (101 MHz, $CDCl_3$, δ_C): 158.5 (C_q), 158.3 (C_q), 136.3 (C_q), 135.1 (C_q), 129.2 (+), 128.8 (+), 114.1 (+), 114.0 (+), 75.6 (+), 75.2 (+), 55.4 (+), 45.4 (+), 45.2 (+), 33.8 (–), 33.5 (–), 31.3 (–), 31.1 (–), 18.1 (+), 16.4 (+), 15.7 (+), 15.5 (+).

HRMS (APCI) (m/z): [MNH_4^+] ($C_{13}H_{24}NO_2S^+$) calc.: 258.1522, found: 258.1525.

Yield: 37% (slightly yellow liquid).

2-(4-Methoxyphenyl)-1-phenylpropan-1-ol (6g)[21]

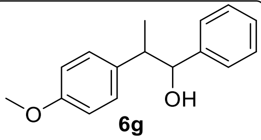

6g

Column chromatography: *n*-Pentane/EtOAc 0-35%.

^{1}H-NMR (diastereomeric mixture) (300 MHz, $CDCl_3$, δ_H): 7.29-7.09 (m, 5H), 7.00-6.68 (m, 4H), 4.68 [4.51] (d, *J* = 5.7 Hz, [*J* = 8.7 Hz], 1H), 3.73 [3.69] (s, 3H), 3.04-2.83 (m, 1H), 1.84-1.71 (m, 1H), 1.19 [0.96] (d, *J* = 7.1 Hz, 3H).

^{13}C-NMR (diastereomeric mixture) (75 MHz, $CDCl_3$, δ_C): 158.6 (C_q), 158.2 (C_q), 143.0 (C_q), 142.7 (C_q), 135.6 (C_q), 135.3 (C_q), 129.1 (+), 128.4 (+), 128.1 (+), 127.9 (+), 127.3 (+), 127.1 (+), 126.4 (+), 114.2 (+), 113.7 (+), 79.9 (+), 78.9 (+), 55.4 (+), 55.3 (+), 47.5 (+), 46.4 (+), 18.6 (+), 15.2 (+).

HRMS (APCI) (m/z): [MNH_4^+] ($C_{16}H_{22}NO_2^+$) calc.: 260.1645, found: 260.1645.

Yield: 23% (slightly yellow liquid).

Methyl 4-(1-hydroxy-2-(4-methoxyphenyl)propyl)benzoate (6h)

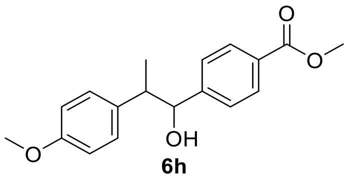

6h

Column chromatography: *n*-Pentane/EtOAc 0-25%.

^{1}H-NMR (diastereomeric mixture) (400 MHz, $CDCl_3$, δ_H): 8.02-7.90 (m, 2H), 7.40-7.23 (m, 2H), 7.18-7.00 (m, 2H), 6.90-6.76 (m, 2H), 4.79 [4.66] (d, *J* = 5.8 Hz, [*J* = 8.1 Hz], 1H), 3.91 [3.89] (s, 3H), 3.80 [3.76] (s, 3H), 3.10-2.92 (m, 1H), 1.98 (s, 1H), 1.26 [1.08] (d, *J* = 7.1 Hz , 3H).

^{13}C-NMR (diastereomeric mixture) (101 MHz, $CDCl_3$, δ_C): 167.1 (C_q), 167.1 (C_q), 158.7 (C_q), 158.4 (C_q), 148.2 (C_q), 147.9 (C_q), 135.0 (C_q), 134.5 (C_q), 129.6 (+), 129.6 (C_q), 129.4 (+), 129.2 (+), 129.1 (+), 129.0 (C_q), 127.0 (+), 126.4 (+), 114.2 (+), 113.8 (+), 79.3 (+), 78.6 (+), 55.4 (+), 55.3 (+), 52.2 (+), 52.2 (+), 47.4 (+), 46.4 (+), 18.2 (+), 15.2 (+).

HRMS (ESI) (m/z): [MNH_4^+] ($C_{18}H_{24}NO_4^+$) calc.: 318.1700, found: 318.1704.

Yield: 39% (slightly yellow liquid).

2-Phenylhetan-3-ol (6i)[22-24]

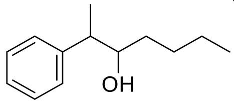

6i

Column chromatography: *n*-Pentane/EtOAc 0-30%.

^{1}H-NMR (diastereomeric mixture) (300 MHz, $CDCl_3$, δ_H): 7.37-7.17 (m, 5H), 3.70-3.62 (m, 1H), 2.84-2.70 (m, 1H), 1.63-1.22 (m, 10H), 0.91 [0.87] (t, *J* = 7.0 Hz, 3H).

^{13}C-NMR (diastereomeric mixture) (75 MHz, $CDCl_3$, δ_C): 144.8 (C_q), 143.7 (C_q), 128.7 (+), 128.6 (+), 128.3 (+), 127.9 (+), 126.8 (+), 126.5 (+), 76.4 (+), 76.2 (+), 46.2 (+), 45.7 (+), 34.5 (–), 34.4 (–), 28.4 (–), 28.1 (–), 22.9 (–), 22.8 (–), 18.2 (+), 15.5 (+), 14.3 (+), 14.2 (+).

HRMS (APCI) (m/z): [MNH_4^+] ($C_{13}H_{24}NO^+$) calc.: 210.1852, found: 210.1853.

Yield: 28% (slightly yellow liquid).

General procedure for the intramolecular ring closure with esters as electrophiles (general procedure C)

A 5 mL crimp cap vial equipped with a magnetic stirring bar was loaded with 3DPA2FBN (3.8 mg, 6.00 µmol, 3 mol%), K_2CO_3 (13.8 mg, 100 µmol, 50 mol%), $(^iPr)_3SiSH$ (4.3 µL, 20.0 µmol, 10 mol%), the corresponding ester (200 µmol, 1 eq.), 4Å molecular sieve (50 mg) and dry MeCN. In doing so, all solid compounds were added before capping the vial, whereas all liquid compounds were added *via* syringe after setting the capped vial under inert conditions. The reaction mixture was degassed by three cycles of freeze-pump-thaw and subsequently stirred under light irradiation using a 455 nm (±15 nm) LED for 16 h at 25 °C. Two reaction batches were combined and diluted with brine (10 mL), water (10 mL) and ethyl acetate (15 mL). The phases were separated, and the water phase was extracted with ethyl acetate (3 x 7 mL). The combined organic phases were washed with brine (10 mL) and dried over Na_2SO_4. The solvent was removed under reduced pressure and the crude product was purified by automated flash column chromatography (n-pentane/DCM 50-100%)

2-Phenylcyclopentan-1-one (8a)[25]

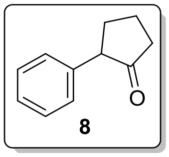

^{1}H-NMR (diastereomeric mixture) (300 MHz, $CDCl_3$, δ_H): 7.38-7.16 (m, 5H), 3.38-3.28 (m, 1H), 2.57-2.43 (m, 2H), 2.37-2.24 (m, 1H), 2.23-2.05 (m, 2H), 2.03-1.87 (m, 1H).

^{13}C-NMR (diastereomeric mixture) (75 MHz, $CDCl_3$, δ_C): 218.2 (C_q), 138.5 (C_q), 128.7 (+), 128.3 (+), 127.0 (+), 55.5 (+), 38.6 (–), 31.9 (–), 21.0 (–).

HRMS (EI) (m/z): [M^+] ($C_{11}H_{12}O^+$) calc.: 160.0883, found: 160.0881.

Yield: 40% (slightly yellow liquid).

2-Phenylcyclohexan-1-one (8b)[26]

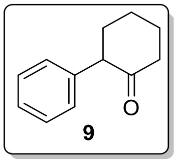

Column chromatography: *n*-Pentane/DCM 50-100%.

^{1}H-NMR (diastereomeric mixture) (300 MHz, $CDCl_3$, δ_H): 7.37-7.22 (m, 3H), 7.17-7.11 (m, 2H), 3.63 [3.59] (d, J = 5.4 Hz, 1H), 2.58-2.40 (m, 2H), 2.33-2.22 (m, 1H), 2.21-2.09 (m, 1H), 2.09-1.92 (m, 2H), 1.91-1.74 (m, 2H).

^{13}C-NMR (diastereomeric mixture) (75 MHz, $CDCl_3$, δ_C): 210.5 (C_q), 138.9 (C_q), 128.7 (+), 128.5 (+), 127.0 (+), 57.6 (+), 42.4 (–), 35.3 (–), 28.0 (–), 25.5 (–).

HRMS (EI) (m/z): [M^+] ($C_{12}H_{14}O^+$) calc.: 174.1039, found: 174.1035.

Yield: 9% (white solid).

3. Detailed optimization of the reaction conditions

3.1. Optimization process with ketones as electrophiles

When the reaction was performed with ethylbenzene **1a**, 4CzIPN **A** as a photocatalyst, (^{i}Pr)$_3$SiSH as a hydrogen atom transfer catalyst, K_2CO_3 as a base and acetone **2a** as an electrophile, traces of the desired product **3a** could be observed (able 1, entry 1). A higher yield of 21% could be obtained by adding grinded 4 Å molecular sieves to the reaction (table 2, entry 2). Increasing the amount of **2a** by using it as a co-solvent in a 1:1 mixture with dry acetonitrile gave a yield of 49% (table 1, entry 3). A higher loading of hydrogen atom transfer catalyst (30 mol% instead of 20 mol%) or photocatalyst from (10 mol% instead of 5) decreased the yield (table 1, entries 4 and 5) while reducing the amount of (^{i}Pr)$_3$SiSH gave a slightly enhanced yield (table 1, entry 6). Using 3DPA2FBN **B** as a photocatalyst increased the yield to 50% when 10 equivalents **2a** was used and 86% when acetone was used as a co-solvent (table 1, entries 7 and 8). The reaction could be improved slightly by reducing the loading of photocatalyst **B** to 3 mol% and the amount of K_2CO_3 to 10 mol% (table 1, entry 9). Control experiments showed, that the yield is significantly lower when the reaction is performed without the base and no product is formed at all without light, photocatalyst or hydrogen atom transfer catalyst (table 1, entries 10-13).

Table S1 – Optimization of the reaction conditions for the photocatalytic HAT-reaction of ethylbenzene with acetone as an electrophile.[a]

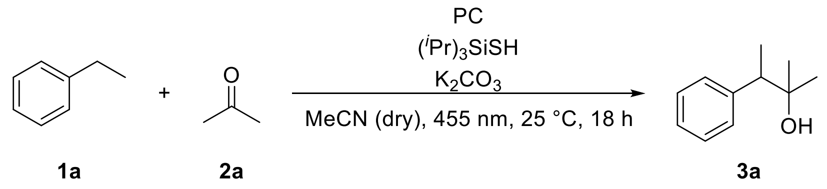

Entry	Amount of 2a	Photocatalyst (mol%)	Amount of (^{i}Pr)$_3$SiSH	Amount of base	Additive	Yield [%][b]
1	10 eq.	4CzIPN (5)	20 mol%	20 mol%	–	3
2	10 eq.	4CzIPN (5)	20 mol%	20 mol%	4 Å MS (100 mg)	21
3	Co-solvent (1:1)	4CzIPN (5)	20 mol%	20 mol%	4 Å MS (100 mg)	49
4	10 eq.	4CzIPN (5)	30 mol%	30 mol%	4 Å MS (100 mg)	17
5	10 eq.	4CzIPN (10)	10 mol%	20 mol%	4 Å MS (50 mg)	18
6	10 eq.	4CzIPN (5)	10 mol%	20 mol%	4 Å MS (50 mg)	30
7	10 eq.	3DPA2FBN (5)	10 mol%	20 mol%	4 Å MS (50 mg)	50
8	Co-solvent (1:1)	3DPA2FBN (5)	10 mol%	20 mol%	4 Å MS (50 mg)	86
9	10 eq.	3DPA2FBN (3)	10 mol%	10 mol%	4 Å MS (50 mg)	59
10	10 eq.	3DPA2FBN (5)	10 mol%	–	4 Å MS (50 mg)	27
11	10	–	10 mol%	20 mol%	4 Å MS (100 mg)	0
12[c]	10	4CzIPN (5)	10 mol%	20 mol%	4 Å MS (100 mg)	0
13	10	4CzIPN (5)	–	20 mol%	4 Å MS (100 mg)	0

[a] The reaction was performed using 1 eq. (0.2 mmol) **1a** in 2 mL degassed solvent, [b] yields were determined with GC-FID analysis using *n*-decane as an internal standard, [c] reaction was performed in the dark.

3.1.1. Screening of different photocatalysts

Table S2 – Optimization of the reaction conditions: screening of different photocatalysts.[a]

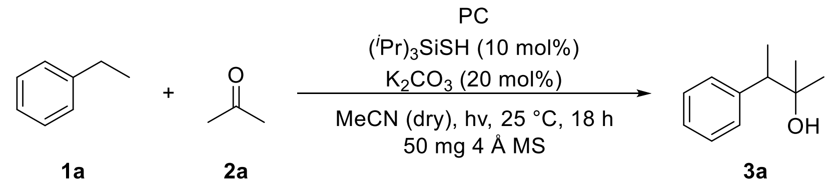

Entry	Photocatalyst (mol%, hν [nm])	Yield[b] [%]
1	4CzIPN (5, 455)	30
2	3DPA2FBN (5, 455)	50
3	3DPAFIPN (5, 455)	28
4	$Ru(bpy)_3Cl_2$ (5, 455)	0
5	Eosin Y (5, 535)	0
6	Fluorescein (5, 535)	0
7	Rhodamine 6G (5, 455)	0
8	*fac*-$Ir(ppy)_3$ (2, 400)	0
9	$(Ir[dF(CF_3)ppy]_2(dtbpy))PF_6$ (2, 400)	0
10	Miyake catalyst (5, 400)	0

[a] The reaction was performed using 1 eq. (0.2 mmol) **1a** and 10 eq. (2 mmol) **2a** in 2 mL dry, degassed acetonitrile, [b] yields were determined with GC-FID analysis using *n*-decane as an internal standard.

3.1.2. Screening of different hydrogen atom transfer catalysts

Table S3 – Optimization of the reaction conditions: screening of different hydrogen atom transfer catalysts.[a]

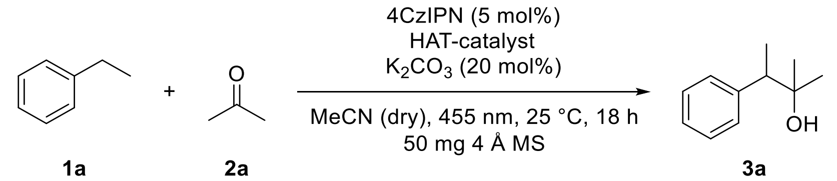

Entry	HAT-catalyst (mol%)	Yield[b] [%]
1	(^{i}Pr)$_3$SiSH (20 mol%)	30
2	Methyl thioglycolate (20 mol%)	Traces
3	Ethyl 2-mercaptopropionate (20 mol%)	Traces
4	3-Mercaptopropyltrimethoxysilan (20 mol%)	0
5	Quinuclidine (20 mol%)	0

6	1,1'-Binaaphthyl-2,2'-diyl hydrogenphosphate (20 mol%)	0
7	*N*-(3,5-bis(trifluoromethyl)phenyl)-2,4,6-triisopropylbenzenesulfonamide[27] (20 mol%)	0
8	NaBr (20 mol%)	0
9	$(NH_4)Br$ (20 mol%)	0

[a] The reaction was performed using 1 eq. (0.2 mmol) **1a** and 10 eq. (2 mmol) **2a** in 2 mL dry, degassed acetonitrile, [b] yields were determined with GC-FID analysis using *n*-decane as an internal standard.

3.1.3. Investigations on product inhibition and in-situ protection of alcohols

Table S4 – Optimization of the reaction conditions: investigations on product inhibition.[a]

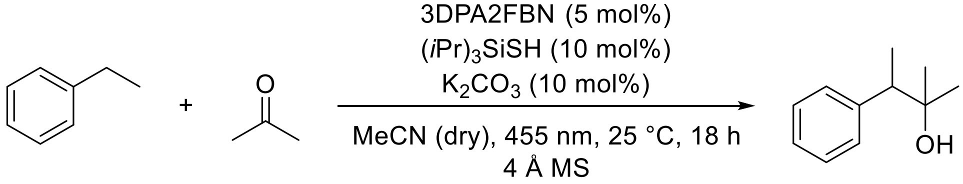

1a **2a** **3a**

Entry	Additive	Yield [%][b]
1[c]	3DPA2FBN (5 mol%)	41
2[c]	$(^iPr)_3SiSH$ (10 mol%)	50
3[c]	3DPA2FBN (3 mol%) $(^iPr)_3SiSH$ (10 mol%)	60
4	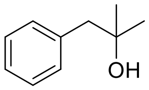 **4** (0.5 eq.)	39
5	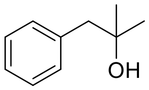 **4** (1 eq.)	11
6	1-Heptanol (1 eq.)	21
7	TMS-Cl (1 eq.)	–
8	TMS-DMA (1 eq.)	–
9	BSTFA (1 eq.)	traces
10	Heptamethyldisilazane (1 eq.)	traces

[a] The reaction was performed using 1 eq. (0.2 mmol) **1a** and 10 eq. (2 mmol) **2a** in 2 mL dry, degassed acetonitrile, [b] yields were determined with GC-FID analysis using *n*-decane as an internal standard, [c] additional catalyst was added after 14 h.

3.2. Optimization process with aldehydes as electrophiles

General procedure for the reaction optimization process with aldehydes as electrophiles (general procedure D)

A 5 mL crimp cap vial equipped with a magnetic stirring bar was loaded with photocatalyst, base, ethylbenzene (**1a**), *n*-pentanal (**5a**), solvent and if noted an additive in the amounts given in the corresponding tables (S5-S15). In doing so, all solid compounds were added before capping the vial, whereas all liquid compounds were added *via* syringe after setting the capped vial under inert conditions. The reaction mixture was degassed by three cycles of freeze-pump-thaw and subsequently stirred under light irradiation for the given time at 25 °C. Subsequently, an aliquot of the reaction mixture was submitted to GC-FID analysis to determine the product yield with 1-heptanol as internal standard.

Table S5 – Benzylation of aldehydes, first successful reaction and control experiments.

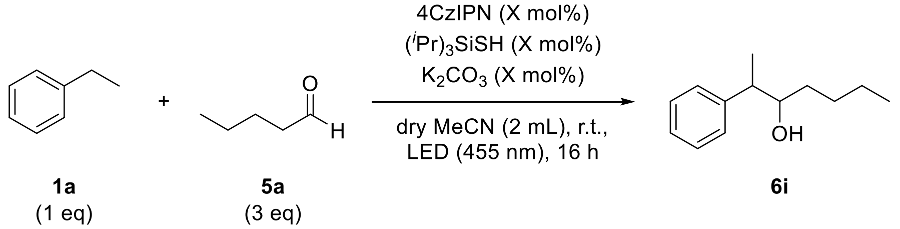

Entry	Photocatalyst	HAT catalyst	Base	Product formation
1	4CzIPN (5 mol%)	(^{i}Pr)$_3$SiSH (10 mol%)	K_2CO_3 (50 mol%)	Traces Detected by GC/MS
2	–	(^{i}Pr)$_3$SiSH (10 mol%)	K_2CO_3 (50 mol%)	not detected (n.d.)
3	4CzIPN (5 mol%)	–	K_2CO_3 (50 mol%)	n.d.
4[b]	4CzIPN (5 mol%)	(^{i}Pr)$_3$SiSH (10 mol%)	K_2CO_3 (50 mol%)	n.d.
5	4CzIPN (5 mol%)	(^{i}Pr)$_3$SiSH (10 mol%)	–	Traces Detected by GC/MS

[a] Reactions were performed with **1a** (150 µmol, 1 eq.) and **5a** (450 µmol, 3 eq.) in degassed dry MeCN (2 mL) under a nitrogen atmosphere and irradiation using a 455 nm (±15 nm) LED. [b] Reaction performed in absence of light.

Table S6 – Benzylation of aldehydes, ethylbenze/aldehyde ratio alteration.[a]

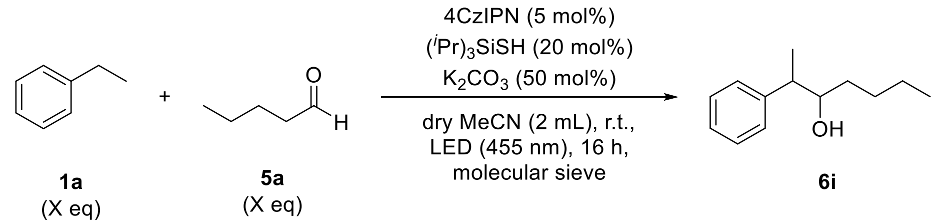

Entry	Ethylbenzene eq.	*n*-Pentanal eq.	Yield[b] [%]
1	1	10	Traces
2	1	3	3
3	1	1	10
4	3	1	16
5[c]	3	1	20

[a] Reactions were performed with 1 eq. being 150 µmol, 4CzIPN (5 mol% in respect to 1 eq), (^{i}Pr)$_3$SiSH (20 mol%), K_2CO_3 (50 mol%) and molecular sieve (4 Å, 50 mg) in degassed dry MeCN (2 mL) under a nitrogen atmosphere and irradiation using a 455 nm (±15 nm) LED. [b] GC-Yield using 1-heptanol as internal standard. [c] No molecular sieve.

Table S7 – Benzylation of aldehydes, variation of base and HAT amount.[a]

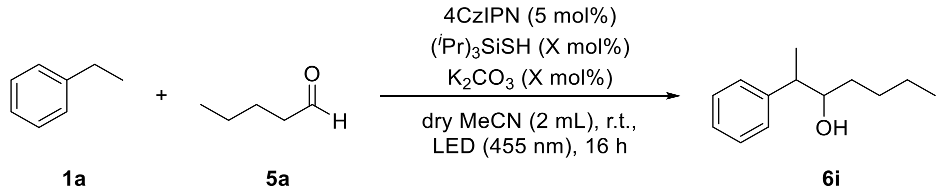

Entry	(^{i}Pr)$_3$SiSH amount [mol%]	K_2CO_3 amount [mol%]	Yield[b] [%]
1	10	50	10
2	20	50	20
3	50	50	8
4	20	–	6
5	20	10	20
6	20	100	18

[a] Reactions were performed with **5a** (150 µmol, 1 eq), **1a** (450 µmol, 3 eq), 4CzIPN (5 mol%), (^{i}Pr)$_3$SiSH and K_2CO_3 in degassed dry MeCN (2 mL) under a nitrogen atmosphere and irradiation using a 455 nm (±15 nm) LED. [b] GC-Yield using 1-heptanol as internal standard.

Table S8 – Benzylation of aldehydes, change of HAT catalyst and solvent.[a]

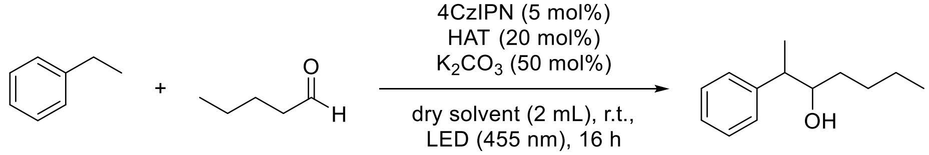

1a **5a** **6i**

Entry	Solvent	HAT catalyst	Yield[b] [%]
1	MeCN	$(^i Pr)_3SiSH$	20
2	DMF	$(^i Pr)_3SiSH$	2
3[c]	DMF	Quinuclidine	n.d.
4	MeCN	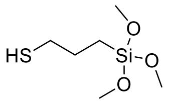	2
5	MeCN	$HS-C_9H_{19}$	3
6	MeCN	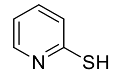	not detected (n.d.)
7	MeCN	NaBr	n.d.

[a] Reactions were performed with **5a** (150 µmol, 1 eq), **1a** (450 µmol, 3 eq), 4CzIPN (5 mol%), HAT catalyst (20 mol%) and K_2CO_3 (50 mol%) in degassed dry MeCN (2 mL) under a nitrogen atmosphere and irradiation using a 455 nm (±15 nm) LED. [b] GC-Yield using 1-heptanol as internal standard. [c] Toluene instead of ethylbenzene was used.

Table S9 – Benzylation of aldehydes, photocatalyst screening.[a]

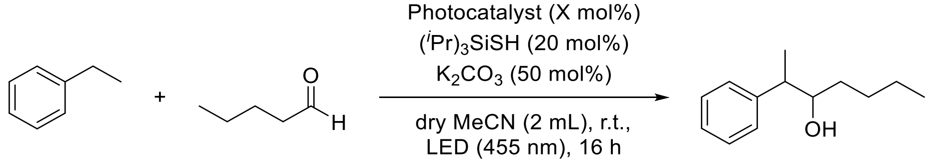

1a **5a** **6i**

Entry	Photocatalyst (ratio)	Yield[b] [%]
1	4CzIPN (5 mol%)	20
2	3DPA2FBN (5 mol%)	10
**3**	**3DPAFIPN (5 mol%)**	**32**
4[c]	3DPAFIPN (5 mol%)	27
5	4Cz(*p*H)BN (5 mol%)	20
6	$(Ir[dF(CF_3)ppy]_2(dtbpy))PF_6$ (2 mol%)	2
7	Miyake catalyst	n.d.

[a] Reactions were performed with **5a** (150 µmol, 1 eq), **1a** (450 µmol, 3 eq), photocatalyst, $(^i Pr)_3SiSH$ (20 mol%) and K_2CO_3 (50 mol%) in degassed dry MeCN (2 mL) under a nitrogen atmosphere and irradiation using a 455 nm (±15 nm) LED. [b] GC-Yield using 1-heptanol as internal standard. [c] 20 mol% of K_2CO_3 was used.

Table S10 – Benzylation of aldehydes, base and additive screening.[a]

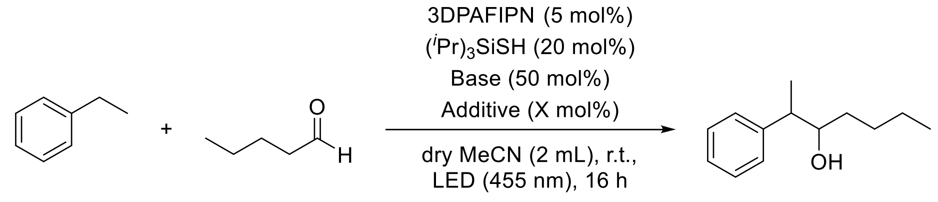

Entry	Base	Additive	Yield[b] [%]
1	K_2CO_3	–	32
2	Li_2CO_3	–	13
3	Na_2CO_3	–	15
4	Cs_2CO_3	–	7
5	Lutidin	–	13
6	K_2CO_3	B_2pin_2 (25 mol%)	33
7	K_2CO_3	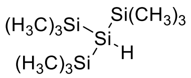 (50 mol%)	15
8	Lutidin	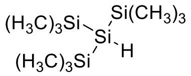 (50 mol%)	26

[a] Reactions were performed with **5a** (150 µmol, 1 eq), **1a** (450 µmol, 3 eq), 3DPAFIPN (5 mol%), (^{i}Pr)$_3$SiSH (20 mol%), base (50 mol%) and an additive if noted in degassed dry MeCN (2 mL) under a nitrogen atmosphere and irradiation using a 455 nm (±15 nm) LED. [b] GC-Yield using 1-heptanol as internal standard.

Table S11 – Benzylation of aldehydes, reaction time variation.[a]

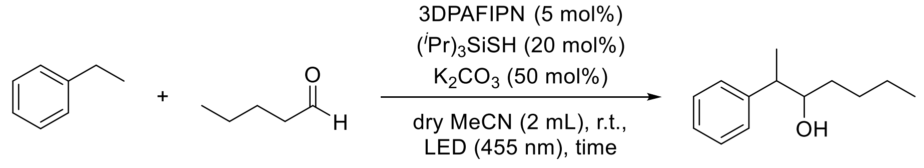

Entry	Time [h]	Yield[b] [%]	Entry	Time [h]	Yield[b] [%]
1	1	27	4	16	32
2	2	31	5[c]	16	32
3	4	33	6[d]	16	22

[a] Reactions were performed with **5a** (150 µmol, 1 eq), **1a** (450 µmol, 3 eq), 3DPAFIPN (5 mol%), (^{i}Pr)$_3$SiSH (20 mol%) and K_2CO_3 (50 mol%) in degassed dry MeCN (2 mL) under a nitrogen atmosphere and irradiation using a 455 nm (±15 nm) LED. [b] GC-Yield using 1-heptanol as internal standard. [c] The reaction was executed as described in [a] in 1.5 mL dry MeCN. After 2 h of irradiation, a dry MeCN solution (0.5 mL) containing additional 3DPAFIPN (7.5 µmol, 5 mol%) was injected *via* syringe and the mixture was irradiated for further 14 h. [d] Reaction was executed as described in

[a]. After 2 h of irradiation, a dry MeCN solution (0.5 mL) containing additional 3DPAFIPN (7.5 µmol, 5 mol%) and (^{i}Pr)$_3$SiSH (30 µmol, 20 mol%) was injected *via* syringe and the mixture was irradiated for further 14 h.

Table S12 – Benzylation of aldehydes, product inhibition test and catalyst loading.[a]

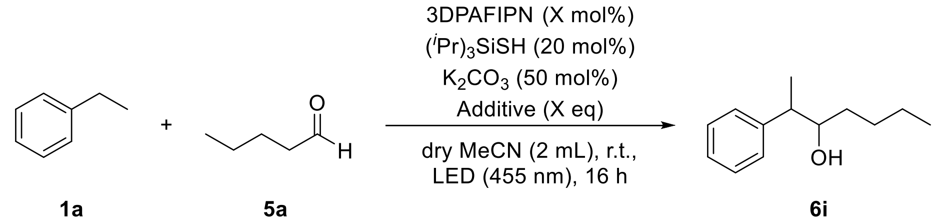

Entry	Additive	Catalyst loading [mol%]	Yield[b] [%]
1	–	3	23
2	–	5	32
3	–	10	22
4	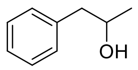 (1 eq)	5	8
5	$C_{10}H_{21}$–OH (1 eq)	5	17

[a] Reactions were performed with **5a** (150 µmol, 1 eq), **1a** (450 µmol, 3 eq), 3DPAFIPN as given in the table, (^{i}Pr)$_3$SiSH (20 mol%), K_2CO_3 (50 mol%) and an additive if noted in degassed dry MeCN (2 mL) under a nitrogen atmosphere and irradiation using a 455 nm (±15 nm) LED. [b] GC-Yield using 1-heptanol as internal standard.

Table S13. Benzylation of aldehydes, addition of alcohol protecting agents.[a]

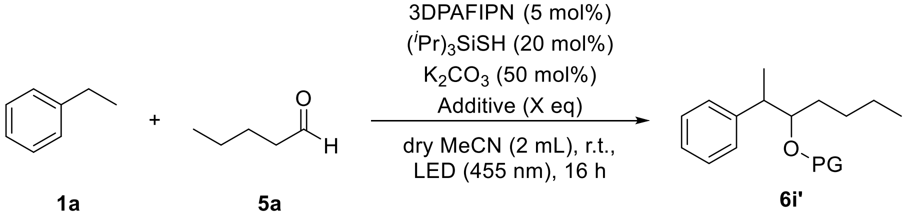

Entry	Additive	Yield 13'[b] or 13[c] [%]
1	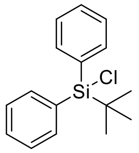 TPDPSCL (2 eq)	–
2	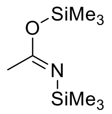 BAS (1 eq)	–

3	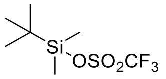	TBMS triflate (1 eq)	–
4	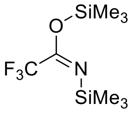	BSTFA (1 eq)	Small amount[b]
5	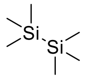	Hexamethyldisilane (1 eq)	29[c]

[a] Reactions were performed with **5a** (150 µmol, 1 eq), **1a** (450 µmol, 3 eq), 3DPAFIPN (5 mol%), (^{i}Pr)$_3$SiSH (20 mol%), K_2CO_3 (50 mol%) and protecting agent in degassed dry MeCN (2 mL) under a nitrogen atmosphere and irradiation using a 455 nm (±15 nm) LED. [b] **13'** detected by GC-MS. [c] GC-Yield of **13** using 1-heptanol as internal standard.

Table S14. Benzylation of aldehydes, variation of experimental execution.

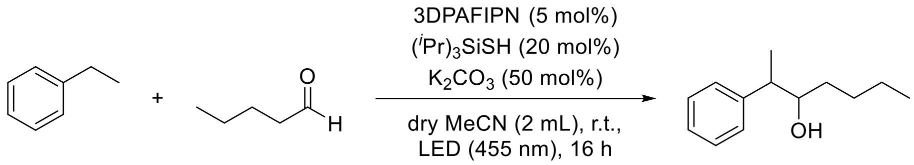

1a **5a** **6i**

Entry	Experimental variation	Yield[a] [%]
1[b]	Aldehyde added *via* syringe pump	19
2[c]	Executed in micro-flow reactor	12 (26)[d]
3[e]	Reaction temperature 0 °C	27
4[f]	1 eq set to 200 µmol instead of 150 µmol	23

[a] GC-Yield using 1-heptanol as internal standard. [b] Reaction was performed with **1a** (450 µmol, 3 eq), 3DPAFIPN (5 mol%), (^{i}Pr)$_3$SiSH (20 mol%) and K_2CO_3 (50 mol%) in degassed dry MeCN (1.5 mL) under a nitrogen atmosphere and irradiation using a 455 nm (±15 nm) LED. A solution of **5a** (150 µmol, 1 eq) in dry MeCN (0.5 mL) was added *via* syringe pump in 4 h (0.125 mL/h). After completed addition the mixture was stirred overnight (12 h). [c] Reaction was performed with **5a** (150 µmol, 1 eq), **1a** (450 µmol, 3 eq), 3DPAFIPN (5 mol%), (^{i}Pr)$_3$SiSH (20 mol%), lutidin (50 mol%) and tris(trimethylsilyl)silane (50 mol%) in degassed dry MeCN (2 mL) under a nitrogen atmosphere and irradiation using a 455 nm (±15 nm) LED in a micro-flow reactor (reactor retention time 1.7 h). [d] Reaction was performed as described in [c], yet in batch over 16 h. [e] Reaction was performed with **5a** (150 µmol, 1 eq), **1a** (450 µmol, 3 eq), 3DPAFIPN (5 mol%), (^{i}Pr)$_3$SiSH (20 mol%) and K_2CO_3 (50 mol%) in degassed dry MeCN (2 mL) under a nitrogen atmosphere and irradiation using a 455 nm (±15 nm) LED at 0 °C. [f] Reaction was performed with **1a** (200 µmol, 1 eq), **5a** (600 µmol, 3 eq), 3DPAFIPN (5 mol%), (^{i}Pr)$_3$SiSH (20 mol%) and K_2CO_3 (50 mol%) in degassed dry MeCN (2 mL) under a nitrogen atmosphere and irradiation using a 455 nm (±15 nm) LED.

Table S15. Benzylation of aldehydes, repetition of control experiments with optimized conditions.[a]

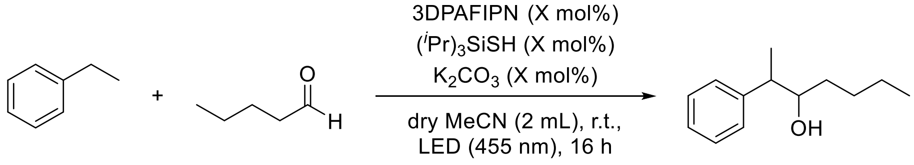

Entry	Photocatalyst	HAT catalyst	Base	Yield[b] [%]
**1**	**3DPAFIPN (5 mol%)**	**(^{i}Pr)$_3$SiSH (20 mol%)**	**K_2CO_3 (50 mol%)**	**32 (28)**[c]
2	–	(^{i}Pr)$_3$SiSH (20 mol%)	K_2CO_3 (50 mol%)	n.d.
3	3DPAFIPN (5 mol%)	–	K_2CO_3 (50 mol%)	n.d.
4	3DPAFIPN (5 mol%)	(^{i}Pr)$_3$SiSH (20 mol%)	–	10
5[d]	3DPAFIPN (5 mol%)	(^{i}Pr)$_3$SiSH (20 mol%)	K_2CO_3 (50 mol%)	n.d.

[a] Reactions were performed with **5a** (150 µmol, 1 eq) and **1a** (450 µmol, 3 eq) in degassed dry MeCN (2 mL) under a nitrogen atmosphere. [b] GC-Yield using 1-heptanol as internal standard. [c] Isolated yield in parentheses. [d] Executed in the dark.

4. Unsuccessful transformations

Table S16. Unsuitable substrates for the photocatalytic C-H to carbanion activation followed by the addition to carbonyl compounds.

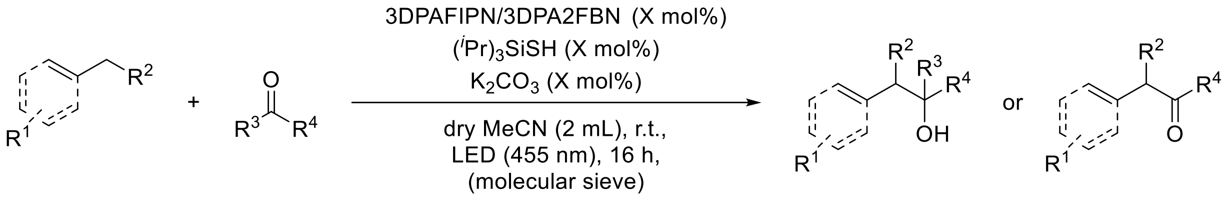

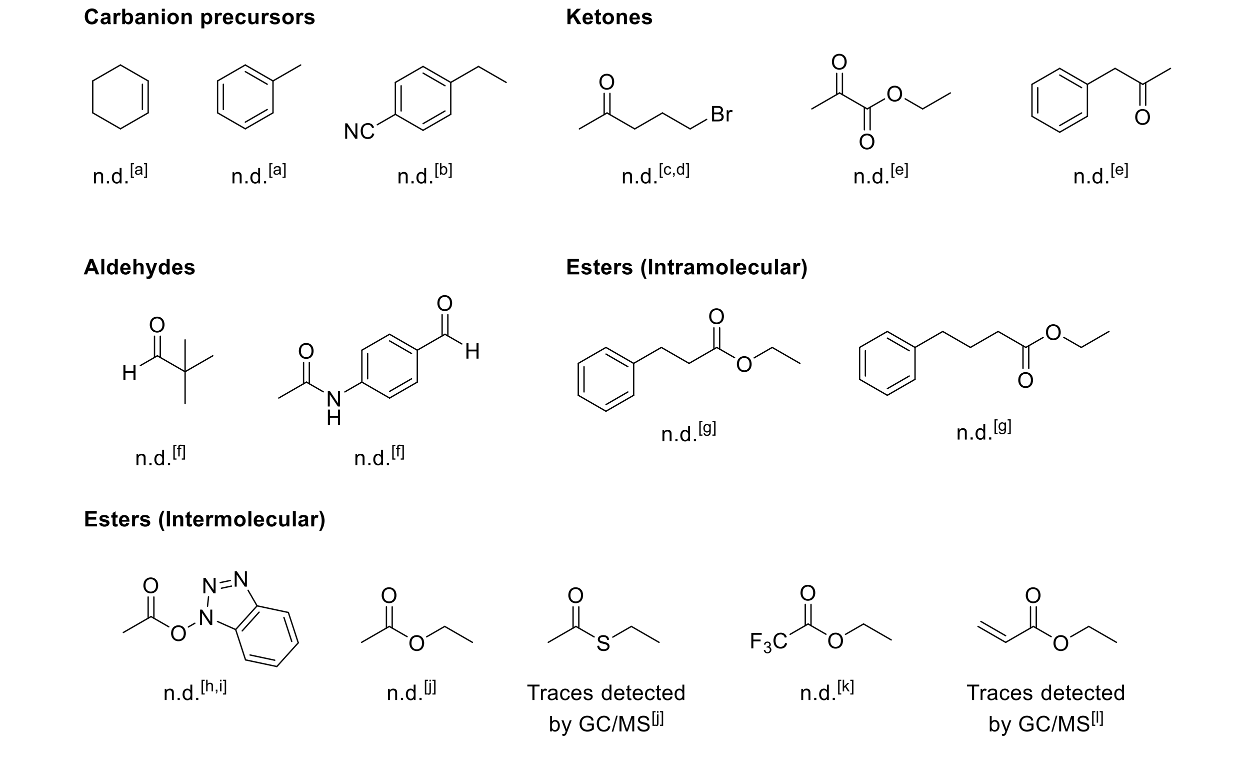

[a] Reaction performed according to general procedure A with acetone as electrophile. [b] Reaction performed according to General procedure B with n-pentanal as electrophile. [c] Reaction performed according to general procedure A with ethylbenzene as carbanion precursor. [d] Substrate synthesized according to literature procedure.[28] [e] Reaction performed according to general procedure A with 4-ethylaniosl as carbanion precursor. [f] Reaction performed according to general grocedure B with 4-ethylanisol as carbanion precursor. [g] Reaction performed according to general procedure C. [h] Reaction performed according to general procedure C with ethylbenzene (200 µmol, 1 eq.) as carbanion precursor and the corresponding electrophile (1 eq.). [i] Substrate synthesized according to an adapted literature procedure.[29] [j] Reaction performed according to general procedure C with ethylbenzene (200 µmol, 1 eq.) as carbanion precursor and the corresponding electrophile (10 eq.). [k] Reaction performed according to [j] with 3 eq. electrophile. [l] Reaction performed according to [j] with 1 eq. electrophile.

Table S17. Attempted S_N2 reactions.

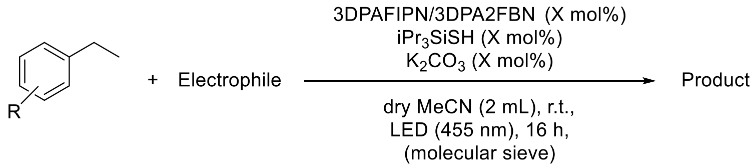

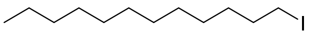

n.d.[a]

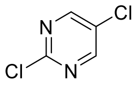

n.d.[b]

[a] Reaction performed according to General Procedure B with ethylbenzene as carbanion precursor. [b] Reaction performed according to General Procedure A with ethylbenzene (200 µmol, 1 eq.) as carbanion precursor and the corresponding electrophile (2 eq.).

These unsuccessful transformations, especially the ones with esters as substrates, indicate a diminished reactivity of the generated carbanion intermediate presumably due to coordination or complexation with other reactants like the photocatalyst. Further investigations on the exact nature of this intermediate are currently ongoing.

5. Mechanistic investigations

5.1. Reaction kinetics

The reactions were performed using 1 eq. ethylbenzene **1a** (0.2 mmol), 10 eq. acetone **2a** (2 mmol), 3 mol% photocatalyst, 10 mol% (^{i}Pr)$_3$SiSH, 10 mol% K_2CO_3 and 50 mg grinded 3Å molecular sieves in 2 ml dry, degassed acetonitrile. The reaction was irradiated with blue LEDs (455 nm ±15 nm). The yield of the reactions were determined with GC-FID analysis using *n*-decane as an internal standard.

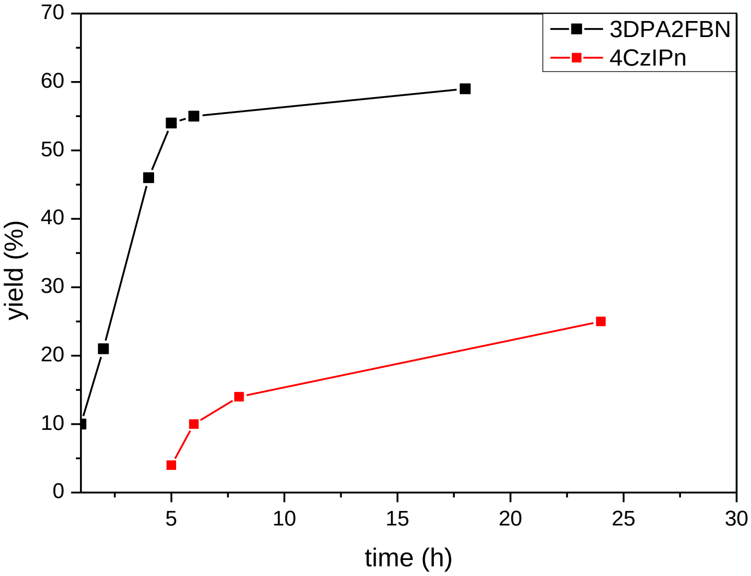

Figure S1 – Kinetic profile of the reaction with 4CzIPN (**A**) and 3DPA2FBN (**B**) as a photocatalyst.

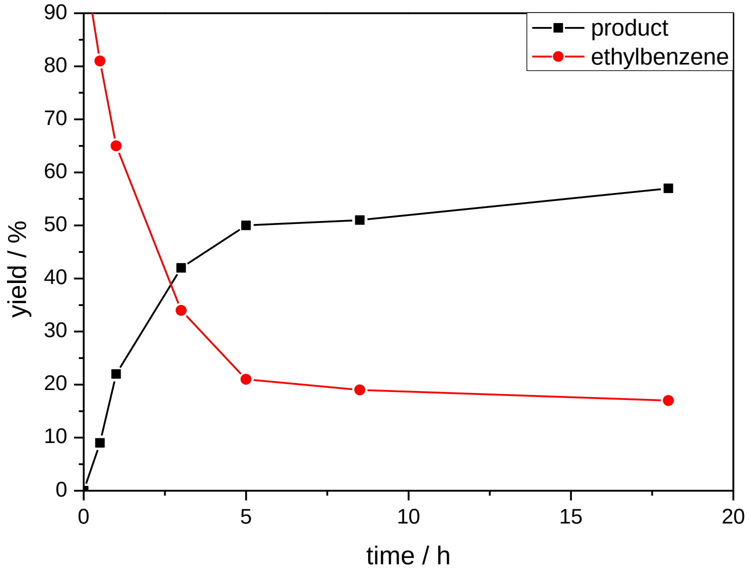

Figure S2 – Product formation and consumption of starting material during the reaction.

5.2. Emission quenching studies (support for SET oxidation of HAT- by excited photocatalyst)

For the emission quenching of 3DPAFIPN with $(^{i}Pr)_3SiS^-$, a 37.5 μM solution of 3DPAFIPN in degassed p.A. MeCN (6.1 mg 3DPAFIPN diluted in 250 mL p.A. MeCN) was given into a gas-tight 10 mm quartz cuvette and set under a nitrogen atmosphere. The photocatalyst was irradiated at 435 nm and the change of the fluorescence emission upon addition of different amounts of quencher solution was measured (figure S3). The quencher solution was prepared by the addition of the above described 37.5 μM 3DPAFIPN solution to K_2CO_3 (11.1 mg, 80 μmol) and $(^{i}Pr)_3SiSH$ (17.1 μL, 80 μmol) in a volumetric flask (2 mL). As K_2CO_3 is not soluble in organic solvents, a solid residue remains, which can be K_2CO_3 or $KHCO_3$.

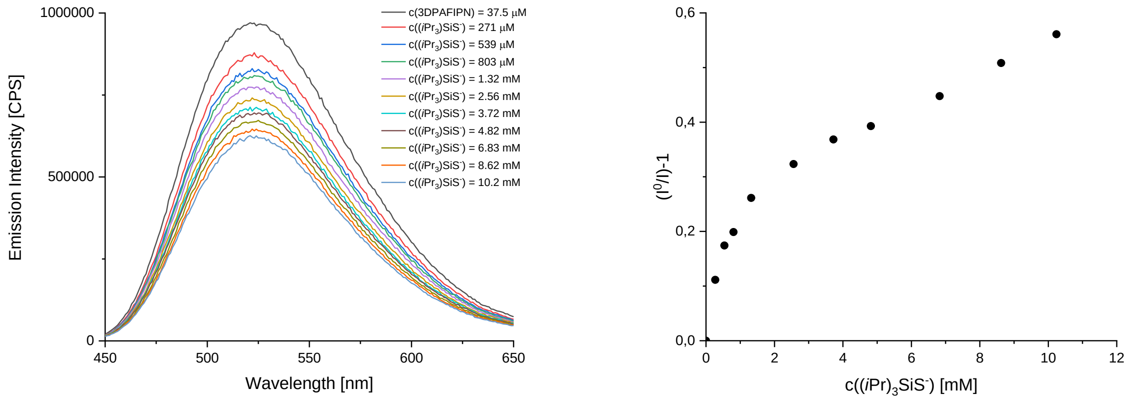

Figure S3 – Left: Emission quenching of 3DPAFIPN (37.5 μM in MeCN) upon titration with a quencher solution containing $(^{i}Pr)_3SiSH$ (41 mM in MeCN) and 3DPAFIPN (37.5 μM in dry MeCN) treated with K_2CO_3. Right: Corresponding Ster-Volmer plot (I_{max} at 526 nm).

An efficient fluorescence quenching of 3DPAFIPN upon addition of $(^{i}Pr)_3SiS^-$ was observed, indicating an interaction between the excited photocatalyst and the deprotonated HAT catalyst. However, a linear Stern-Volmer correlation was not obtained.

The measurement was executed in the same manner using 3DPA2FBN instead of 3DPAFIPN (figure S4) using an excitation wavelength of 400 nm. An efficient fluorescence quenching of 3DPAFIPN upon addition of $(^{i}Pr)_3SiS^-$ was observed, indicating an interaction between the excited photocatalyst and the deprotonated HAT catalyst (Figure S4, left). By plotting (I^0/I)-1 versus the quencher concentration, a Stern-Volmer constant of K_{SV} = 42.1 M^{-1} was determined from the slope of the linear fit (figure S4, right):

$$\frac{I^0}{I} - 1 = K_{SV} \cdot [Q]$$

(With I^0 being the fluorescence intensity at 490 nm in absence of the quencher, I the fluorescence intensity at 490 nm in presence of the quencher and [Q] the quencher concentration)

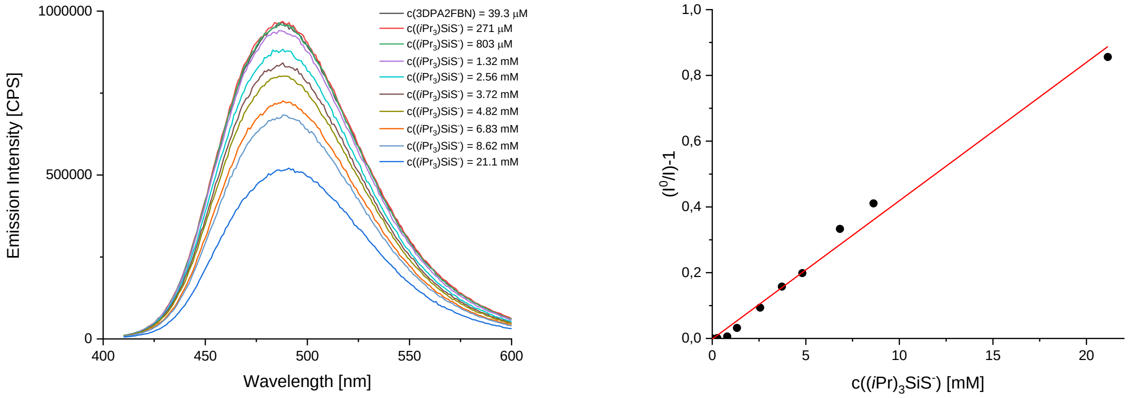

Figure S4 – Left: Emission quenching of 3DPA2FBN (39.3 μM in MeCN) upon titration with a quencher solution containing (^{i}Pr)$_3$SiSH (41 mM in MeCN) and 3DPAFIPN (39.3 μM in dry MeCN) treated with K_2CO_3. Right: Corresponding Ster-Volmer plot (I_{max} at 490 nm).

5.3. Cyclic voltammetry measurements (further support for the feasibility of SET oxidation of the HAT- by the photocatalyst)

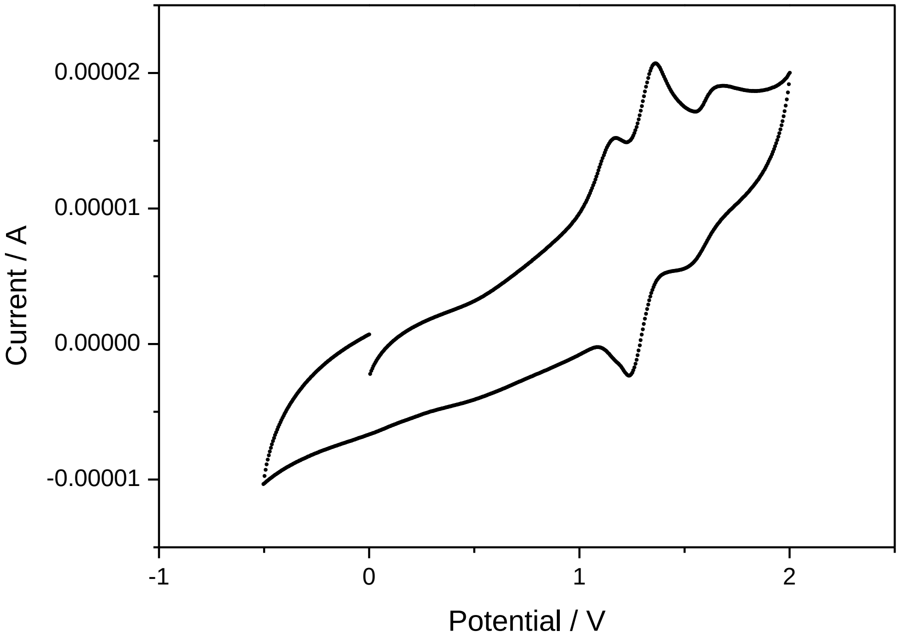

Figure S5 – Cyclic voltammogram of (^{i}Pr)$_3$SiSH and K_2CO_3 (1:2) in MeCN under argon. The reversible peaks at 1. 53 and 1.66 V show the oxidation of (^{i}Pr)$_3$SiS$^-$ and correspond to a potential of 0.67 V *vs* SCE; the reversible peaks at 1.24 and 1.36 V correspond to ferrocene, which was used as an internal standard.

5.4. Radical-radical homocoupling (support for C–H abstraction by activated HAT catalyst)

After the oxidation of the deprotonated HAT species (($(^iPr)_3SiS^-$) by the excited photocatalyst, the activated HAT catalyst (($(^iPr)_3SiS$•) is proposed to abstract a hydrogen atom from the ethyl benzene derivative yielding the corresponding benzyl radical **1**•. The presence of **1f**• is supported by the detection of the resulting radical-radical homocoupling product of 4-ethylanisol (**9**) by GC/MS and by NMR during the isolation of product **6c** (figure S6).

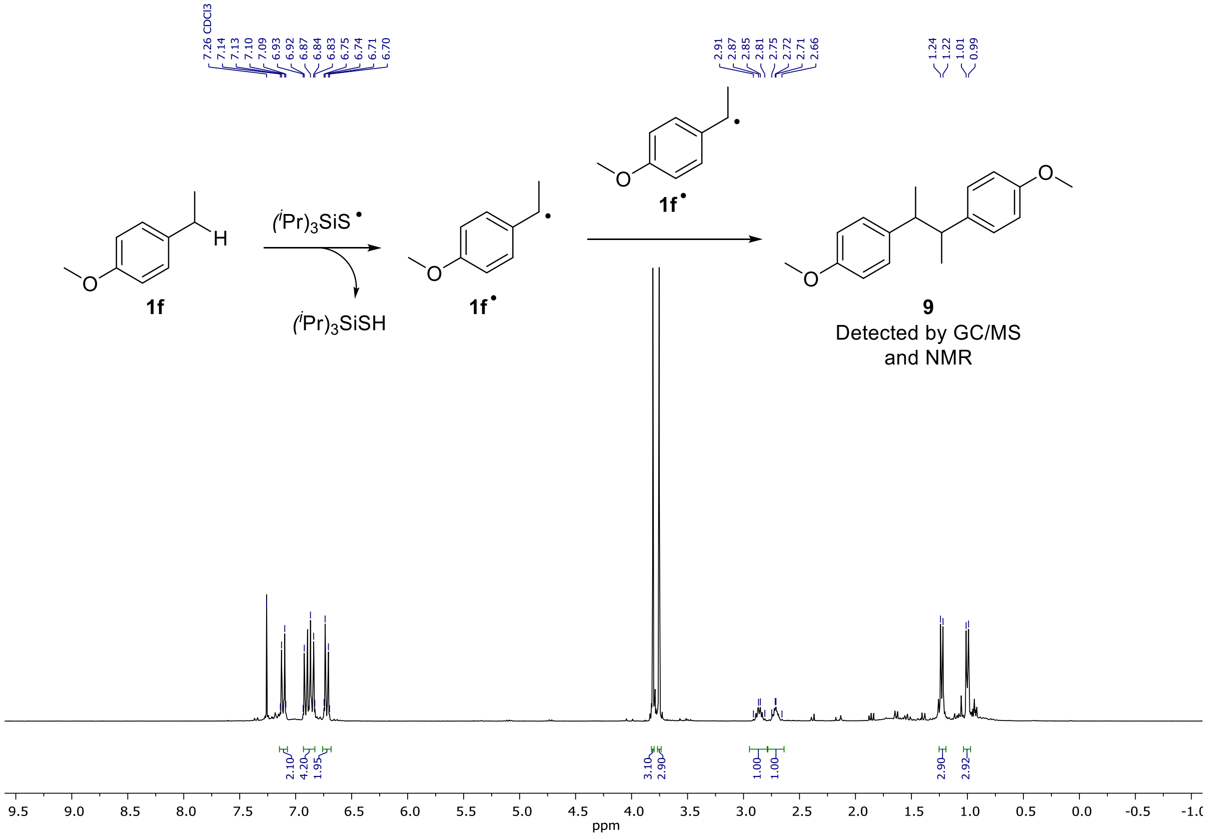

Figure S6 – Formation of radical-radical homocoupling product **9** and crude NMR of **9** obtained during isolation of **6c**.

5.5. Intramolecular ring closure using an ester as an electrophile (support for carbanion formation)

The formation of a reactive carbanion is the key step of the proposed reaction mechanism. The carbanion is proposed to be generated by the SET reduction of carbon radical **1**$^{\bullet}$ by the reduced photocatalyst. The generation of the carbanion intermediate is supported by the successful intramolecular ring closure using esters as electrophiles (scheme 2/S8). As described by Murphy *et al.*,[30] esters are not known to react with radicals, yet are susceptible to an addition by ionic nucleophiles. Hence, Murphy and co-workers designed compound **10** as carbanion testing system, which will give product **11** if a radical intermediate is involved, while product **12** can only be formed if the corresponding carbanion intermediate is generated (scheme S7).

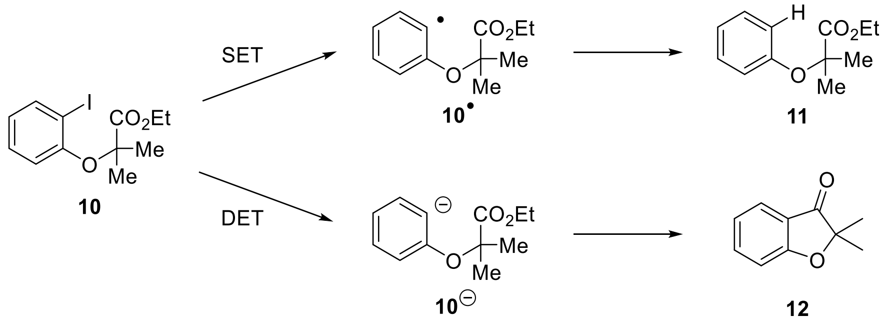

Scheme S1 – Carbanion test system designed by Murphy *et al.*

Compound **7a** (and **7b**) can be regarded as a similar yet simpler carbanion test system. Thus, the formation of cyclization product **8a** (and **8b**) (scheme S8) supports the proposed carbanion intermediate.

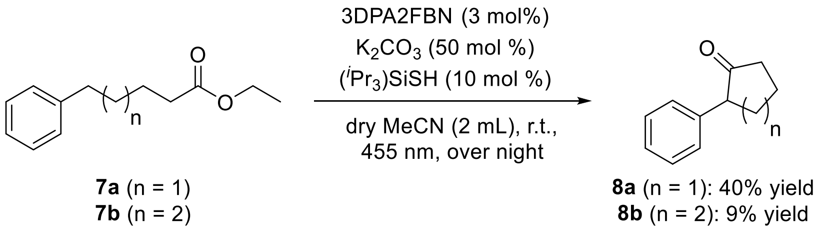

Scheme S2 – Formation of cyclization product **8a** and **8b**.

6. NMR-spectra

4CzIPN, ^{1}H- and ^{13}C-NMR ($CDCl_3$):

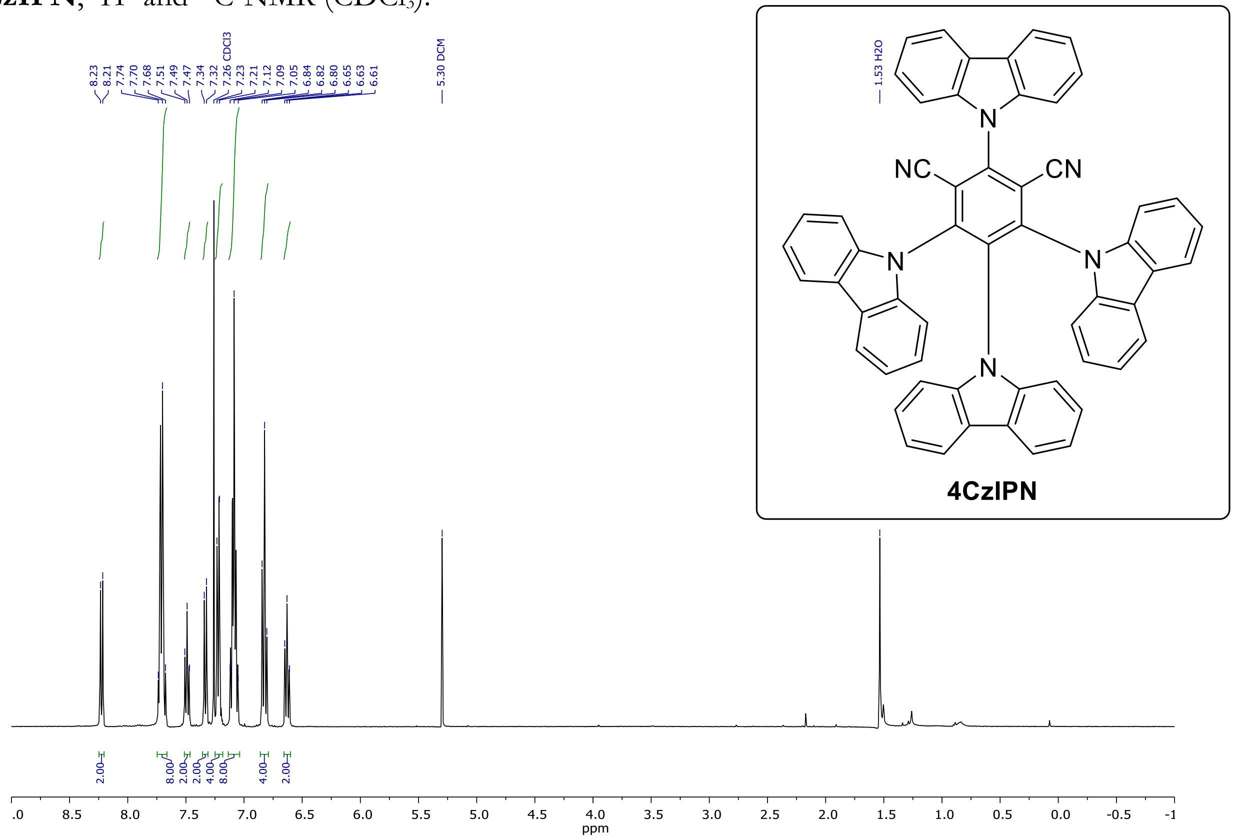

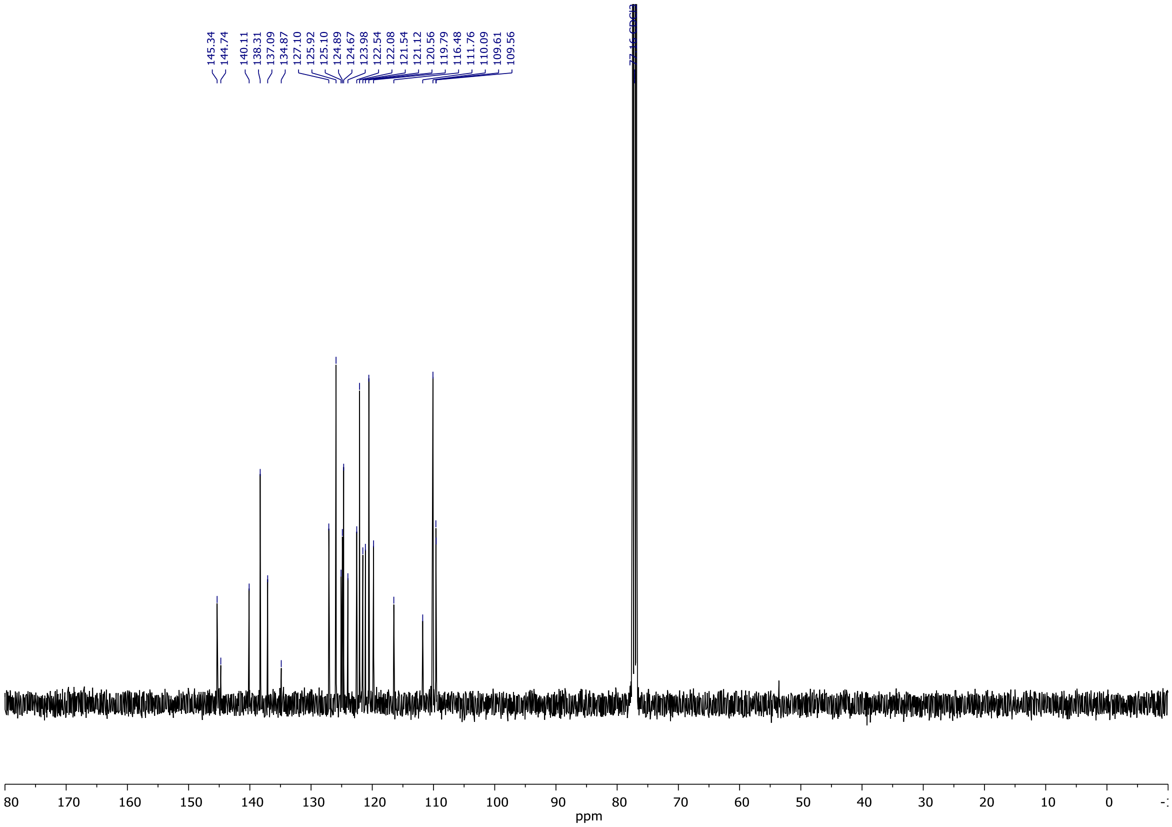

3DPA2FBN, ^{1}H- and ^{19}F-NMR ($CDCl_3$):

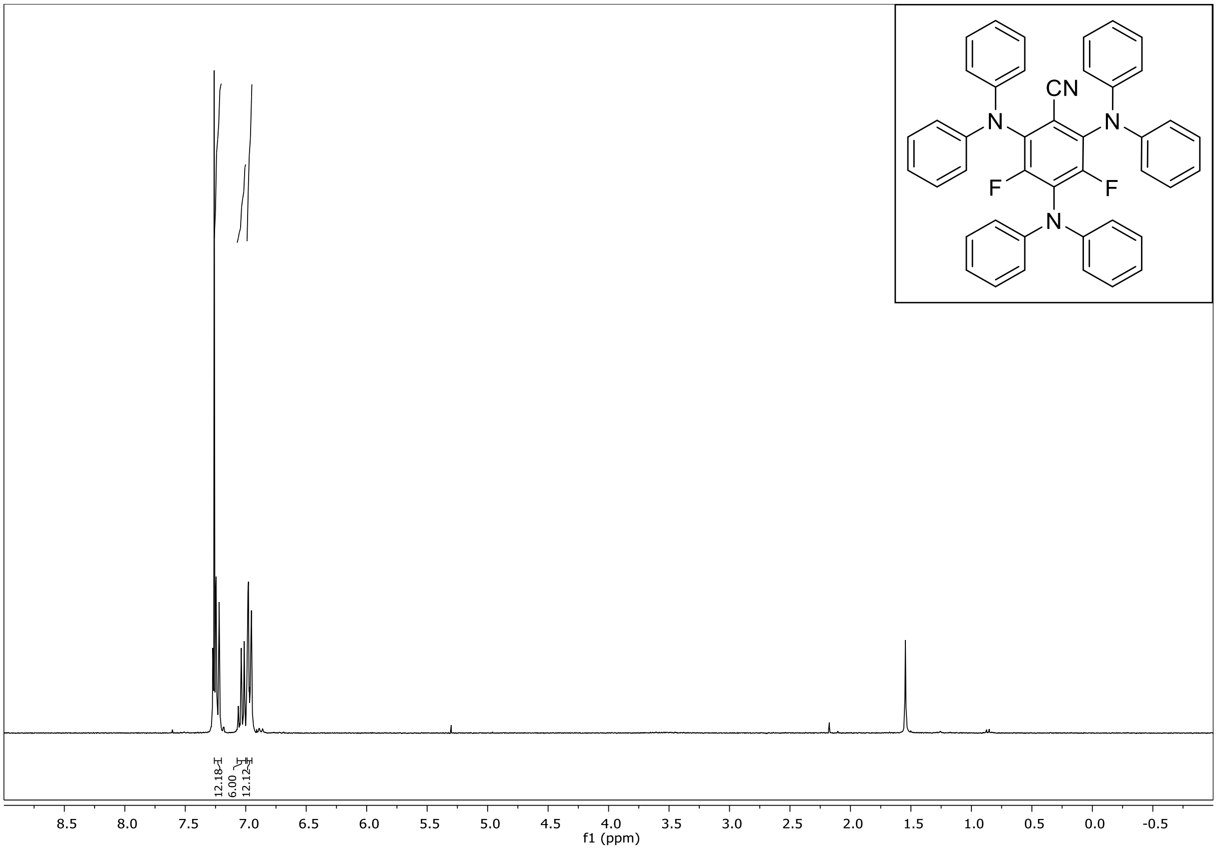

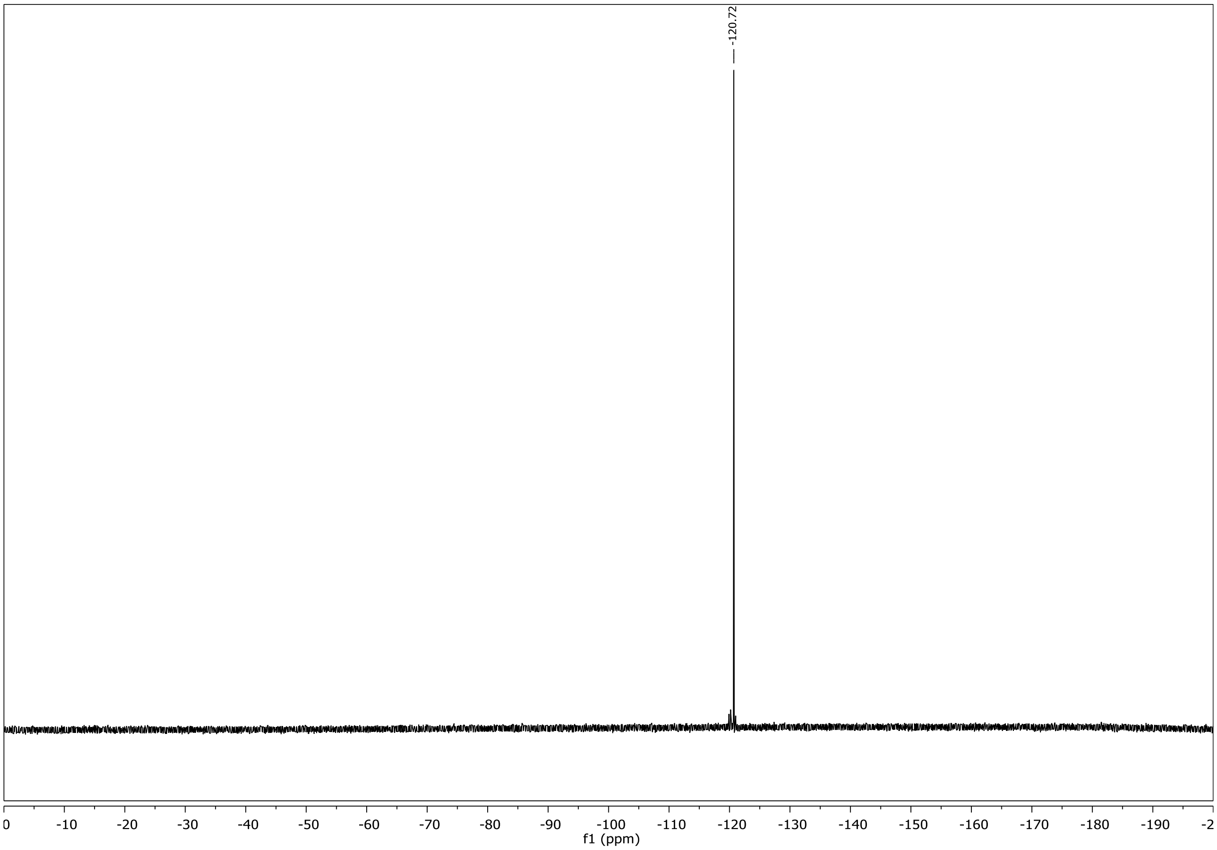

3DPAFIPN, ^{1}H- and ^{19}F-NMR ($CDCl_3$):

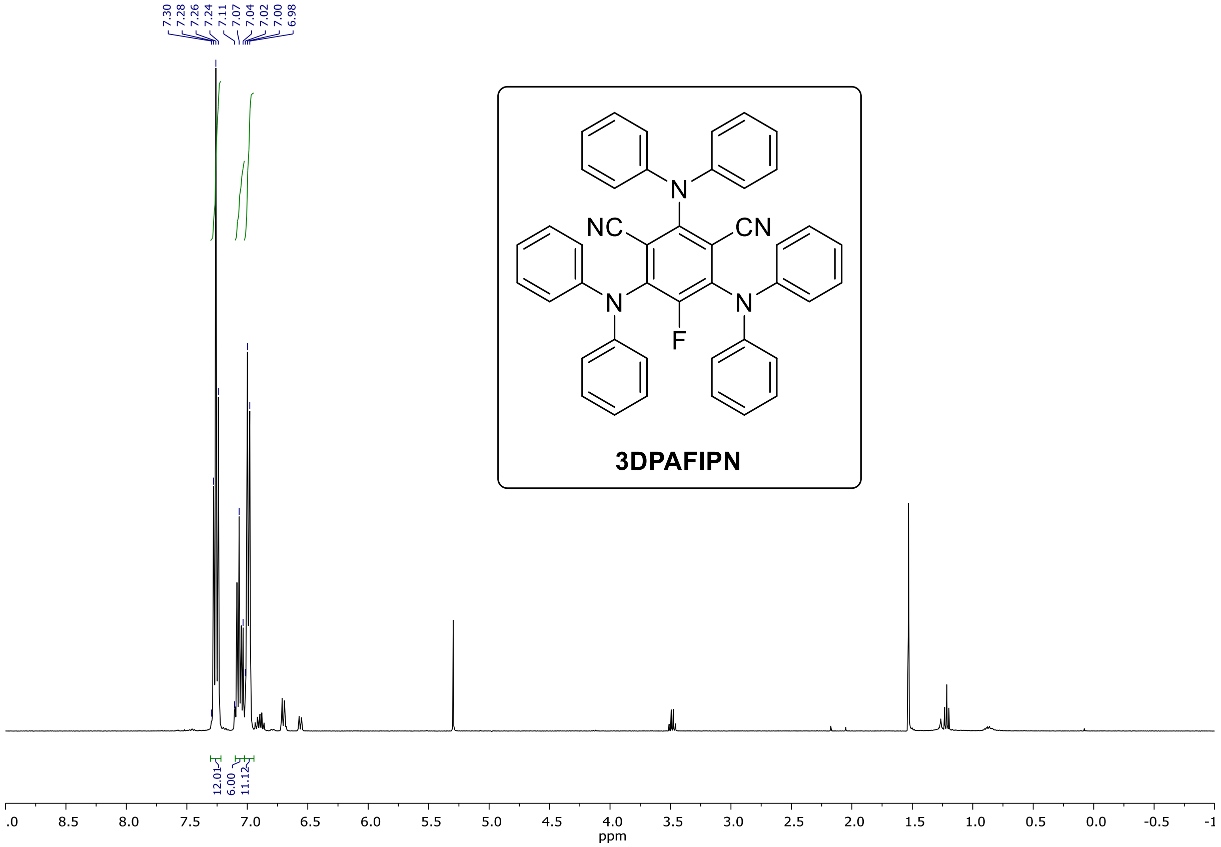

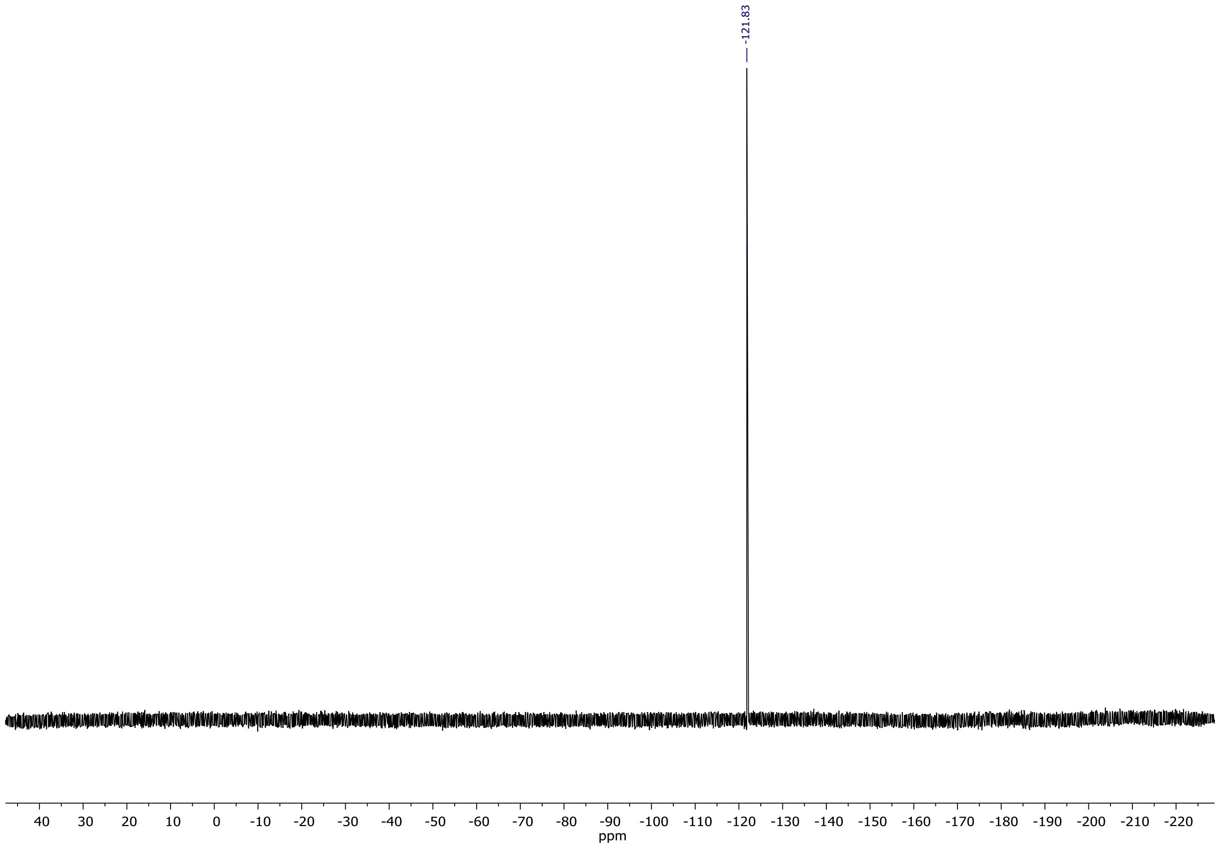

4Cz(*p*H)BN, ^{1}H- and ^{13}C-NMR ($CDCl_3$):

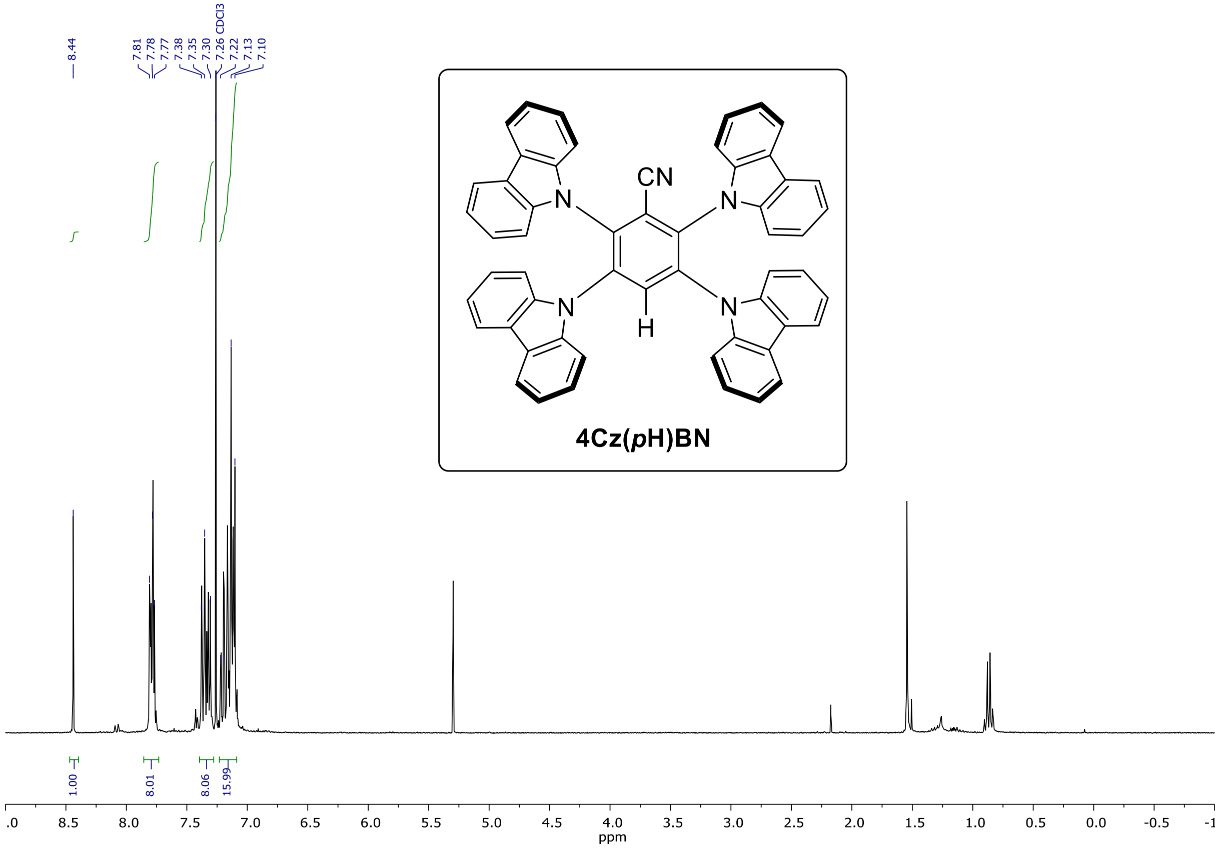

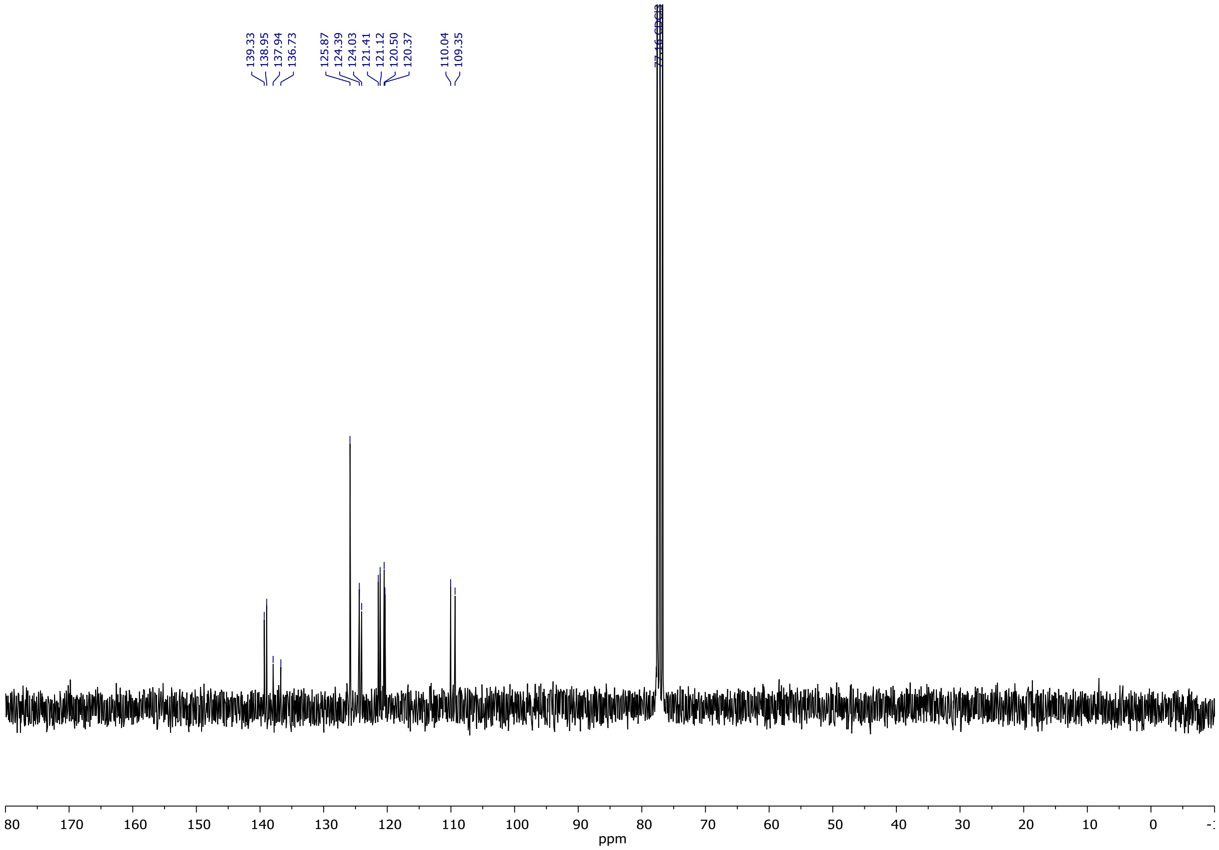

1-Naphthalene-10-phenoxazine, ^{1}H- and ^{13}C-NMR ($CDCl_3$)

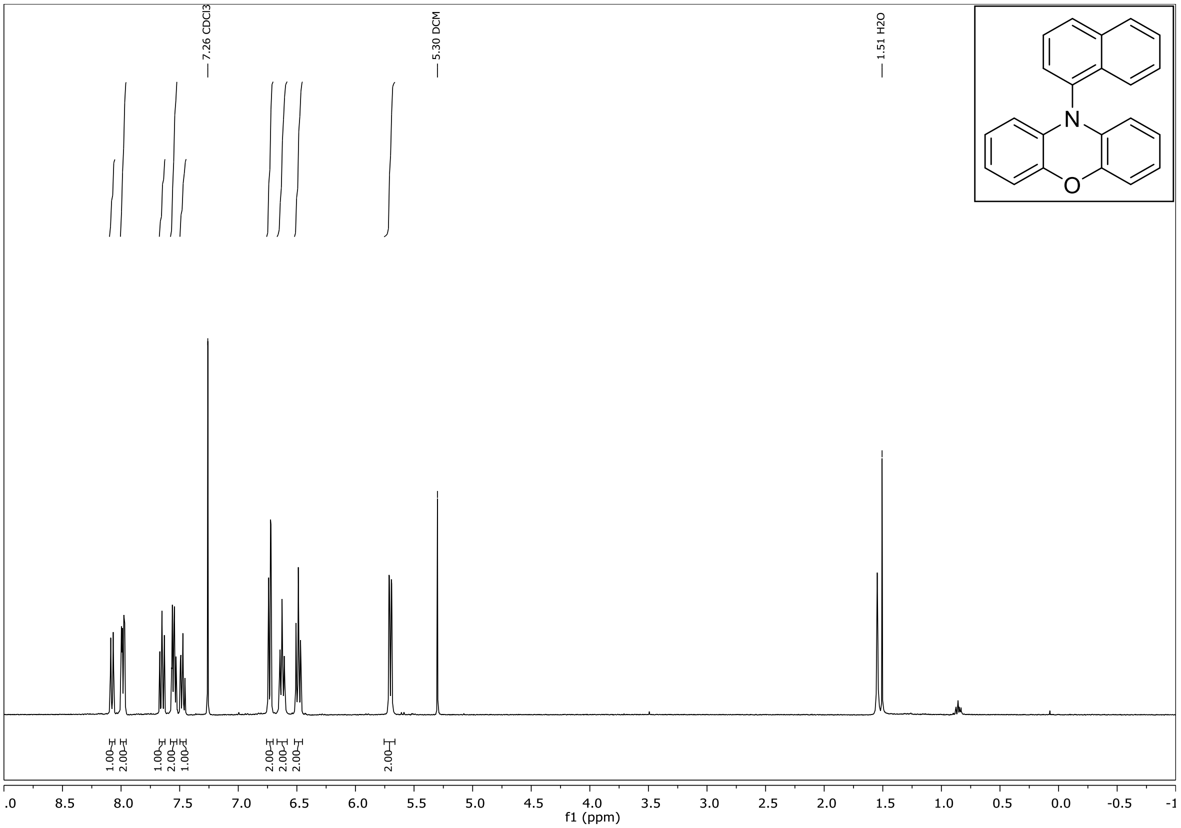

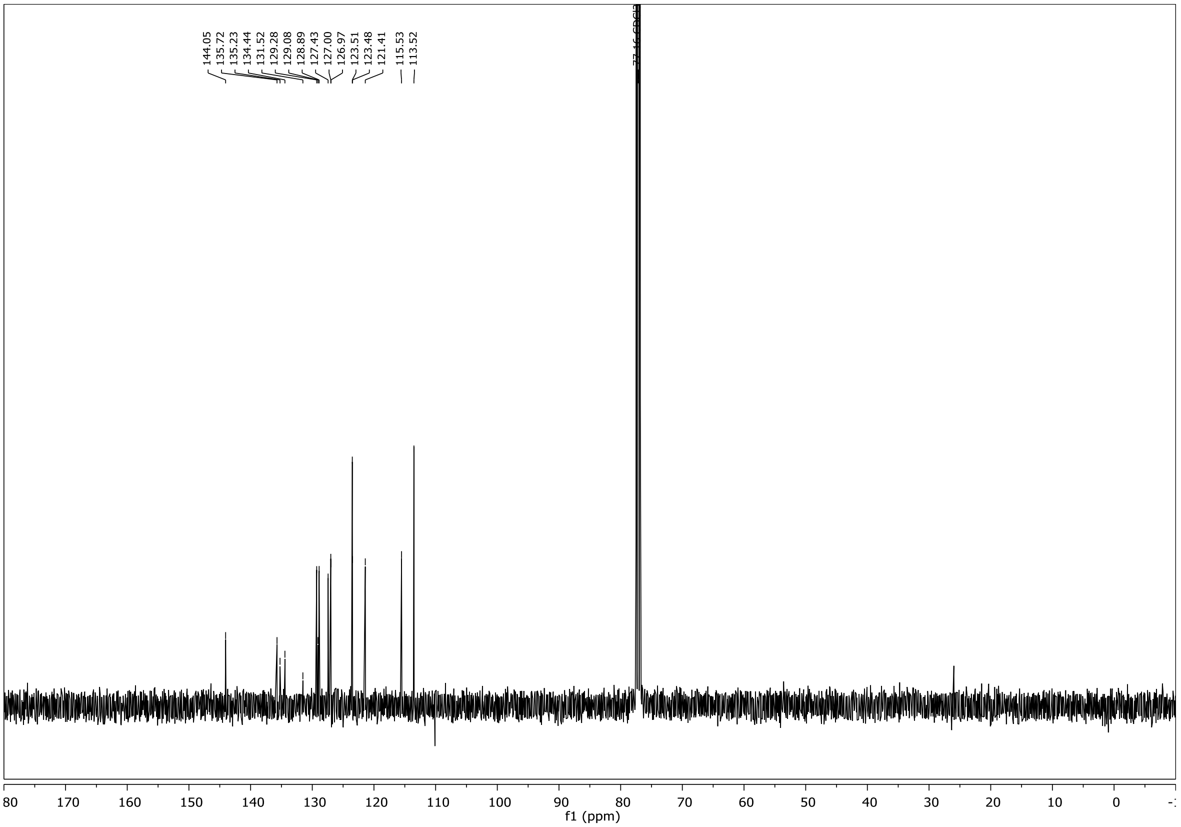

3,7-Dibromo 1-naphthalene-10-phenoxazine, ^{1}H- and ^{13}C-NMR (C_6D_6)

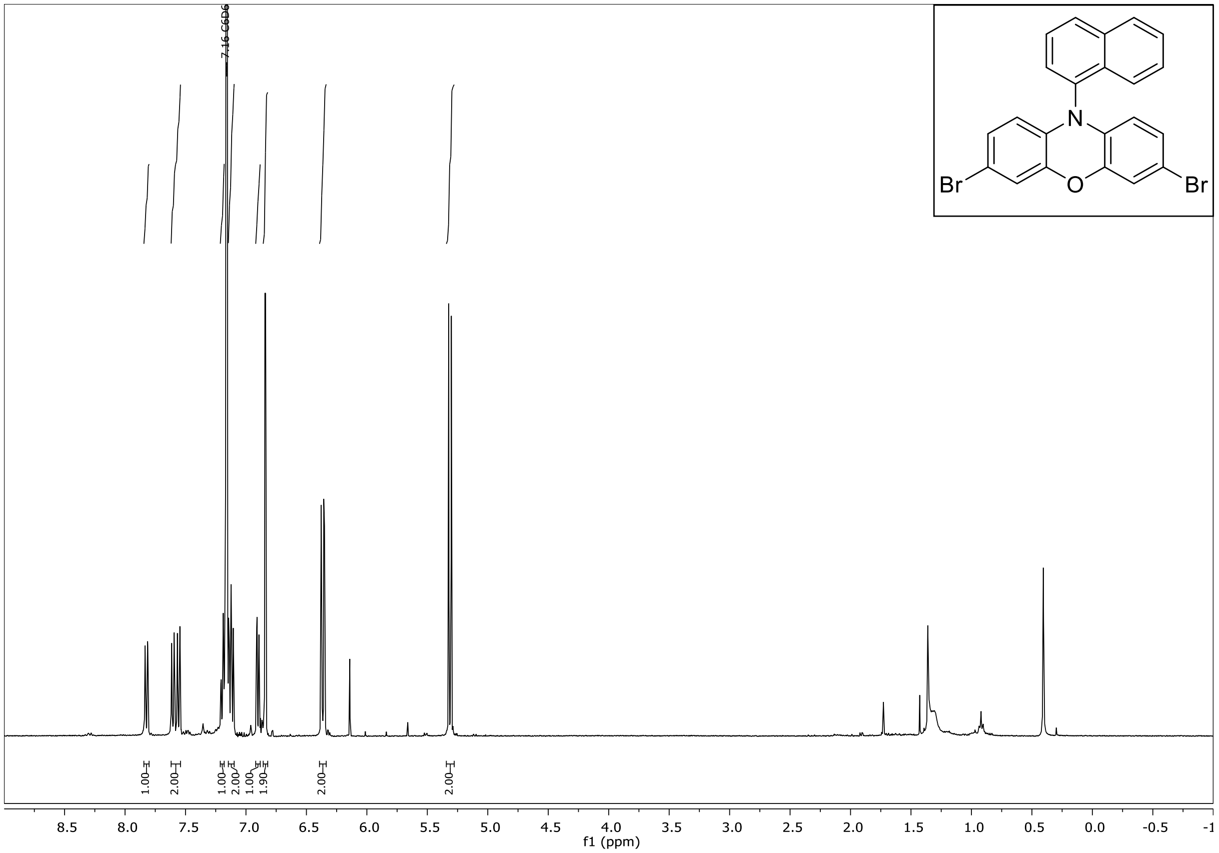

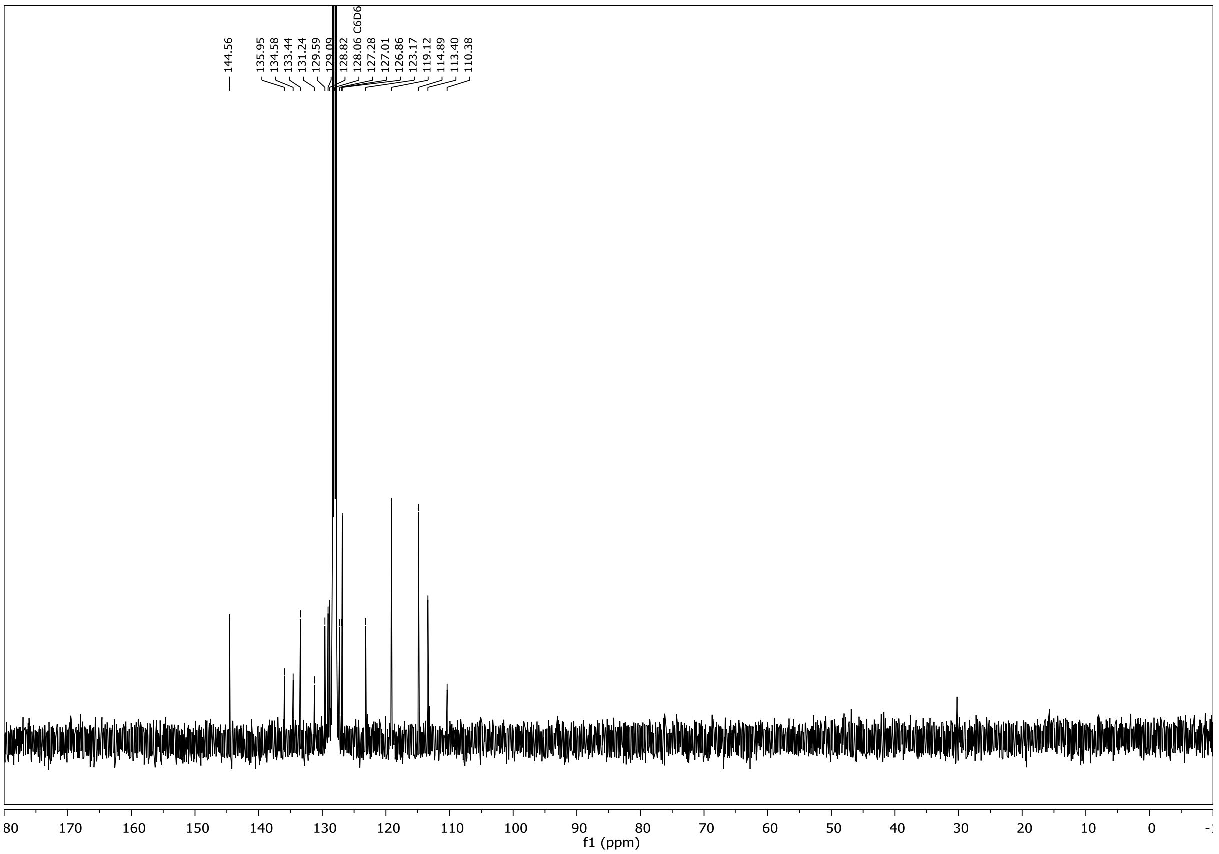

3,7-Di(4-biphenyl) 1-naphthalene-10-phenoxazine, ^{1}H-NMR (DMSO-d_6)

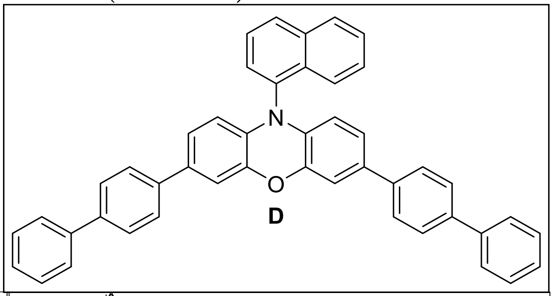

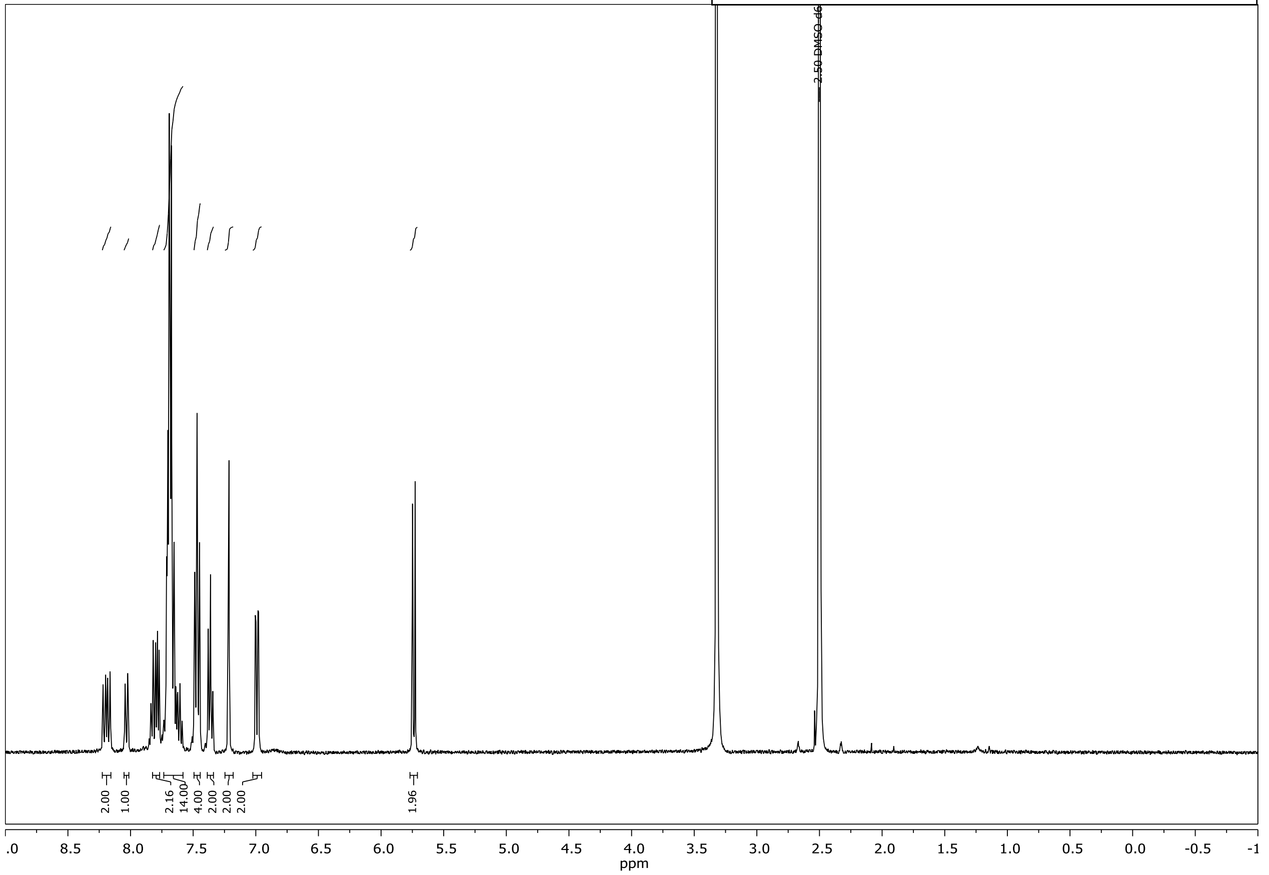

***N*-(4-Ethylphenyl)acetamide**, ^{1}H- and ^{13}C-NMR ($CDCl_3$)

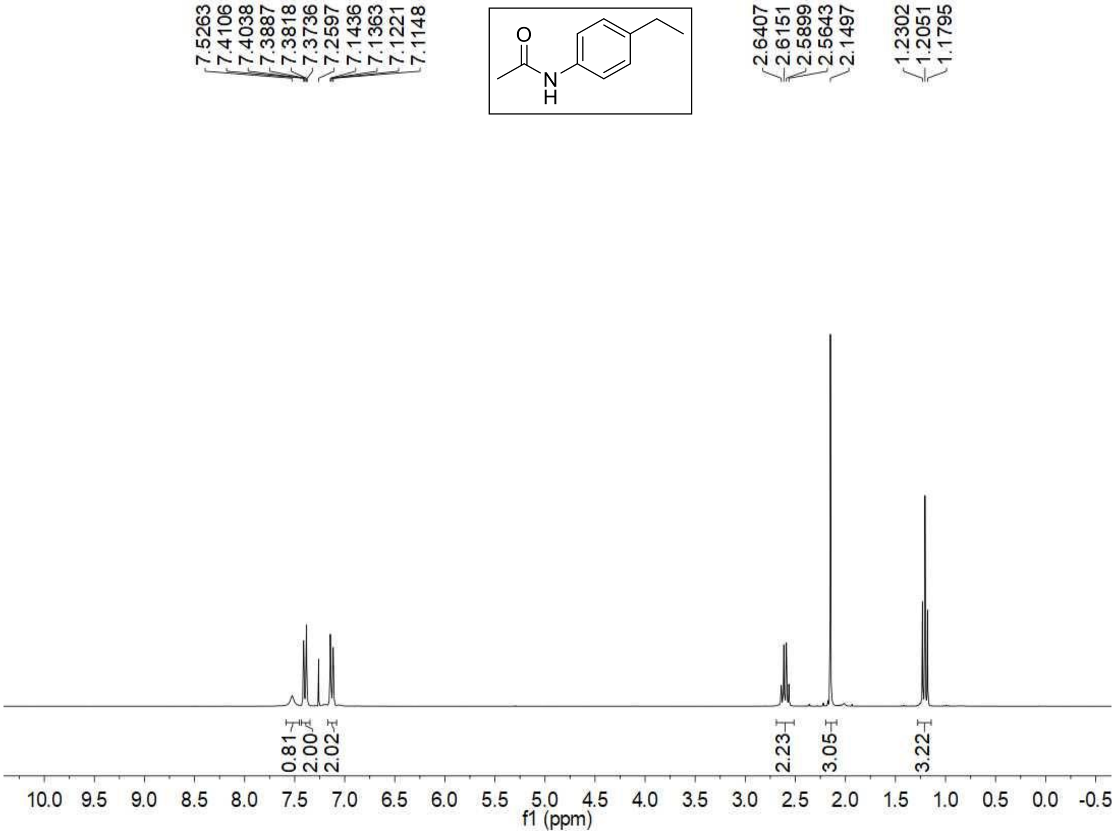

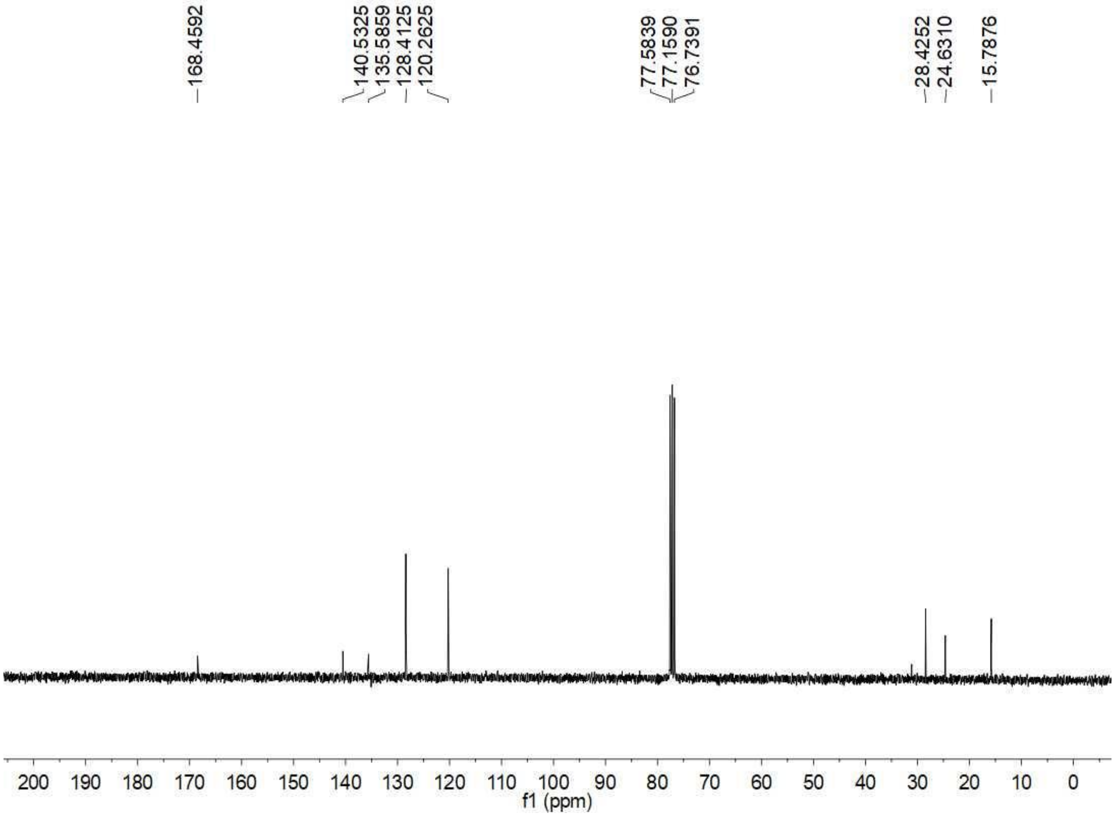

***N*-(3-Oxobutyl)-*p*-toluenesulfonamide**, ^{1}H-NMR ($CDCl_3$)

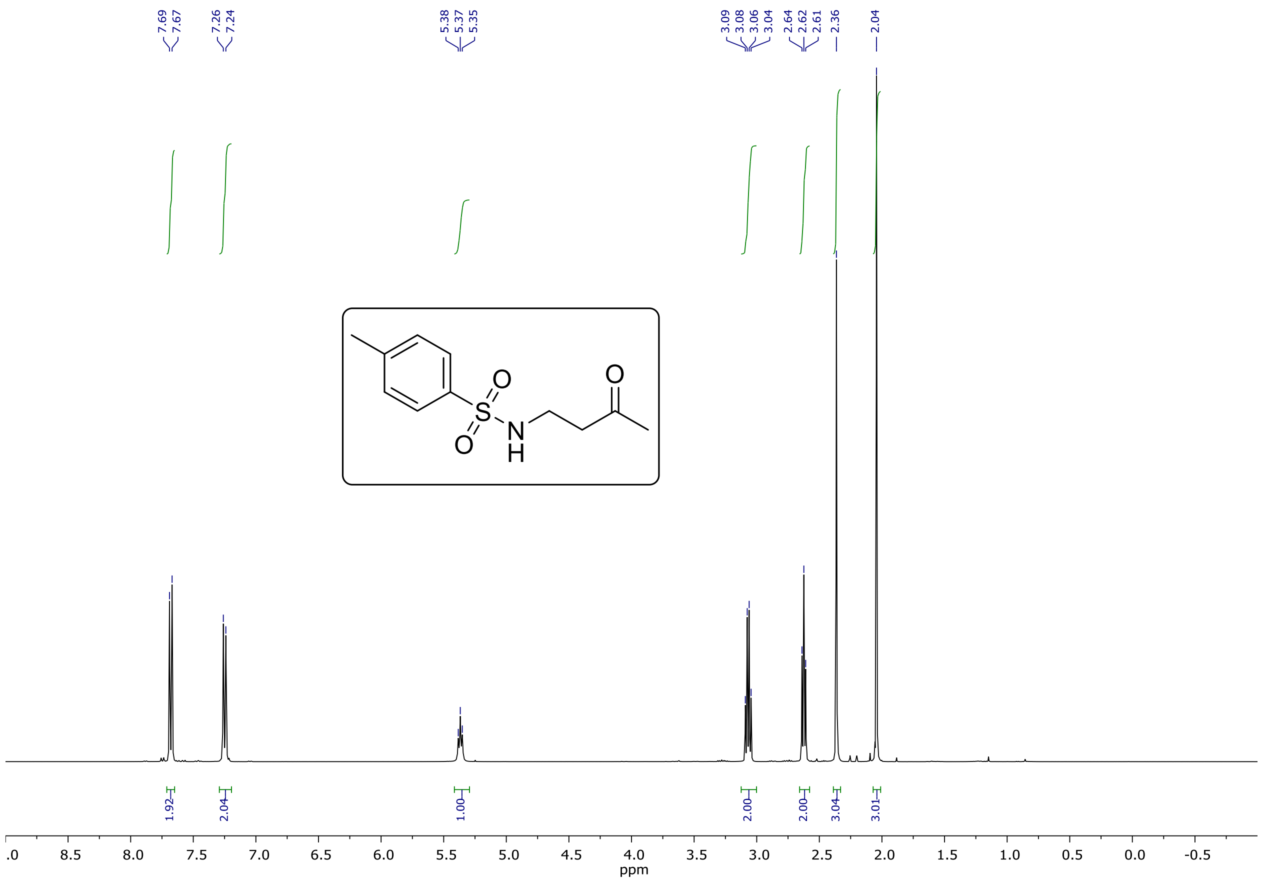

Ethyl-5-phenylpentanoate (7a), ^{1}H- and ^{13}C-NMR ($CDCl_3$)

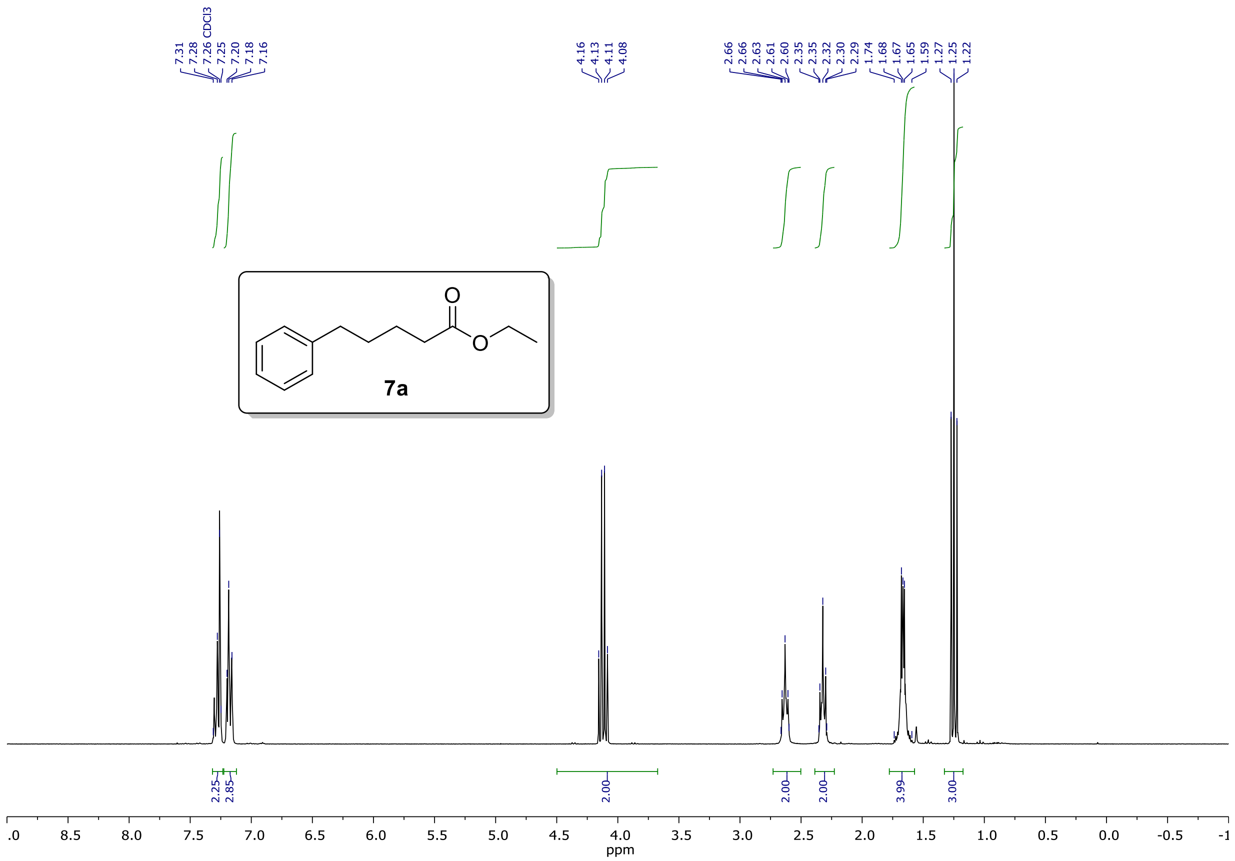

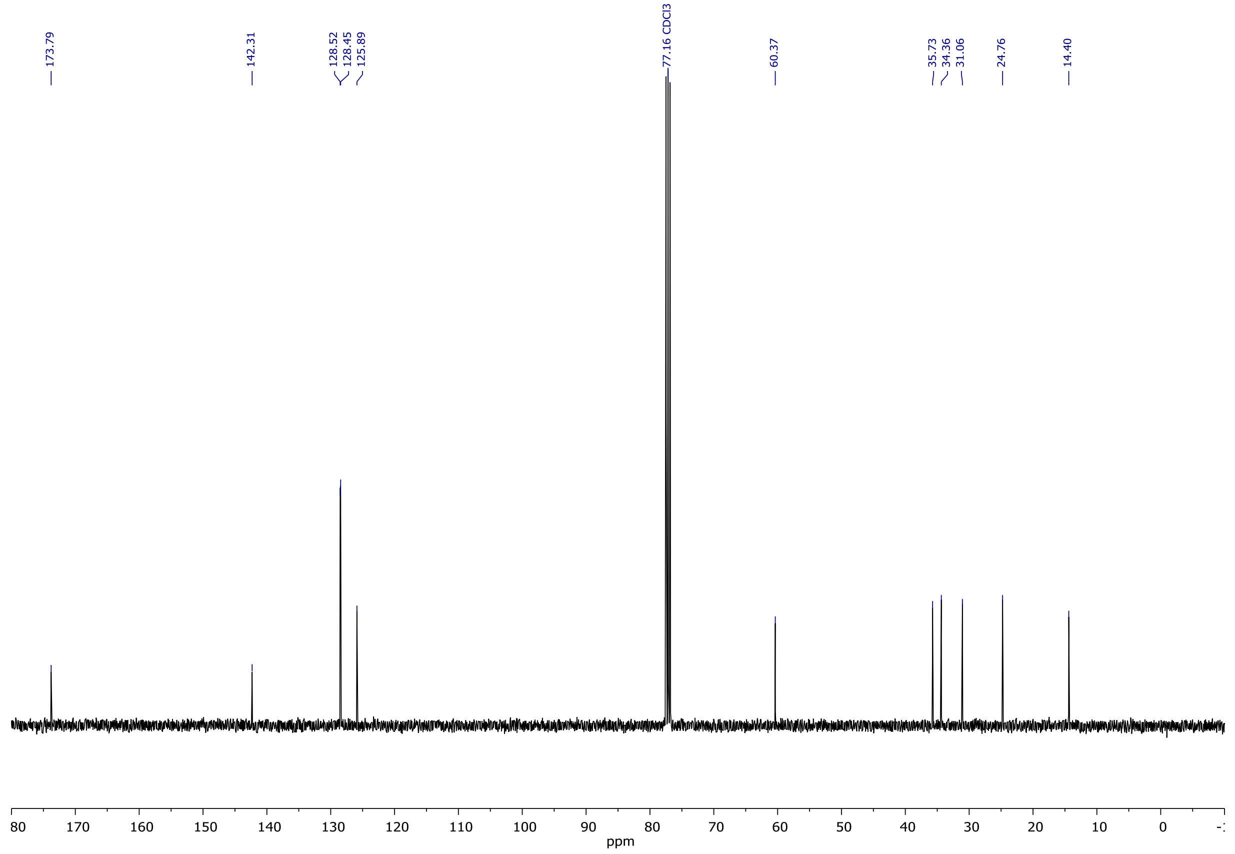

Ethyl-6-phenylhexanoate (7b), ^{1}H- and ^{13}C-NMR ($CDCl_3$)

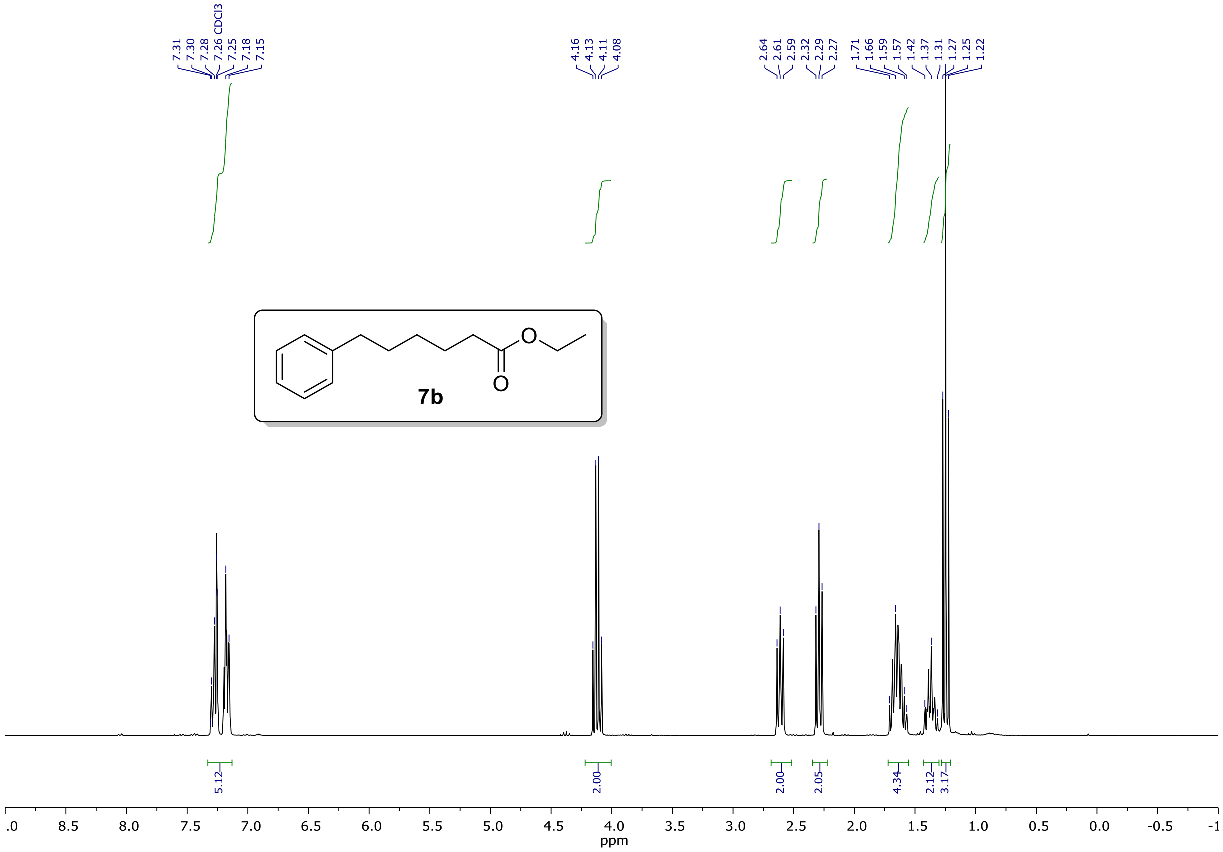

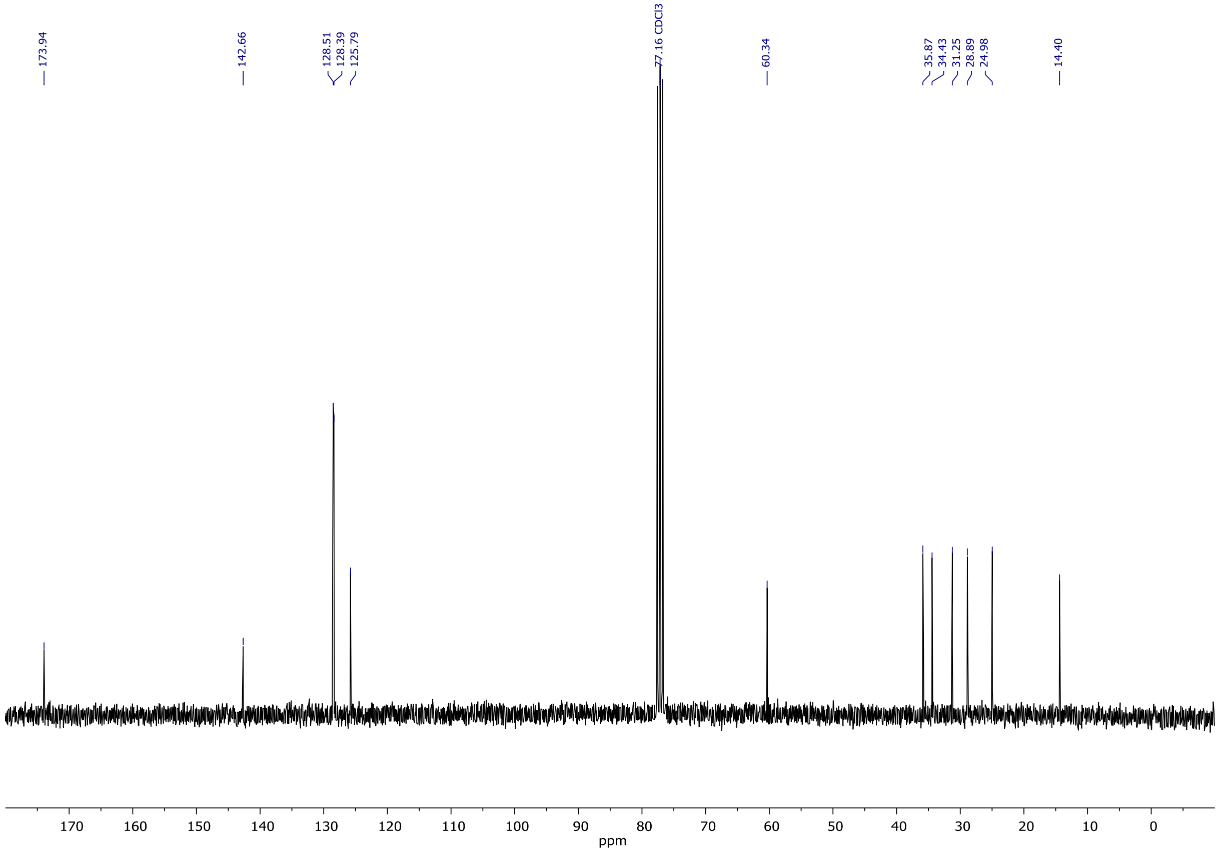

Compound **3a**, ^{1}H- and ^{13}C-NMR ($CDCl_3$)

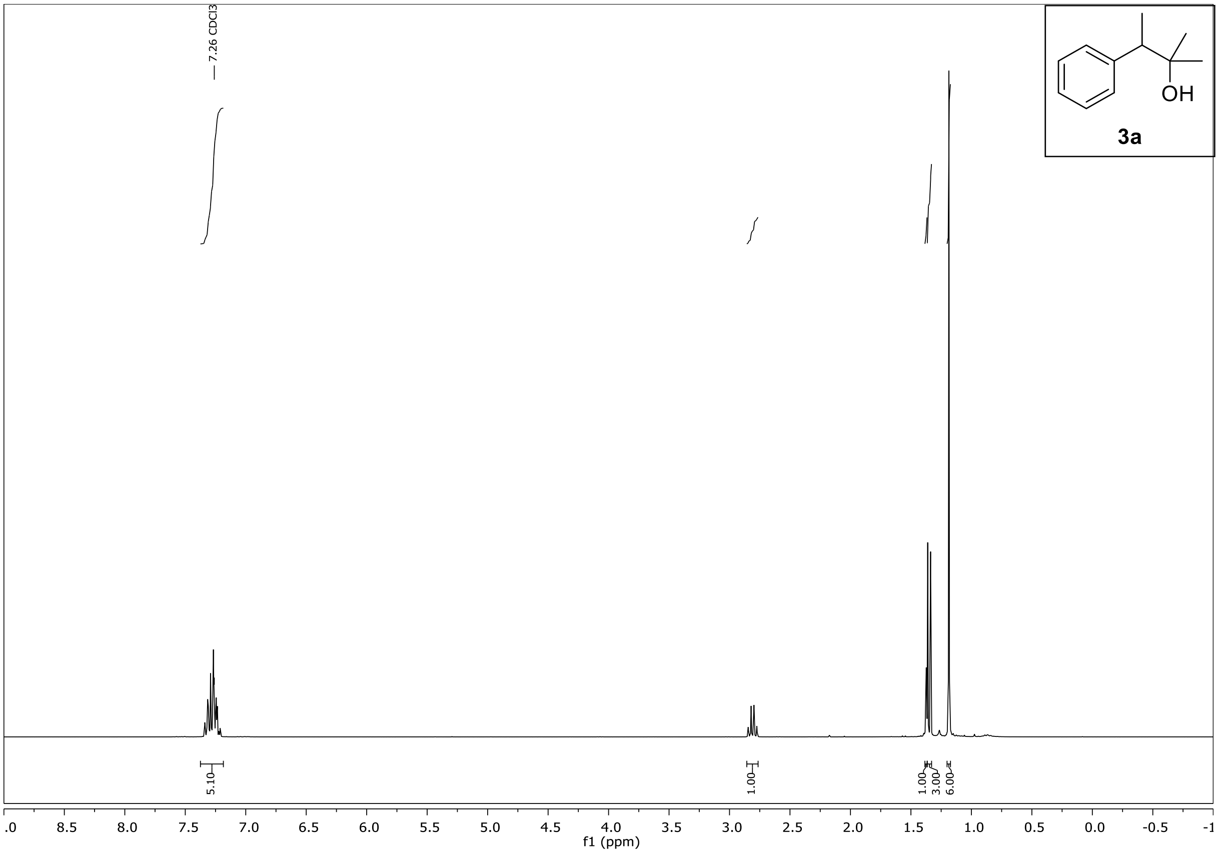

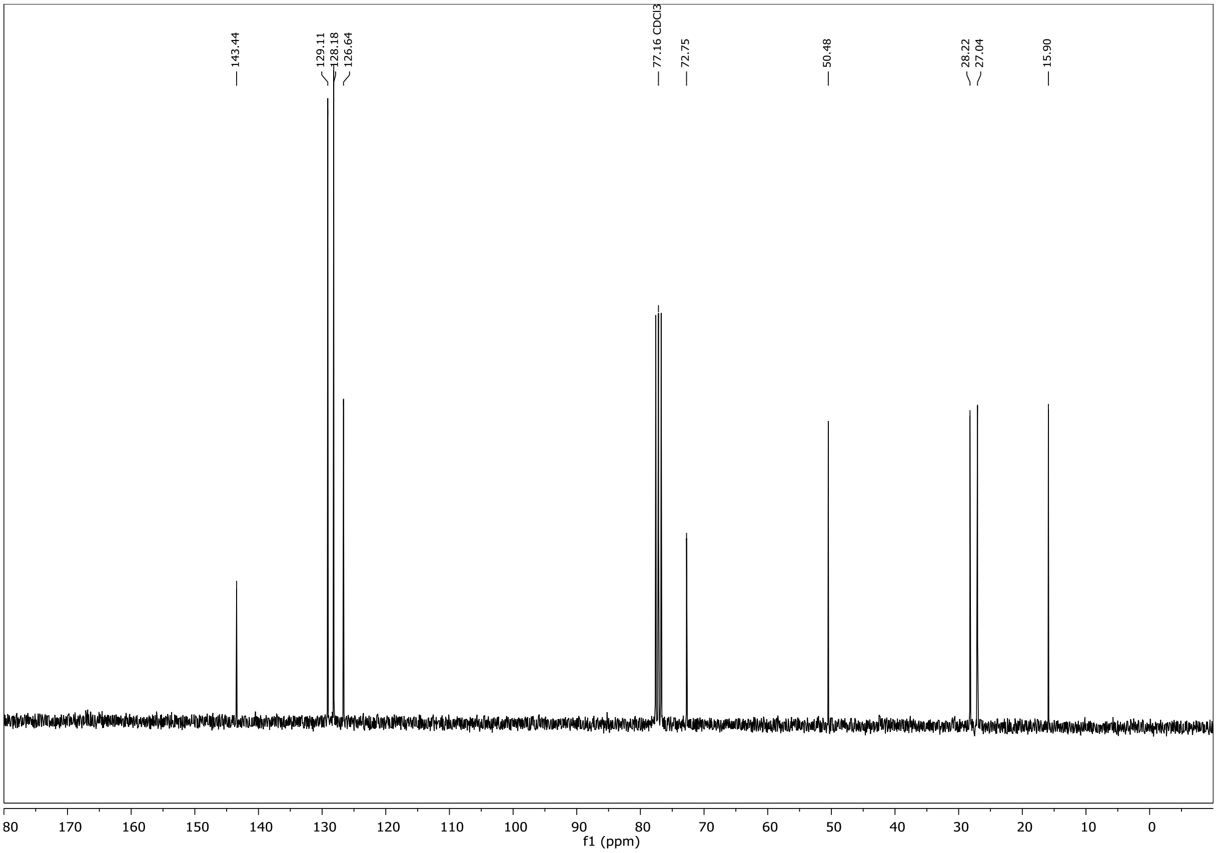

Compound **3b**, ^{1}H- and ^{13}C-NMR ($CDCl_3$)

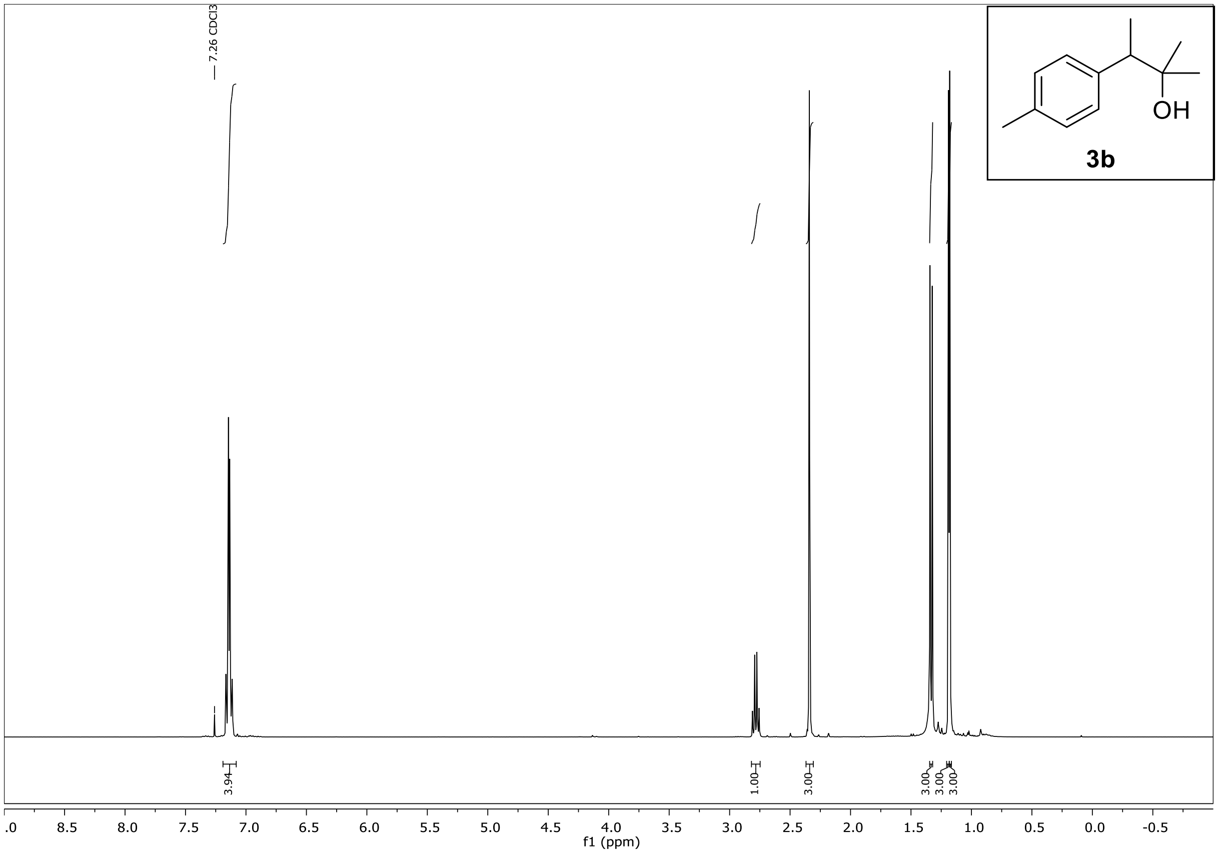

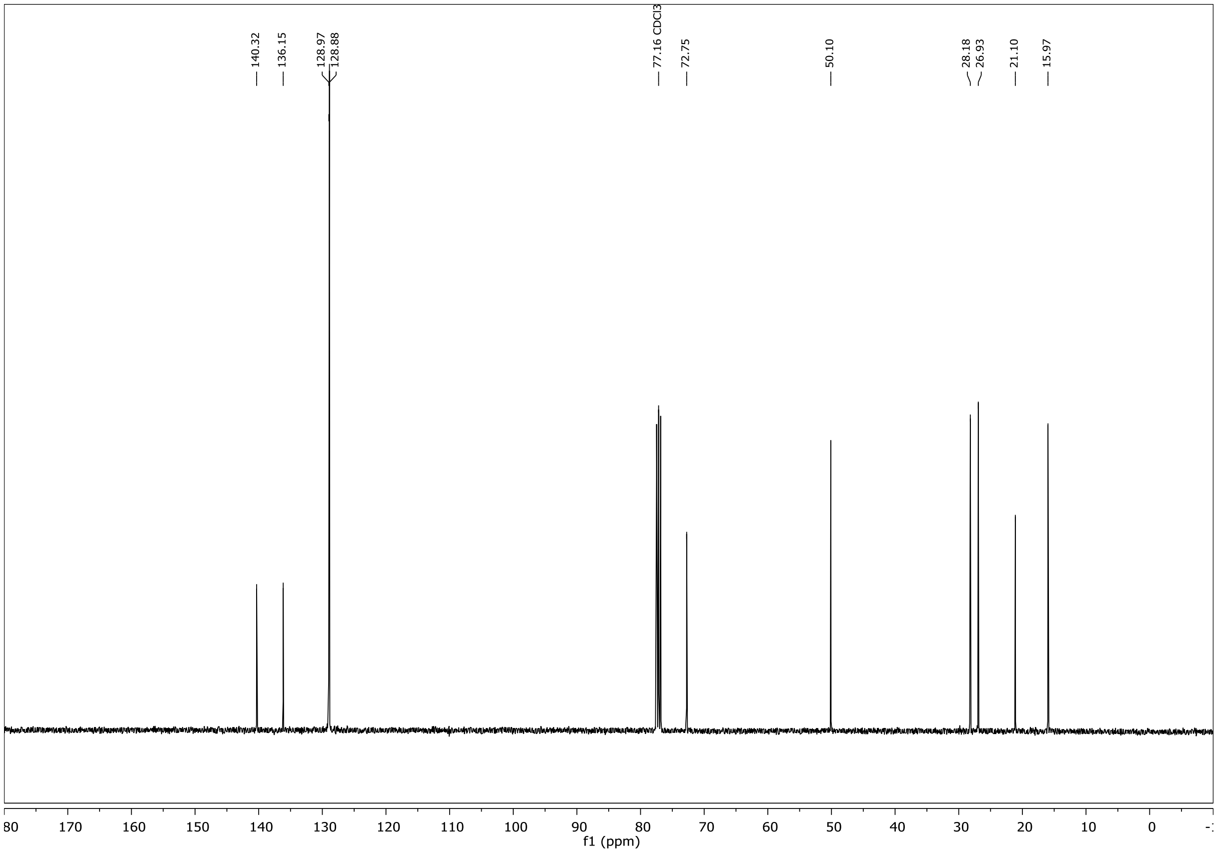

Compound **3c**, ^{1}H- and ^{13}C-NMR ($CDCl_3$)

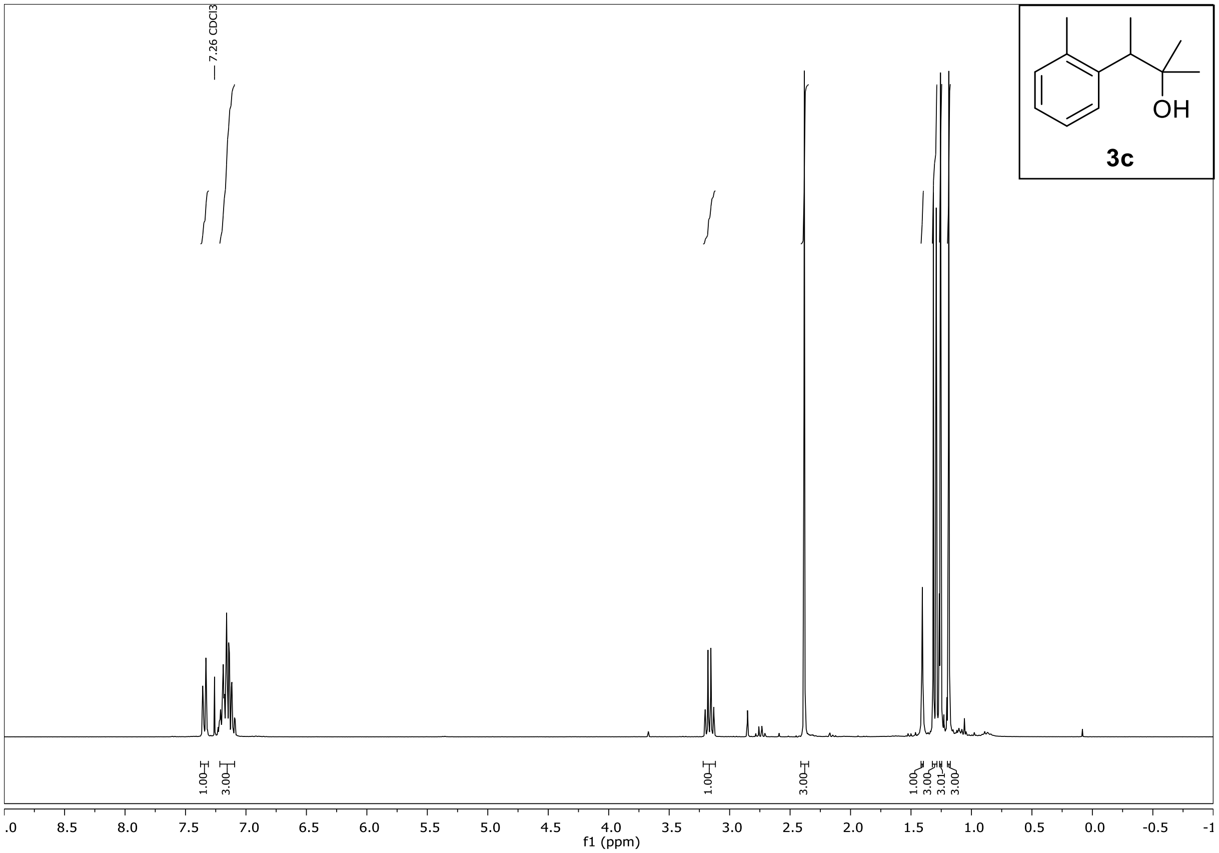

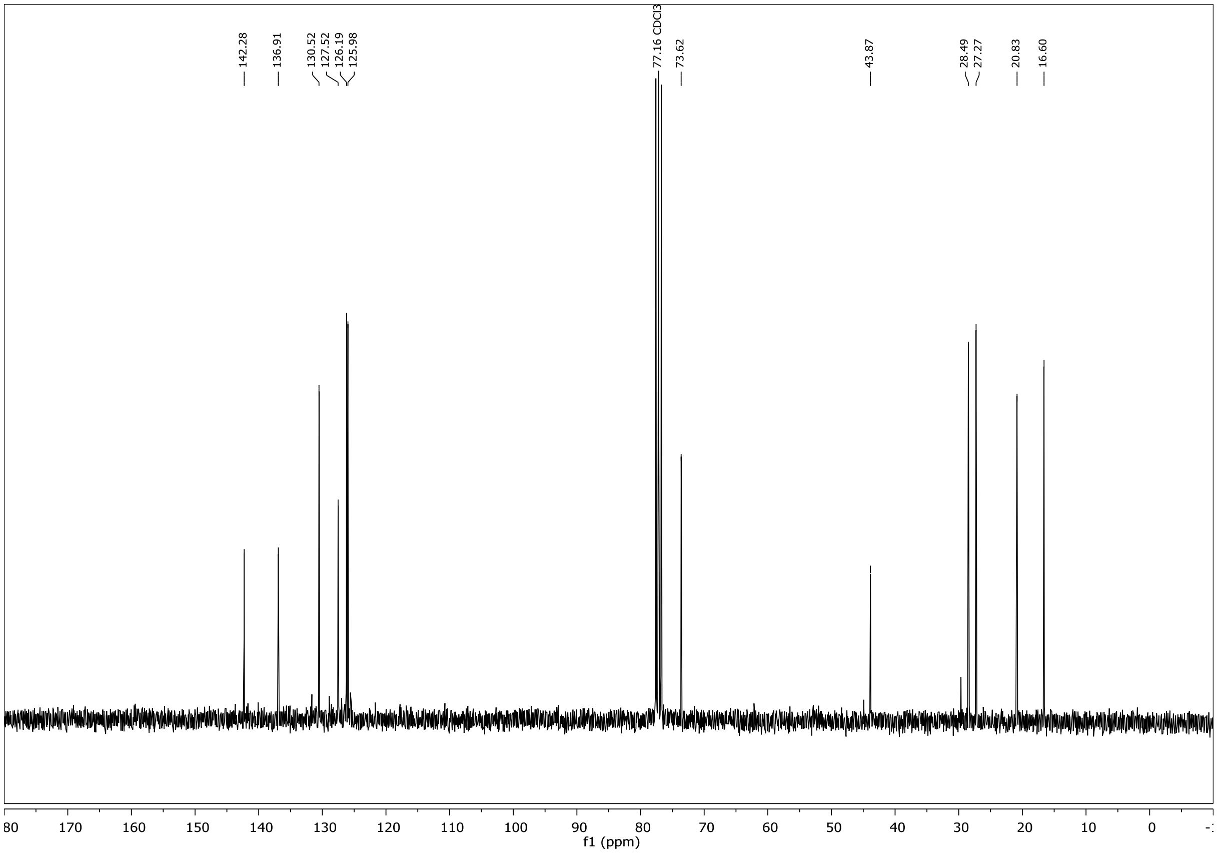

Compound **3d**, ^{1}H- and ^{13}C-NMR ($CDCl_3$)

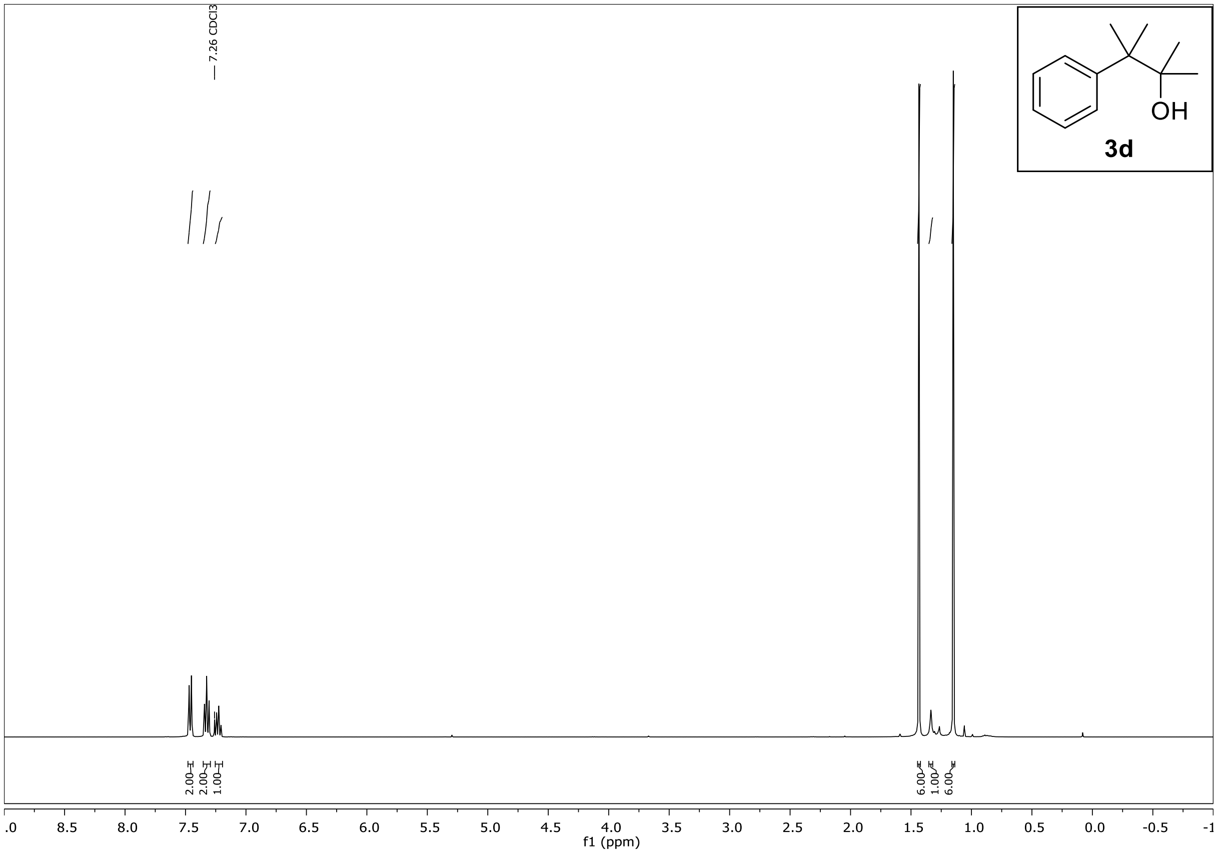

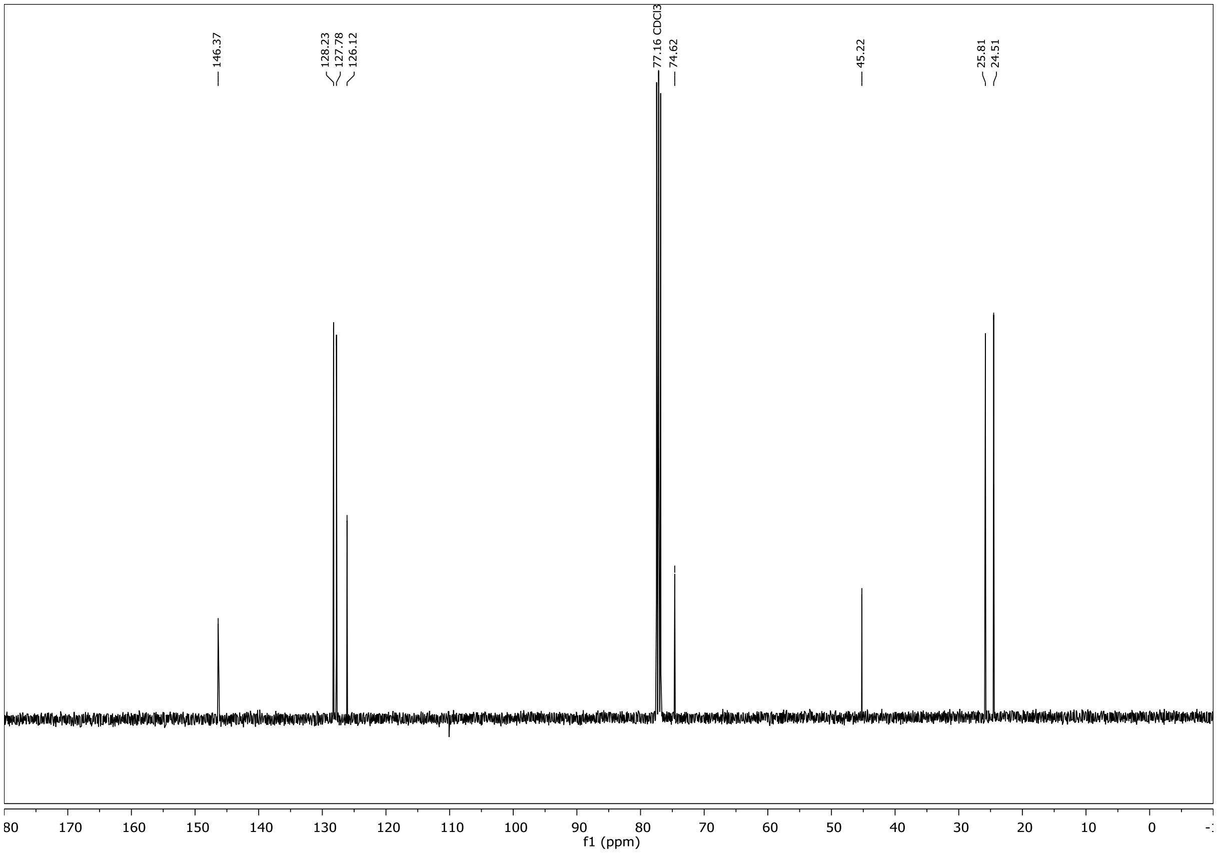

Compound **3e**, ^{1}H- and ^{13}C-NMR ($CDCl_3$)

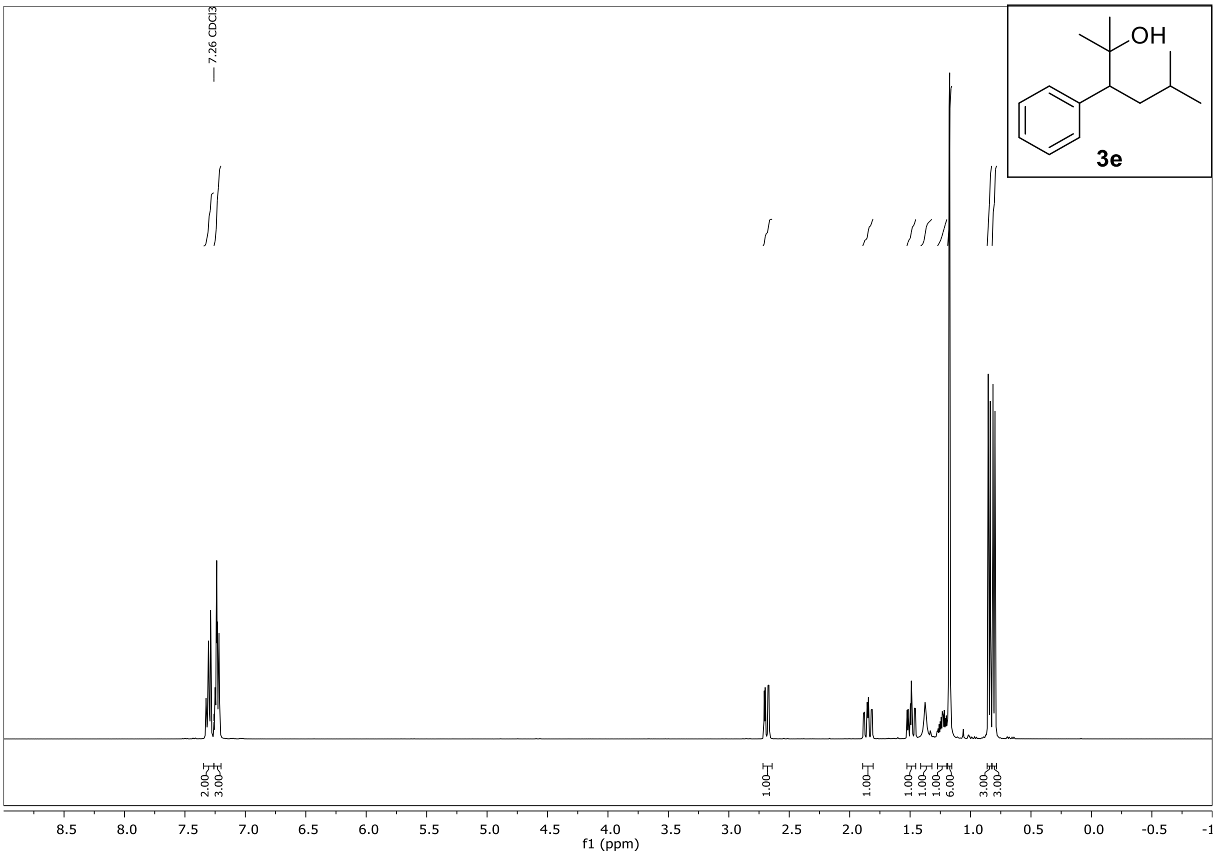

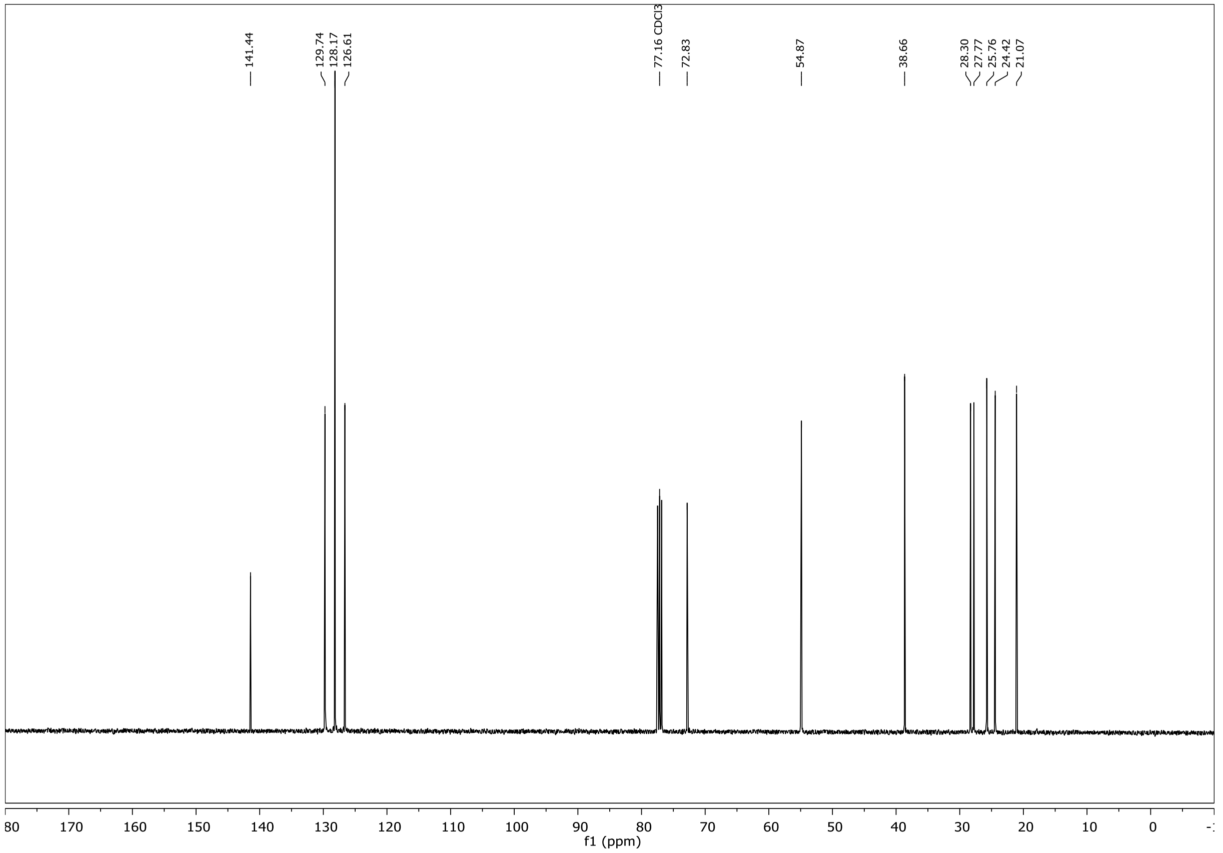

Compound **3f**, ^{1}H- and ^{13}C-NMR ($CDCl_3$)

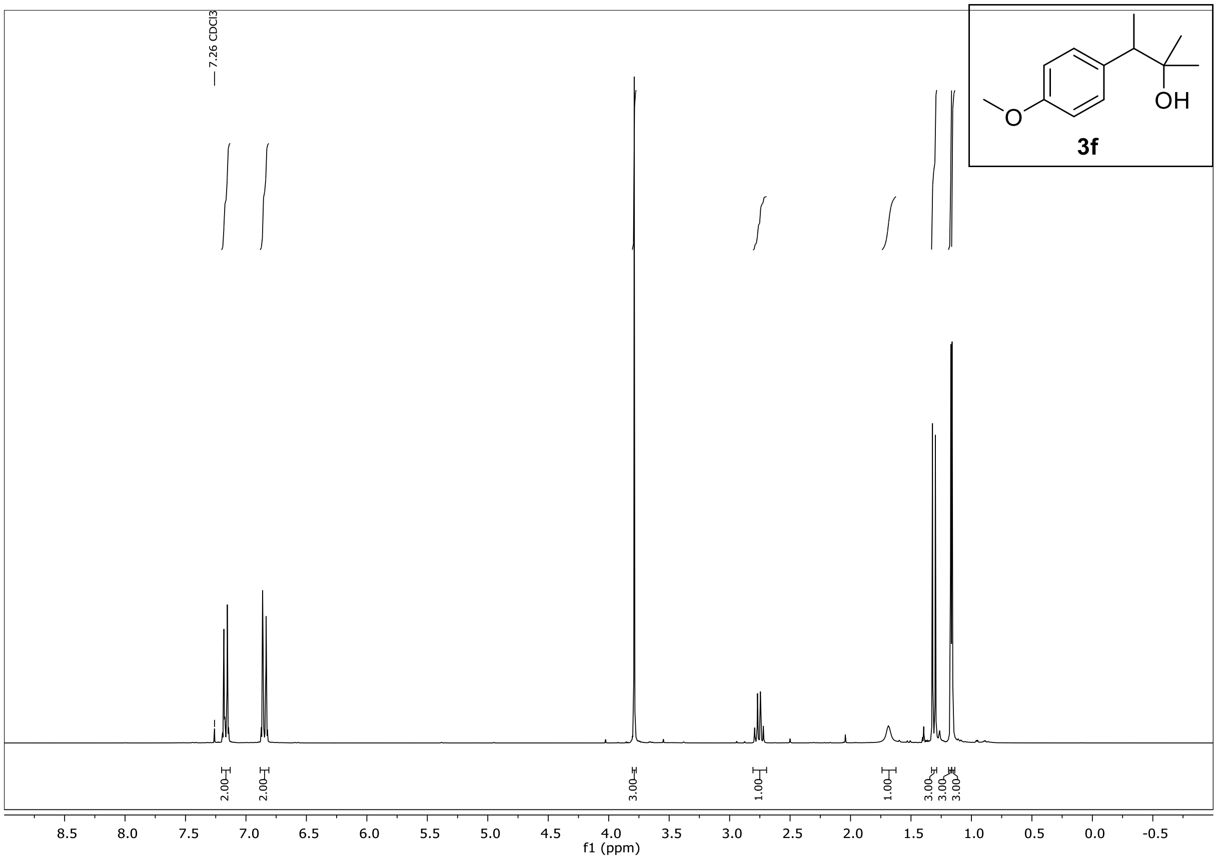

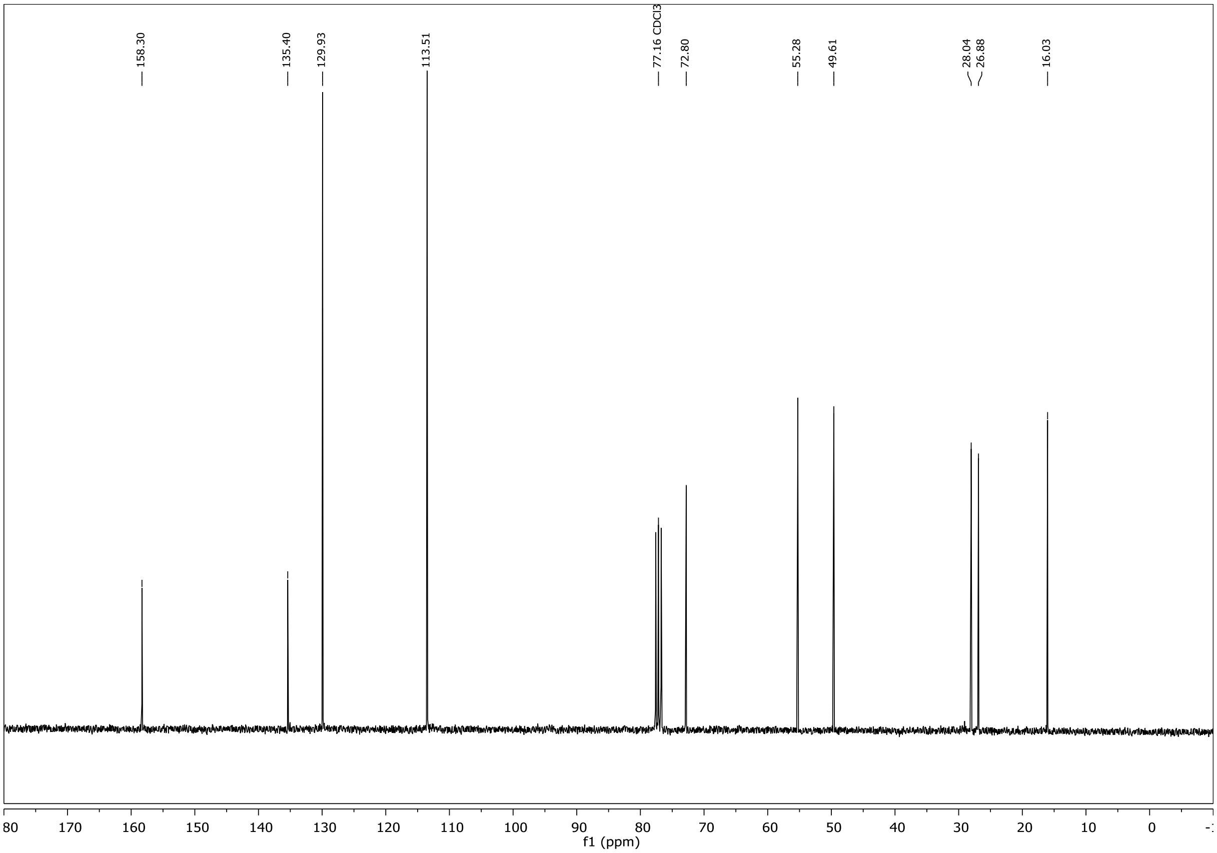

Compound **3g**, ^{1}H- and ^{13}C-NMR ($CDCl_3$)

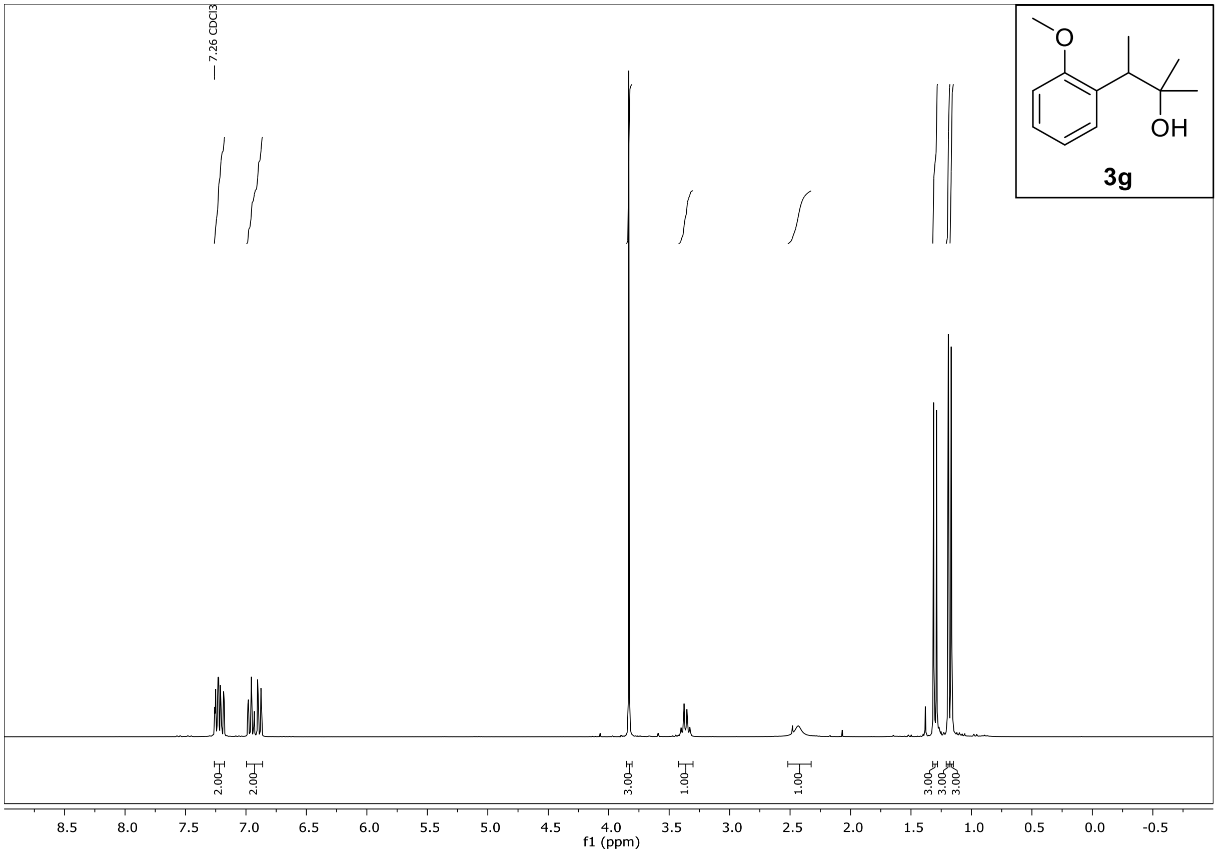

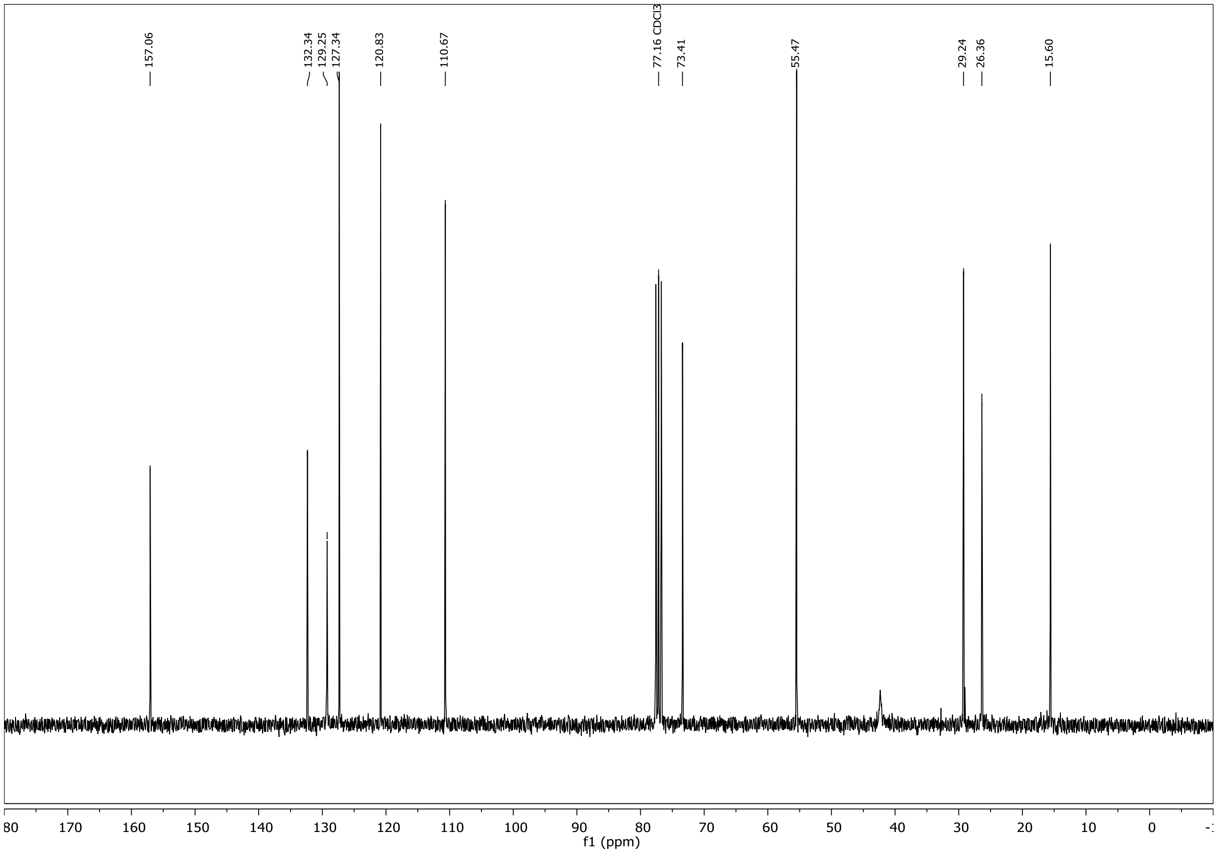

Compound **3h**, ^{1}H- and ^{13}C-NMR ($CDCl_3$)

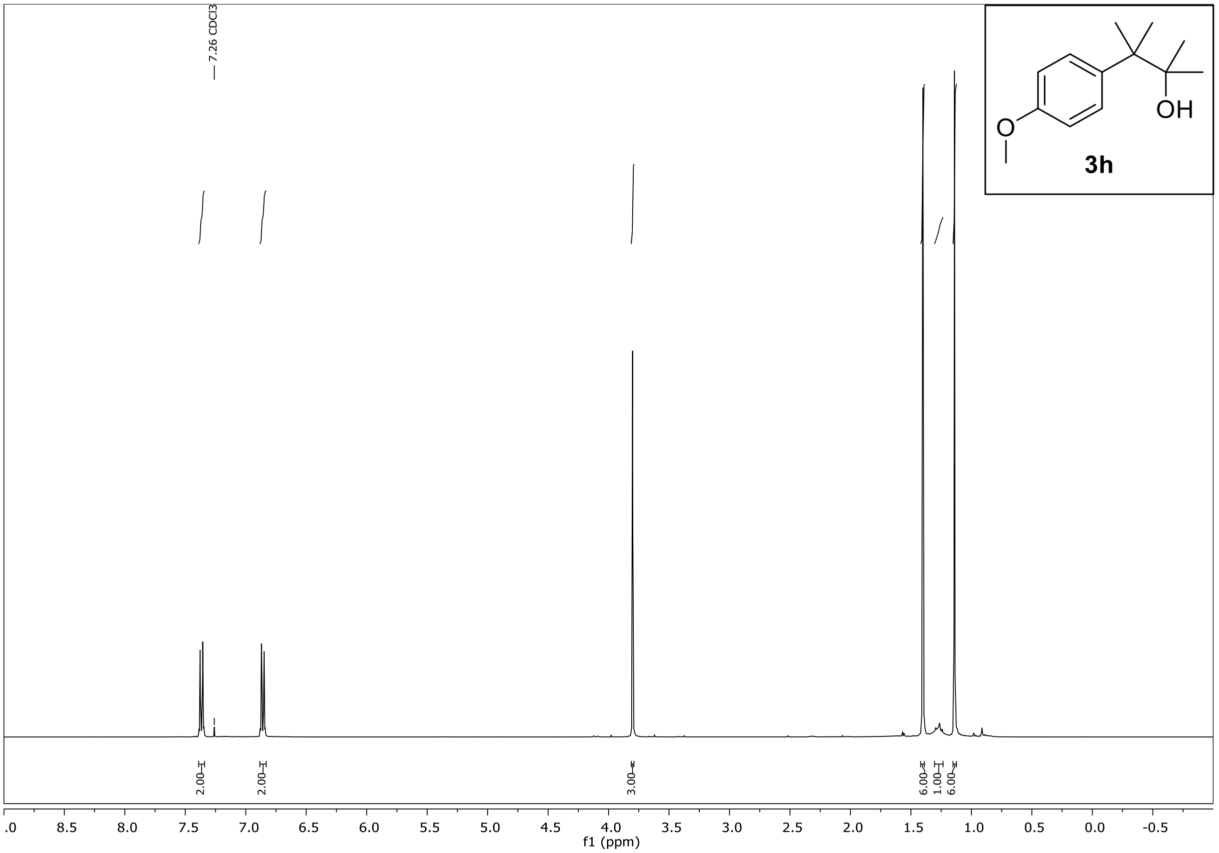

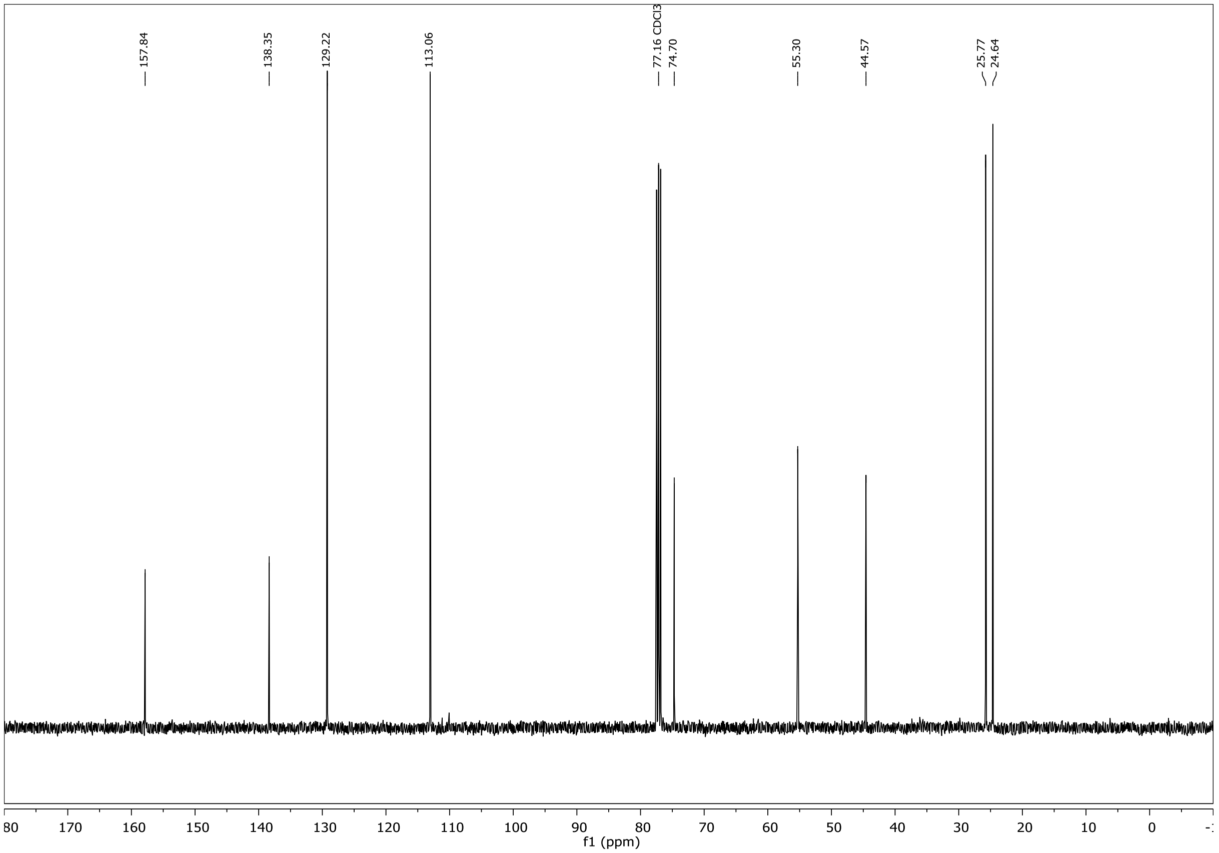

Compound **3i**, ^{1}H- and ^{13}C-NMR ($CDCl_3$)

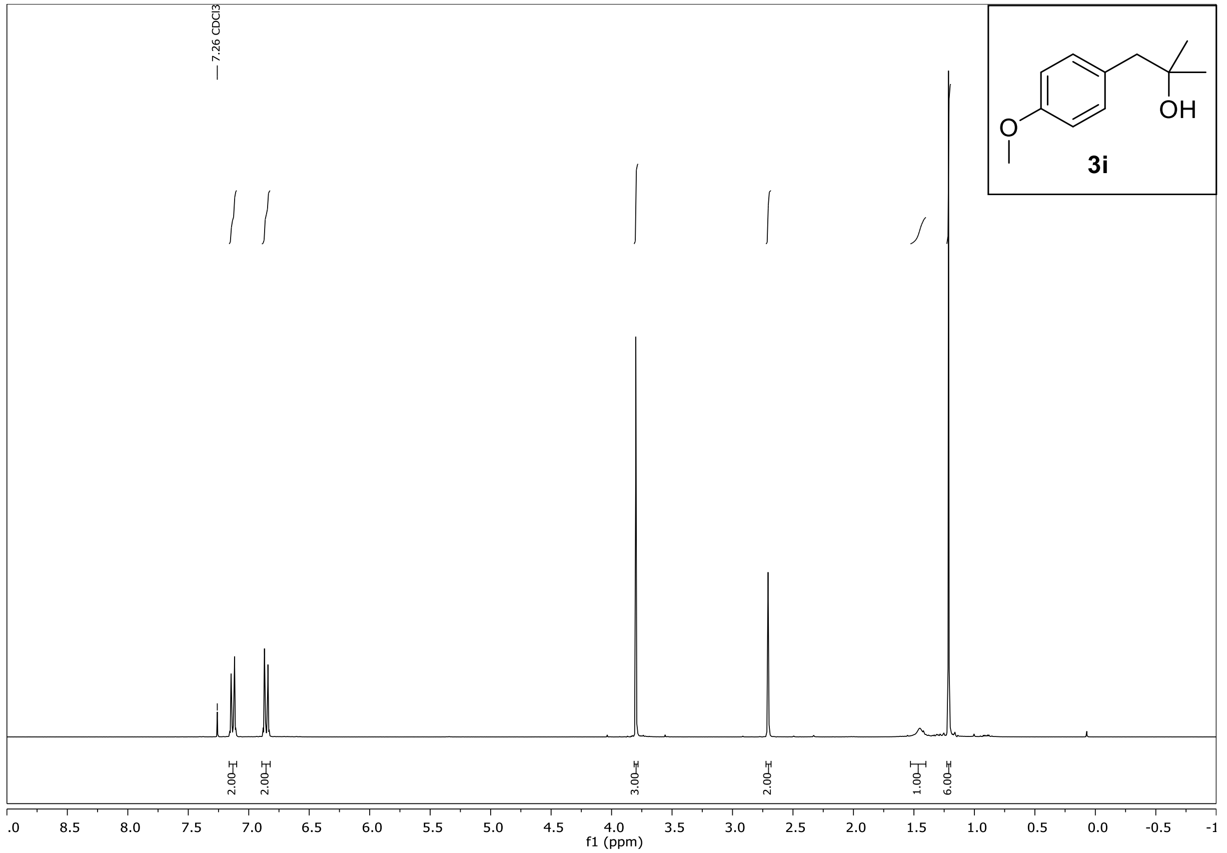

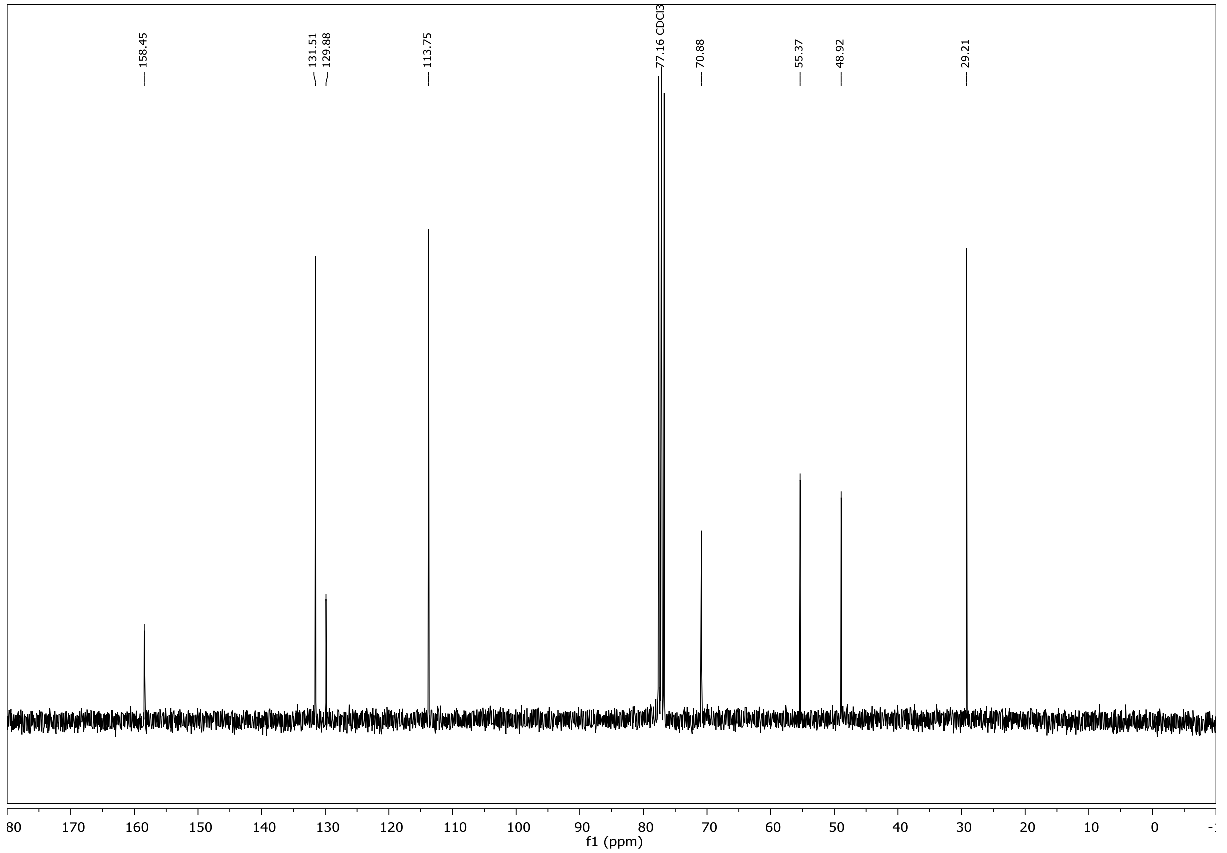

Compound **3j**, ¹H- and ¹³C-NMR ($CDCl_3$)

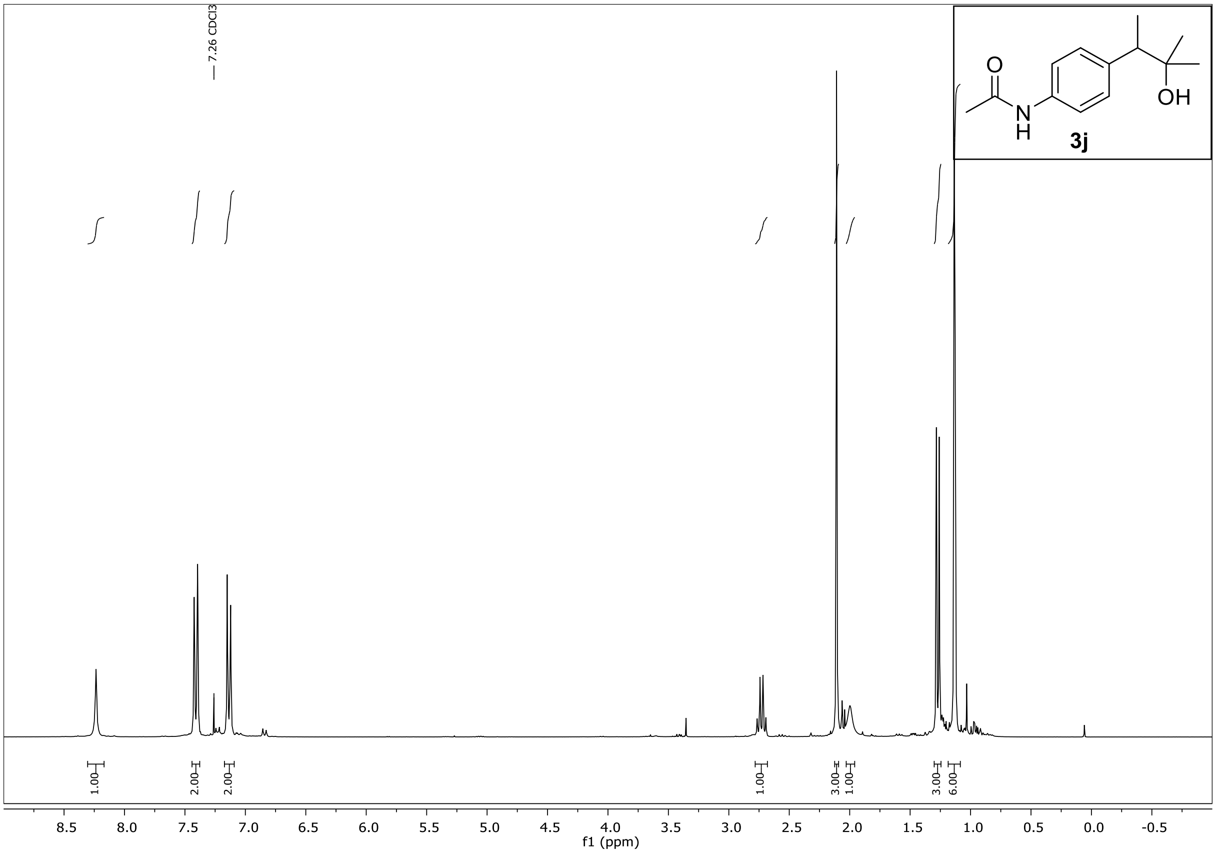

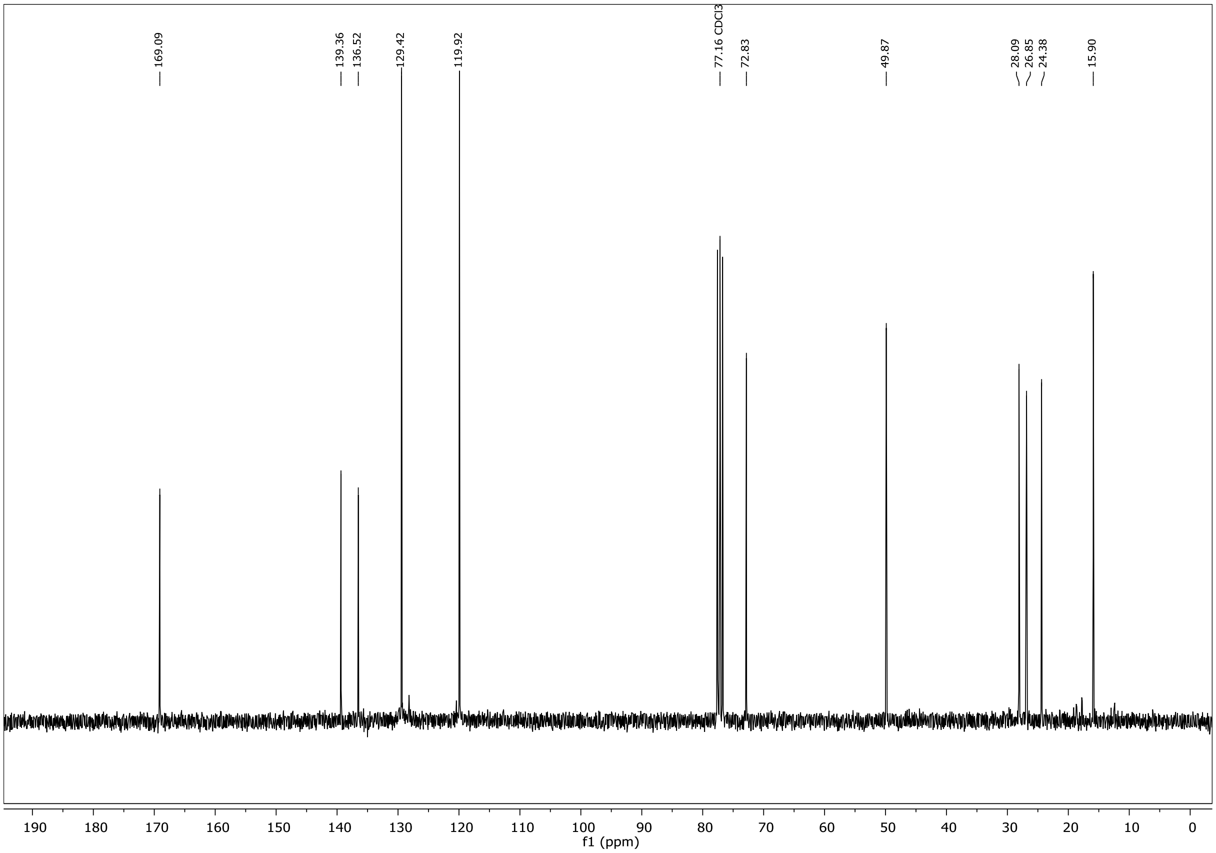

Compound **3k**, ^{1}H- and ^{13}C-NMR ($CDCl_3$)

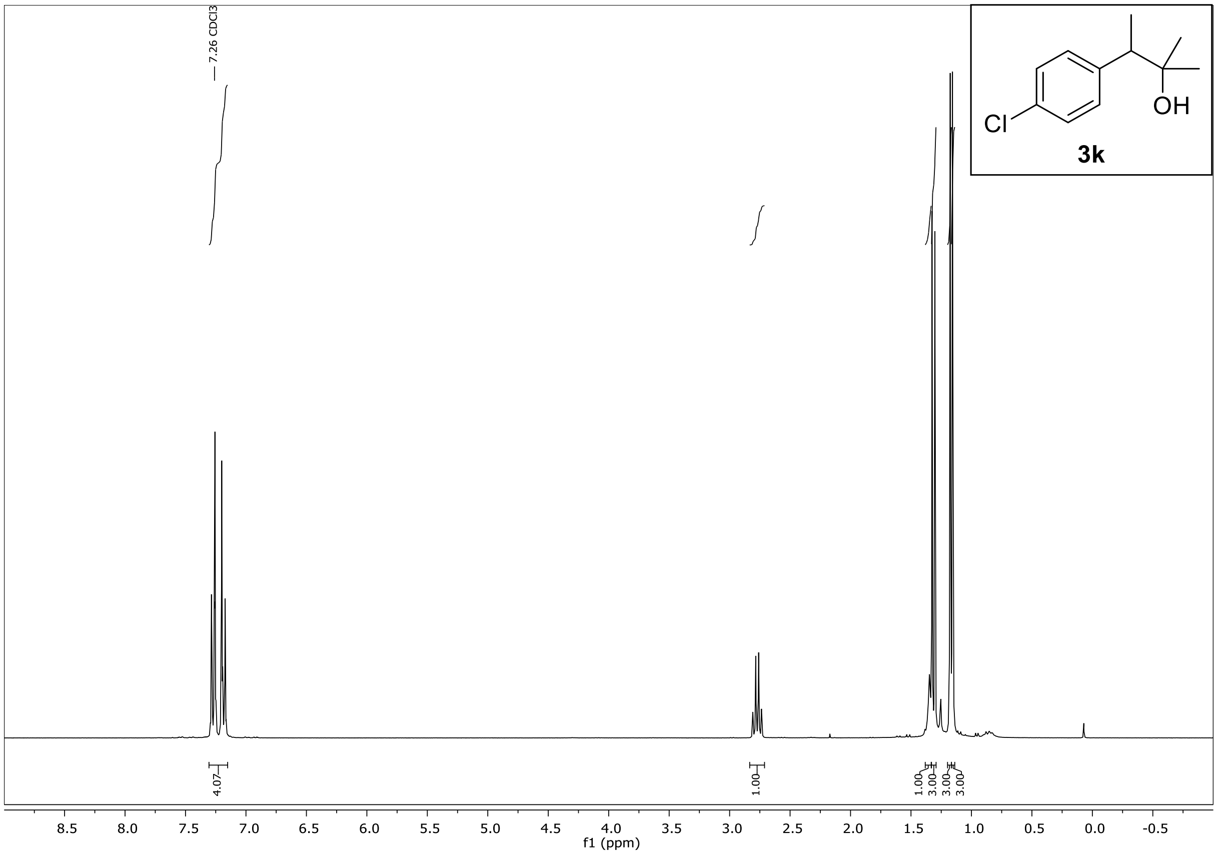

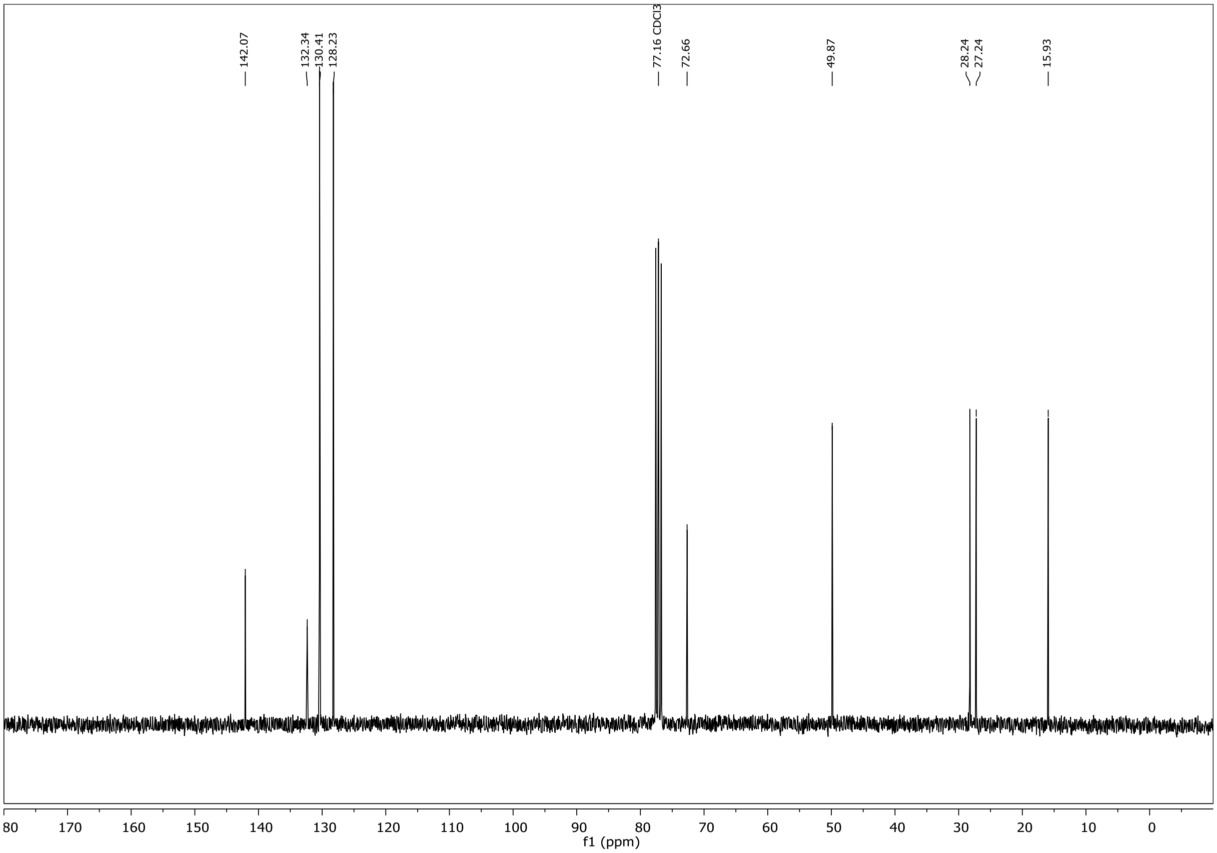

Compound **3l**, ^{1}H-, ^{13}C- and ^{19}F-NMR ($CDCl_3$)

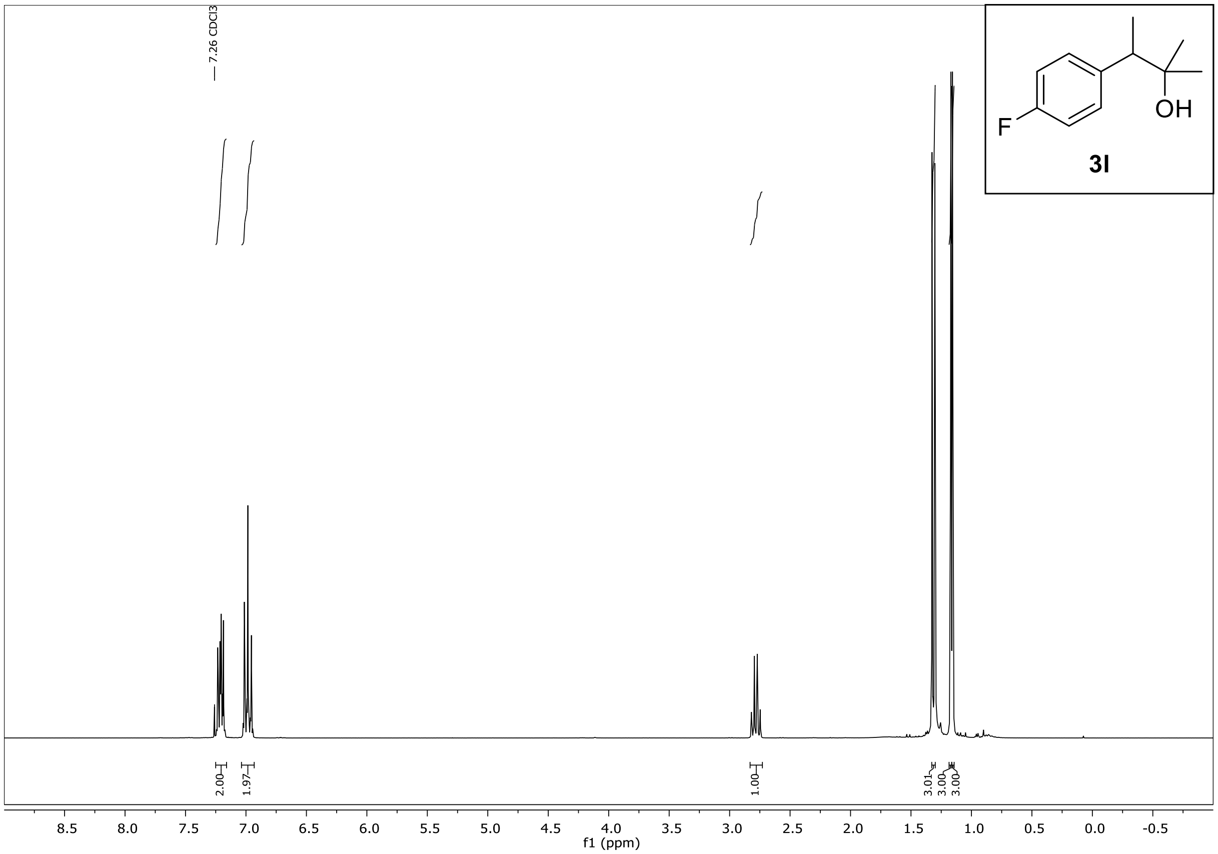

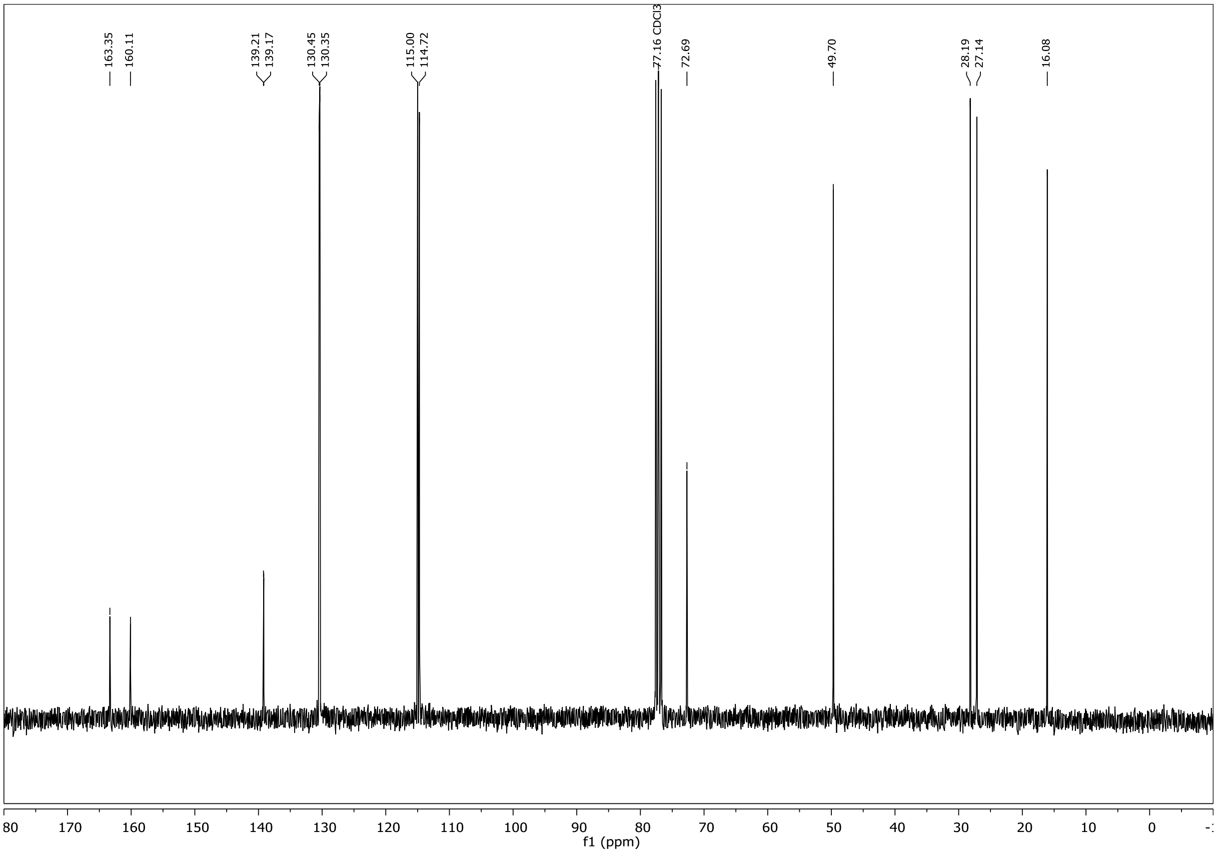

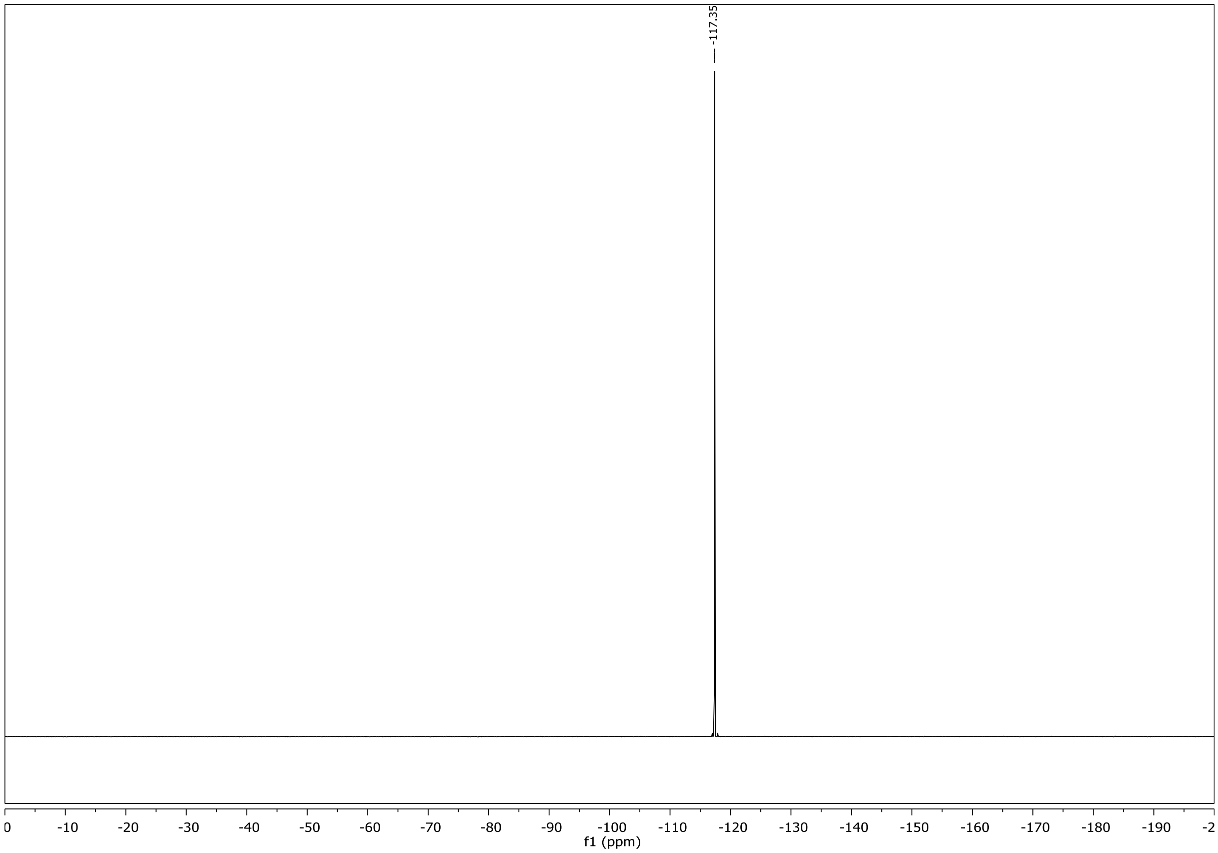
-117.35
0
-10
-20
-30
-40
-50
-60
-70
-80
-90
-100
-110
-120
-130
-140
-150
-160
-170
-180
-190
-2
f1 (ppm)

Compound **3m**, ^{1}H- and ^{13}C-NMR ($CDCl_3$)

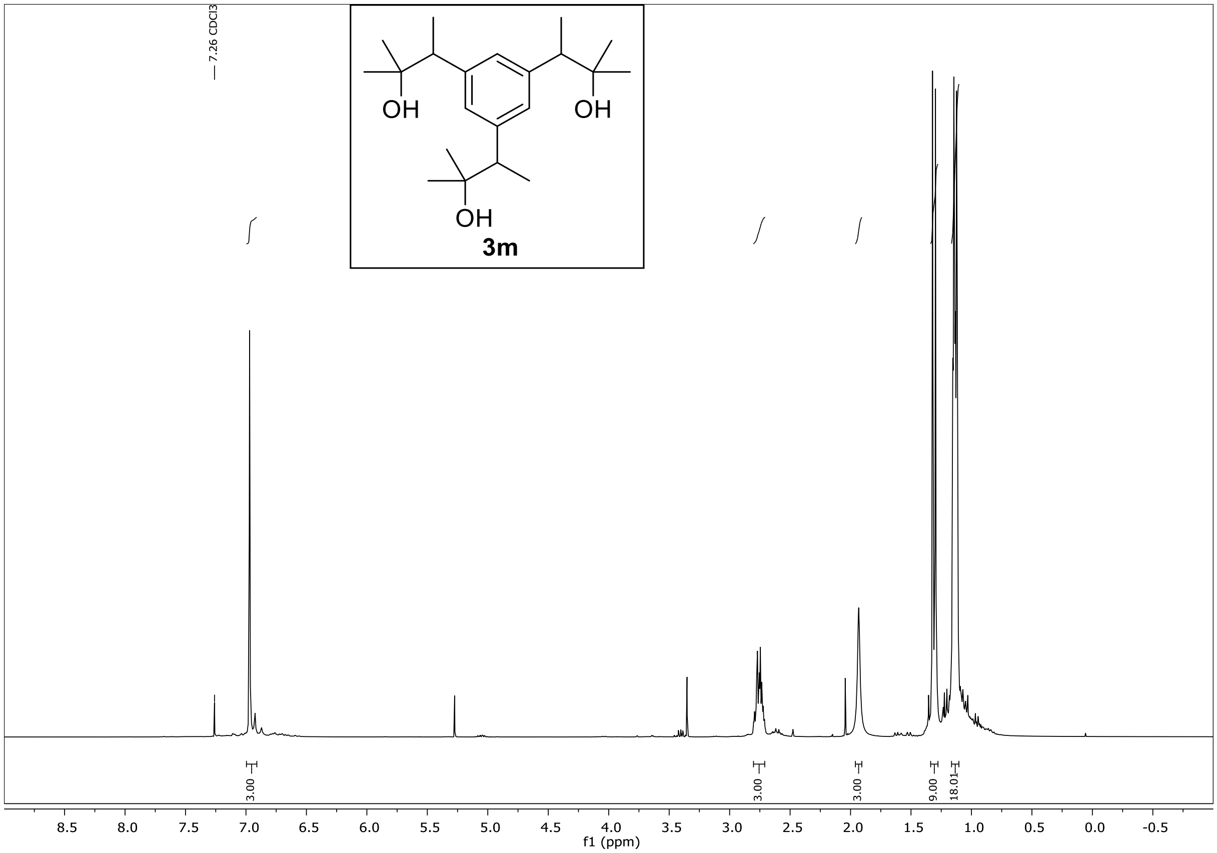

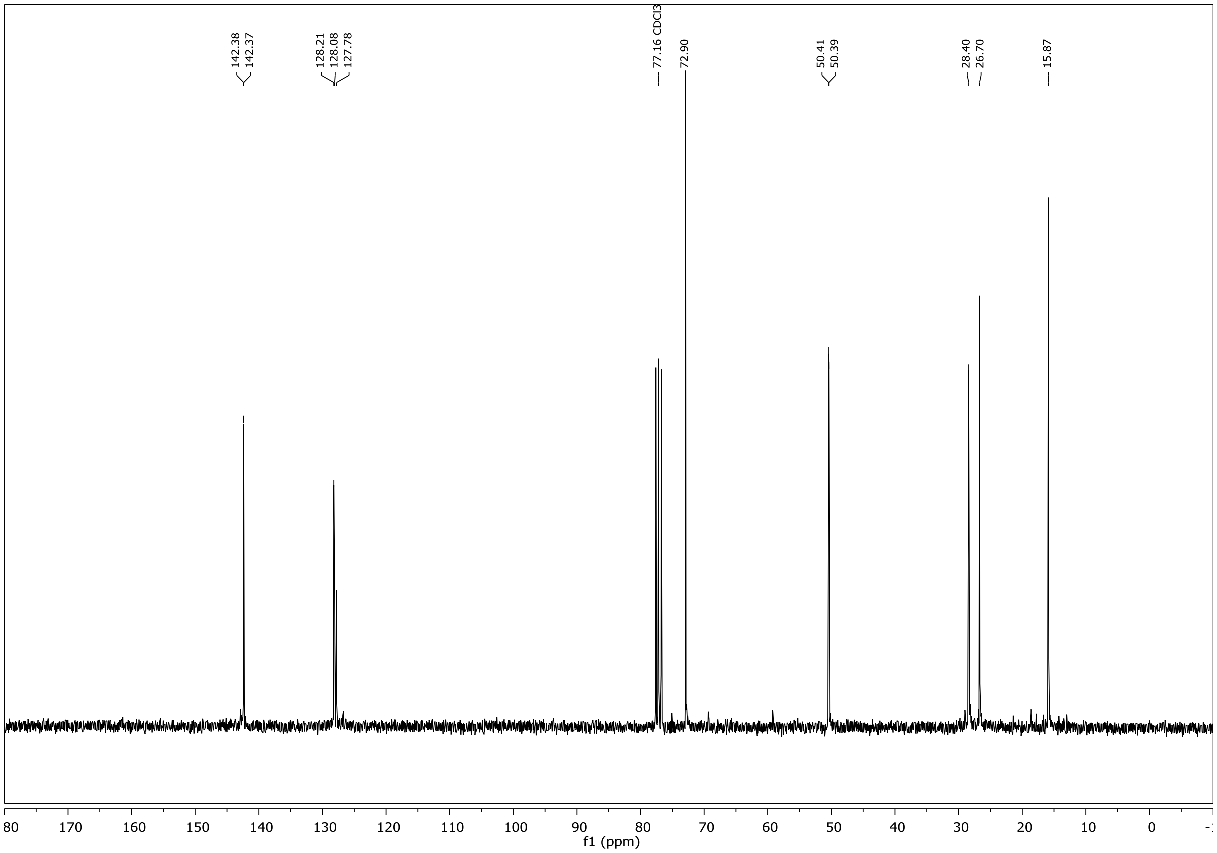

Compound **3n**, ^{1}H- and ^{13}C-NMR ($CDCl_3$)

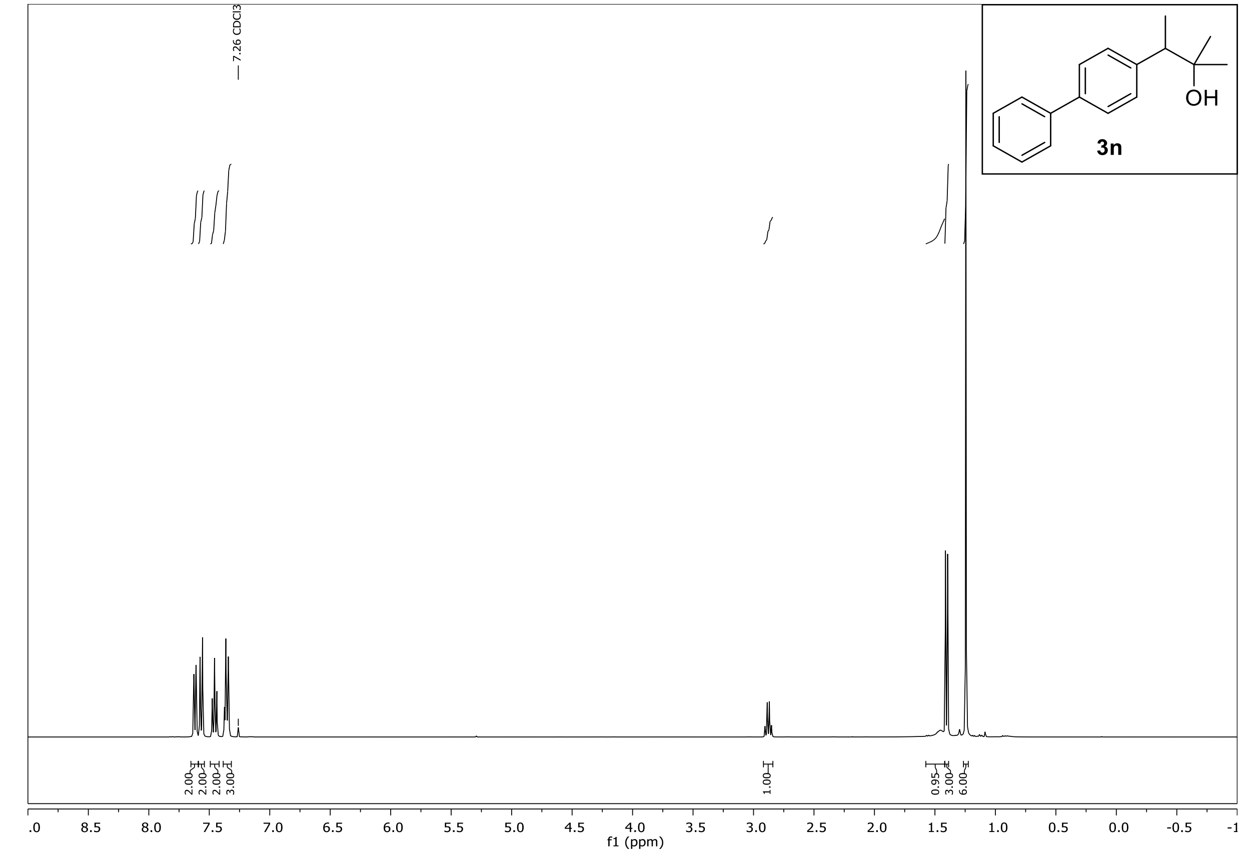

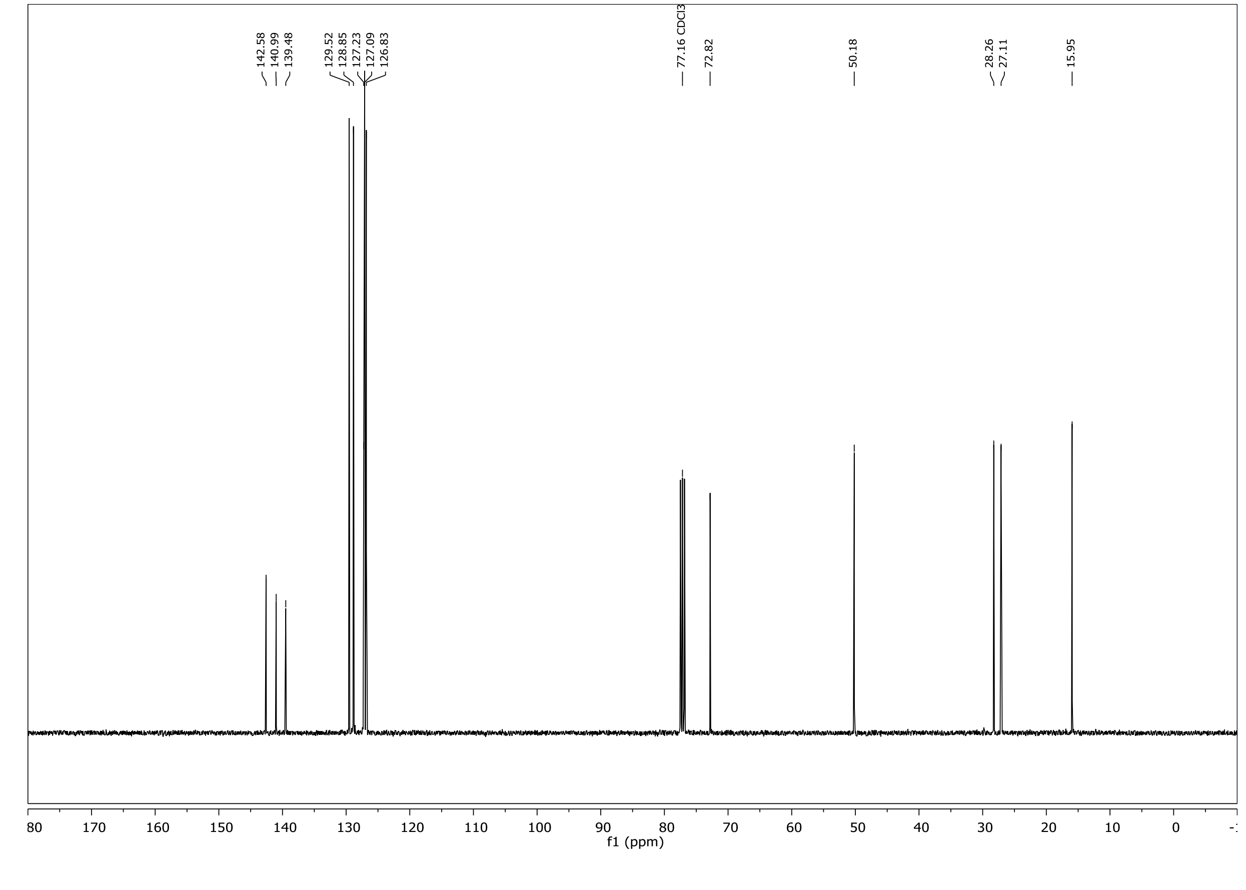

Compound **3o**, ^{1}H- and ^{13}C-NMR ($CDCl_3$)

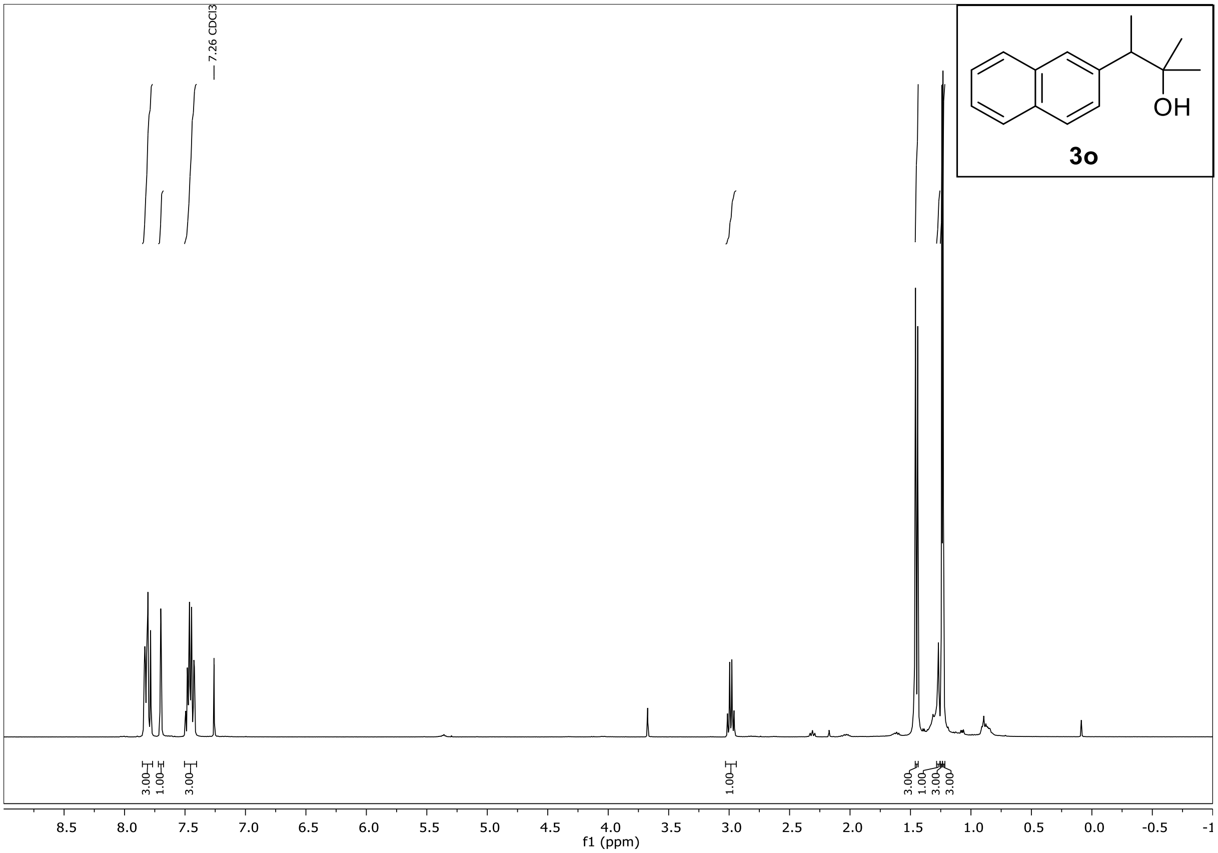

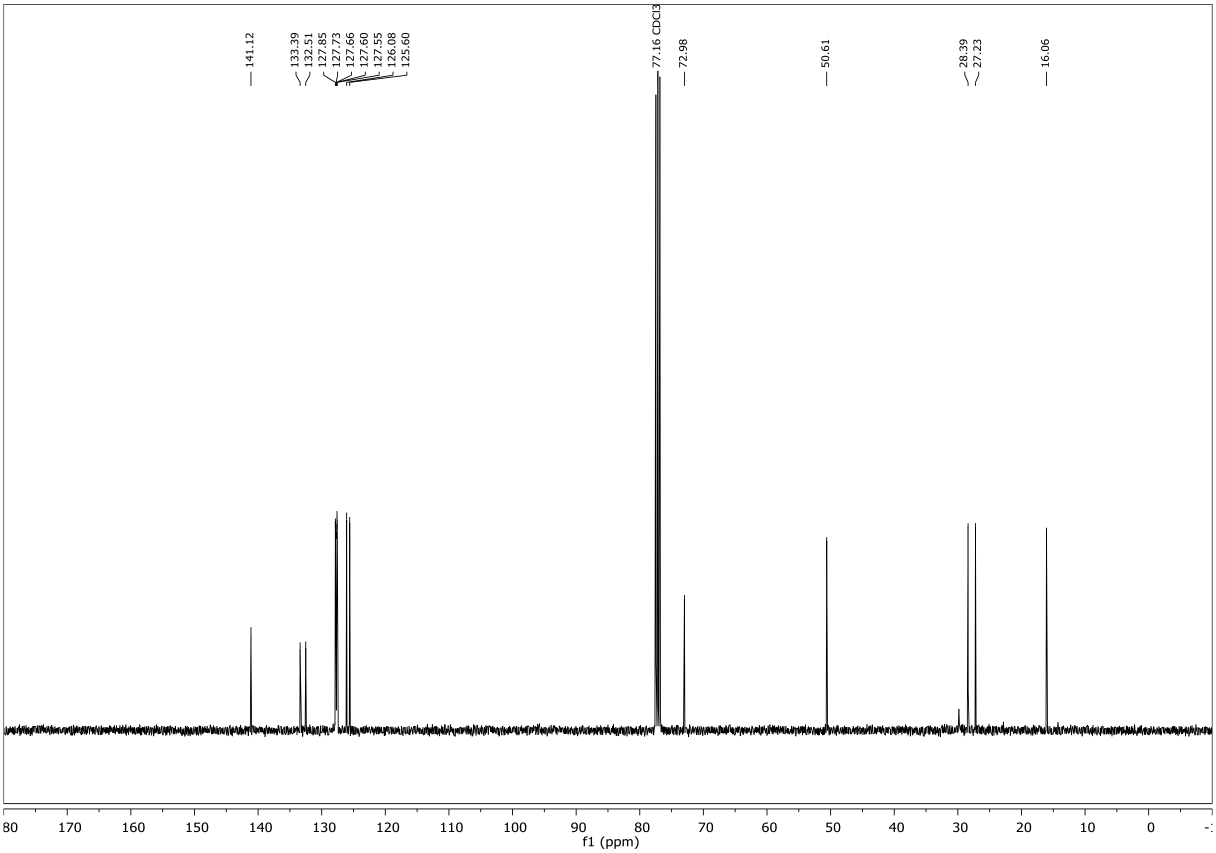

Compound **3p**, ^{1}H- and ^{13}C-NMR ($CDCl_3$)

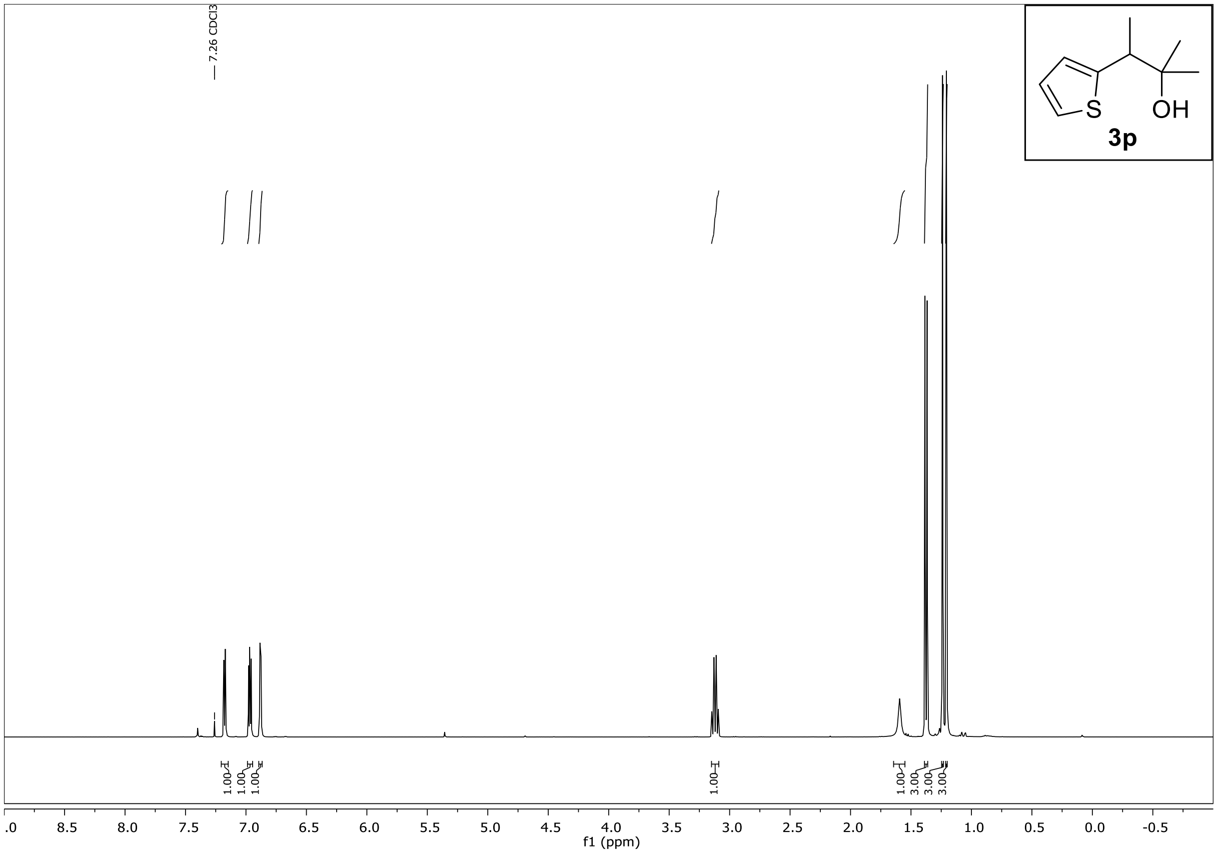

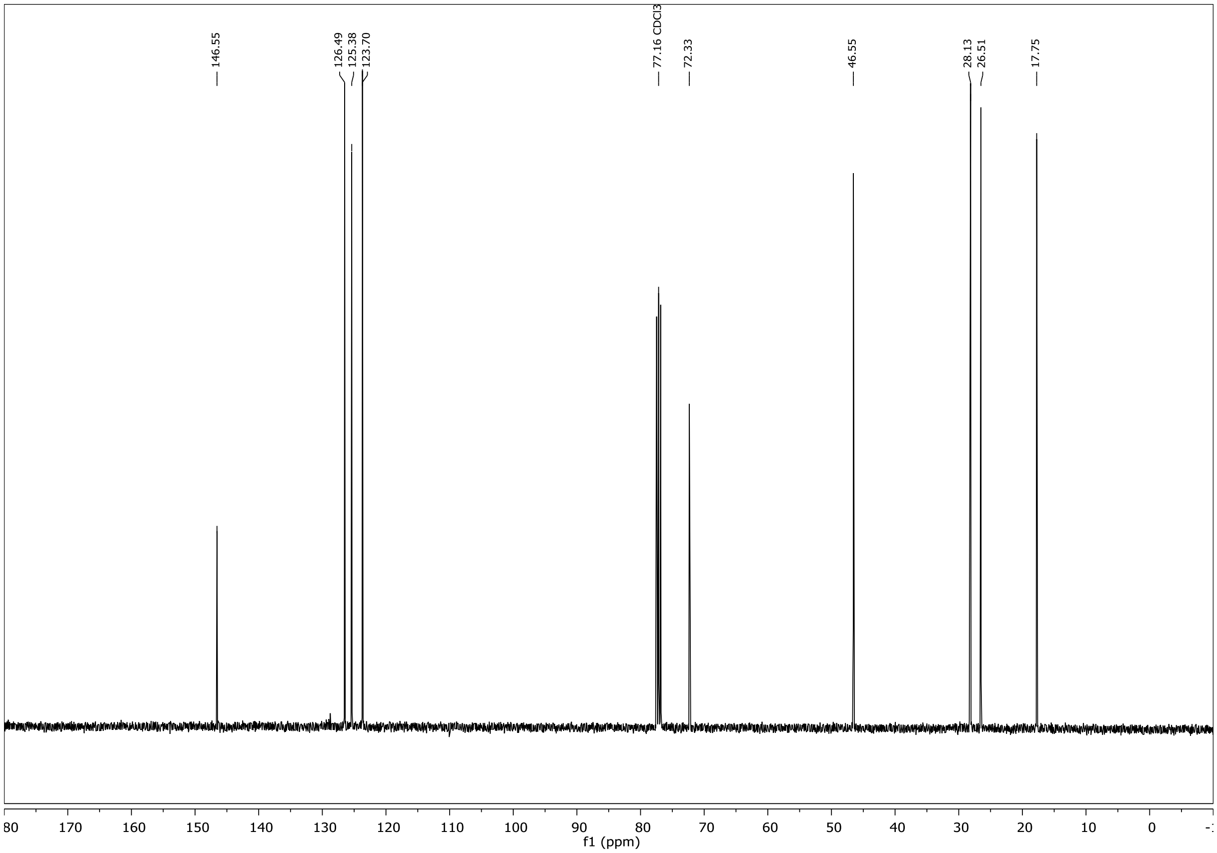

Compound **3q**, ^{1}H- and ^{13}C-NMR ($CDCl_3$)

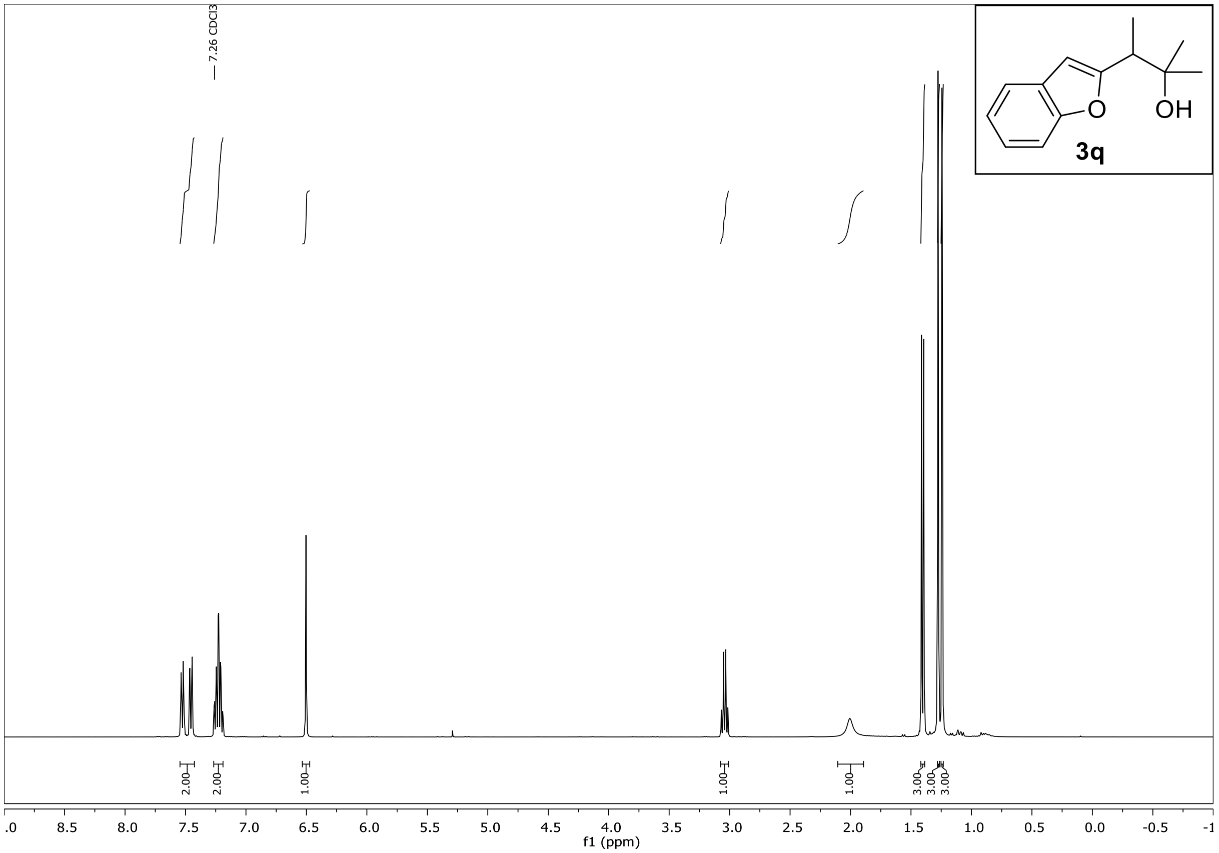

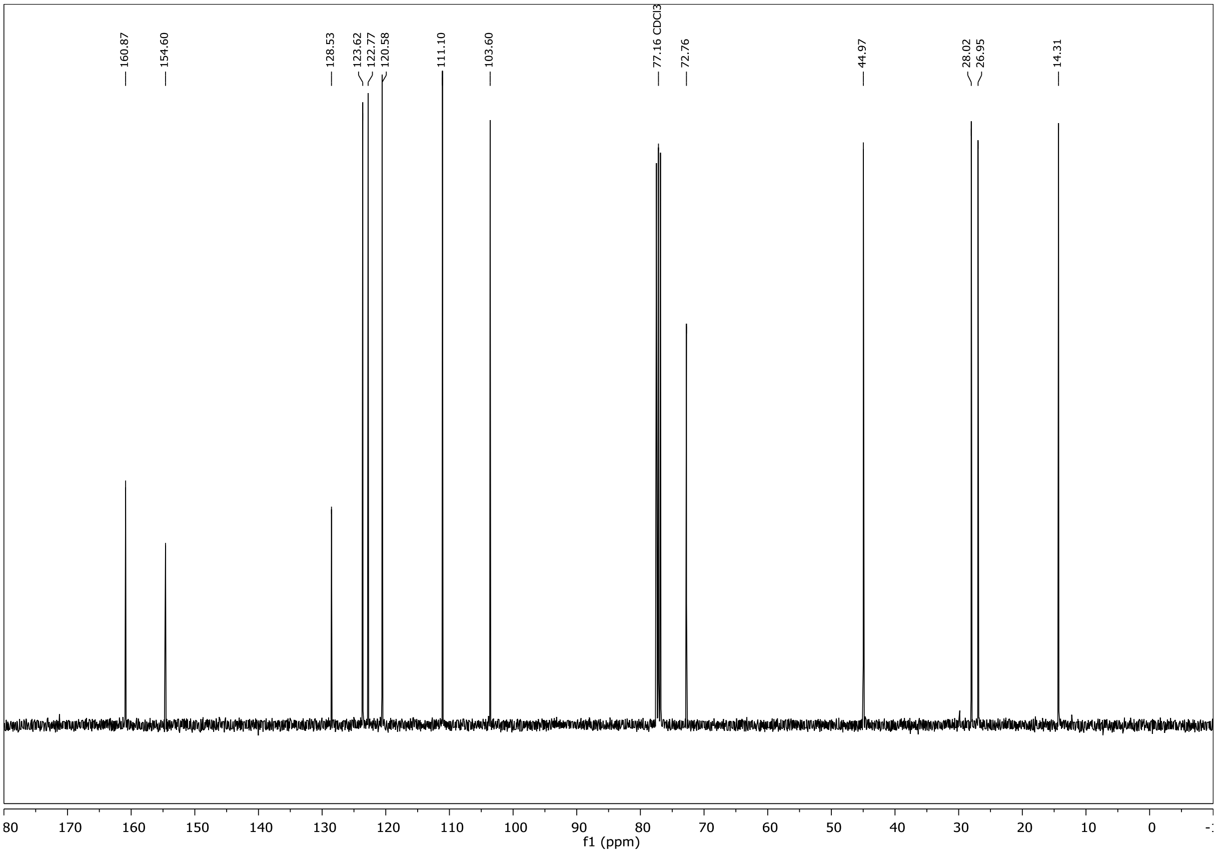

Compound **3r**, ^{1}HNMR and ^{13}C NMR ($CDCl_3$):

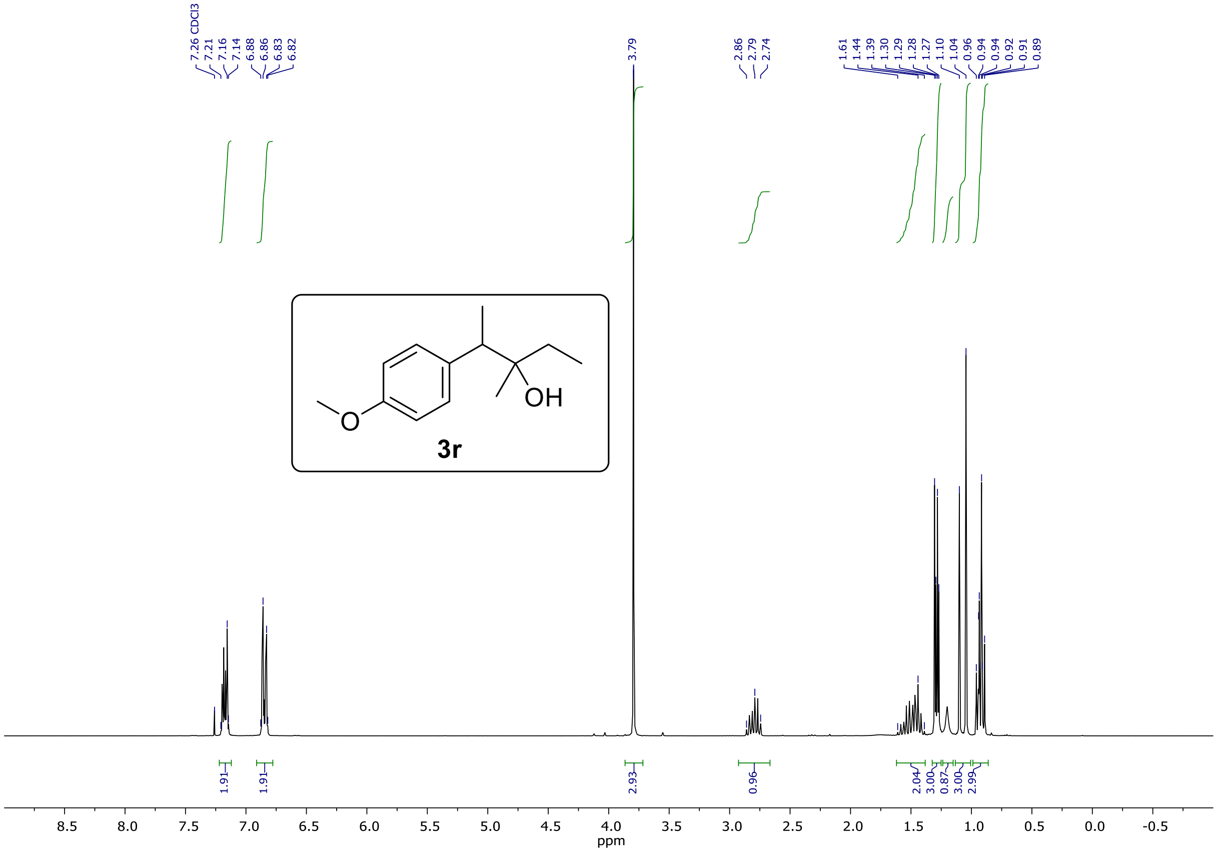

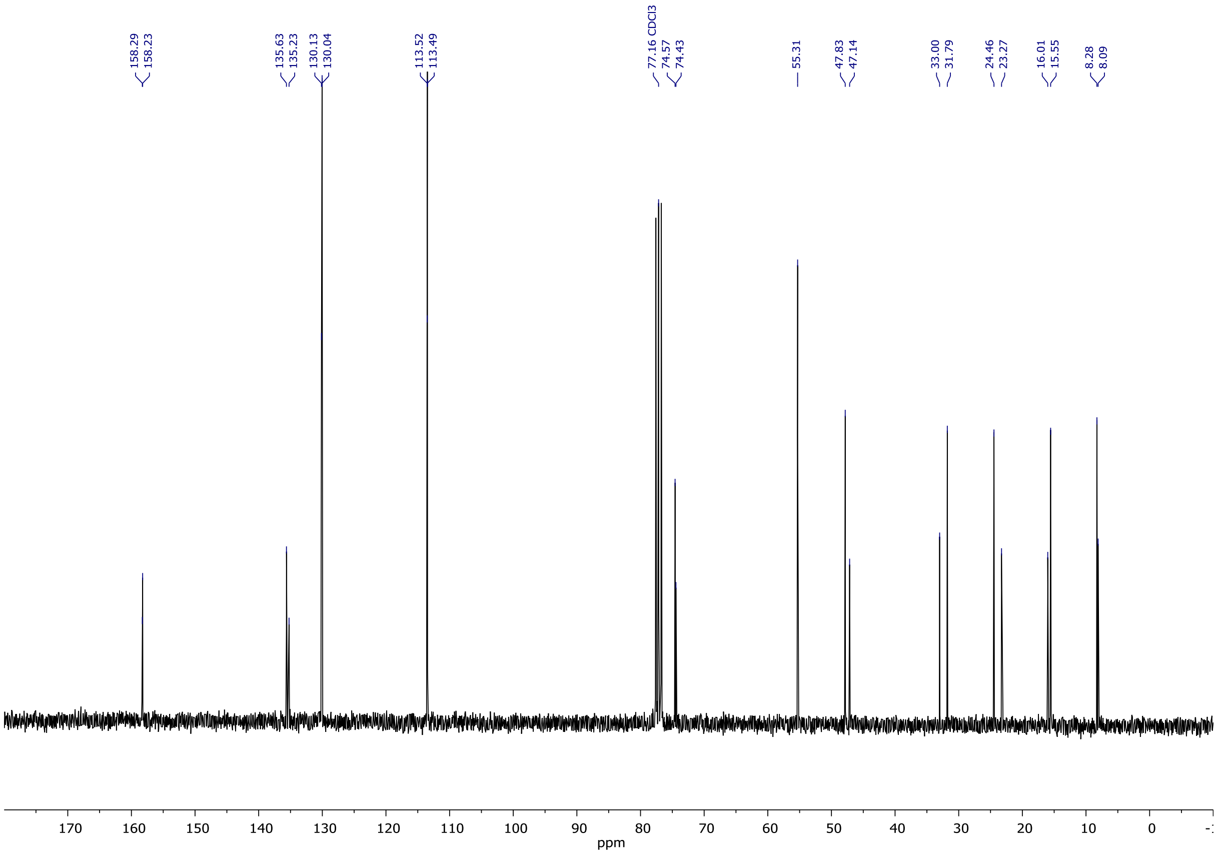

Compound **3s**, ^{1}HNMR and ^{13}C NMR ($CDCl_3$):

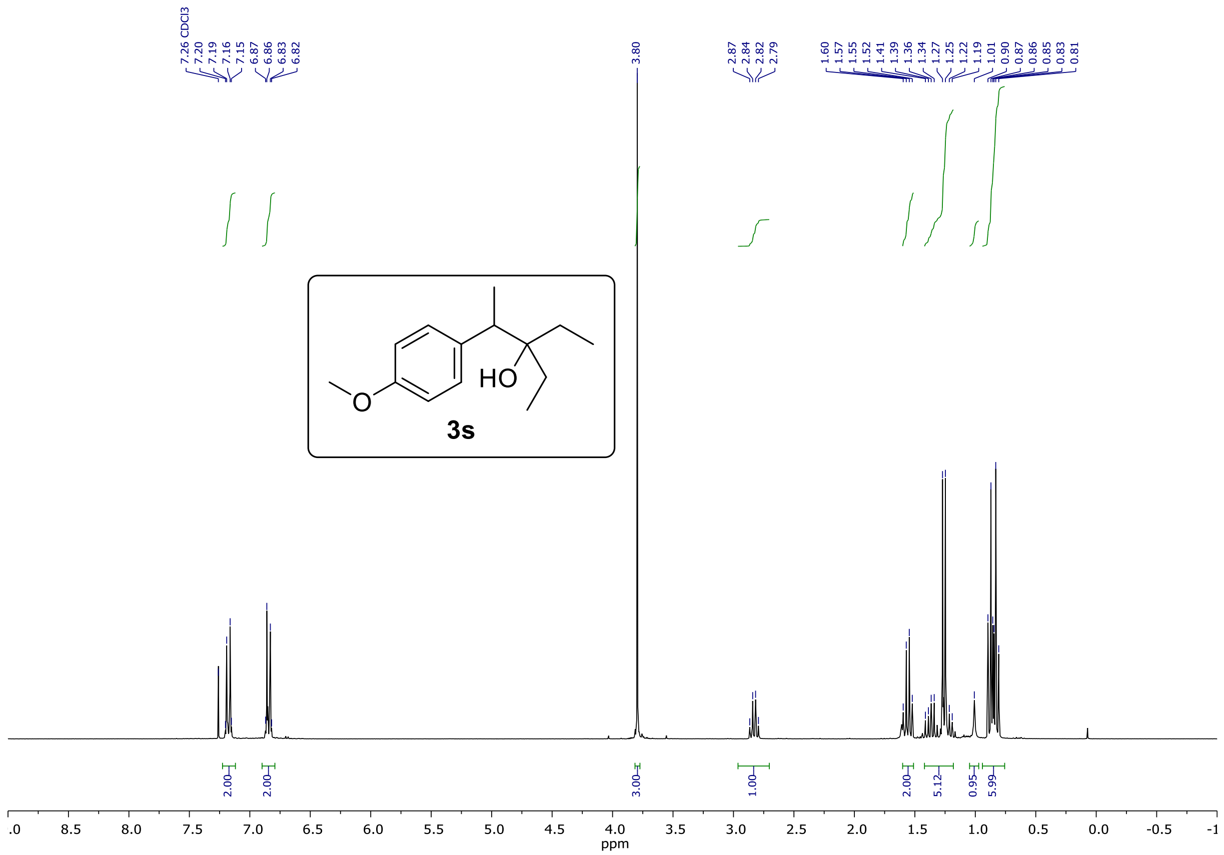

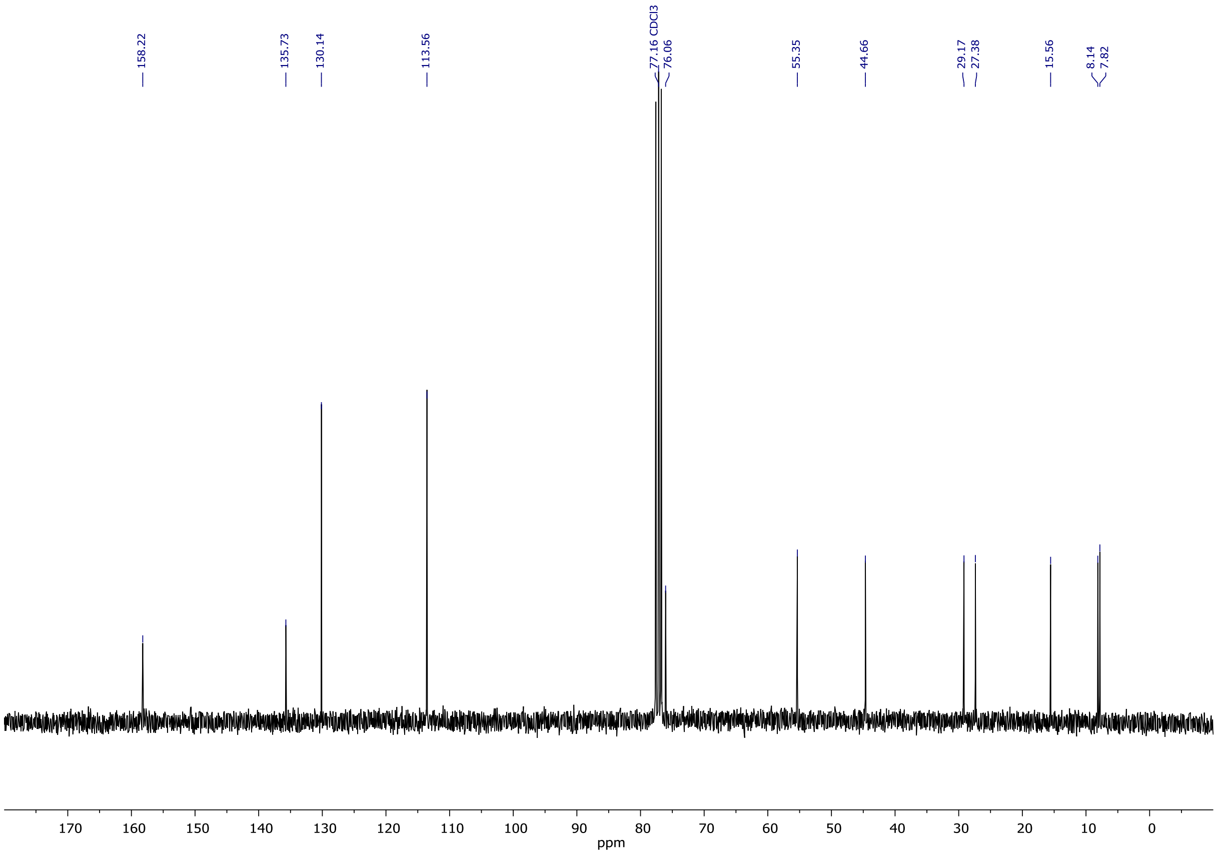

Compound **3t**, ^{1}HNMR and ^{13}C NMR ($CDCl_3$):

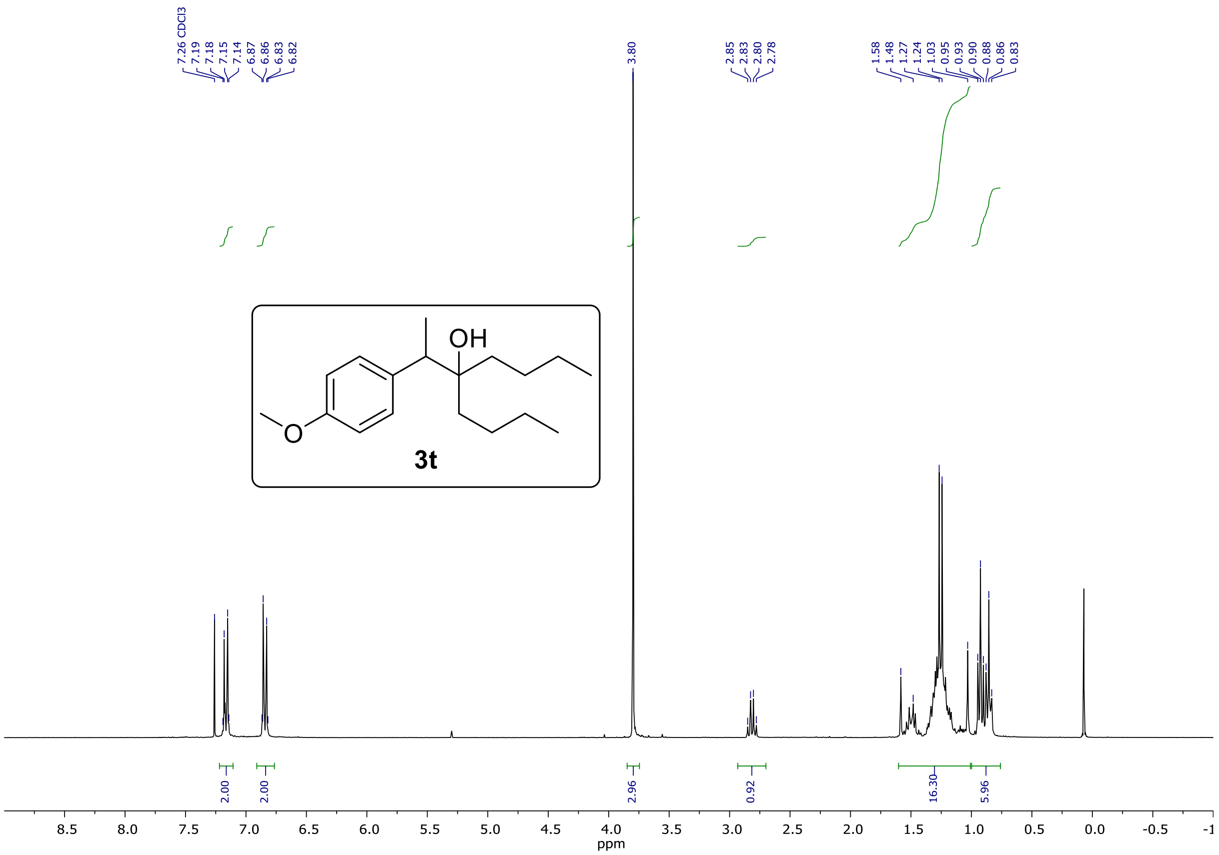

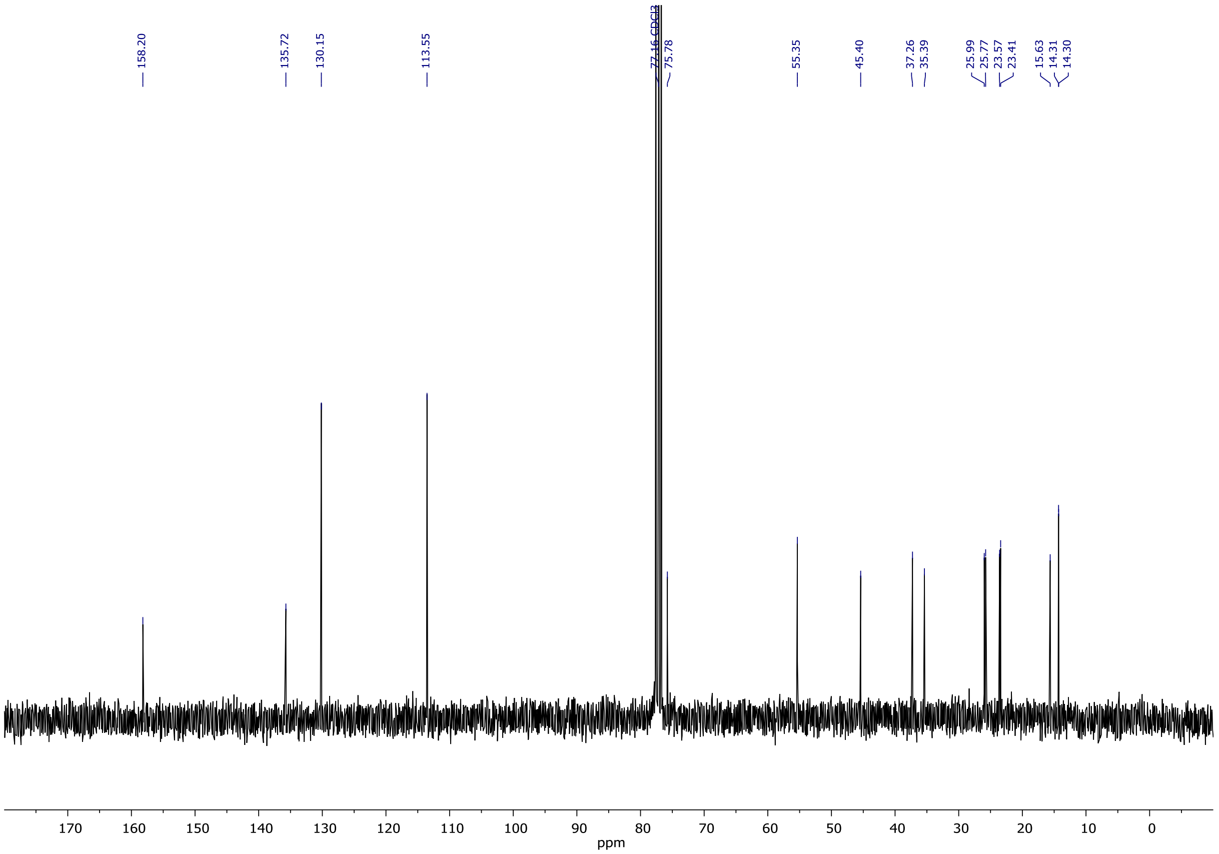

Compound **3u**, ^{1}HNMR and ^{13}C NMR ($CDCl_3$):

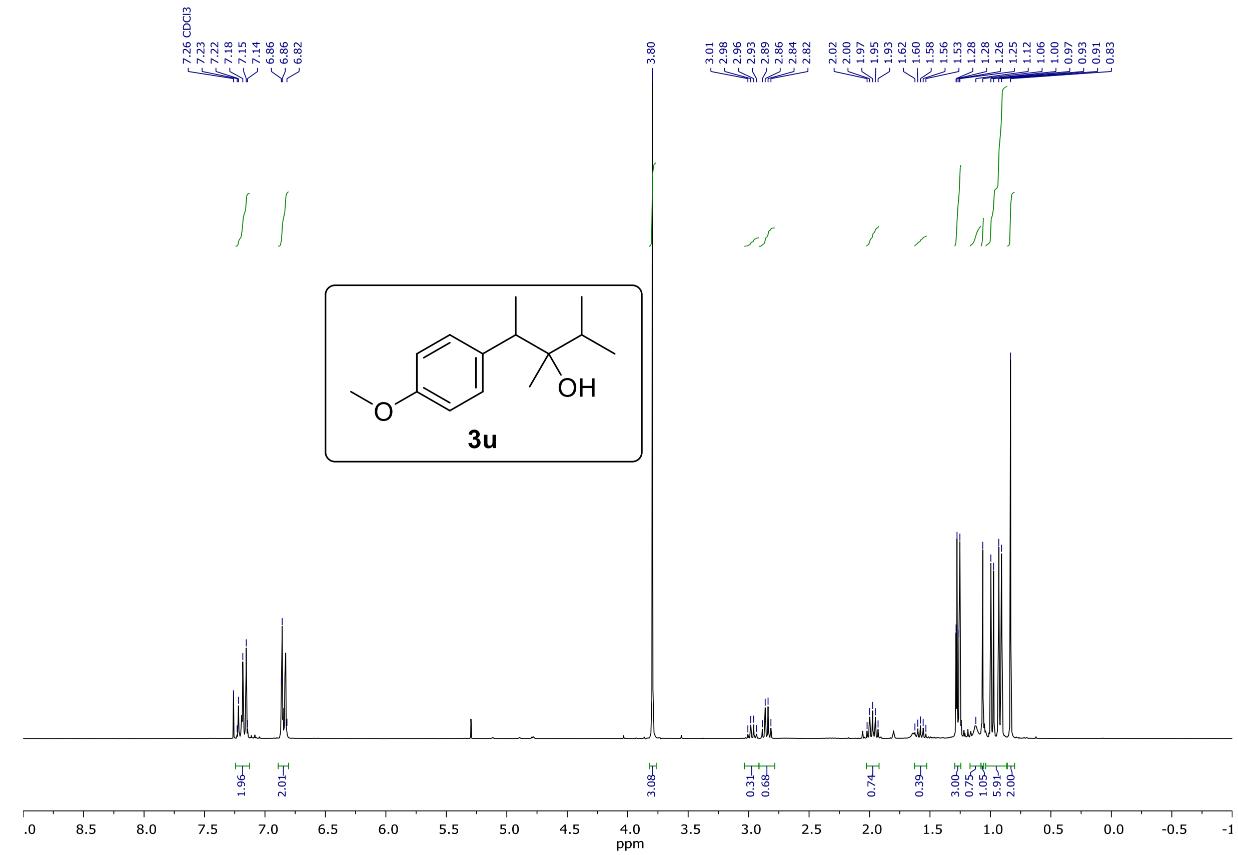

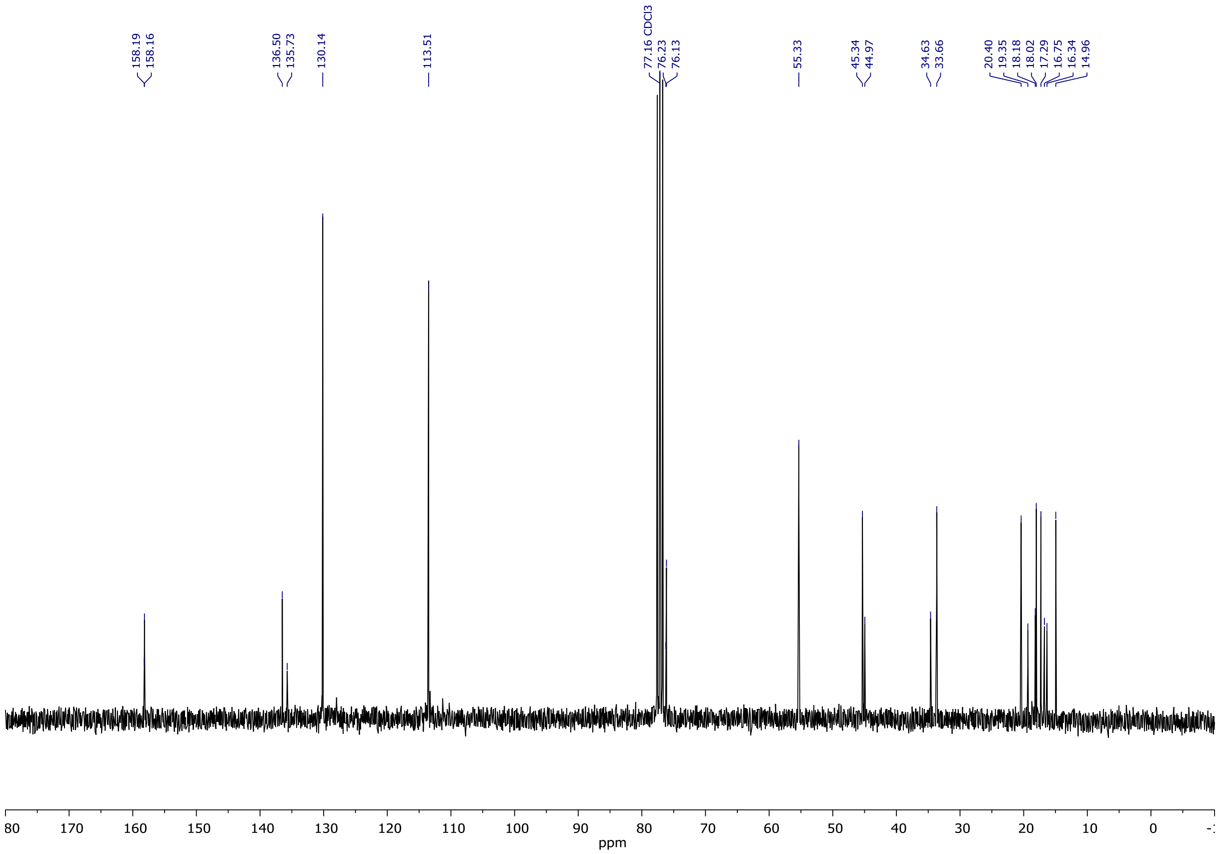

Compound **3v**, ^{1}HNMR and ^{13}C NMR ($CDCl_3$):

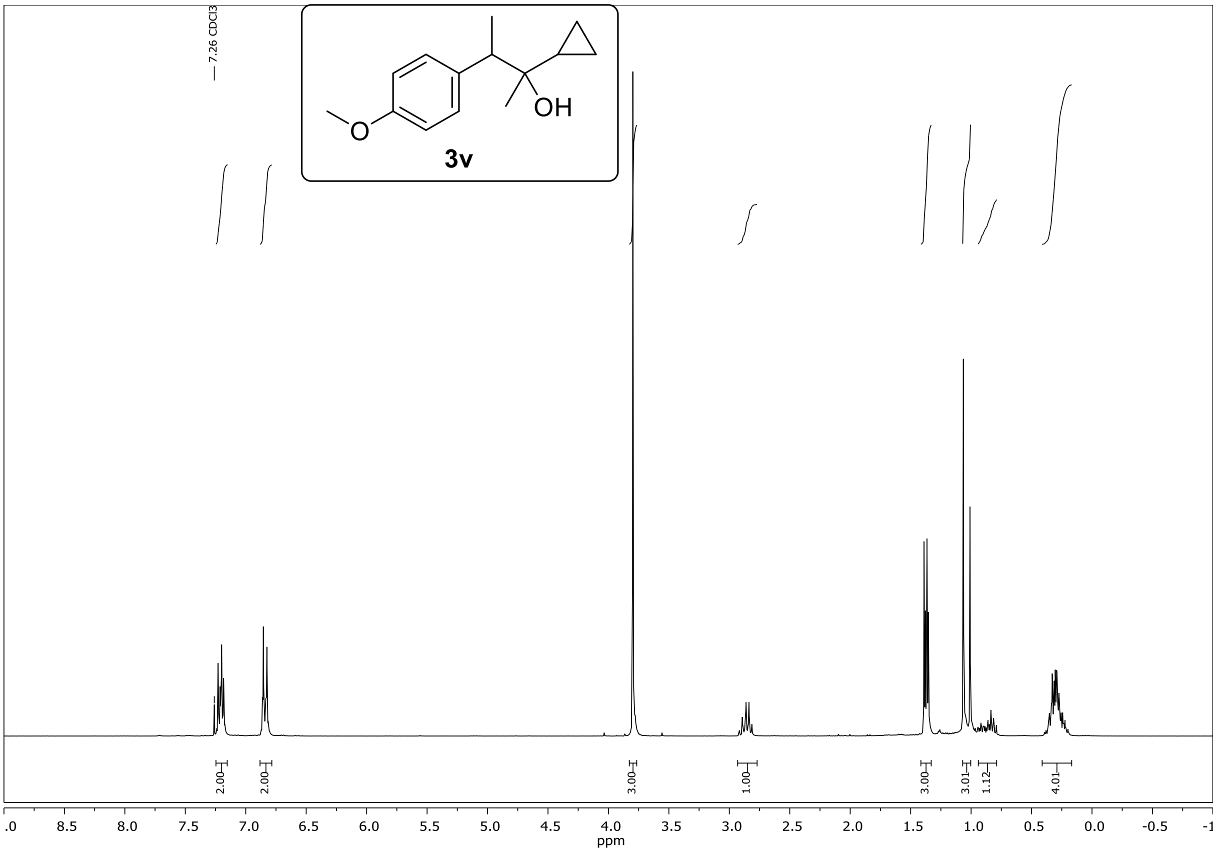

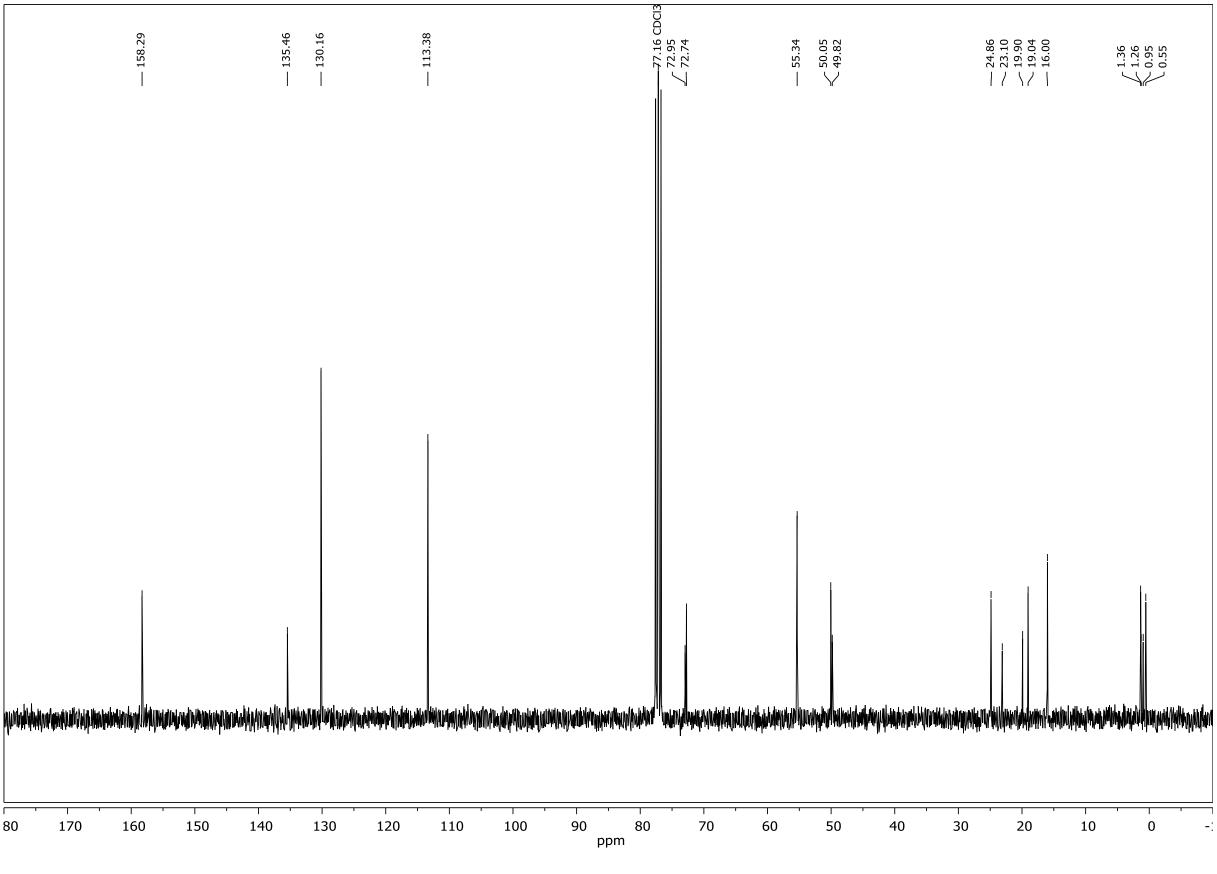

Compound **3w**, ^{1}HNMR and ^{13}C NMR ($CDCl_3$):

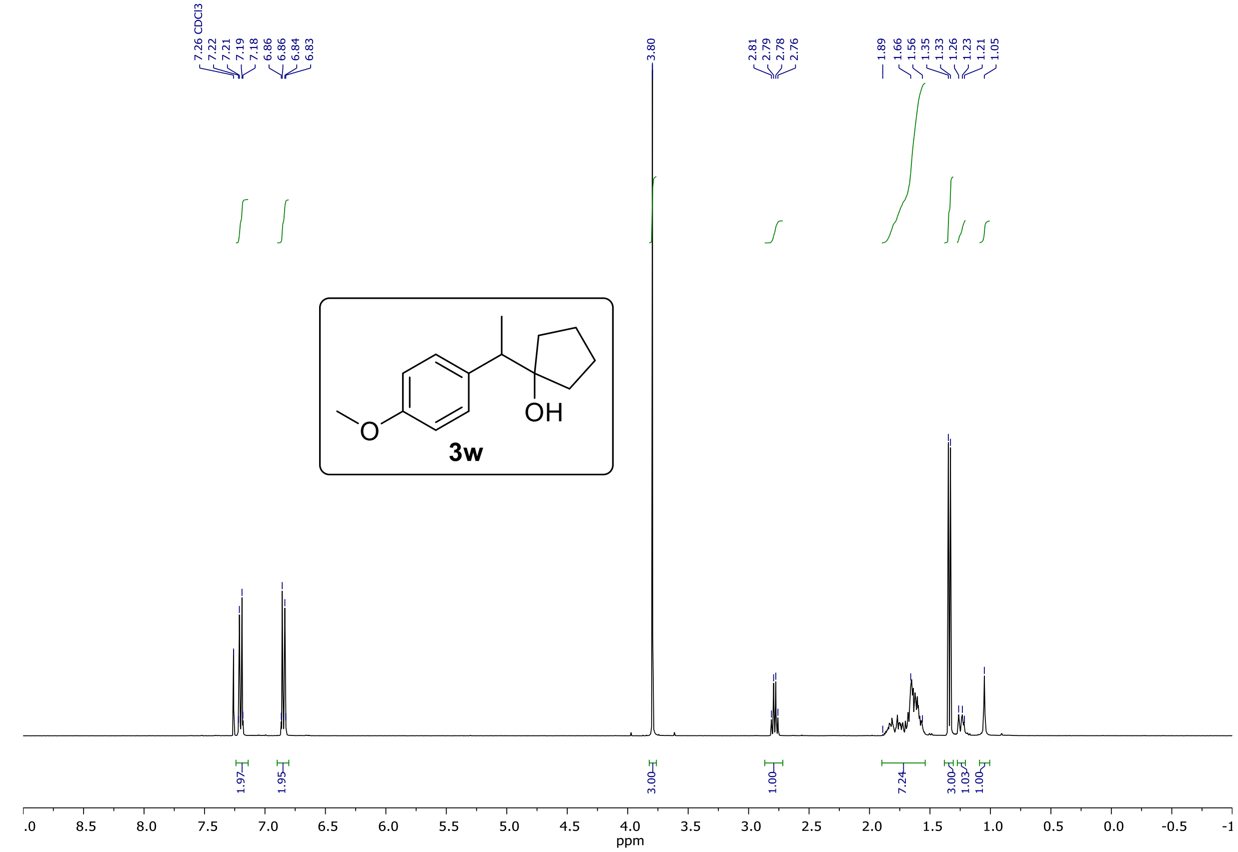

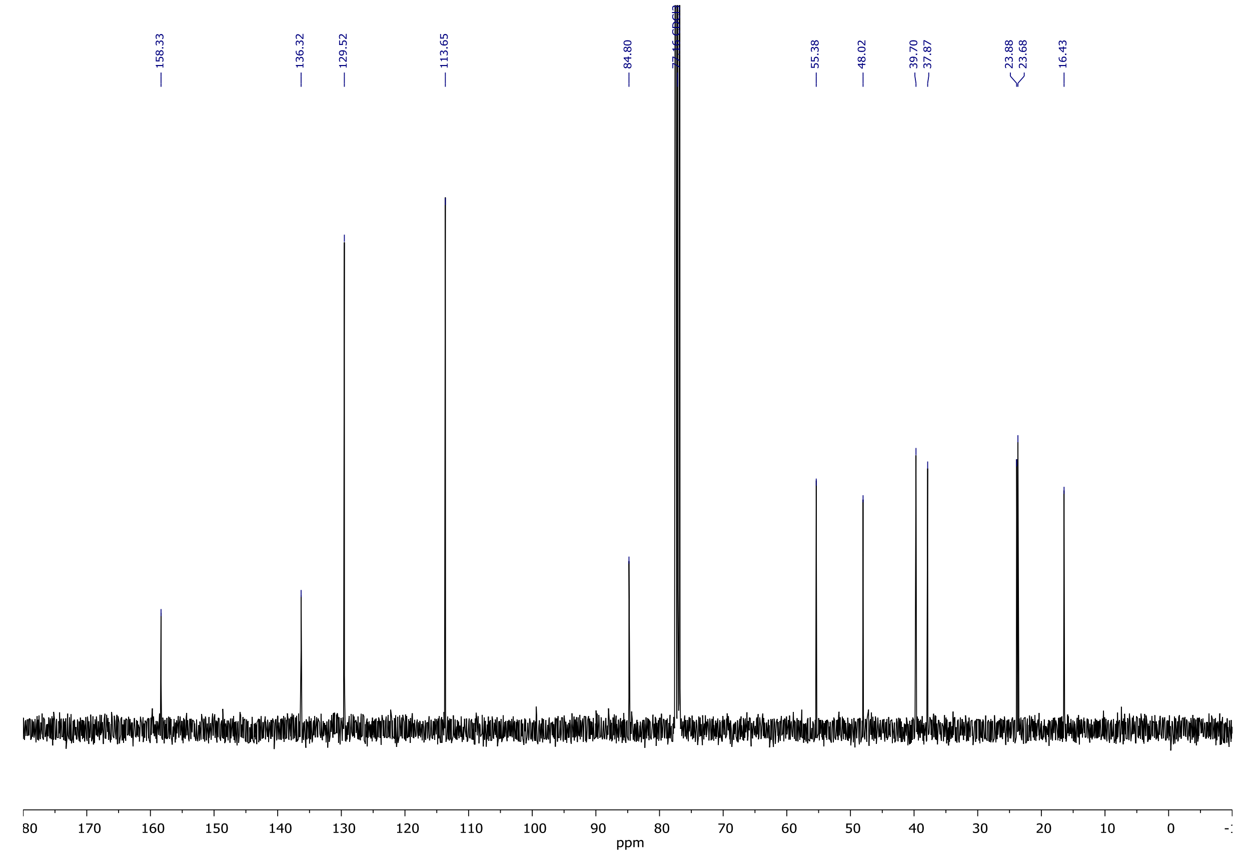

Compound **3x**, ^{1}HNMR and ^{13}C NMR ($CDCl_3$):

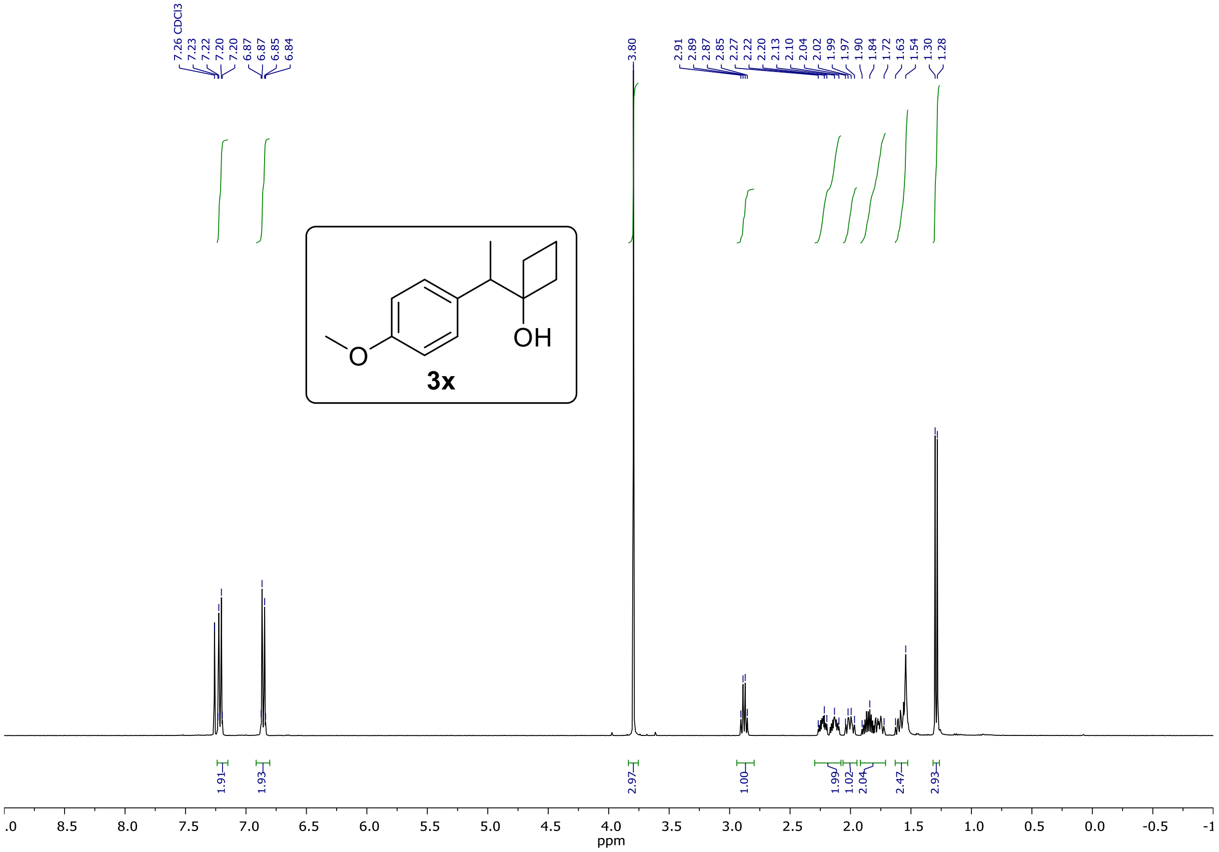

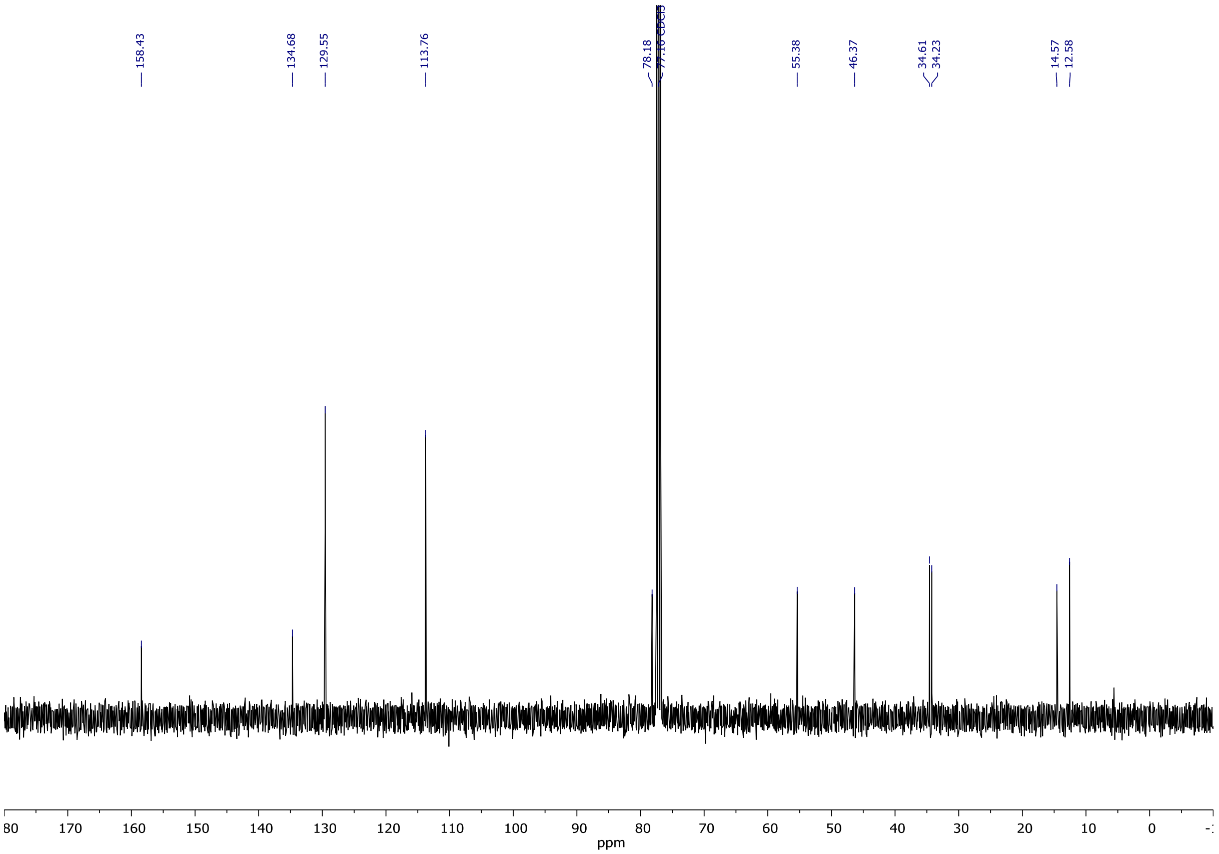

Compound **3y**, ^{1}HNMR and ^{13}C NMR ($CDCl_3$):

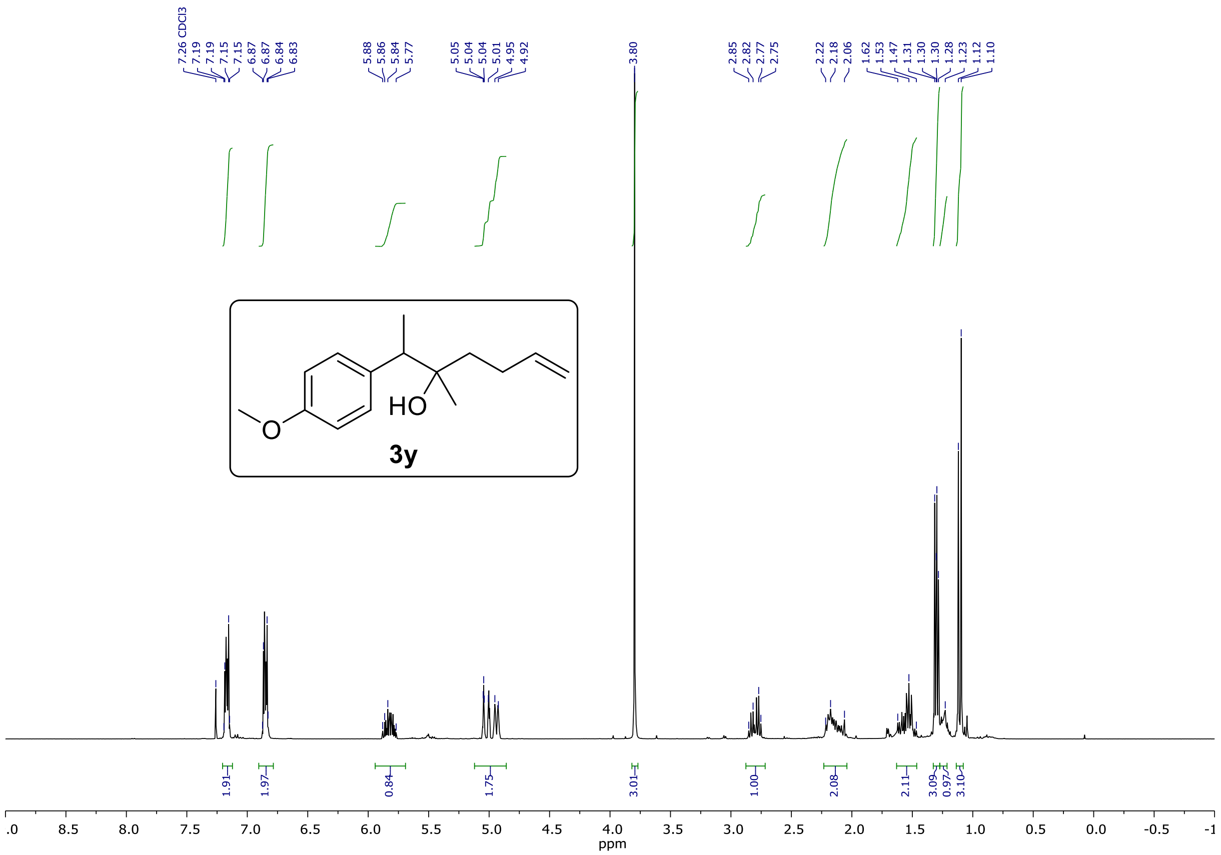

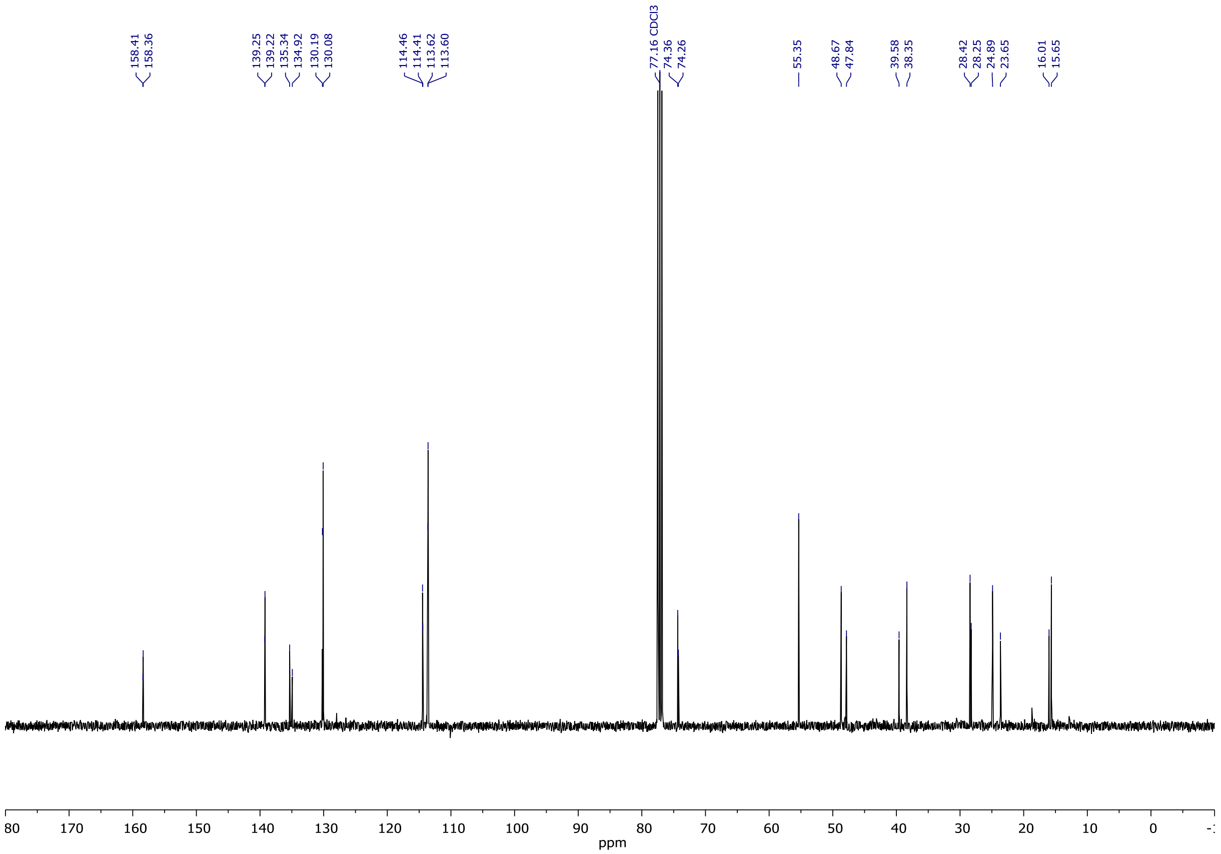

Compound **3z**, ^{1}HNMR and ^{13}C NMR ($CDCl_3$):

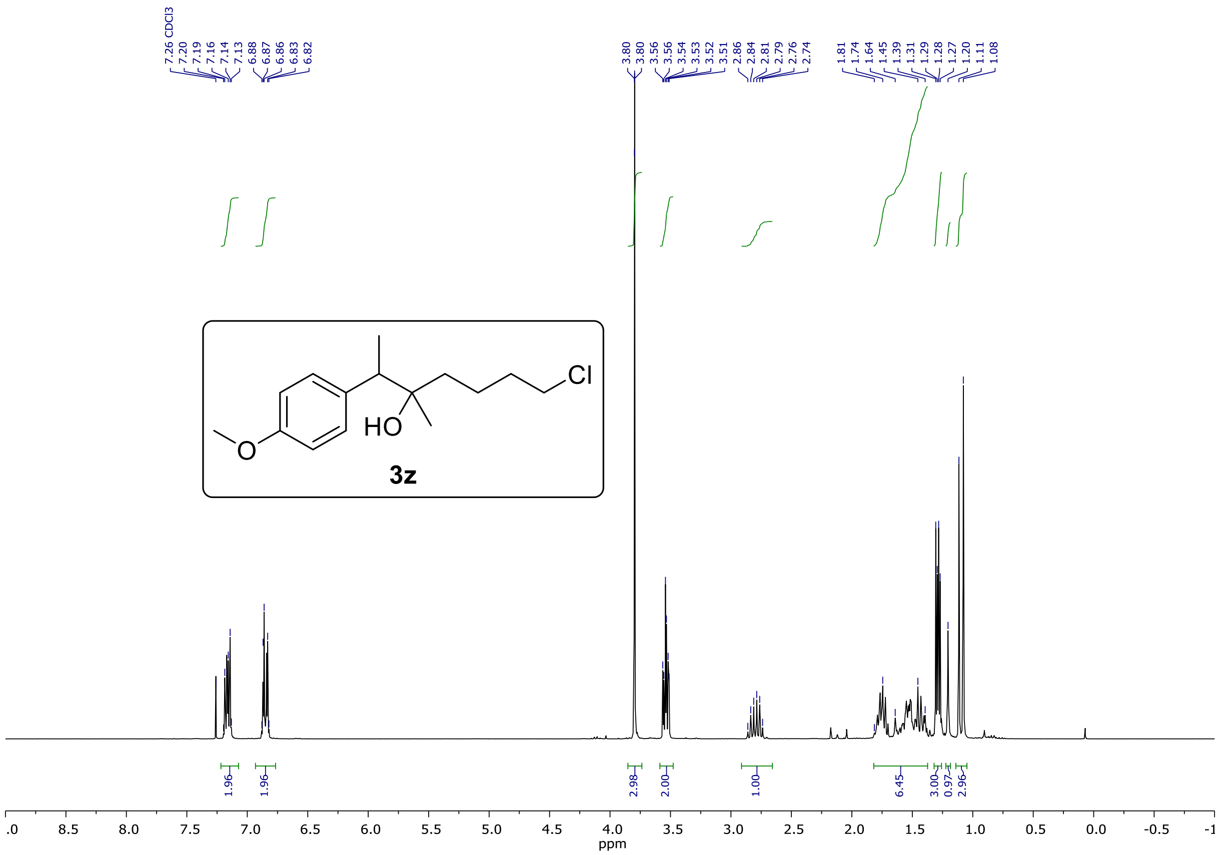

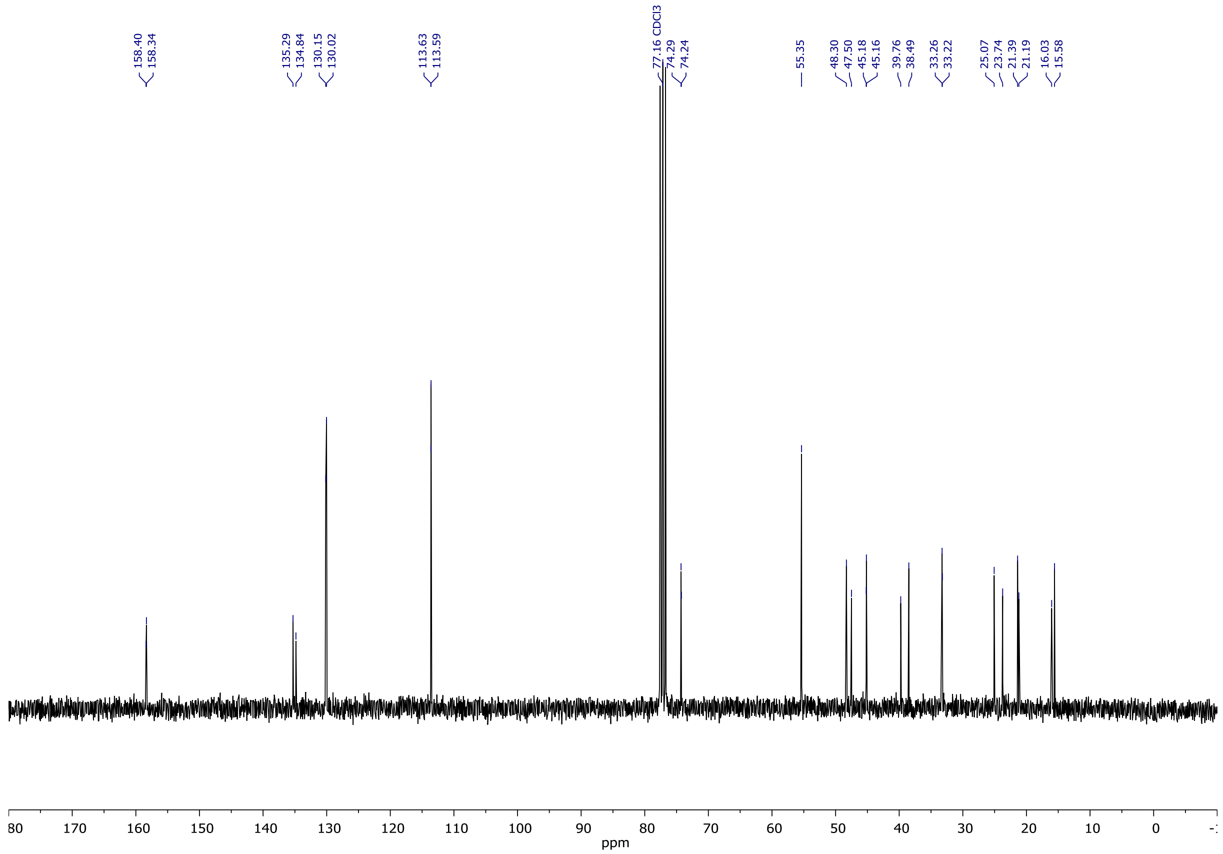

Compound **3aa**, ^{1}HNMR and ^{13}C NMR ($CDCl_3$):

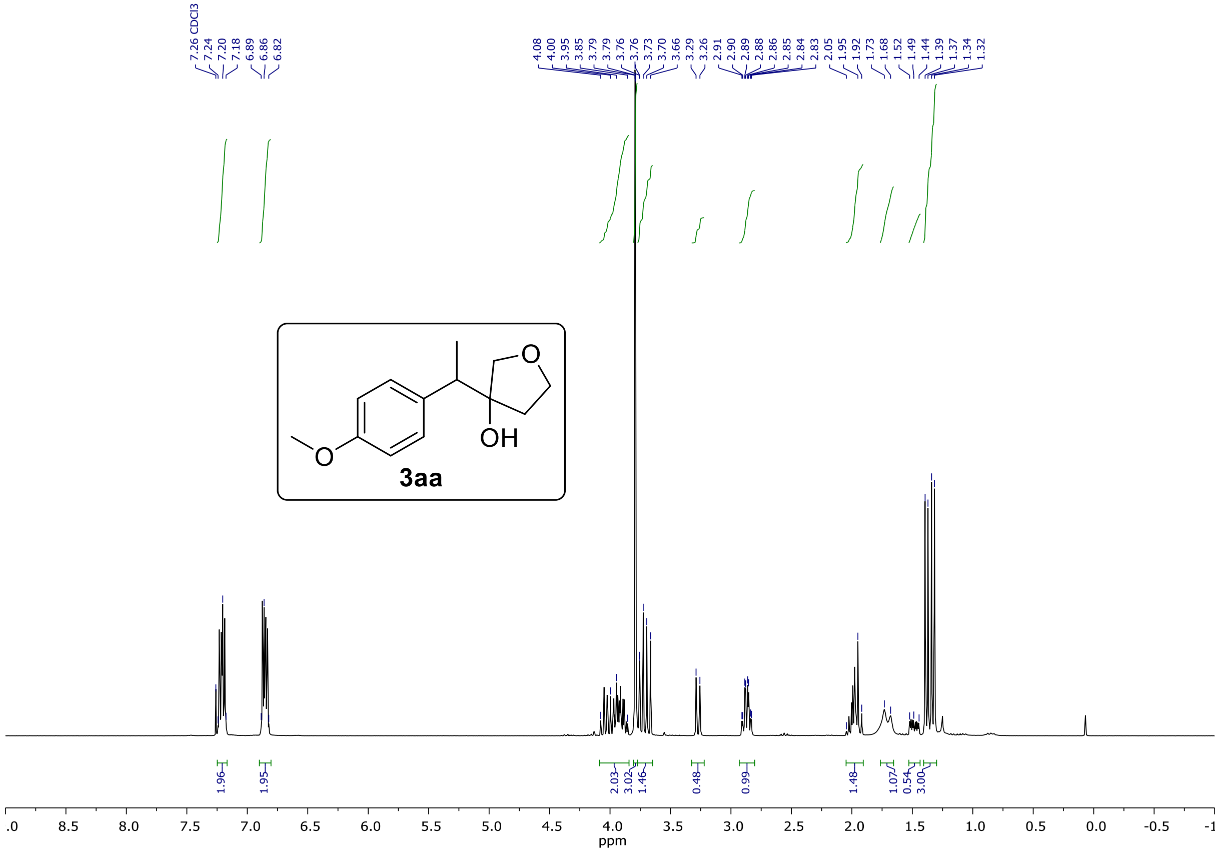

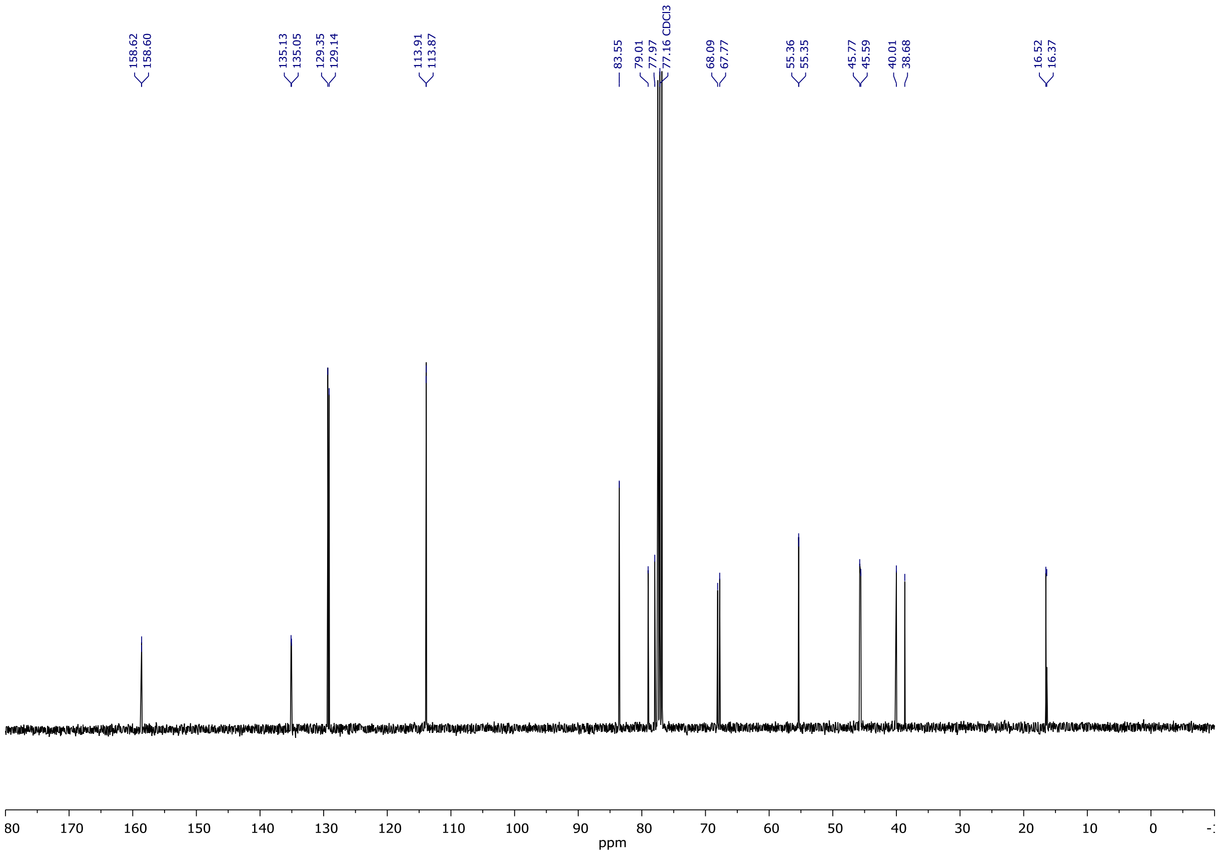

Compound **3ab**, ^{1}HNMR and ^{13}C NMR ($CDCl_3$):

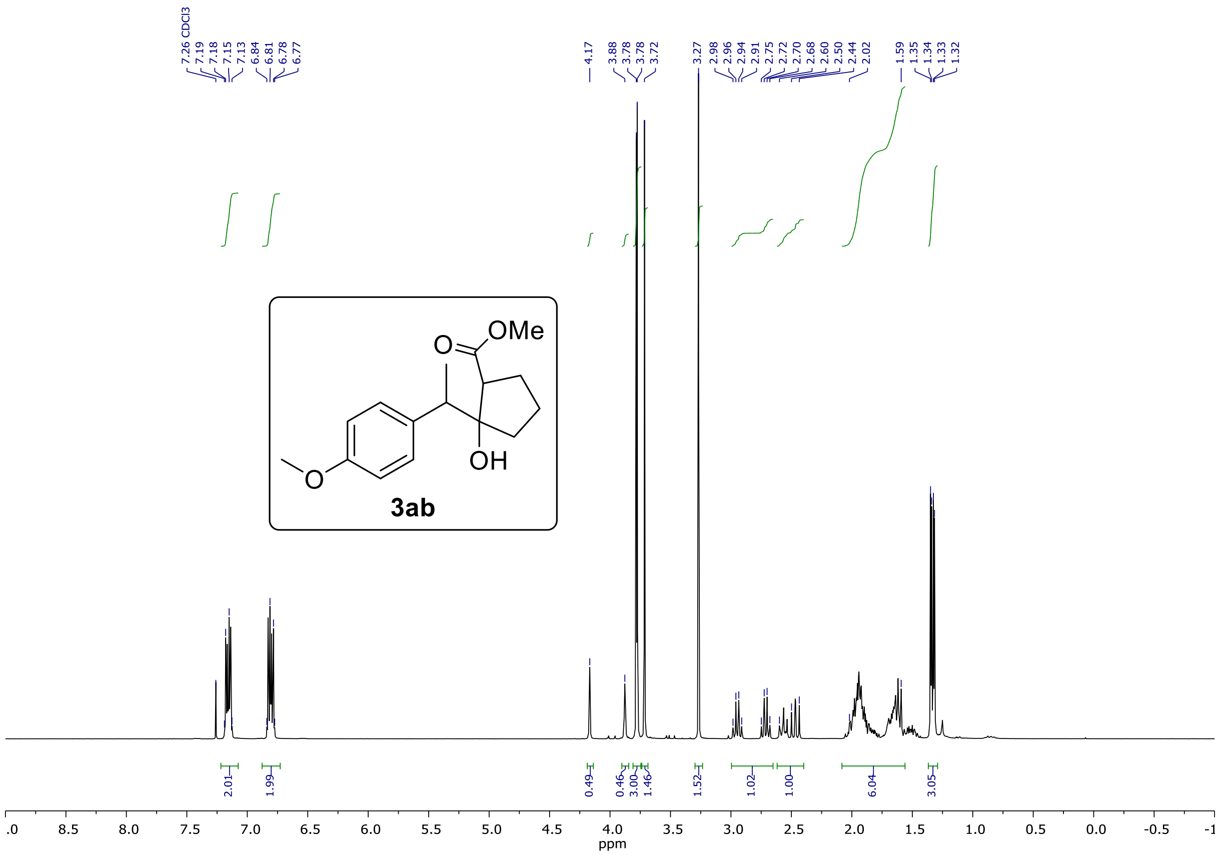

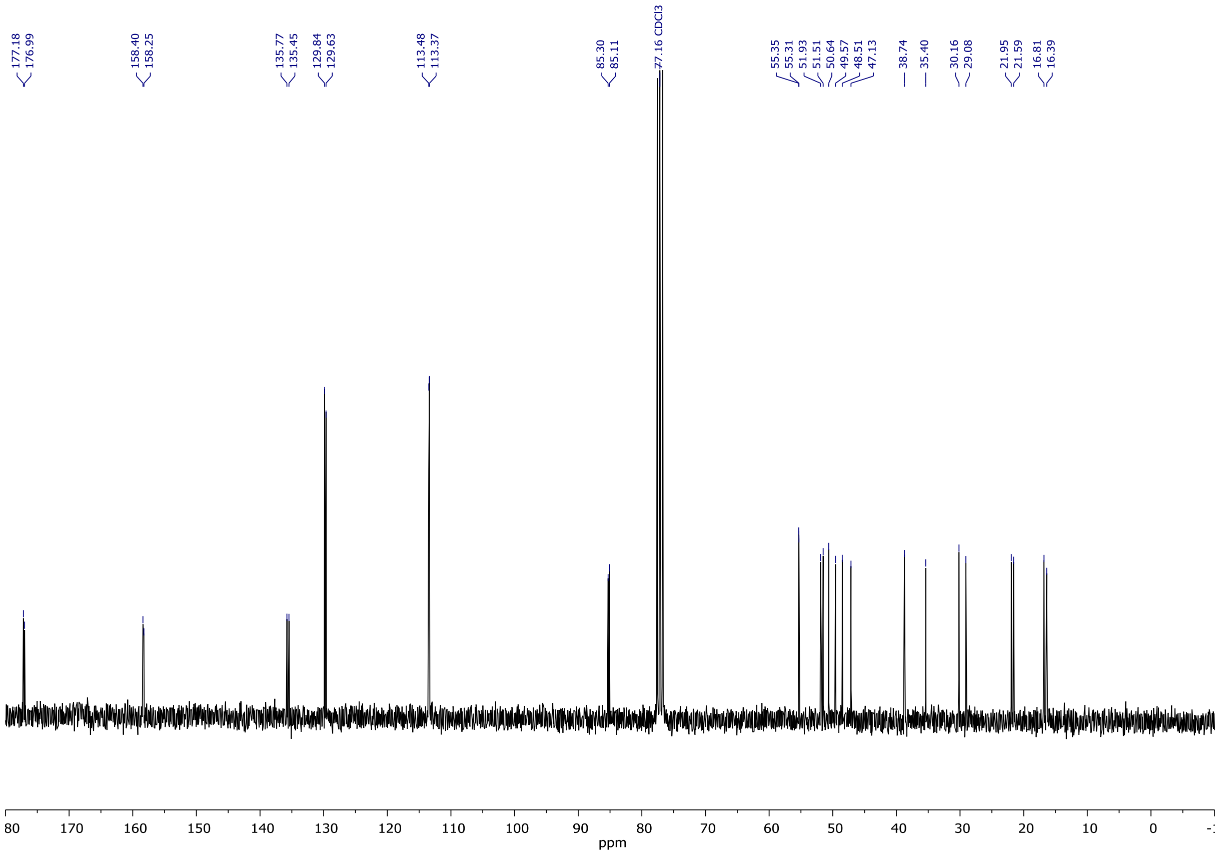

Compound **3ac**, ^{1}HNMR and ^{13}C NMR ($CDCl_3$):

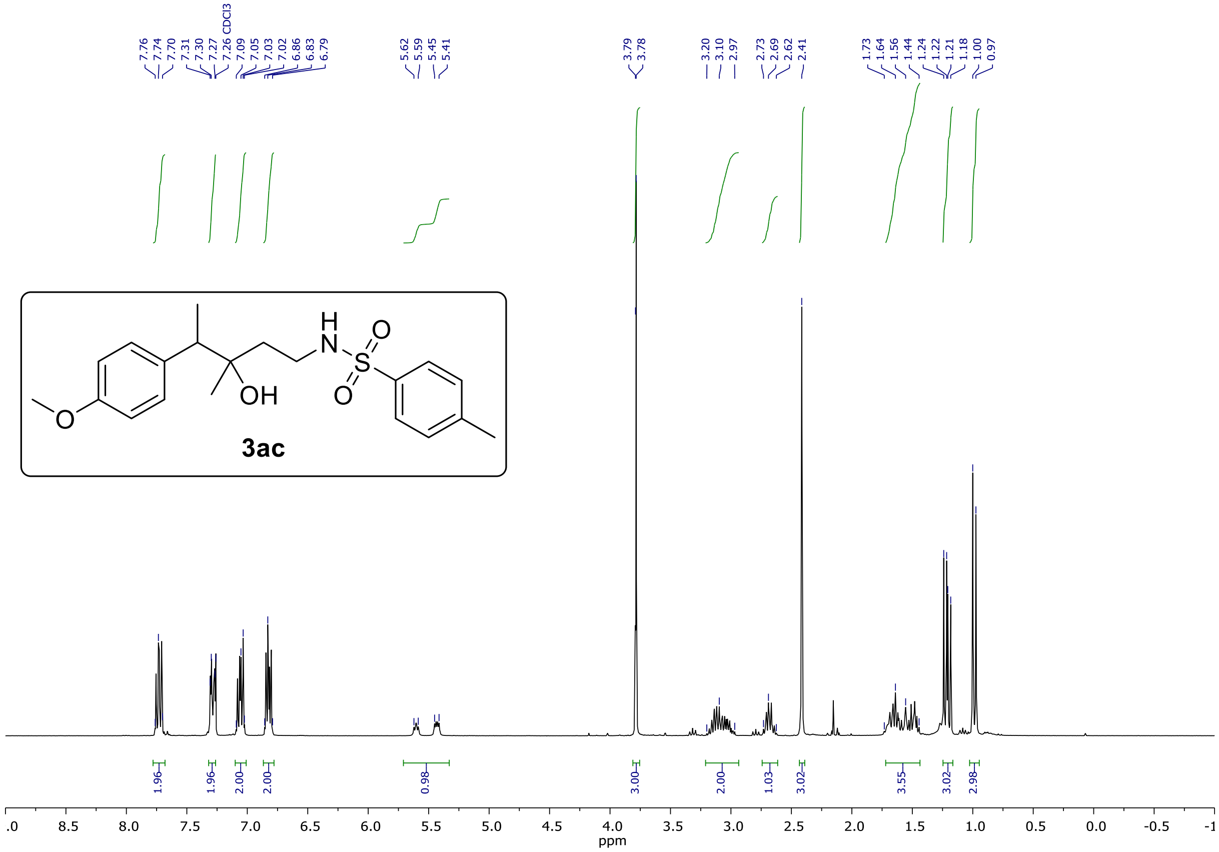

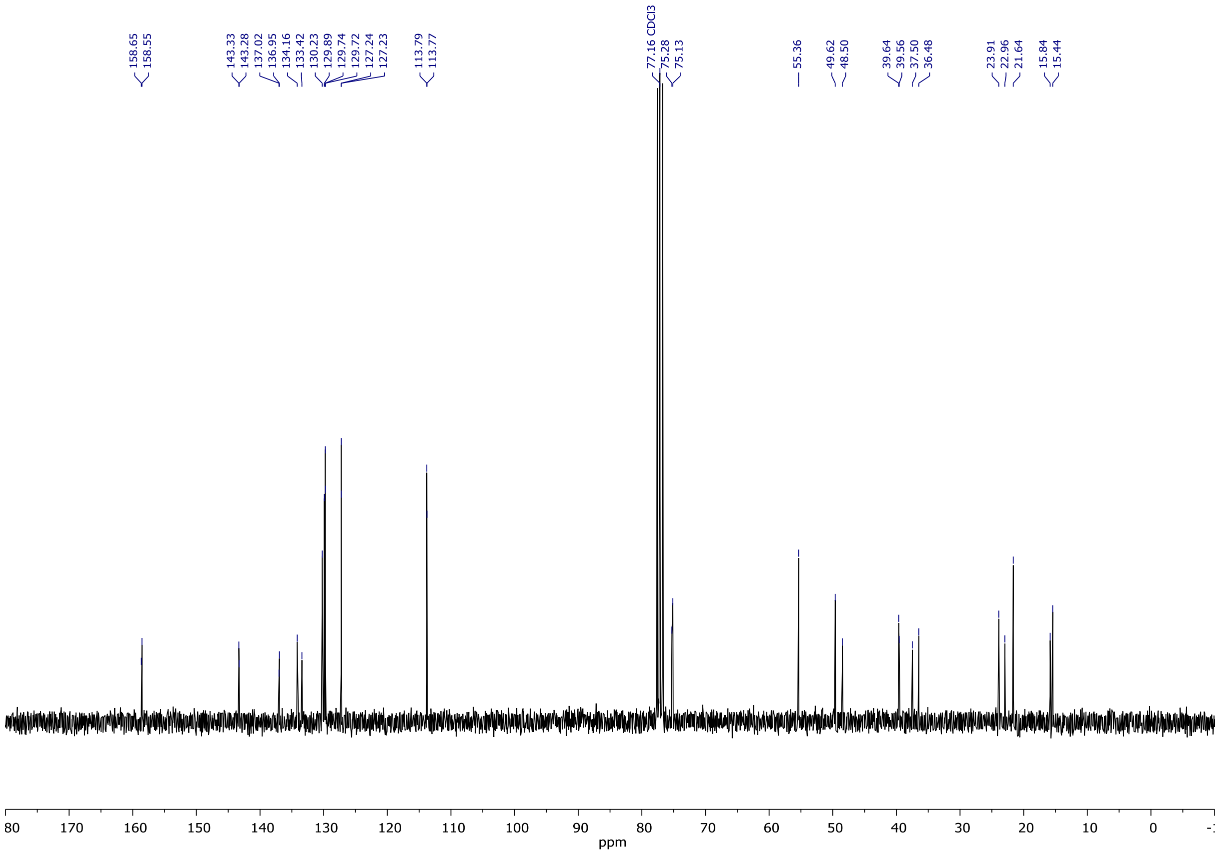

Compound **3ad**, ^{1}HNMR and ^{13}C NMR ($CDCl_3$):

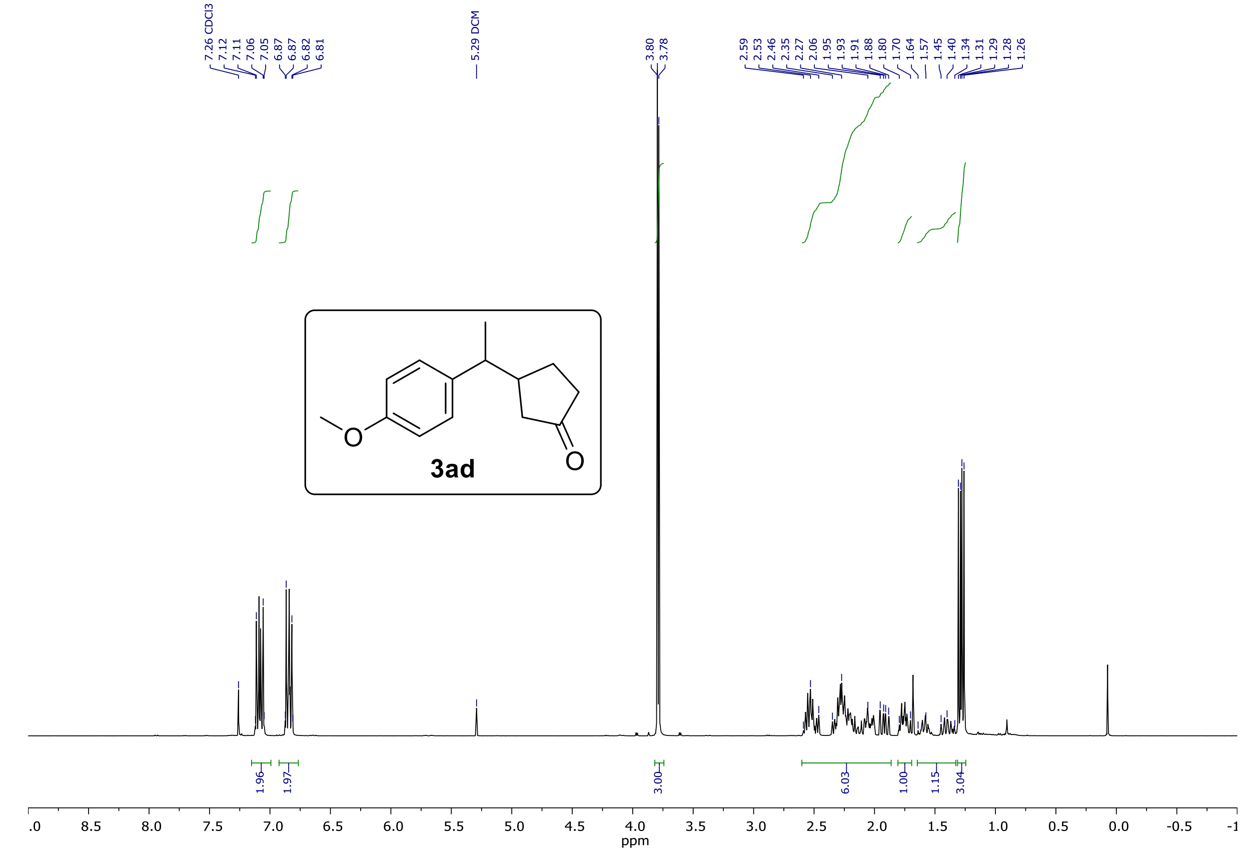

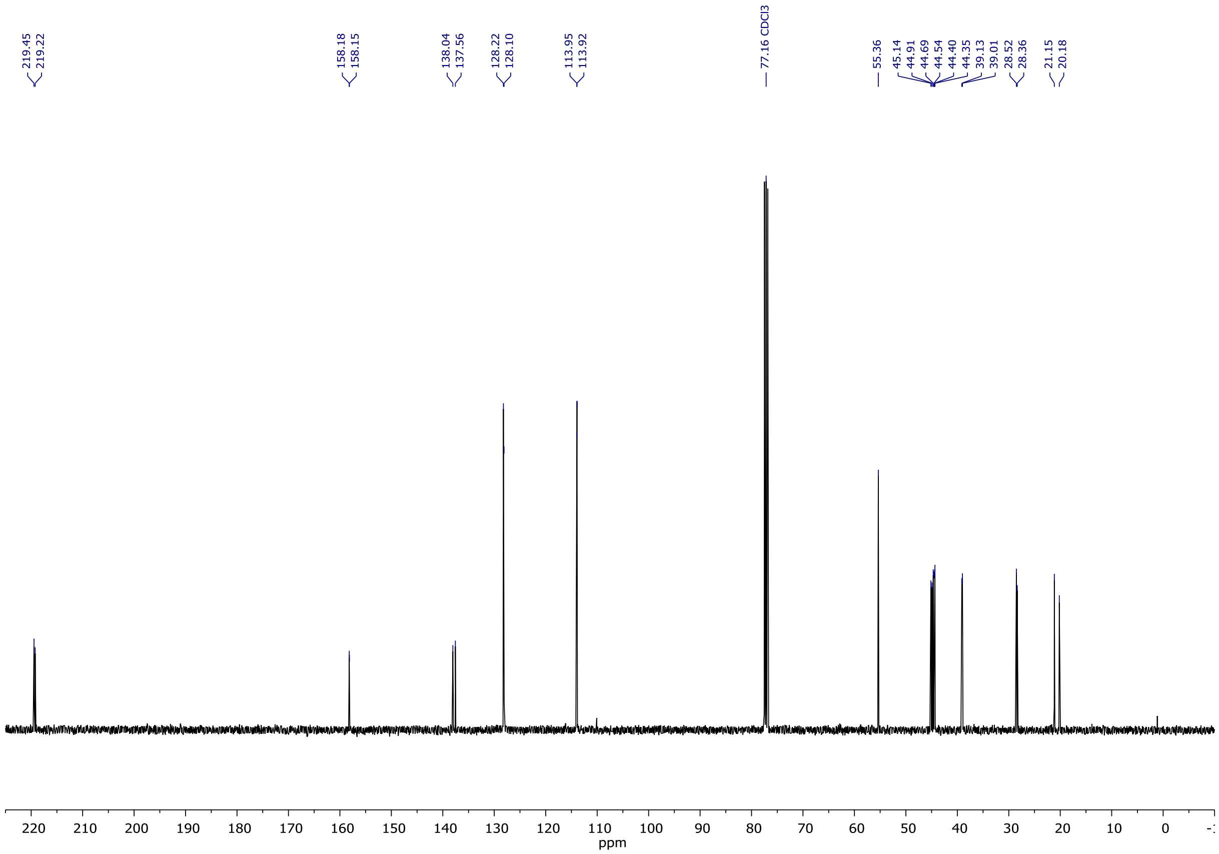

Compound **6a**, ¹HNMR and ¹³C NMR ($CDCl_3$):

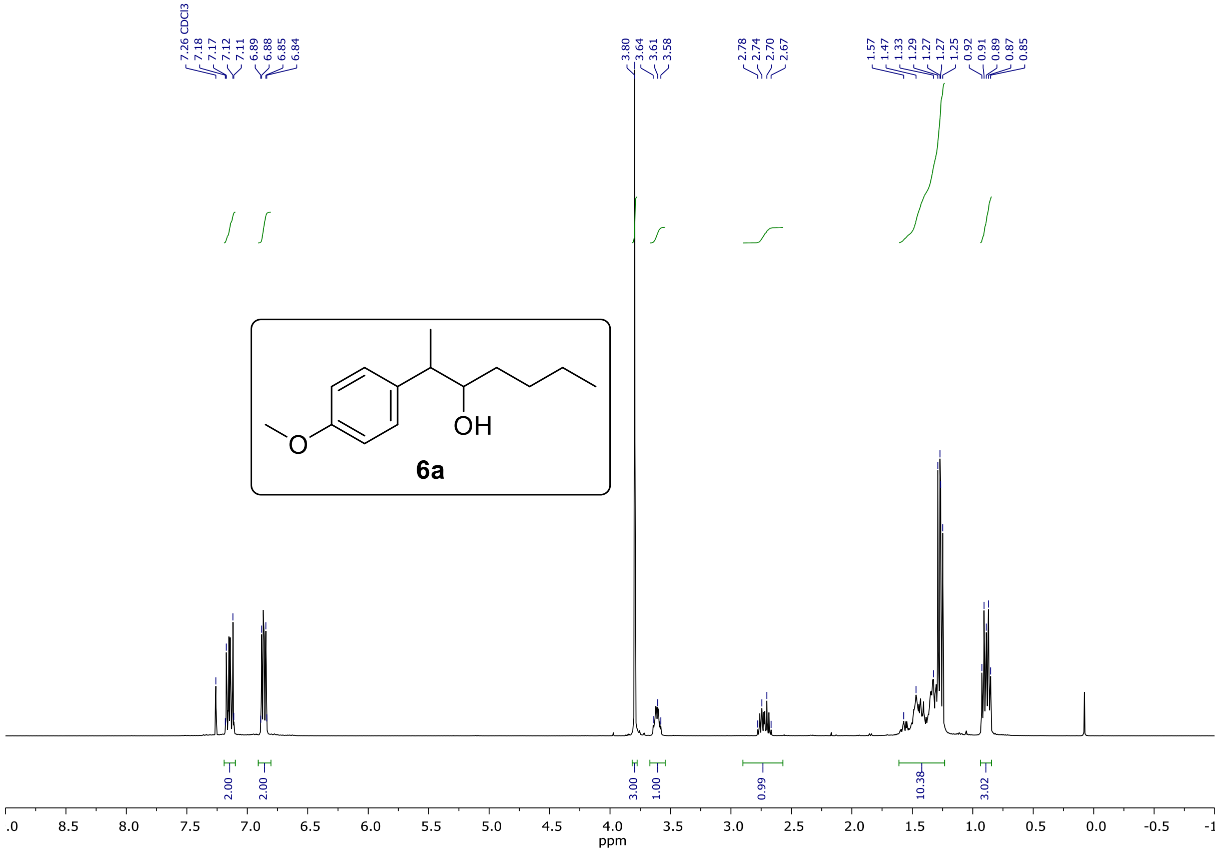

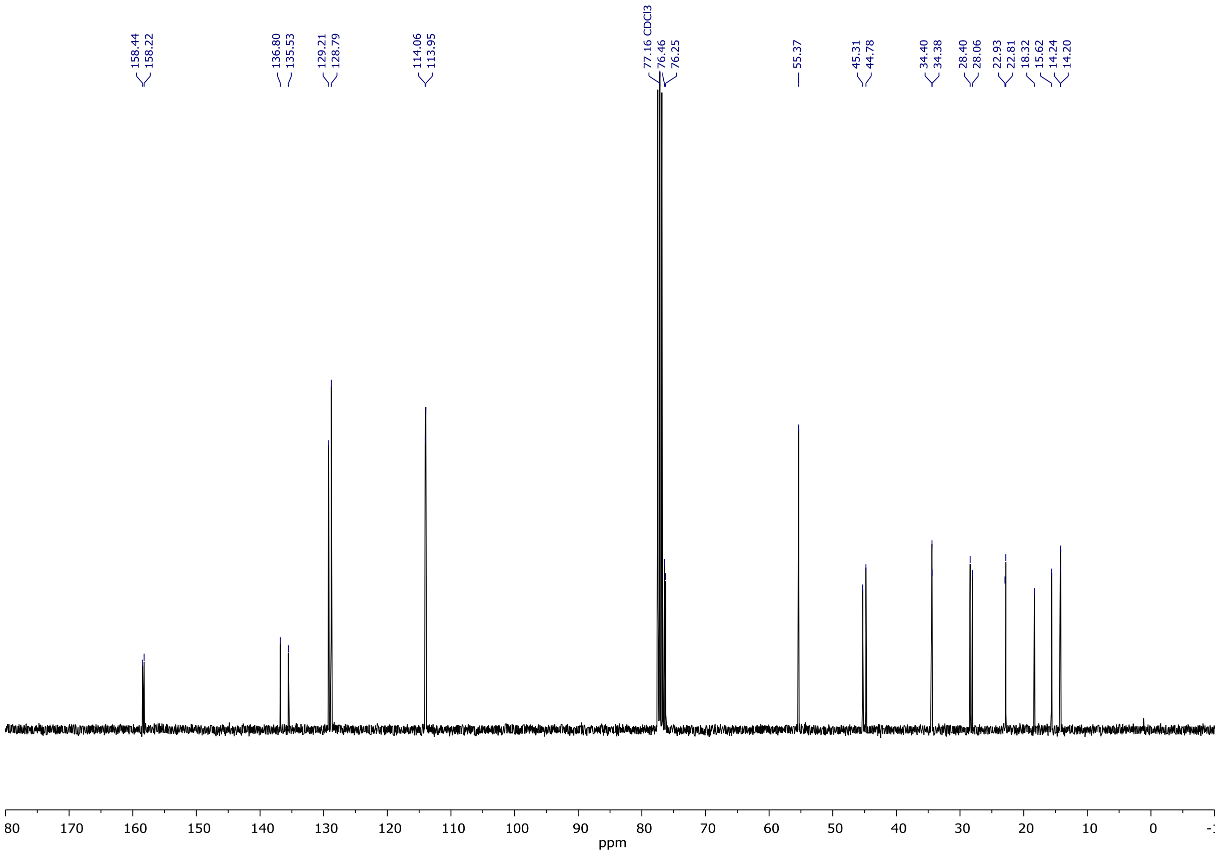

Compound **6b**, ^{1}HNMR and ^{13}C NMR ($CDCl_3$):

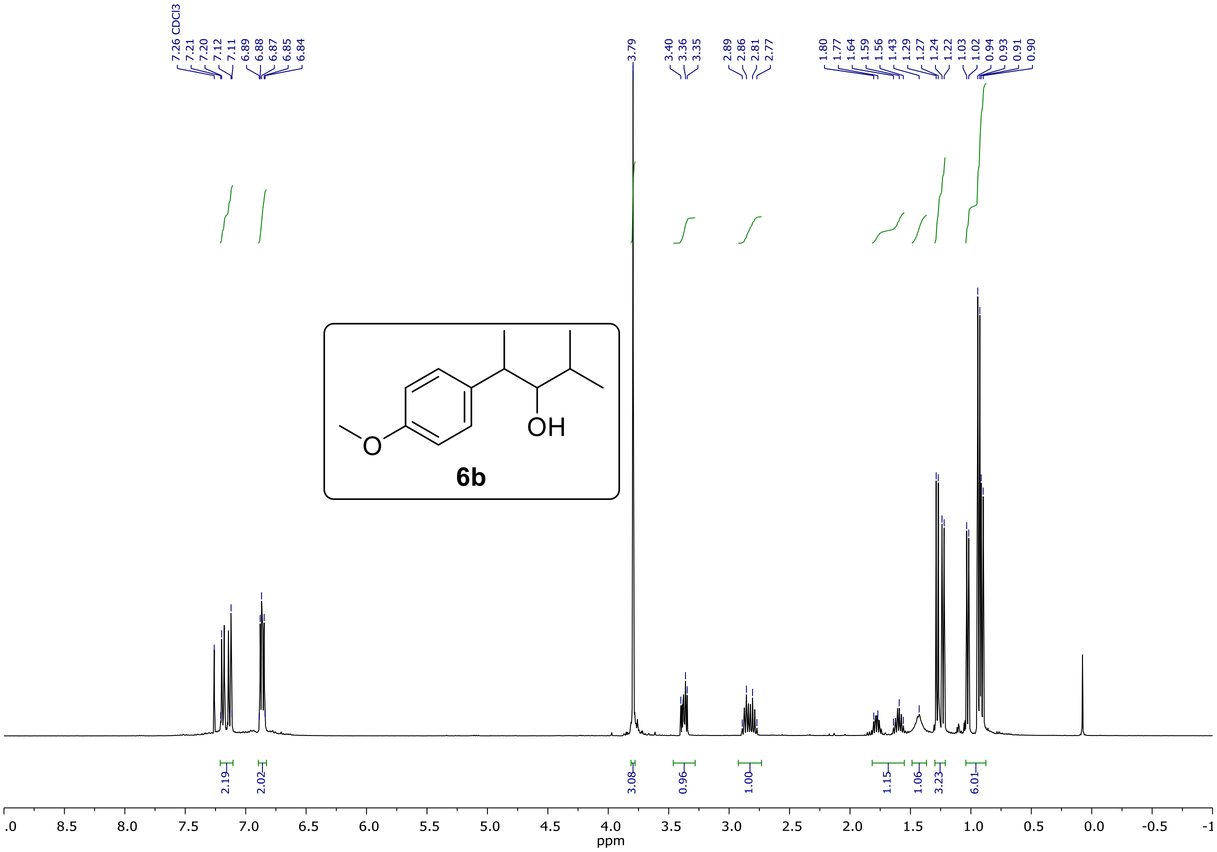

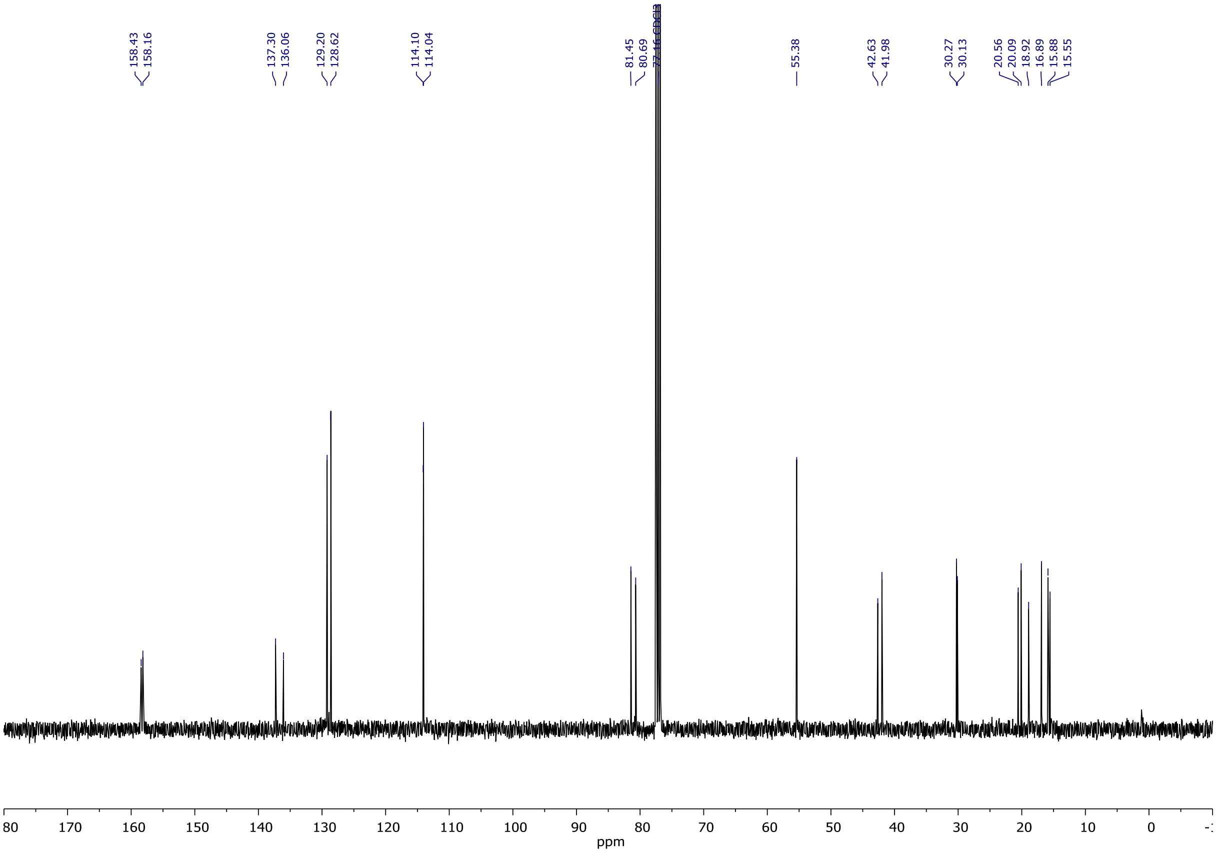

Compound **6c**, ^{1}HNMR and ^{13}C NMR ($CDCl_3$):

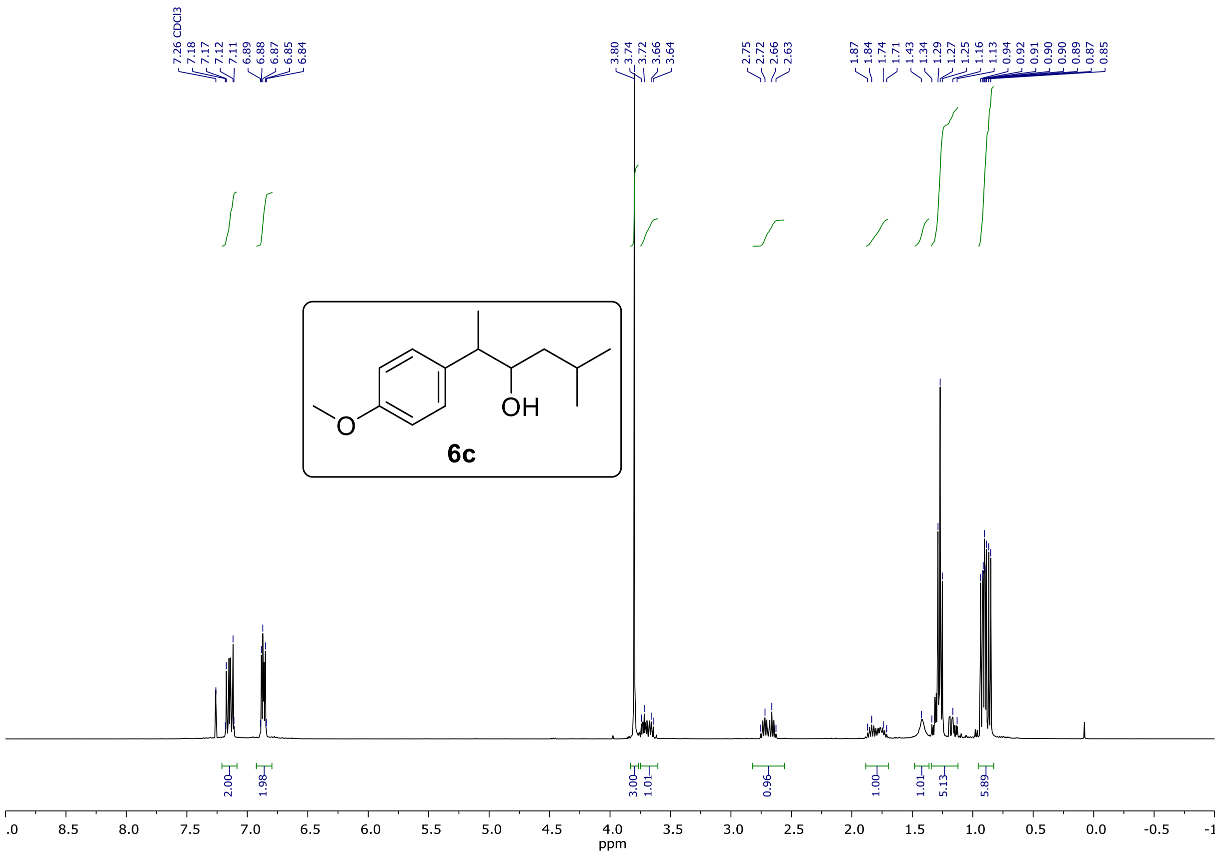

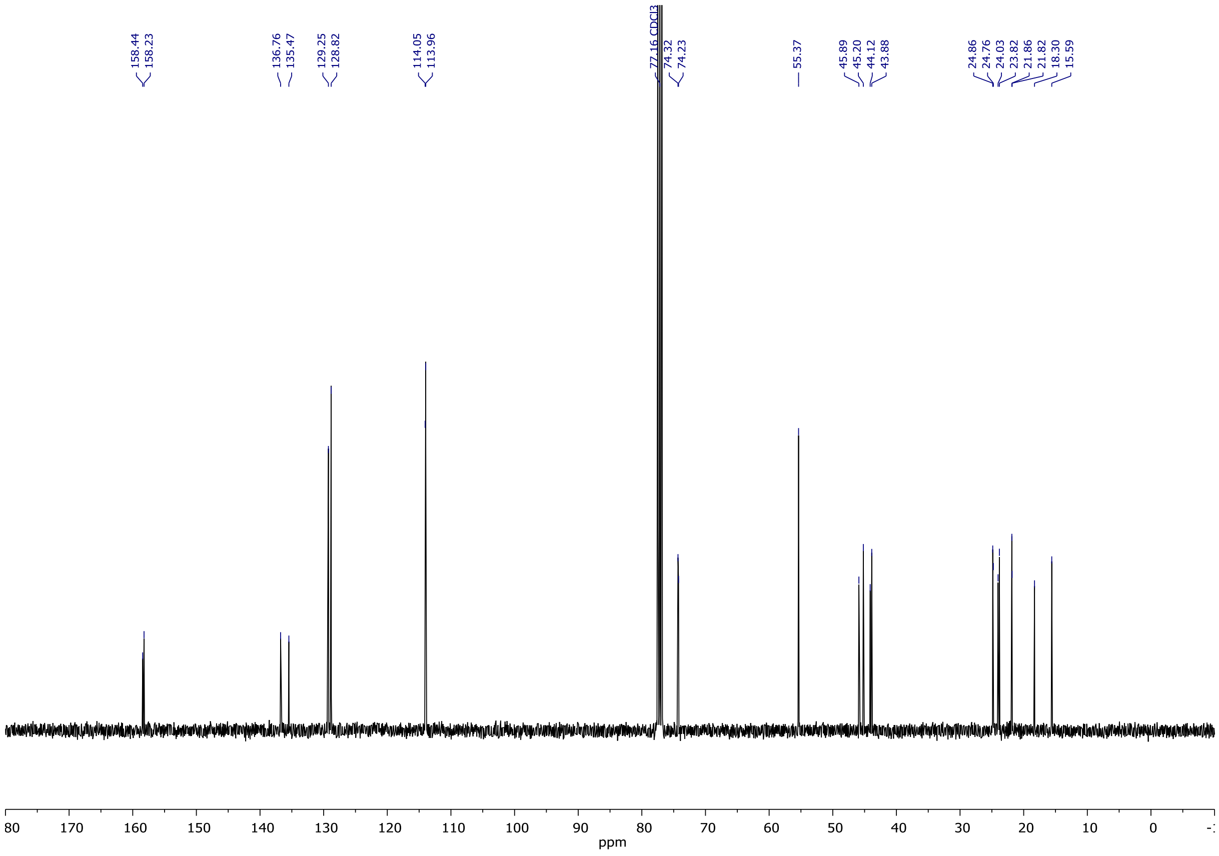

Compound **6d**, ^{1}HNMR and ^{13}C NMR ($CDCl_3$):

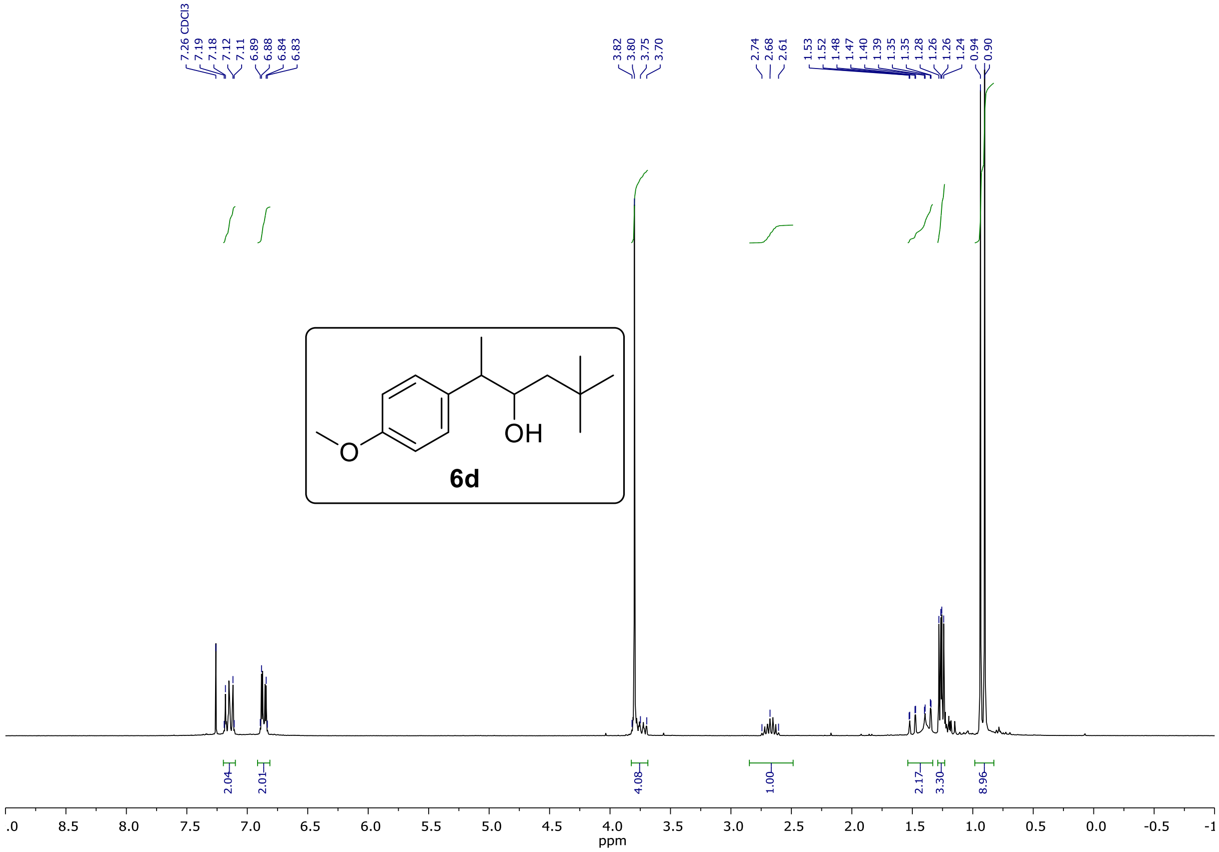

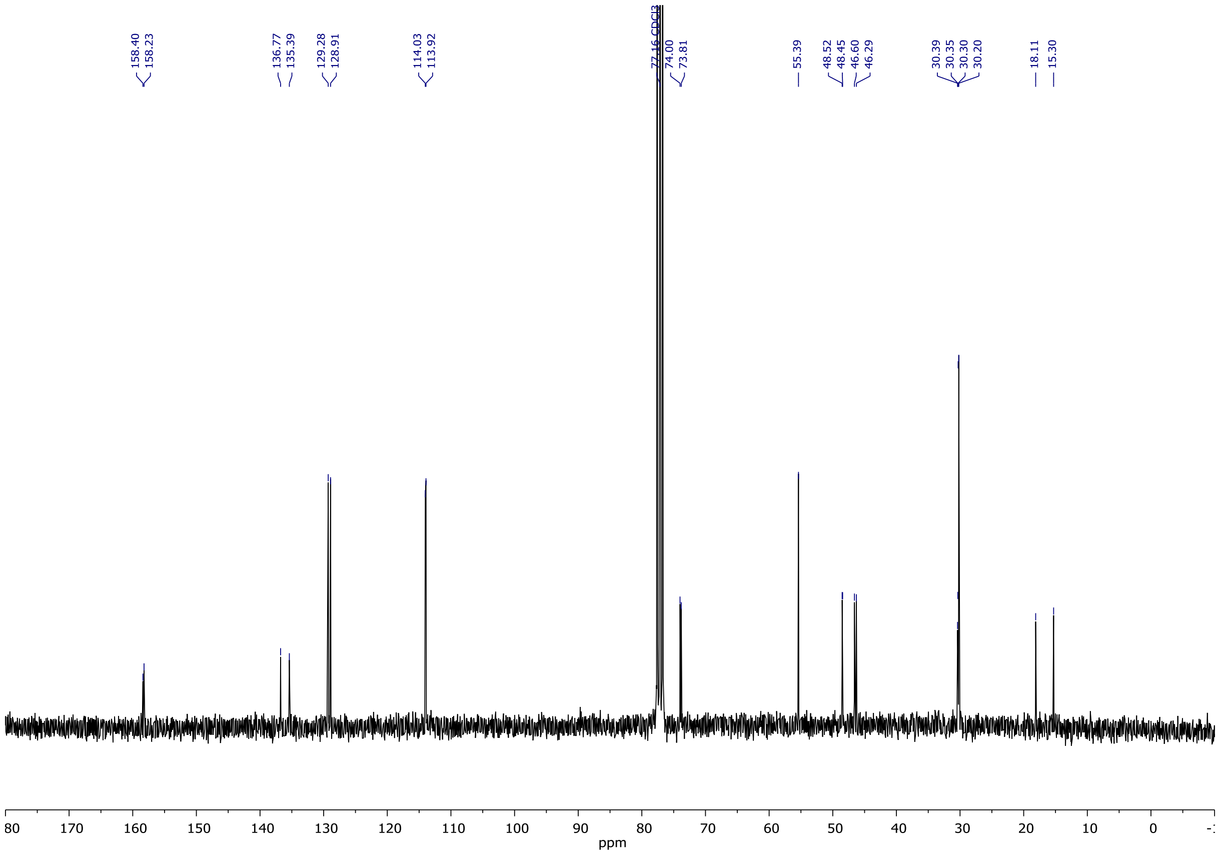

Compound **6e**, ^{1}HNMR and ^{13}C NMR ($CDCl_3$):

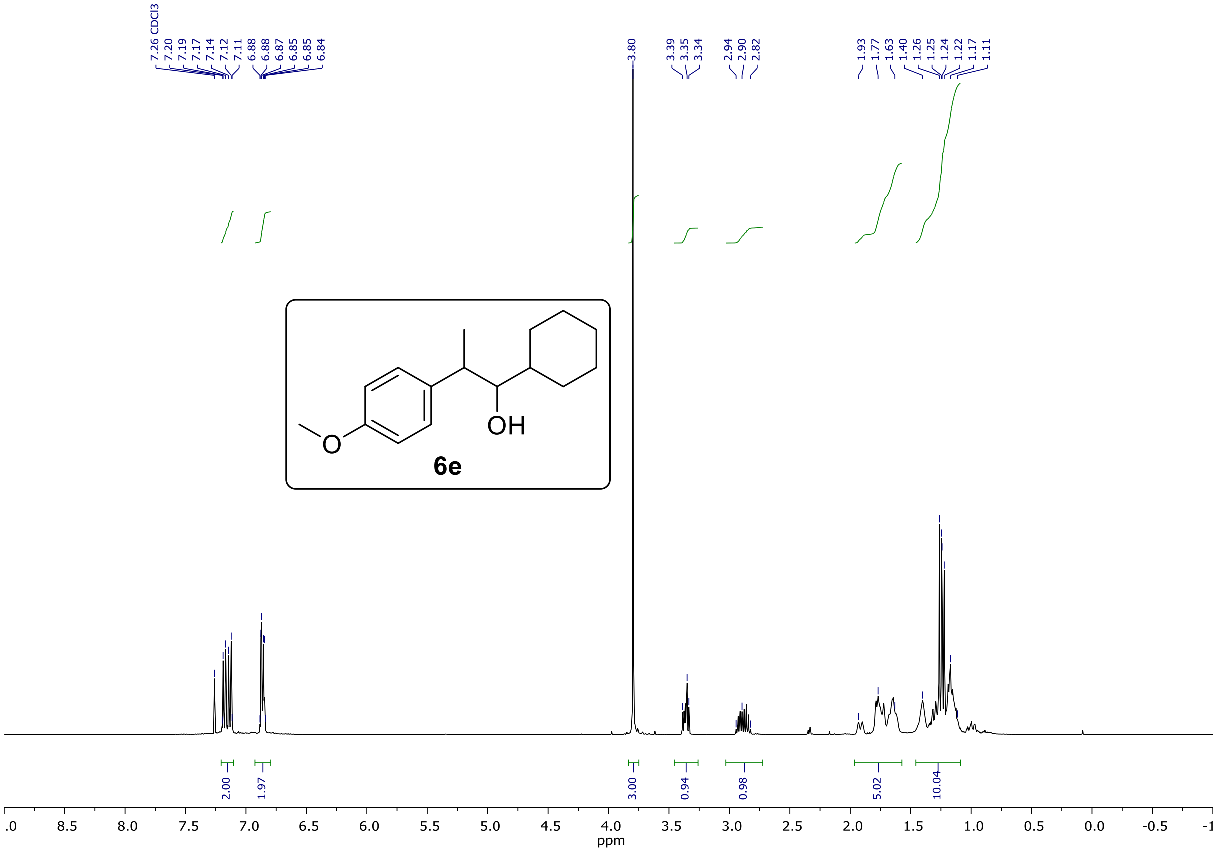

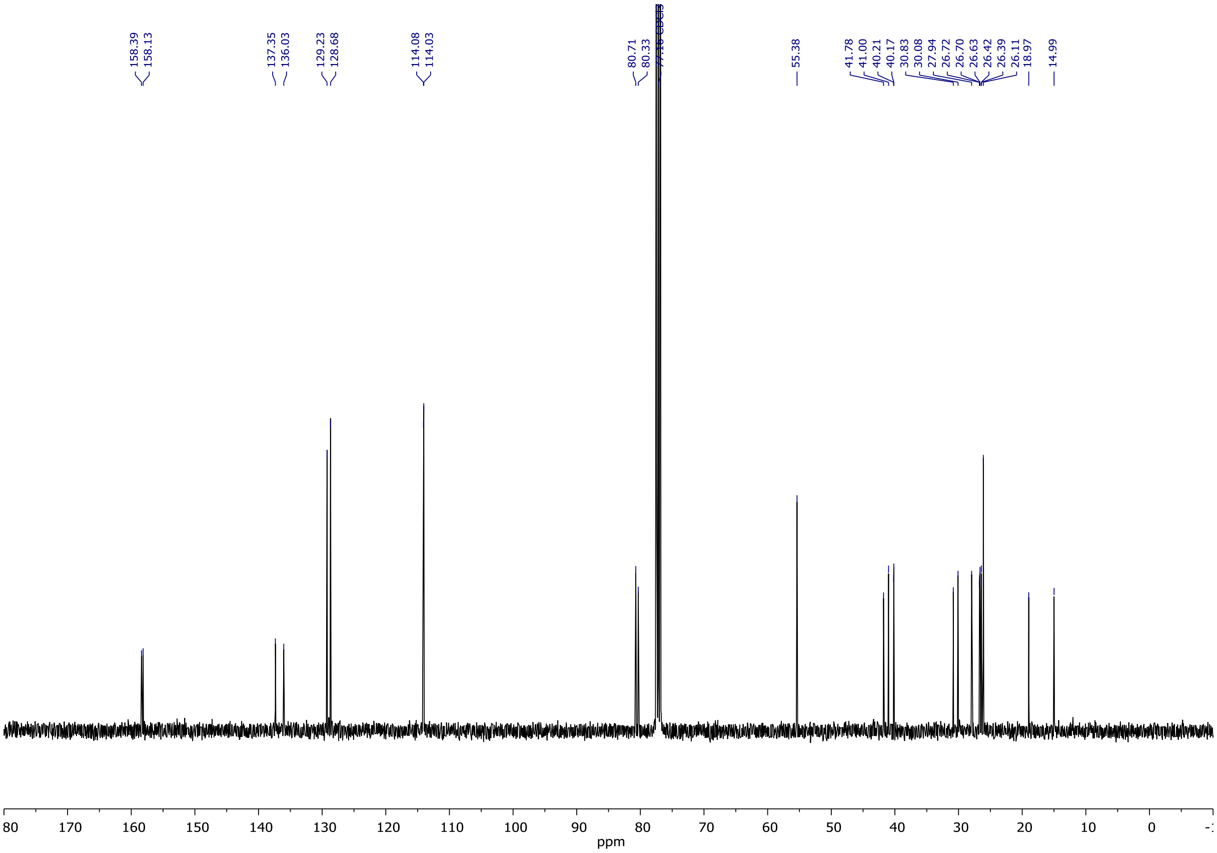

Compound **6f**, ^{1}HNMR and ^{13}C NMR ($CDCl_3$):

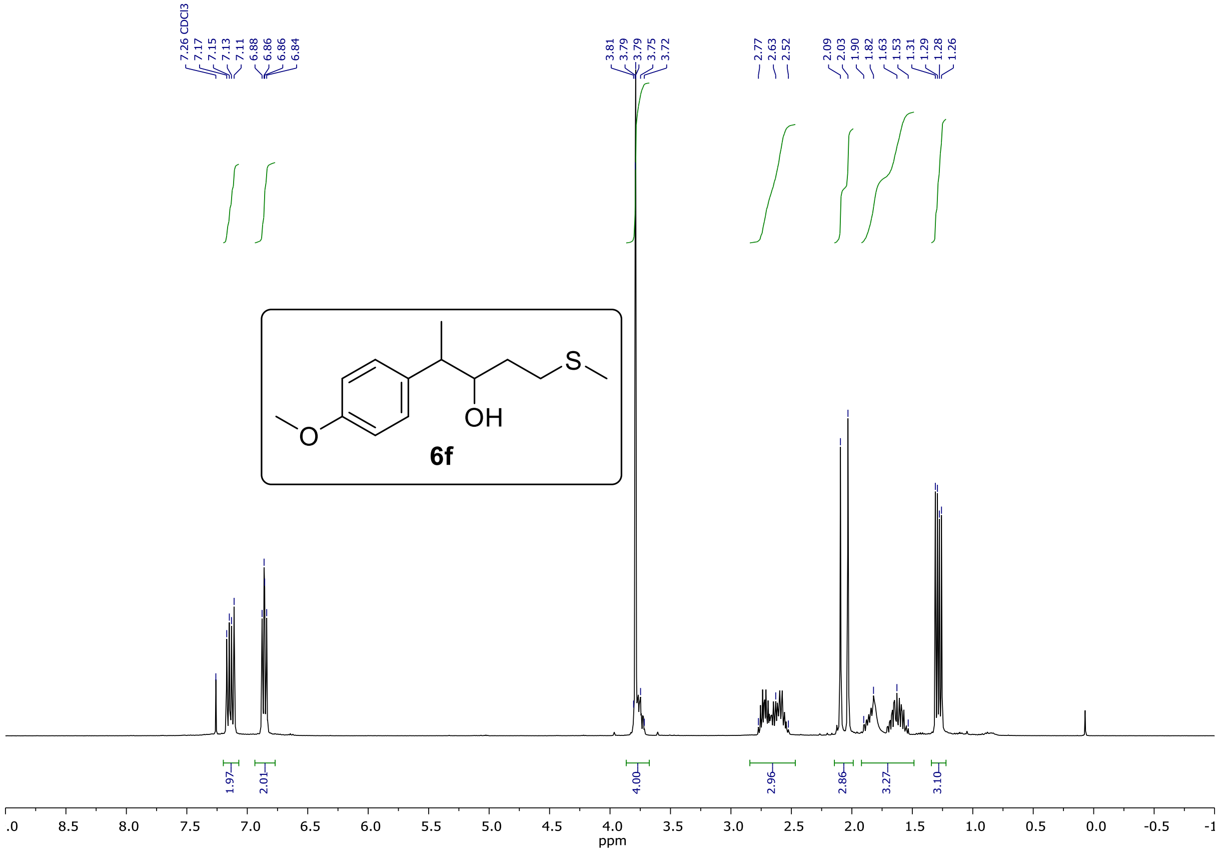

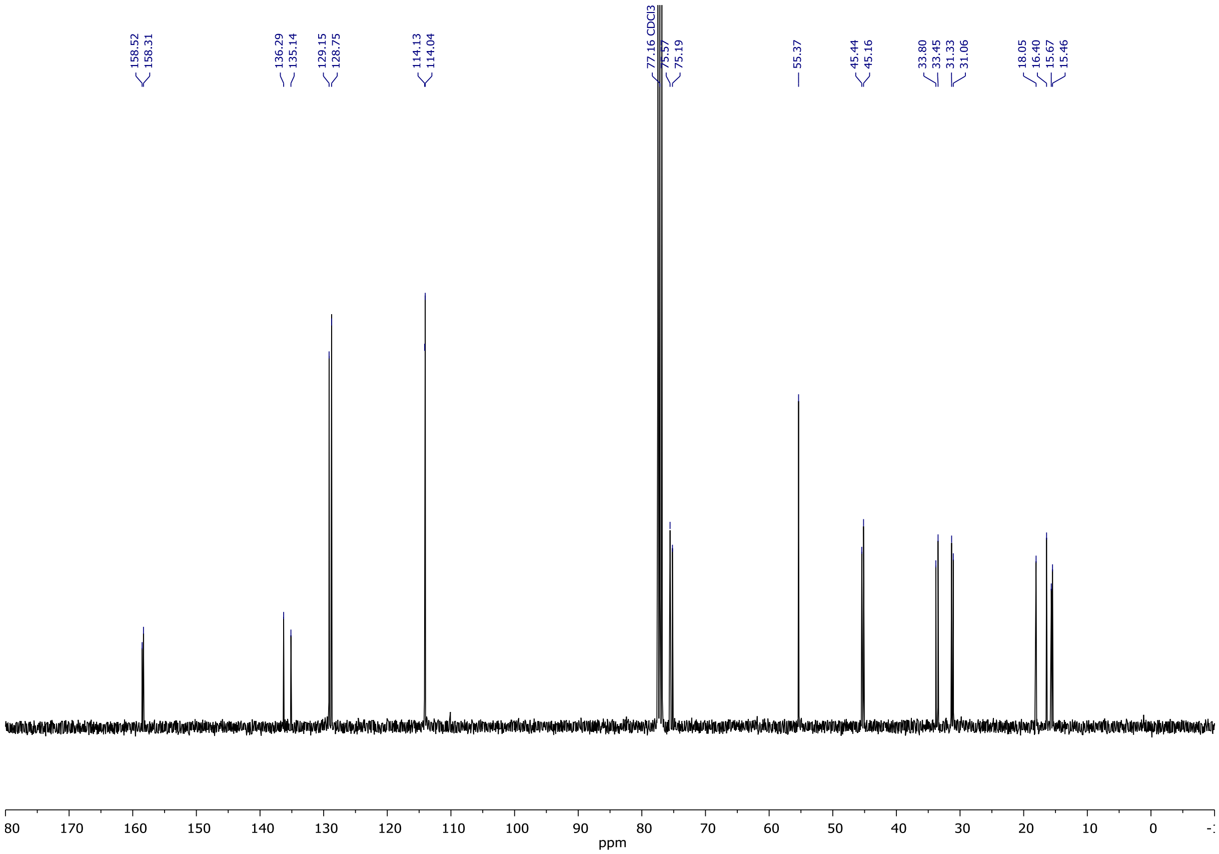

Compound **6g**, ^{1}HNMR and ^{13}C NMR ($CDCl_3$):

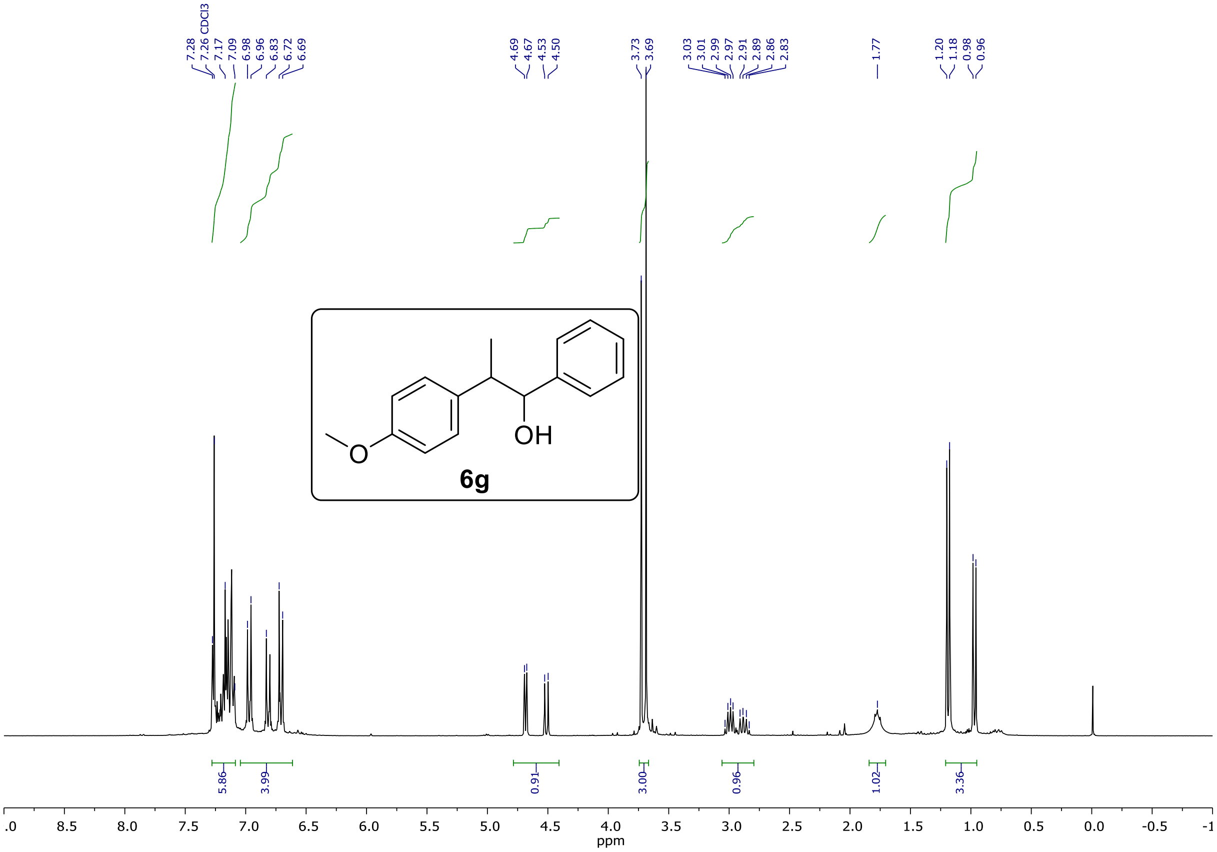

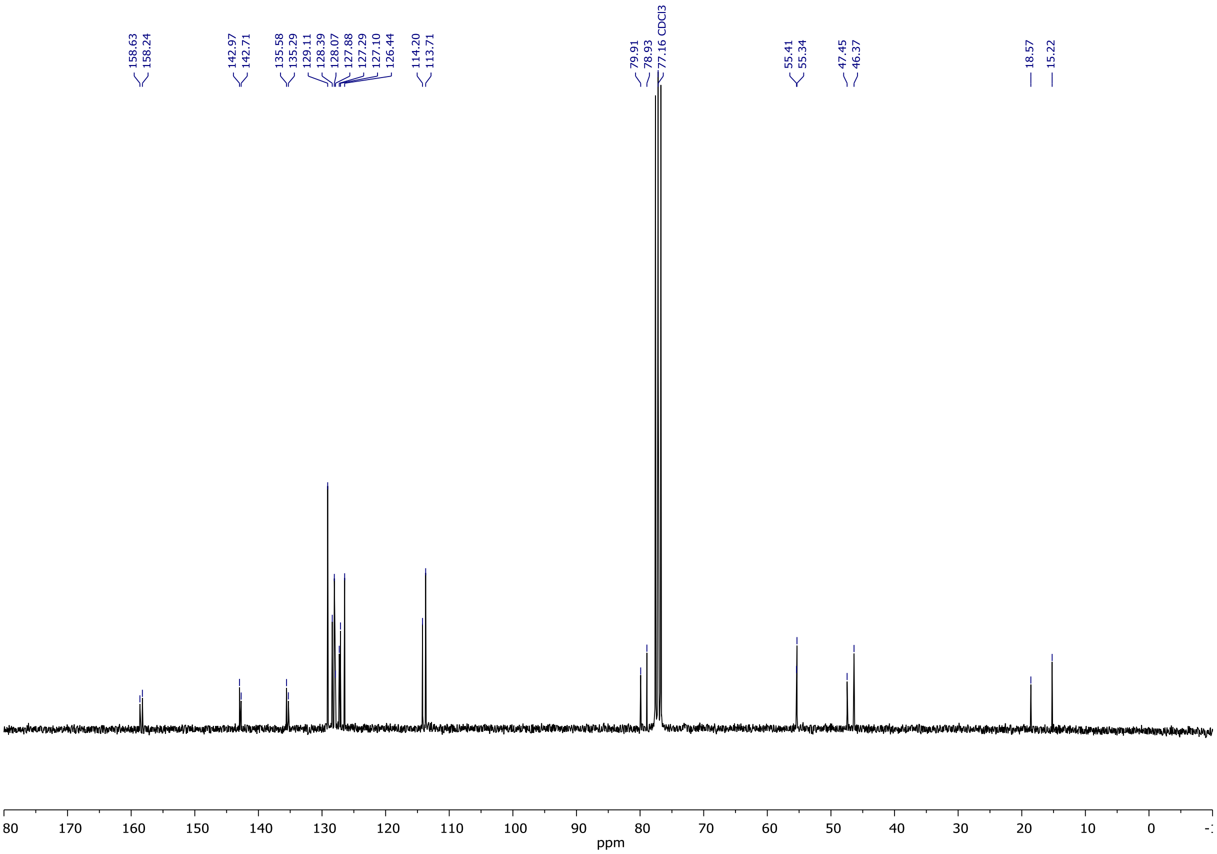

Compound **6h**, ^{1}HNMR and ^{13}C NMR ($CDCl_3$):

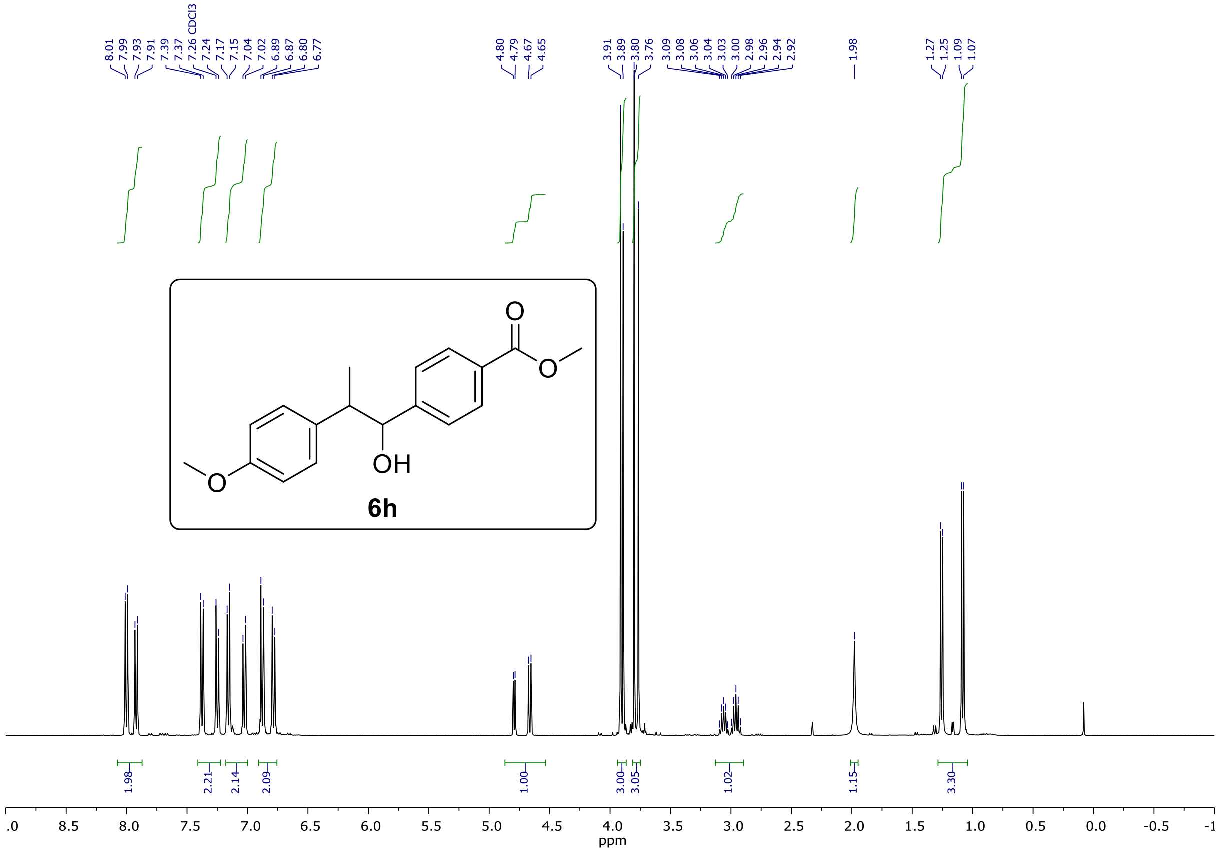

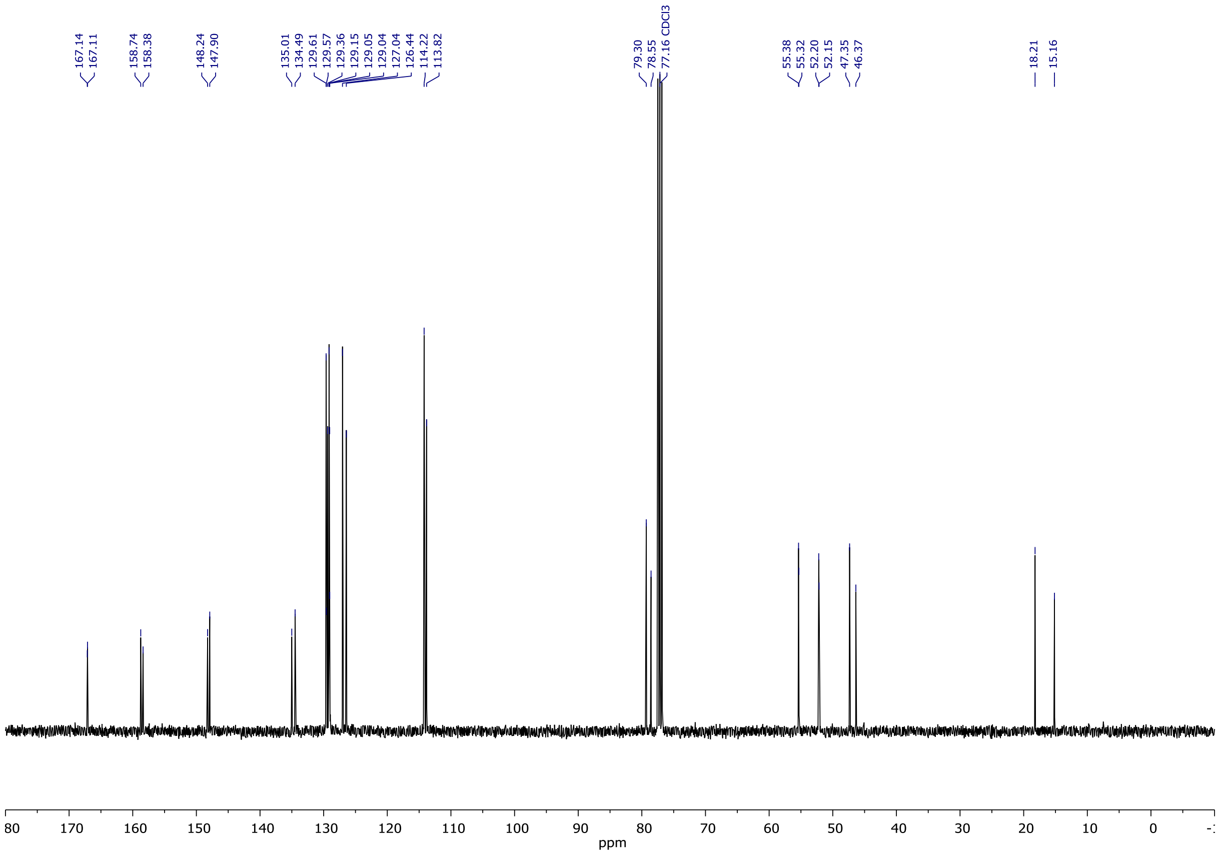

Compound **6i**, ^{1}HNMR and ^{13}C NMR ($CDCl_3$):

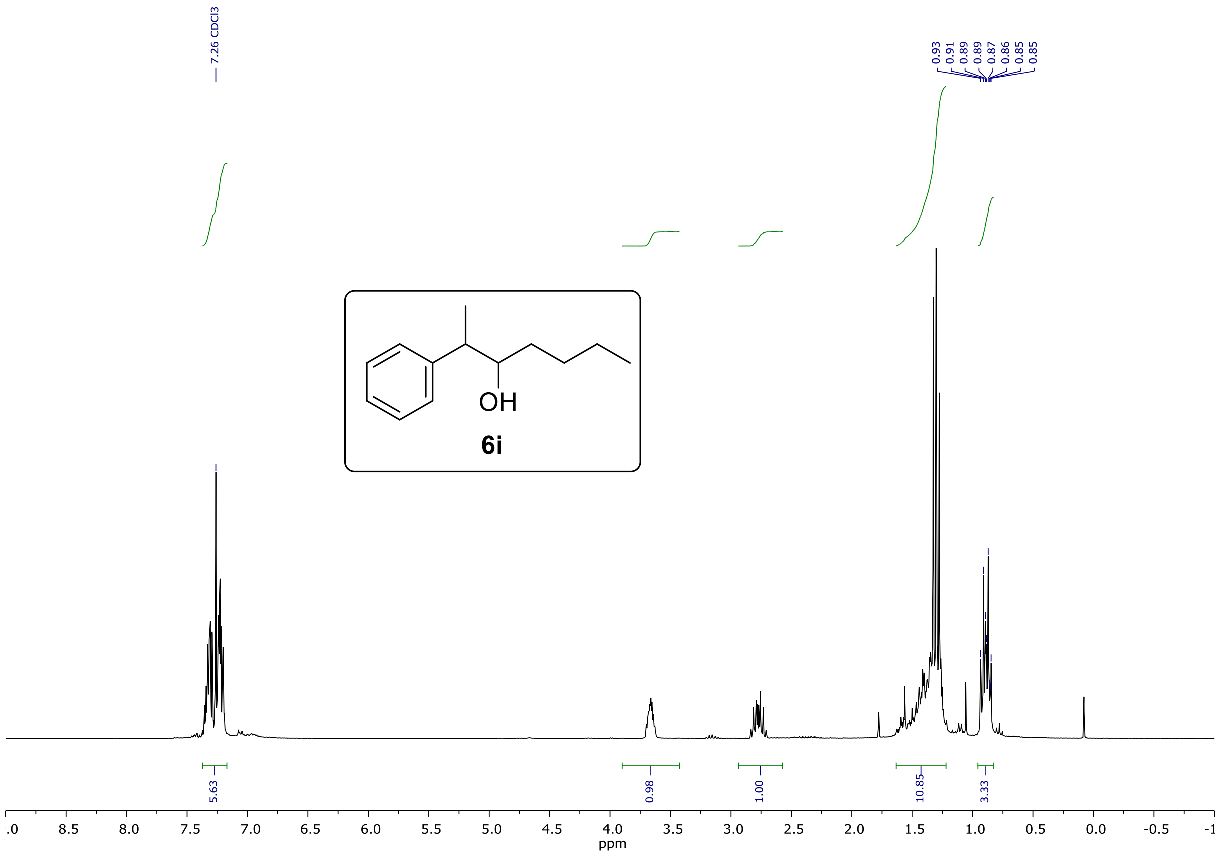

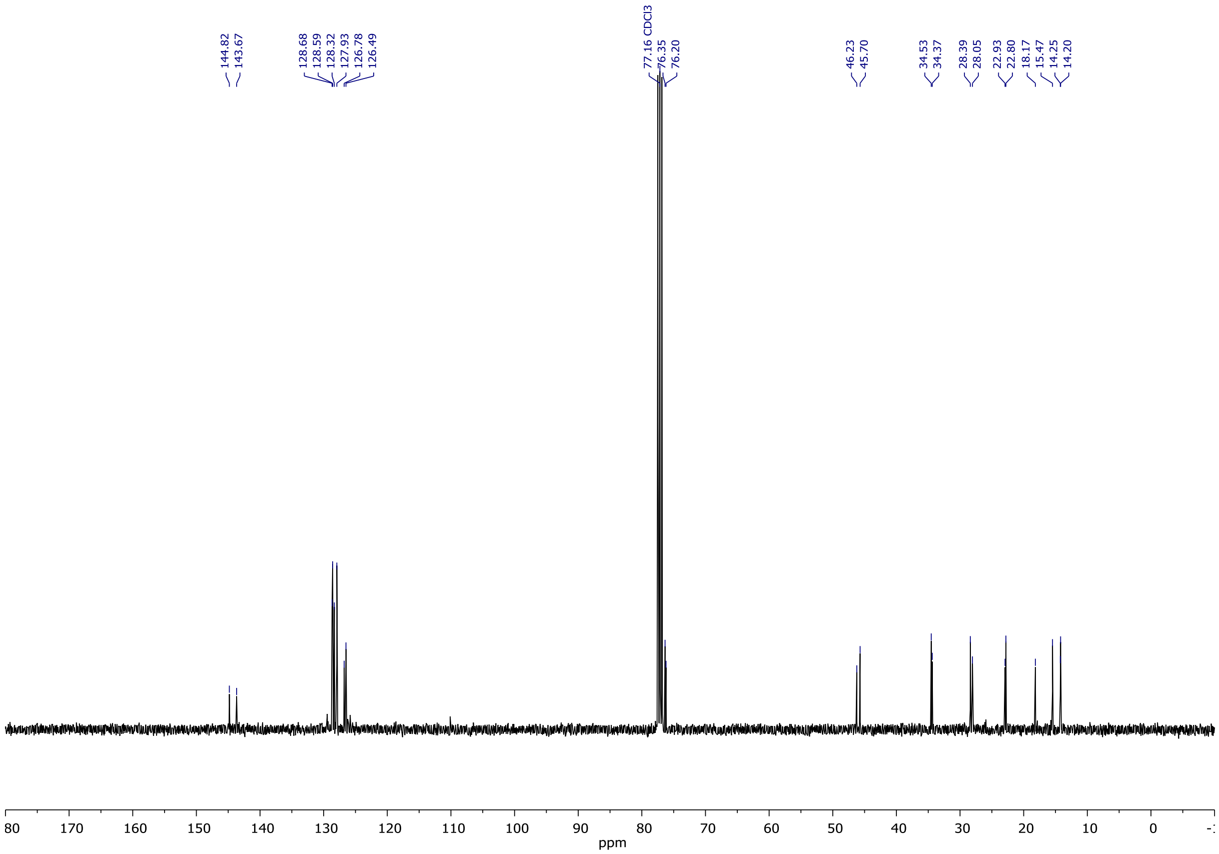

Compound **8a**, ^{1}HNMR and ^{13}C NMR ($CDCl_3$):

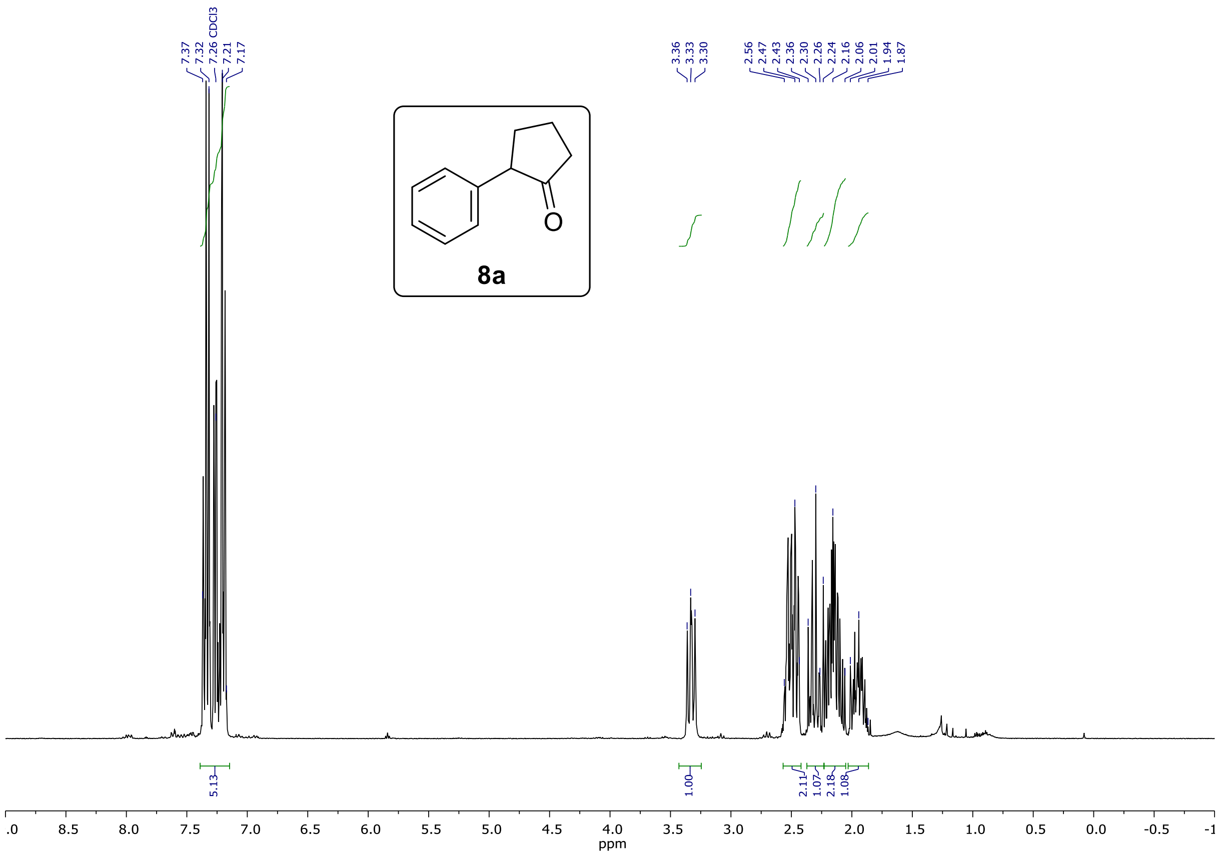

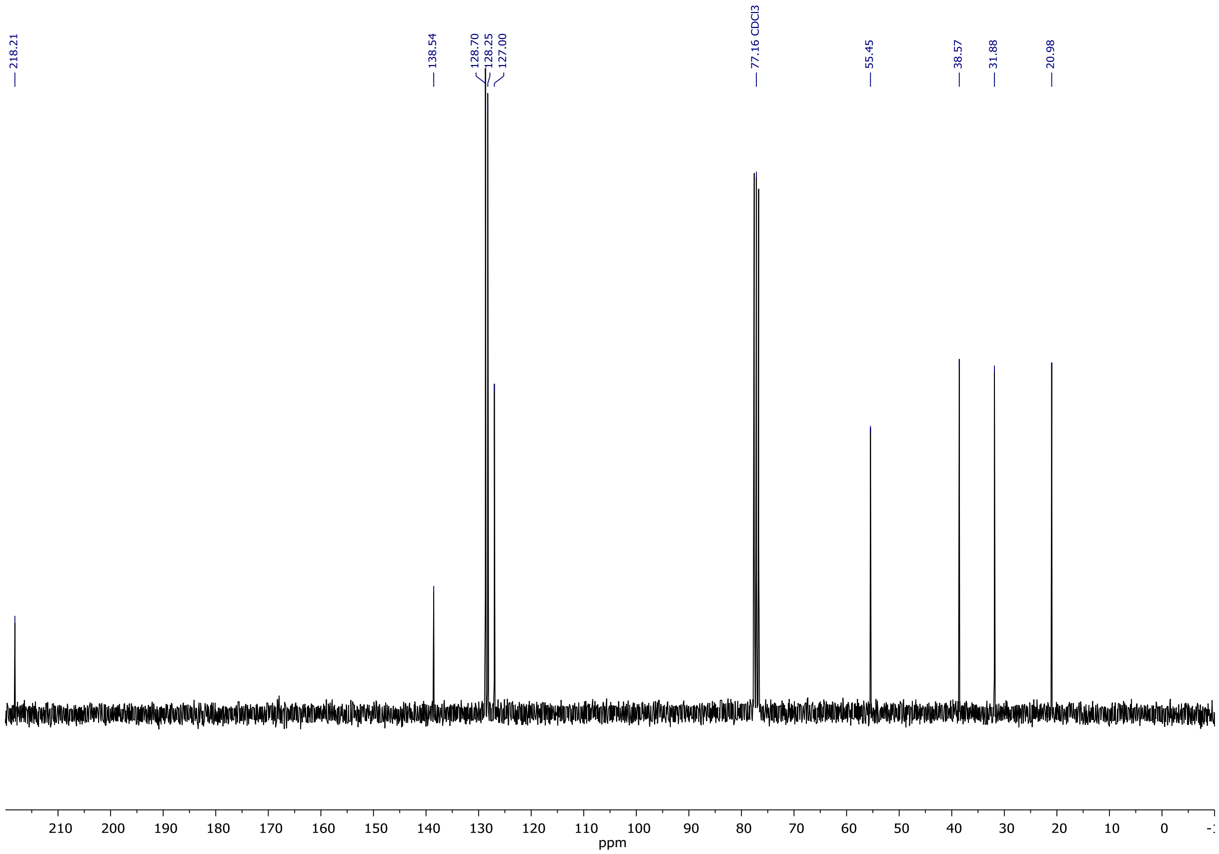

Compound **8b**, ^{1}HNMR and ^{13}C NMR ($CDCl_3$):

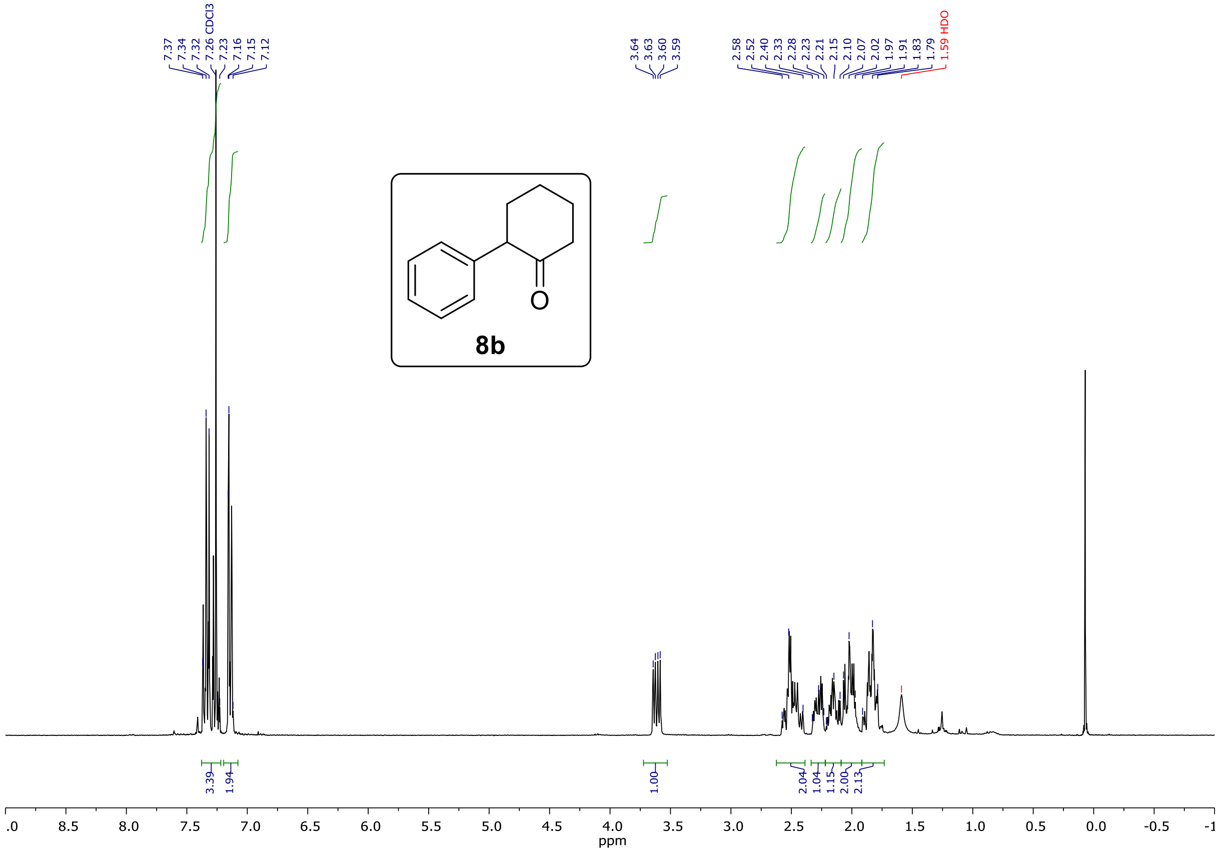

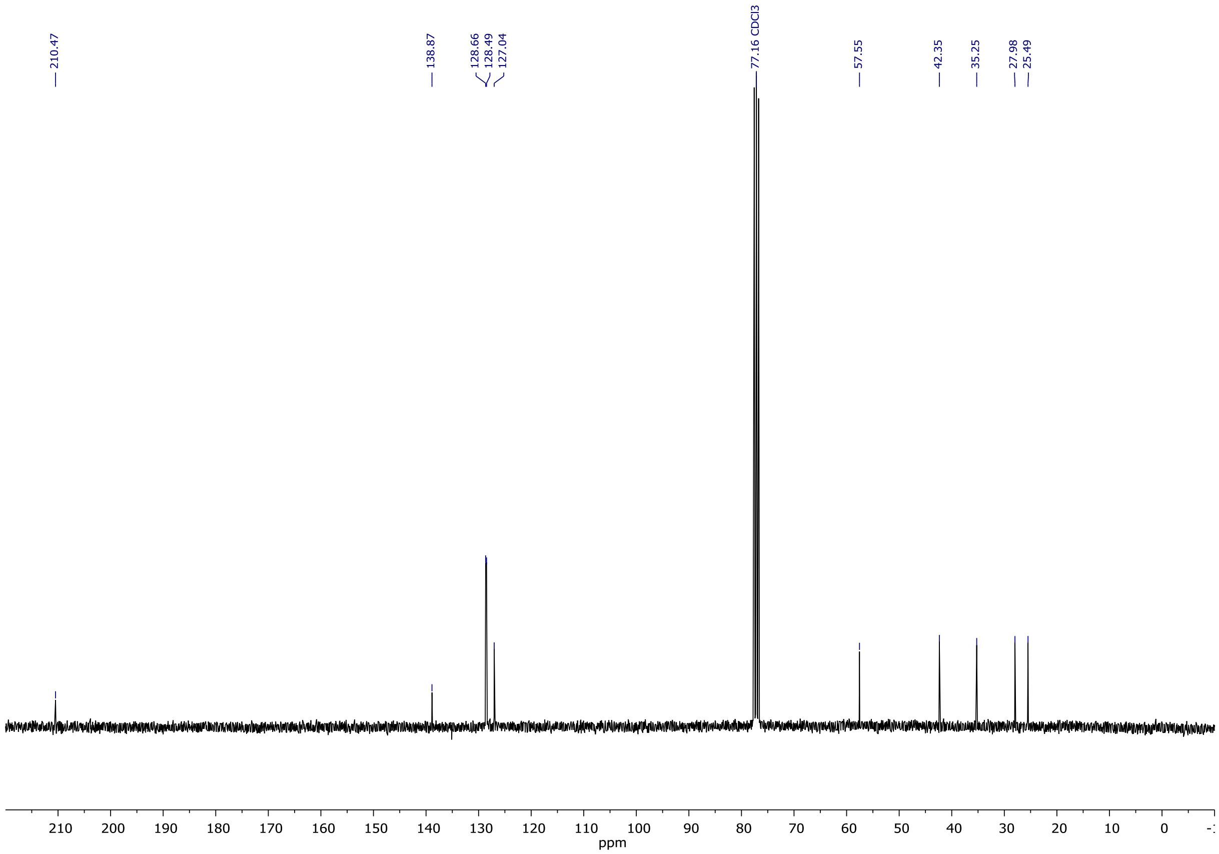

7. References

[1] S. Hünig, P. Kreitmeier, G. Märkl, J. Sauer, *Verlag Lehmanns* **2006**.
[2] R. K. Harris, E. D. Becker, S. M. Cabral de Menezes, R. Goodfellow, P. Granger, *Magn. Reson. Chem.* **2002**, *40*, 489-505.
[3] G. R. Fulmer, A. J. M. Miller, N. H. Sherden, H. E. Gottlieb, A. Nudelman, B. M. Stoltz, J. E. Bercaw, K. I. Goldberg, *Organometallics* **2010**, *29*, 2176-2179.
[4] V. V. Pavlishchuk, A. W. Addison, *Inorg. Chim. Acta* **2000**, *298*, 97-102.
[5] J. Luo, J. Zhang, *ACS Catal.* **2016**, *6*, 873-877.
[6] E. Speckmeier, T. G. Fischer, K. Zeitler, *J. Am. Chem. Soc.* **2018**, *140*, 15353-15365.
[7] Y. Du, R. M. Pearson, C. H. Lim, S. M. Sartor, M. D. Ryan, H. Yang, N. H. Damrauer, G. M. Miyake, *Chem. Eur. J.* **2017**, *23*, 10962-10968.
[8] R. M. Pearson, C. H. Lim, B. G. McCarthy, C. B. Musgrave, G. M. Miyake, *J. Am. Chem. Soc.* **2016**, *138*, 11399-11407.
[9] J. C. Theriot, C. H. Lim, H. Yang, M. D. Ryan, C. B. Musgrave, G. M. Miyake, *Science* **2016**, *352*, 1082-1086.
[10] A. B. Reitz, E. Sonveaux, R. P. Rosenkranz, M. S. Verlander, K. L. Melmon, B. B. Hoffman, Y. Akita, N. Castagnoli, M. Goodman, *J. Med. Chem.* **1985**, *28*, 634-642.
[11] S. Kathiravan, I. A. Nicholls, *Chem. Eur. J.* **2017**, *23*, 7031-7036.
[12] A. Fürstner, R. Martin, H. Krause, G. Seidel, R. Goddard, C. W. Lehmann, *J. Am. Chem. Soc.* **2008**, *130*, 8773-8787.
[13] D. A. Williams, S. A. Zaidi, Y. Zhang, *J. Nat. Prod.* **2015**, *78*, 1859-1867.
[14] T. Miura, Y. Funakoshi, J. Nakahashi, D. Moriyama, M. Murakami, *Angew. Chem. Int. Ed.* **2018**, *57*, 15455-15459.
[15] Y. Lu, D. Leow, X. Wang, K. M. Engle, J.-Q. Yu, *Chem. Sci.* **2011**, *2*, 967.
[16] J.-L. Zhu, H.-J. Liu, J.-P. Tsao, T.-Y. Tsai, I. C. Chen, S.-W. Tsao, *Synthesis* **2010**, *2010*, 4242-4250.
[17] D. J. Cram, H. L. Nyquist, F. A. A. Elhafez, *J. Am. Chem. Soc.* **1957**, *79*, 2876-2888.
[18] B. G. Ramsey, J. A. Cook, J. A. Manner, *J. Org. Chem.* **1972**, *37*, 3310-3322.
[19] X. Wang, Y. Lu, H. X. Dai, J. Q. Yu, *J. Am. Chem. Soc.* **2010**, *132*, 12203-12205.
[20] O. Piccolo, R. Menicagli, L. Lardicci, *Tetrahedron* **1979**, *35*, 1751-1758.
[21] M. Clarembeau, A. Krief, *Tetrahedron Lett.* **1985**, *26*, 1093-1096.
[22] M. T. Reetz, S. Stanchev, H. Haning, *Tetrahedron* **1992**, *48*, 6813-6820.
[23] S.-i. Fukuzawa, K. Mutoh, T. Tsuchimoto, T. Hiyama, *J. Org. Chem.* **1996**, *61*, 5400-5405.
[24] R. D. Rieke, R. M. Wehmeyer, T.-C. Wu, G. W. Ebert, *Tetrahedron* **1989**, *45*, 443-454.
[25] D. C. Moebius, J. S. Kingsbury, *J. Am. Chem. Soc.* **2009**, *131*, 878-879.
[26] M. Shibuya, M. Tomizawa, Y. Sasano, Y. Iwabuchi, *J. Org. Chem.* **2009**, *74*, 4619-4622.
[27] H. Tanaka, K. Sakai, A. Kawamura, K. Oisaki, M. Kanai, *Chem. Commun.* **2018**, *54*, 3215-3218.
[28] A. Narayanappa, D. Hurem, J. McNulty, *Synlett* **2017**, *28*, 2961-2965.
[29] L. A. Carpino, J. Xia, A. El-Faham, *J. Org. Chem.* **2004**, *69*, 54-61.
[30] J. A. Murphy, S. Z. Zhou, D. W. Thomson, F. Schoenebeck, M. Mahesh, S. R. Park, T. Tuttle, L. E. Berlouis, *Angew. Chem. Int. Ed.* **2007**, *46*, 5178-5183.
